# Homologous recombination DNA repair defects in *PALB2-*associated breast cancers

**DOI:** 10.1038/s41523-019-0115-9

**Published:** 2019-08-08

**Authors:** Anqi Li, Felipe C. Geyer, Pedro Blecua, Ju Youn Lee, Pier Selenica, David N. Brown, Fresia Pareja, Simon S. K. Lee, Rahul Kumar, Barbara Rivera, Rui Bi, Salvatore Piscuoglio, Hannah Y. Wen, John R. Lozada, Rodrigo Gularte-Mérida, Luca Cavallone, Zoulikha Rezoug, Tu Nguyen-Dumont, Paolo Peterlongo, Carlo Tondini, Thorkild Terkelsen, Karina Rønlund, Susanne E. Boonen, Arto Mannerma, Robert Winqvist, Marketa Janatova, Pathmanathan Rajadurai, Bing Xia, Larry Norton, Mark E. Robson, Pei-Sze Ng, Lai-Meng Looi, Melissa C. Southey, Britta Weigelt, Teo Soo-Hwang, Marc Tischkowitz, William D. Foulkes, Jorge S. Reis-Filho, kConFab Investigators, Morteza Aghmesheh, David Amor, Leslie Andrews, Yoland Antill, Rosemary Balleine, Jonathan Beesley, Anneke Blackburn, Michael Bogwitz, Melissa Brown, Matthew Burgess, Jo Burke, Phyllis Butow, Liz Caldon, Ian Campbell, Alice Christian, Christine Clarke, Paul Cohen, Ashley Crook, James Cui, Margaret Cummings, Sarah-Jane Dawson, Anna De Fazio, Martin Delatycki, Alex Dobrovic, Tracy Dudding, Pascal Duijf, Edward Edkins, Stacey Edwards, Gelareh Farshid, Andrew Fellows, Michael Field, James Flanagan, Peter Fong, John Forbes, Laura Forrest, Stephen Fox, Juliet French, Michael Friedlander, David Gallego Ortega, Michael Gattas, Graham Giles, Grantley Gill, Margaret Gleeson, Sian Greening, Eric Haan, Marion Harris, Nick Hayward, Ian Hickie, John Hopper, Clare Hunt, Paul James, Mark Jenkins, Rick Kefford, Maira Kentwell, Judy Kirk, James Kollias, Sunil Lakhani, Geoff Lindeman, Lara Lipton, Lizz Lobb, Sheau Lok, Finlay Macrea, Graham Mann, Deb Marsh, Sue-Anne McLachlan, Bettina Meiser, Roger Milne, Sophie Nightingale, Shona O’Connell, Nick Pachter, Briony Patterson, Kelly Phillips, Mona Saleh, Elizabeth Salisbury, Christobel Saunders, Jodi Saunus, Clare Scott, Rodney Scott, Adrienne Sexton, Andrew Shelling, Peter Simpson, Allan Spigelman, Mandy Spurdle, Jennifer Stone, Jessica Taylor, Heather Thorne, Alison Trainer, Georgia Trench, Kathy Tucker, Jane Visvader, Logan Walker, Mathew Wallis, Rachael Williams, Ingrid Winship, Kathy Wu, Mary Anne Young

**Affiliations:** 1 Department of Pathology, Memorial Sloan Kettering Cancer Center, New York, NY USA; 2 Department of Pathology, Fudan University Shanghai Cancer Center and Shanghai Medical College, Fudan University, Shanghai, P.R. China; 3 Radiation Oncology, Memorial Sloan Kettering Cancer Center, New York, NY USA; 4 Departments of Oncology and Human Genetics, McGill University, Montreal, Quebec Canada; 5 Cancer Axis, Lady Davis Institute, Jewish General Hospital, Montreal, Quebec Canada; 6 Institute of Pathology, University Hospital Basel, Basel, Switzerland; 7 Cancer Prevention Center, Jewish General Hospital, Montreal, Quebec Canada; 8 Genetic Epidemiology Laboratory, Department of Clinical Pathology, University of Melbourne, Parkville, Victoria, Australia; 9 Precision Medicine, School of Clinical Sciences at Monash Health, Monash University, Victoria, Australia; 10 IFOM, The Italian Foundation for Cancer Research Institute of Molecular Oncology, Milan, Italy; 11 Ospedale Papa Giovanni XXIII, Bergamo, Italy; 12 Department of Clinical Genetics, Aarhus University Hospital, Aarhus, Denmark; 13 Department of Clinical Genetics, Vejle Hospital, Vejle, Denmark; 14 Clinical Genetics Unit, Department of Pediatrics, Zealand University Hospital, Roskilde, Denmark; 15 Biocenter Kuopio and Cancer Center of Easter Finland, University of Eastern Finland, Kuopio, Finland; 16 Laboratory of Cancer Genetics and Tumor Biology, Cancer and Translational Medicine Research Unit, Biocenter Oulu, University of Oulu, Oulu, Finland; 17 Institute of Biochemistry and Experimental Oncology, First Faculty of Medicine, Charles University, Prague, Czech Republic; 18 Department of Pathology, Subang Jaya Medical Centre, Subang Jaya, Selangor Malaysia; 19 Department of Radiation Oncology, Rutgers Cancer Institute of New Jersey, New Brunswick, NJ USA; 20 Department of Medicine, Memorial Sloan Kettering Cancer Center, New York, NY USA; 21 Cancer Research Malaysia, Subang Jaya, Malaysia; 22 Department of Pathology, Faculty of Medicine, University Malaya, Kuala Lumpur, Malaysia; 23 University Malaya Cancer Research Institute, Faculty of Medicine, University Malaya, Kuala Lumpur, Malaysia; 24 Department of Medical Genetics, University of Cambridge, Cambridge, UK; 25 Cancer Program, Research Institute McGill University Health Centre, Montreal, Quebec Canada; 26 Illawarra Cancer Care Centre, Wollongong Hospital, Wollongong, NSW 2500 Australia; 27 Genetic Health Services Victoria, Royal Children’s Hospital, Melbourne, VIC, 3050 Australia; 28 Prince of Wales Hospital, Randwick, NSW 2031 Australia; 29 The Family Cancer Clinic, Cabrini Hospital, Malvern, VIC 3144 Australia; 30 Department of Medical Oncology, Westmead Hospital, Westmead, NSW 2145 Australia; 31 Queensland Institute of Medical Research, Herston Qld, 4002 Australia; 32 Australian National University, Canberra ACT, Canberra, 2601 Australia; 33 Familial Cancer Centre, The Royal Melbourne Hospital, Parkville, VIC 3050 Australia; 34 University of Queensland, St. Lucia, QLD 4072 Australia; 35 Clinical Genetics Service, Austin Health, Vic, 3084 Australia; 36 Royal Hobart Hospital, Hobart, Tasmania 7001 Australia; 37 Medical Psychology Unit, Royal Prince Alfred Hospital, Camperdown NSW, 2204 Australia; 38 Garvan Institute of Medical Research, Darlinghurst, NSW 2010 Australia; 39 Cancer Genetics Laboratory, Peter MacCallum Cancer Centre, Melbourne, VIC 3000 Australia; 40 Genetics Department, Central Region Genetics Service, Wellington Hospital, Wellington, 6021 New Zealand; 41 Westmead Institute for Cancer Research, University of Sydney, Westmead Hospital, Westmead, NSW 2145 Australia; 42 Gynaecological Cancer Research, St John of God Subiaco Hospital, Subiaco, WA 6008 Australia; 43 Department of Clinical Genetics, Royal North Shore Hospital, St Leonards, NSW 2065 Australia; 44 Epidemiology and Preventive Medicine, Monash University, Prahan, Vic 3004 Australia; 45 Department of Pathology, University of Queensland Medical School, Herston, Qld 4006 Australia; 46 Sir Peter MacCallum Department of Oncology, Melbourne, VIC 3000 Australia; 47 Department of Gynaecological Oncology, Westmead Institute for Cancer Research, Westmead Hospital, Westmead, NSW 2145 Australia; 48 Hunter Genetics, Hunter Area Health Service, Waratah, 2298 NSW Australia; 49 The University of Queensland Diamantina Institute, Brisbane, QLD 4102 Australia; 50 Clinical Chemistry, Princess Margaret Hospital for Children, Perth, WA 6001 Australia; 51 SA Tissue Pathology, IMVS, Adelaide, SA 5000 Australia; 52 Epigenetics Unit, Department of Surgery and Oncology, Imperial College London, London, W12 0NN UK; 53 Regional Cancer and Blood Services, Auckland City Hospital, Auckland, 1023 New Zealand; 54 Surgical Oncology, University of Newcastle, Newcastle Mater Hospital, Waratah, NSW 2298 Australia; 55 Psychosocial Cancer Genetics Research Group, Parkville Familial Cancer Centre, Melbourne, Vic 3000 Australia; 56 School of Molecular and Microbial Sciences, University of Queensland, St Lucia, Qld 4072 Australia; 57 Department of Medical Oncology, Prince of Wales Hospital, Randwick, NSW 2031 Australia; 58 Tumour Development Group, Garvan Institute of Medical Research, The Kinghorn Cancer Centre, Darlinghurst, NSDW 2010 Australia; 59 Queensland Clinical Genetic Service, Royal Children’s Hospital, Herston, QLD 4020 Australia; 60 Anti-Cancer Council of Victoria, Melbourne, VIC 3004 Australia; 61 Department of Surgery, Royal Adelaide Hospital, Adelaide, 5000 Australia; 62 Hunter Family Cancer Service, Waratah, NSW 2298 Australia; 63 Department of Medical Genetics, Women’s and Children’s Hospital, North Adelaide, SA 5006 Australia; 64 Family Cancer Clinic, Monash Medical Centre, Clayton, 3168 Australia; 65 Brain and Mind Centre, Camperdown, NSW 2050 Australia; 66 Centre for M.E.G.A. Epidemiology, University of Melbourne, Carlton, VIC 3010 Australia; 67 Familial Cancer Service, Department of Medicine, Westmead Hospital, Westmead, NSW 2145 Australia; 68 Breast Endocrine and Surgical Unit, Royal Adelaide Hospital, North Terrace, SA 5000 Australia; 69 University of Queensland, The Royal Brisbane & Women’s Hospital, Herston, QLD 4029 Australia; 70 Walter and Eliza Hall Institute, PO Royal Melbourne, Hospital, Parkville, VIC 3050 Australia; 71 Medical Oncology and Clinical Haematology Unit, Western Hospital, Footscray, VIC 3011 Australia; 72 School of Medicine, University of Notre Dame, Kogarah, NSW 2217 Australia; 73 Department of Medical Oncology, The Royal Melbourne Hospital, Parkville, Vic 3050 Australia; 74 Kolling Institute of Medical Research, Royal North Shore Hospital, St Leonards, NSW 2065 Australia; 75 Department of Oncology, St Vincent’s Hospital, Fitzroy, VIC 3065 Australia; 76 Department of Surgery, Peter MacCallum Cancer Centre, Melbourne, VIC 3000 Australia; 77 Genetic Services of WA, King Edward Memorial Hospital, Subiaco, WA 6008 Australia; 78 Centre for Genetic Education, Prince of Wales Hospital, Randwick, NSW 2031 Australia; 79 Anatomical Pathology, Prince of Wales Hospital, Randwick, 2031 NSW Australia; 80 School of Surgery and Pathology, QE11 Medical Centre, Nedlands, WA 6907 Australia; 81 Breast Pathology, University of Queensland Centre for Clinical Research, Royal Brisbane and Women’s Hospital, Herston, Qld 4029 Australia; 82 Hunter Area Pathology Service, John Hunter Hospital, NSW, 2310 Australia; 83 Obstetrics and Gynaecology, University of Auckland, Auckland, 1023 New Zealand; 84 Family Cancer Clinic, St Vincent’s Hospital, Darlinghurst, NSW 2010 Australia; 85 Centre for Genetic Origins of Health and Disease, University of Western Australia, Crawley, WA 6009 Australia; 86 Department of Pathology, University of Otago, Christchurch, 8011 New Zealand; 87 Department of Medicine, Royal Melbourne Hospital, Parkville, VIC 3050 Australia

**Keywords:** Breast cancer, Cancer genetics, Cancer genomics

## Abstract

Mono-allelic germline pathogenic variants in the *Partner And Localizer of BRCA2* (*PALB2*) gene predispose to a high-risk of breast cancer development, consistent with the role of PALB2 in homologous recombination (HR) DNA repair. Here, we sought to define the repertoire of somatic genetic alterations in *PALB2*-associated breast cancers (BCs), and whether *PALB2*-associated BCs display bi-allelic inactivation of *PALB2* and/or genomic features of HR-deficiency (HRD). Twenty-four breast cancer patients with pathogenic *PALB2* germline mutations were analyzed by whole-exome sequencing (WES, *n* = 16) or targeted capture massively parallel sequencing (410 cancer genes, *n* = 8). Somatic genetic alterations, loss of heterozygosity (LOH) of the *PALB2* wild-type allele, large-scale state transitions (LSTs) and mutational signatures were defined. *PALB2*-associated BCs were found to be heterogeneous at the genetic level, with *PIK3CA* (29%), *PALB2* (21%), *TP53* (21%), and *NOTCH3* (17%) being the genes most frequently affected by somatic mutations. Bi-allelic *PALB2* inactivation was found in 16 of the 24 cases (67%), either through LOH (*n* = 11) or second somatic mutations (*n* = 5) of the wild-type allele. High LST scores were found in all 12 *PALB2*-associated BCs with bi-allelic *PALB2* inactivation sequenced by WES, of which eight displayed the HRD-related mutational signature 3. In addition, bi-allelic inactivation of *PALB2* was significantly associated with high LST scores. Our findings suggest that the identification of bi-allelic *PALB2* inactivation in *PALB2*-associated BCs is required for the personalization of HR-directed therapies, such as platinum salts and/or PARP inhibitors, as the vast majority of *PALB2*-associated BCs without *PALB2* bi-allelic inactivation lack genomic features of HRD.

## Introduction

The Partner And Localizer of BRCA2 (PALB2) is a key protein that interacts with BRCA1 and BRCA2 and plays pivotal roles in homologous recombination (HR) DNA repair.^1^ Bi-allelic *PALB2* germline mutations (i.e., affecting both parental alleles of *PALB2*) cause Fanconi anemia,^2^ whereas mono-allelic *PALB2* germline mutations result in increased risk of breast, pancreatic and ovarian cancer.^3–5^ The frequency of *PALB2* germline mutations in familial breast cancer ranges from 0.6% to 2.7%,^4^ and the average cumulative breast cancer risk in *PALB2* germline mutation carriers by the age of 70 years is ~35%,^4^ similar to that conferred by *BRCA2* germline mutations.^6^ Akin to sporadic and *BRCA2* breast cancers, *PALB2*-associated breast cancers are heterogeneous in terms of their clinicopathologic features, being predominantly estrogen receptor (ER)-positive.^4^ As compared to non-*PALB2* mutation carriers, patients with *PALB2* germline mutations have been reported to display a shorter 10-year survival.^7^ Consistent with the role of PALB2 in HR DNA repair, PALB2-deficient cells have been shown to be sensitive to platinum-based chemotherapy and poly (ADP-ribose) polymerase (PARP) inhibitors;^8,9^ hence, therapies targeting HR deficiency (HRD) may benefit breast cancer patients with *PALB2* germline mutations.^10^

Although *PALB2* constitutes a tumor suppressor gene, there is controversy as to whether it follows the Knudson two-hit model.^11,12^ A recent study revealed that ten of 15 *PALB2*-associated breast cancers harbored bi-allelic *PALB2* inactivation through somatic loss of heterozygosity (LOH) of the *PALB2* wild-type allele (*n* = 6) or somatic *PALB2* mutations (*n* = 4).^12^ *PALB2* promoter hypermethylation has been reported in tumors from sporadic and *BRCA1/2* mutation-negative familial breast and ovarian cancers,^13^ however, it appears to be vanishingly rare in tumors from *PALB2* germline mutation carriers.^12,14^ Germane to the understanding of the biology of *PALB2*-associated breast cancers and to the identification of optimal therapeutic approaches for patients with *PALB2* germline mutations is to ascertain the mechanisms that contribute to bi-allelic *PALB2* inactivation, and to define whether *PALB2*-associated breast cancers without bi-allelic inactivation lack genomic features consistent with HRD (e.g., large-scale state transitions (LSTs) and mutational signatures). Importantly, Lee et al.,^12^ based on a targeted capture sequencing analysis of 487 genes, reported that, with one exception, *PALB2*-associated breast cancers that retained the *PALB2* wild-type allele displayed HRD scores consistent with those of tumors harboring *PALB2* bi-allelic inactivation.

Here we sought to characterize the repertoire of somatic genetic alterations of breast cancers from pathogenic *PALB2* germline mutation carriers using a combination of whole-exome and targeted massively parallel sequencing to define whether bi-allelic *PALB2* inactivation is present in these tumors. Based on whole-exome sequencing (WES) results, we employed validated approaches to determine whether the genomic hallmark features of HRD^15^ are present in *PALB2*-associated breast cancers irrespective of the presence of a second hit affecting *PALB2*. Moreover, as an exploratory, hypothesis-generating analysis, we compared the genomic landscape of breast cancers from pathogenic *PALB2* germline mutation carriers to that of breast cancers arising in *BRCA1* or *BRCA2* germline mutation carriers, and non-*BRCA1/2/PALB2*-associated breast cancers.

## Results

### Clinicopathologic features of *PALB2*-associated breast cancers

Twenty-four invasive breast cancers from carriers of fourteen distinct pathogenic *PALB2* germline mutations^4,9,16–18^ were included in this study. Fourteen cases were subjected to WES and WES sequencing data from two cases were retrieved from TCGA^19^ (*n* = 16; median depth of tumor 112 × (range 33 × −289 ×) and normal 129 × (range 46 × −247 ×) samples). In addition, 8 cases were analyzed by targeted capture massively parallel sequencing using the Memorial Sloan Kettering-Integrated Mutation Profiling of Actionable Cancer Targets (MSK-IMPACT) sequencing assay^20^ (median depth of tumor 232 × (range 73 × −904 ×), and normal 545 × (range 172 × −1452 ×) samples; Table 1, Supplementary Table 1). All samples included in this study were derived from formalin-fixed paraffin-embedded (FFPE) material. Sample quality was evaluated and was found to be appropriate for the analyses conducted (Supplementary Table 2). All but one *PALB2* germline mutations were bona fide loss-of-function (frameshift or truncating) mutations; one case carried a missense substitution (L35P), which we have previously demonstrated to be pathogenic.^9^

**Table 1:** Clinicopathologic features and sequencing results of the 24 *PALB2*-associated breast cancers studied

Case ID	Age (years)	Tumor size (cm)	Grade	ER	HER2	*PALB2* germline mutation	Germline mutation type	Sequencing type	Somatic mutations (n)	Non-synonymous somatic mutations (n)	*PALB2* bi-allelic inactivation	LST score*	Mutational signature
IDC4	47	1.9	2	+	−	c.3504_3505delCT (His1170Phefs)	Frameshift	WES	109	76	LOH	High	Signature 3
IDC51	82	4	3	+	−	c.1037_1041delAAGAA (Lys346Thrfs)	Frameshift	WES	189	131	LOH	High	Signature 3
IDC60	49	NA	2	+	−	c.424 A > T (Lys142*)	Truncating	WES	141	103	LOH	High	Signature 3
IDC61	53	NA	3	−	−	c.509_510delGA (Arg170fs)	Frameshift	WES	118	86	LOH	High	Signature 3
IDC55	33	1.2	3	−	−	c.3116delA (Asn1039Ilefs)	Frameshift	WES	138	105	LOH	High	Signature 3
IDC59	49	1.1	2	+	−	c.2323 C > T (Gln775*)	Truncating	WES	92	72	LOH	High	Signature 3
IDC8	42	6	2	+	−	c.2323 C > T (Gln775*)	Truncating	WES	63	41	LOH	High	Unstable
IDC9	29	1.2	2	+	−	c.2521delA (Thr841Glnfs)	Frameshift	WES	250	161	LOH	High	Signature 1
IDC15	54	2	3	+	−	c.1592delT (Leu531Cysfs)	Frameshift	WES	215	155	LOH	High	Signature 1
IDC35	64	2.4	3	+	−	c.3113 G > A (Trp1038*)	Truncating	IMPACT	8	8	LOH	N/A	N/A
IDC33	49	0.48	3	+	−	c.3113 G > A (Trp1038*)	Truncating	IMPACT	9	6	LOH	N/A	N/A
IDC53	32	NA	2	+	−	c.104 T > C (Leu35Pro)	Missense	WES	269	195	p.Gln61*	High	Signature 3
IDC50	64	2	3	+	−	c.1037_1041delAAGAA (Lys346Thrfs)	Frameshift	WES	220	147	p.Gln921Argfs	High	Signature 3
IDC24	51	1.2	2	+	−	c.3113 G > A (Trp1038*)	Truncating	WES	73	54	p.Gln479*	High	Signature 1
IDC62	52	1.9	3	+	−	c.172_175delTTGT (Gln60Argfs)	Frameshift	IMPACT	11	10	p.Q822*	N/A	N/A
IDC52	44	3	2	+	−	c.1783delG (Glu584Lysfs)	Frameshift	IMPACT	7	5	p.Tyr79fs	N/A	N/A
IDC37	61	0.15	2	+	−	c.3113 G > A (Trp1038*)	Truncating	WES	99	79	No	Low	Signature 3
IDC46	38	NA	3	−	−	c.1037_1041delAAGAA (Lys346Thrfs)	Frameshift	WES	107	73	No	High	Signature 1
IDC28	47	0.7	2	+	−	c.3113 G > A (Trp1038*)	Truncating	WES	109	67	No	Low	Signature 1
IDC3	49	1.6	3	+	+	c.3504_3505delCT (His1170Phefs)	Frameshift	WES	59	37	No	Low	Unstable
IDC49	30	1.5	1	+	−	c.1059delA (Lys353Asnfs)	Frameshift	IMPACT	5	4	No	N/A	N/A
IDC19	38	NA	2	+	−	c.172_175delTTGT (Gln60Argfs)	Frameshift	IMPACT	4	4	No	N/A	N/A
IDC13	54	1	3	−	−	c.1592delT (Leu531Cysfs)	Frameshift	IMPACT	9	6	No	N/A	N/A
IDC63	48	3.7	2	−	−	c.2488delG (Glu830Serfs)	Frameshift	IMPACT	3	1	No	N/A	N/A

LSTs and mutational signatures could only be defined in tumors subjected to WES

*Age* age at diagnosis, *ER* estrogen receptor, *IMPACT* MSK-integrated mutation profiling of actionable cancer targets, *LST* large-scale state transition, *N/A* not assessable, *WES* whole-exome sequencing

*LST high, ≥15; LST low, <15

The median age at breast cancer diagnosis was 49 years (range 29–82 years), and the median tumor size was 1.6 cm (range 0.15–6 cm; Table 1). All *PALB2*-associated breast cancers were invasive ductal carcinomas of no special type, and one, 12, and eleven cases were of histologic grades 1, 2, and 3, respectively (Table 1). Eighteen cases (75%) were ER-positive/HER2-negative (ER+/HER2−), five (21%) were ER-negative/HER2-negative (ER−/HER2−), and one (4%) was ER-positive/HER2-positive (ER+/HER2+; Table 1). Whilst the distribution of *PALB2*-associated breast cancers into ER/HER2 clinical subgroups was found to be comparable to that of sporadic and *BRCA2* breast cancers,^19,21^ it differed from that of *BRCA1* breast cancers, which are preferentially of triple-negative phenotype (approximately 70%–85%).^21–23^

### Repertoire of somatic genetic alterations in *PALB2*-associated breast cancers

Somatic mutation analysis of the WES data of 16 *PALB2*-associated breast cancers revealed a median of 113.5 (range 59–269) somatic mutations per case, of which 82.5 (range 37–195) were non-synonymous. The eight *PALB2*-associated breast cancers analyzed by MSK-IMPACT displayed a median of 3 (range 0–5) somatic mutations per case, of which 2 (range 0–4) were non-synonymous (Table 1, Supplementary Table 3). Selected somatic mutations (*n* = 17) were validated by Sanger sequencing (Supplementary Fig. 1).

**Fig. 1:**
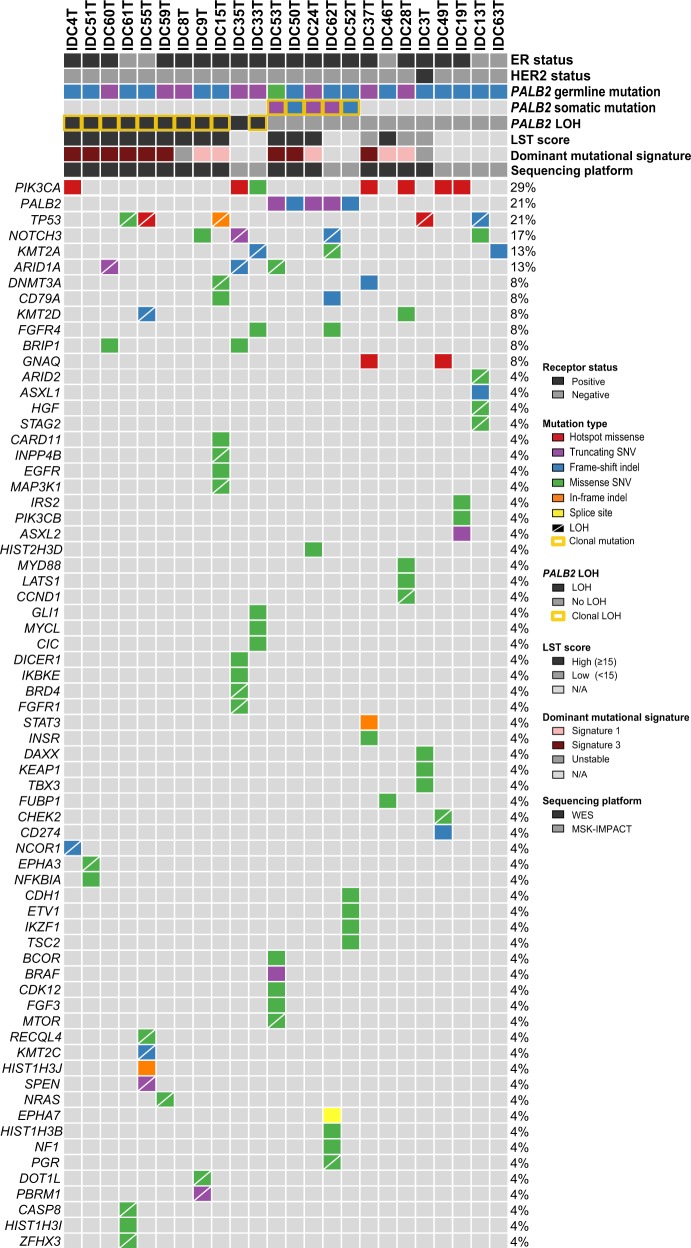
Non-synonymous somatic mutations in *PALB2*-associated breast cancers. Heatmap depicting the somatic genetic alterations identified in the 24 *PALB2*-associated breast cancers analyzed by whole-exome (*n* = 16) or targeted MSK-IMPACT (*n* = 8) massively parallel sequencing. Somatic mutations affecting the 410 cancer genes present in MSK-IMPACT, in decreasing overall mutational frequency observed in *PALB2*-associated breast cancers are plotted. Cases are shown in columns, and genes in rows. Estrogen receptor (ER) and HER2 status, *PALB2* germline mutation type, presence of a second somatic *PALB2* mutation or loss of heterozygosity (LOH) of the *PALB2* wild-type allele, large-scale state transition (LST) score, dominant mutational signature and sequencing platform are indicated in the phenobar (top), color-coded according to the legend. Note that mutational signatures and LST scores could not be assessed in tumors subjected to MSK-IMPACT sequencing due to the limited number of mutations present. Clonal somatic *PALB2* mutations or clonal LOH of the *PALB2* wild-type allele are indicated by yellow boxes. Somatic mutations are color-coded according to the legend, and LOH of the wild-type allele of mutated genes other than *PALB2* is represented by a diagonal bar. *Indel* small insertion/deletion; *LOH* loss of heterozygosity, *LST* large-scale state transition, *N/A* not assessable, *SNV* single nucleotide variant, *WES* whole-exome sequencing

Of the 410 cancer genes included in MSK-IMPACT, recurrently mutated genes found in the 24 *PALB2*-associated breast cancers included *PIK3CA* (*n* = 7, 29%), *PALB2* (*n* = 5, 21%), *TP53* (*n* = 5, 21%), *NOTCH3* (*n* = 4, 17%), *KMT2A* (*n* = 3, 17%) and *ARID1A* (*n* = 3, 17%; Fig. 1, Supplementary Table 3). Six *PIK3CA* missense mutations affected hotspot residues, including H1047R (*n* = 3), E545K (*n* = 1), N345K (*n* = 1) and C420R (*n* = 1), and four were predicted to be clonal (Supplementary Fig. 2, Supplementary Table 3). All but one somatic *TP53* mutation, all coupled with loss of heterozygosity (LOH) of the *TP53* wild-type allele, were predicted to be clonal (Supplementary Fig. 2, Supplementary Table 3). Additional recurrently mutated genes detected in the 16 *PALB2*-associated breast cancers profiled by WES included *CTNNA2*, *TMPRSS13*, *KRTAP4–11, LAMA5*, *KALRN*, and *COLL22A1* (all, *n* = 3; Supplementary Fig. 3, Supplementary Table 3).

**Fig. 2:**
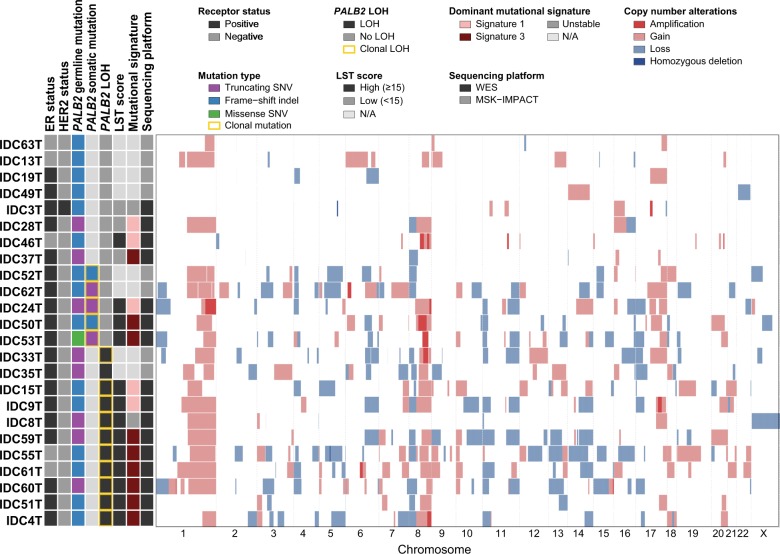
Repertoire of copy number alterations in *PALB2*-associated breast cancers. Copy number alterations in the 24 *PALB2*-associated breast cancers analyzed by whole-exome (*n* = 16) or targeted MSK-IMPACT (*n* = 8) massively parallel sequencing. Cases are represented in rows and chromosomes in columns along the *x*-axis. Immunohistochemical features, *PALB2* germline mutation type, presence of a somatic *PALB2* mutation or loss of heterozygosity (LOH) of the *PALB2* wild-type allele, large-scale state transition (LST) score, mutational signature and sequencing platform are provided in the phenobar (left), color-coded according to the legend. Dark red, amplification; light red, copy number gain; dark blue, homozygous deletion; light blue, copy number loss; white, no change. *LOH* loss of heterozygosity, *LST* large-scale state transition; *N/A* not assessable, *SNV* single nucleotide variant, *WES* whole-exome sequencing

Copy number (CN) analysis revealed recurrent gains of 1q, 8q, 16p, 17q, and 20q, and losses of 1p, 4p, 8p, 11p, and 17p in the 24 *PALB2*-associated breast cancers analyzed (Fig. 2). Although the majority (*n* = 18) of cases were ER+/HER2−, concurrent 1q gains and 16q losses, the hallmark features of luminal breast cancers,^19^ were only found in four cases. Loci recurrently amplified included those mapping to 8q21.3 (encompassing the locus of *NBN*, *n* = 5) and 8q24.21 (encompassing the locus of *MYC*, *n* = 5; Fig. 2).

### Bi-allelic *PALB2* inactivation

Bi-allelic *PALB2* inactivation was found in 16 of the 24 *PALB2*-associated breast cancers (67%; Table 1, Fig. 1). In eleven cases, the second hit was in the form of LOH of the *PALB2* wild-type allele, whereas in five tumors, it was in the form of an inactivating (i.e., truncating or frameshift) somatic *PALB2* mutation. Fifteen of the 16 somatic genetic events leading to bi-allelic inactivation of the *PALB2* wild-type allele were predicted to be clonal (Fig. 1, Supplementary Table 3), suggesting that bi-allelic *PALB2* inactivation and subsequent complete loss-of-function of PALB2 may constitute an early somatic event in the development of a subset of *PALB2*-associated breast cancers.

### *PALB2*-associated breast cancers with bi-allelic inactivation display genomic features consistent with HRD

**Fig. 3:**
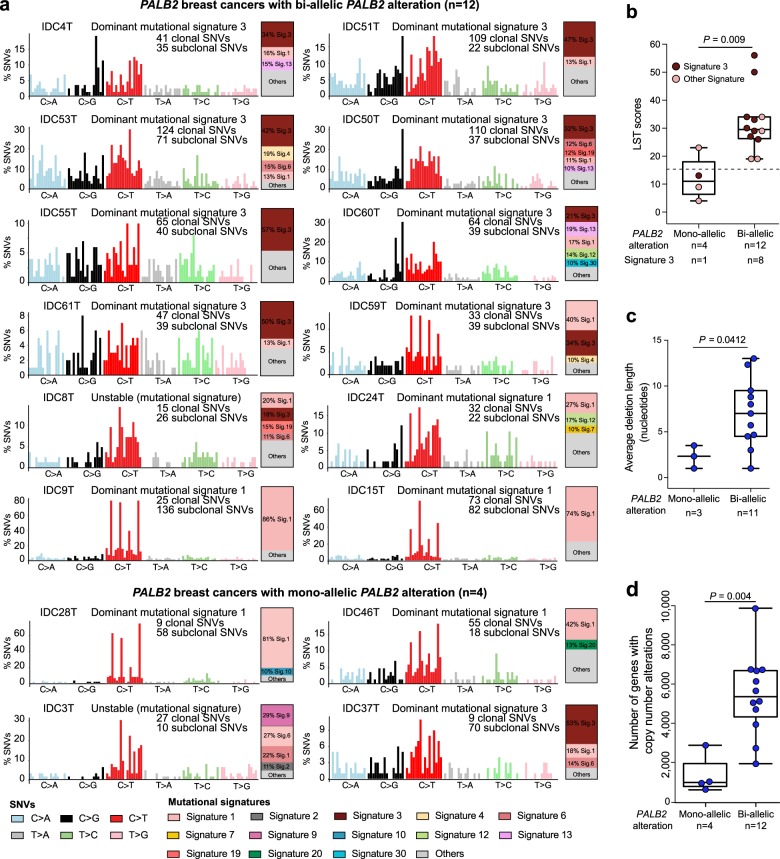
HRD genomic features in breast cancers with and without bi-allelic *PALB2* inactivation. **a** Mutational signatures of all somatic SNVs in the 16 *PALB*2-associated breast cancers sequenced by whole-exome sequencing (left) as inferred by deconstructSigs^41^ based on the 30 signatures represented in COSMIC, and a bar plot indicating the proportion of the major mutational signatures identified in each case (right), in decreasing proportion of each signature. The dominant mutational signatures were assigned according to Alexandrov et al.,^40^ following the consensus of at least two of three approaches (deconstructSigs based on 30 signatures from COSMIC, based on the 12 signatures known to occur in breast cancer, and NMF method^42^ based on 30 signatures from COSMIC) where signature 1 relates to aging and signature 3 to defective homologous recombination DNA repair, and are shown for cases with bi-allelic *PALB2* alterations (top) and mono-allelic *PALB2* alterations (bottom). The number of SNVs is shown in parentheses. Sig signature, SNV single nucleotide variant. **b** Large-scale state transition (LST) scores of the four *PALB2*-associated breast cancers with mono-allelic *PALB2* alterations and the 12 *PALB2*-associated breast cancers with bi-allelic *PALB2* alterations. The median LST scores, and the 75th and 25th percentiles are displayed at the top and bottom of the boxes, respectively. Each dot corresponds to the LST score and the mutational signature of a given case. Dominant mutational signatures are color-coded according to the legend. Comparisons of LST scores between groups were performed using the Mann–Whitney *U* test. **c** Average deletion length (nucleotides) in *PALB2*-associated breast cancers with mono-allelic *PALB2* alterations (*n* = 3) and with bi-allelic *PALB2* alterations (*n* = 11). Only *PALB2*-associated breast cancers harboring small insertions and deletions were included in the analysis. The median value of deletion length, and the 75th and 25th percentiles are displayed at the top and bottom of the boxes, respectively. Comparisons of deletion lengths between groups were performed using the Mann–Whitney *U* test. **d** Number of genes affected by copy number alterations (CNAs) of the four *PALB2*-associated breast cancers with mono-allelic *PALB2* alterations and the 12 *PALB2*-associated breast cancers with bi-allelic *PALB2* alterations. The median value of the number of genes with CNAs, and the 75th and 25th percentiles are displayed at the top and bottom of the boxes, respectively. Comparisons were performed using Fisher’s exact test

We^15^ and others^24^ have demonstrated that bi-allelic inactivation but not mono-allelic alterations of HR-related genes are associated with genomic features consistent with HRD. Hence, we sought to define whether LST scores and dominant mutational signature 3 would be associated with bi-allelic *PALB2* inactivation. LST scores and mutational signatures were inferred in the 16 *PALB2*-associated breast cancers analyzed by WES, of which 13 cases were found to display high LST scores (LST^high^), and nine cases (eight LST^high^ and one LST^low^) were found to have a dominant mutational signature 3 associated with HRD (i.e., BRCA1/2 signature; Table 1, Figs. 1 and 3a). No significant association between *PALB2* germline mutation types and HRD-related genomic features was observed (*P* > 0.05; Table 1).

Bi-allelic *PALB2* inactivation was significantly associated with LST^high^ (1/4 vs. 12/12, *P* = 0.0071, Fisher’s exact test; *P* = 0.009, Mann–Whitney *U* test; Fig. 3b), in agreement with the findings that bi-allelic inactivation rather than mono-allelic alterations of HR-related genes is associated with HRD-related genomic features.^15,24^ All but one (IDC37) cases displaying mutational signature 3 and all but one case (IDC46) displaying high LST scores were found to harbor bi-allelic *PALB2* inactivation (Table 1, Fig. 1 and Fig. 3b). Notably, we did not identify pathogenic germline mutations, bi-allelic or mono-allelic somatic mutations or homozygous deletions affecting other HRD-related genes^15^ in IDC37 or IDC46. Alternative mechanisms of inactivation of the wild-type allele of *PALB2*, such as complex *PALB2* rearrangements or, less likely *PALB2* promoter hypermethylation,^12^ which are not detectable by WES, or other mechanisms that result in HRD may be operative in IDC37 and IDC46. Four of the 12 *PALB2*-breast cancers with bi-allelic *PALB2* inactivation subjected to WES lacked a dominant signature 3, despite displaying high LST scores (Fig. 1). One could hypothesize that these cases could correspond to sporadic breast cancers arising in *PALB2* germline mutation carriers, in which the second *PALB2* allele was inactivated later in tumor evolution. Two of the 12 *PALB2*-associated breast cancers analyzed by WES lacked both evidence of bi-allelic *PALB2* inactivation and genomic features of HRD (Table 1, Fig. 1). One could posit that these invasive breast cancers may constitute non-*PALB2*-related cancers arising in the context of a *PALB2* germline mutation.

As an exploratory, hypothesis-generating analysis, we compared the genomic profiles of invasive breast cancers developing in the context of pathogenic *PALB2* germline mutations with or without loss of the *PALB2* wild-type allele. Among the 16 *PALB2*-associated breast cancers analyzed by WES, the 12 cases with bi-allelic *PALB2* inactivation harbored a numerically higher somatic mutation rate (median 139.5, range 63–269) than the four cases without bi-allelic inactivation (median 103, range 59–109; *P* *=* 0.09). Moreover, in *PALB2*-associated breast cancers analyzed by WES and harboring indels (*n* = 14), the average deletion length was significantly longer in cases with bi-allelic *PALB2* inactivation (*n* = 11) as compared to those with mono-allelic alterations (*n* = 3; 7 bp vs. 2.3 bp; *P* = 0.041; Fig. 3c), a feature associated with HRD.^25^ We further found a significantly higher number of copy number alterations (CNAs) in the tumors with bi-allelic *PALB2* inactivation (*n* = 12) than in those without (*n* = 4; *P* *=* 0.004; Fig. 3d). These results suggest that *PALB2*-associated breast cancers with bi-allelic inactivation, display higher levels of genetic instability, which may potentially be associated with the early onset of HRD in their development.

Lastly, upon combining the *PALB2*-associated breast cancers reported by Lee et al.^12^ with the cases analyzed here, we observed that 67% (26/39) of *PALB2*-associated breast cancers harbored bi-allelic *PALB2* inactivation (Supplementary Table 4). Consistently, bi-allelic *PALB2* inactivation was significantly associated with a high LST score, whilst no significant association was observed between bi-allelic *PALB2* inactivation and clinicopathologic characteristics (*P* > 0.05; Supplementary Table 4).

### *PALB2*-associated breast cancers with bi-allelic inactivation display higher mutation burden and HRD-associated features more frequently than sporadic breast cancers

As an exploratory, hypothesis-generating analysis we investigated whether *PALB2*-associated breast cancers would differ from non-*BRCA1/2/PALB2*-associated breast cancers from TCGA.^19^ Given that none of the *PALB2*-associated breast cancers included here was of ER-/HER2+ phenotype, ER-/HER2+ non-*BRCA1/2/PALB2*-associated breast cancers from TCGA were excluded, and the remaining 683 ER−/HER2− and ER+ (including ER+/HER2+and ER+/HER2−) breast cancers were employed for the analyses. The 16 *PALB2*-associated breast cancers analyzed by WES were found to harbor a higher number of somatic mutations (median 113.5, range 59–269) than the 683 ER−/HER2− and ER+non-*BRCA1/2/PALB2*-associated breast cancers (median 51, range 2–6666; *P* < 0.002, Mann–Whitney *U* test), difference that remained significant upon 1:3 bootstrap resampling (*P* = 0.002, see Methods). Given that the majority of the *PALB2*-associated breast cancers were ER+/HER2−, we restricted the comparison of mutation burden to the 12 ER+/HER2− *PALB2*-associated breast cancers sequenced by WES (median of somatic mutations 125, range 63–269) and the 441 ER+/HER2− non-*BRCA1/2/PALB2*-associated breast cancers (median somatic mutations 42, range 2–6666), and the difference remained significant (*P* *<* 0.0001, Mann–Whitney *U* test; *P* = 0.0002, bootstrapping-corrected). As expected, the 12 *PALB2*-associated breast cancers with bi-allelic *PALB2* inactivation analyzed by WES (ten ER+/HER2− and two ER−/HER2−) harbored a significantly higher number of somatic mutations (median 139.5, range 63–269) than the 568 ER+/HER2− and ER−/HER2− (median somatic mutations 50, range 2–6666; *P* = 0.0001, Mann–Whitney *U* test; *P* = 0.005, bootstrapping-corrected). It should be noted that no significant differences in the number of somatic mutations were found between the four *PALB2*-associated breast cancers analyzed by WES lacking bi-allelic *PALB2* inactivation (one ER−/HER2− and three ER+; median 103, range 59–109) and the 683 ER−/HER2− and ER+ non-*BRCA1/2/PALB2*-associated breast cancers (median 51, range 2–6666; *P* *>* 0.05, Mann–Whitney *U* test and bootstrapping-corrected).

**Fig. 4:**
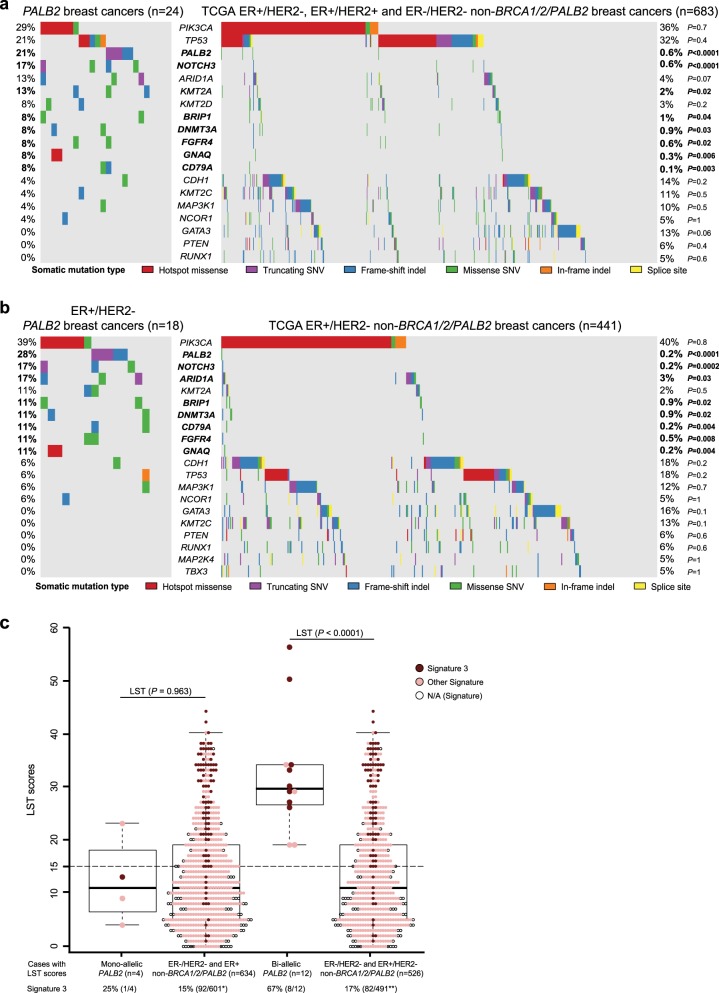
Comparison of *PALB2*-associated breast cancers and non-*BRCA1/2*/*PALB2*-associated breast cancers. **a**, **b** Heatmap depicting the most recurrently mutated genes affecting 410 cancer genes identified in *PALB2*-associated breast cancers and non-*BRCA1/2/PALB2*-associated breast cancers from TCGA.^19^ Cases are shown in columns, genes in rows. Multi-Fisher’s exact test comparisons of mutational frequencies of the mutated genes were performed between **a** the 24 *PALB2*-associated breast cancers and the 683 ER+/HER2−, ER+/HER2+ and ER−/HER2− non-*BRCA1/2/PALB2*-associated breast cancers from TCGA, and **b** the 18 ER+/HER2− *PALB2*-associated breast cancers and the 441 ER+/HER2− non-*BRCA1/2/PALB2*-associated breast cancers from TCGA. *P*-value of each comparison is shown on the right side of the heatmap, with statistically significant *P*-values in bold. Indel, small insertion/deletion; SNV, single nucleotide variant. **c** Box and whisker plots showing the large-scale state transition (LST) scores of the *PALB2*-associated breast cancers with mono-allelic/bi-allelic *PALB2* alterations, ER−/HER2− and ER+ non-*BRCA1/2/PALB2*-associated breast cancers, and ER−/HER2− and ER+/HER2− non-*BRCA1/2/PALB2*-associated breast cancers where LST scores could be inferred. The median value of LST scores, and the 75th and 25th percentiles are displayed at the top and bottom of the boxes, respectively. Each dot represents the LST score and/or mutational signature of a given case. Mutational signatures are color-coded according to the legend. *of the 601 ER−/HER2− and ER+ non-*BRCA1/2/PALB2*-associated breast cancers, the 34 cases lacking LST scores but displaying mutational signatures are not shown, three of these cases display signature 3. **of the 491 ER+/HER2− non-*BRCA1/2/PALB2*-associated breast cancers, the 29 cases lacking LST scores but displaying mutational signatures are not shown, three of these cases display signature 3. *P*-values of the comparisons of LST scores are shown using Fisher’s exact tests. *N/A* signatures not assessable, *LST* large-scale state transition

A comparison of the frequencies of somatic mutations affecting the 410 cancer genes between *PALB2* and non-*BRCA1/2/PALB2*-associated breast cancers revealed that *PALB2*, *NOTCH3*, *KMT2A*, *BRIP1*, *DNMT3A*, *FGFR4*, *GNAQ*, and *CD79A* (all *P* < 0.05; Fisher’s exact test) were more frequently mutated in the 24 *PALB2*-associated breast cancers than in the 683 ER−/HER2− and ER+ non-*BRCA1/2/PALB2*-associated breast cancers (Fig. 4a), however only *PALB2* and *NOTCH3* remained significantly differently mutated between the two groups after bootstrap resampling (*P* < 0.01; Supplementary Table 5). No significant differences in the frequency of *PIK3CA* and *TP53* mutations, the two genes most frequently mutated in breast cancer,^19^ were detected between the 24 *PALB2* and the 683 ER−/HER2− and ER+non-*BRCA1/2/PALB2*-associated breast cancers (Fig. 4a). Upon restriction of the comparison to the 18 ER+ /HER2− *PALB2*-associated breast cancers and the 441 ER+/HER2− non-*BRCA1/2/PALB2*-associated breast cancers, *PALB2*, *NOTCH3*, *ARID1A*, *DNMT3A*, *BRIP1*, *FGFR4*, *CD79A*, and *GNAQ* (all *P* < 0.05; Fisher’s exact test) were significantly more frequently mutated in the *PALB2*-associated breast cancers (Fig. 4b), but similarly only *PALB2* and *NOTCH3* remained significantly different after bootstrapping resampling (*P* < 0.01, Fisher’s exact test and bootstrapping-corrected; Supplementary Table 5).

Differences in the patterns of CNAs were observed between *PALB2*-associated breast cancers and non-*BRCA1/2/PALB2*-associated breast cancers. The 24 *PALB2*-associated breast cancers harbored gains of 16p and losses of 13p and 16q less frequently than the 683 ER−/HER2− and ER+ non-*BRCA1/2/PALB2*-associated breast cancers (*P* *<* 0.05, Fisher’s exact test and bootstrapping-corrected; Supplementary Fig. 4a). When restricting the comparison to the 18 ER+/HER2− *PALB2*-associated breast cancers and the 441 ER+/HER2− non-*BRCA1/2/PALB2*-associated breast cancers, the differences were less overt, with more frequent 16p gains and 16q losses in the non-*BRCA1/2/PALB2*-associated breast cancers (*P* *<* 0.05, Fisher’s exact test and bootstrapping-corrected; Supplementary Fig. 4b). Fewer differences were detected in the comparisons between the eight *PALB2*-associated breast cancers with mono-allelic inactivation (two ER−/HER2− and four ER+) and the 683 ER−/HER2− and ER+non-*BRCA1/2/PALB2*-associated breast cancers (Supplementary Fig. 4c). No significant difference in the frequency of amplifications and homozygous deletions was observed in any of the comparisons when the bootstrap resampling analysis was performed (*P* *>* 0.05; Supplementary Fig. 4d–f).

As part of the exploratory analysis, we investigated whether *PALB2*-associated breast cancers would differ from non-*BRCA1/2/PALB2*-associated breast cancers in regard to the frequencies of genomic features indicative of HRD. The 12 *PALB2*-associated breast cancers with bi-allelic inactivation (two ER−/HER2− and ten ER+/HER2− analyzed by WES) were found to display significantly higher LST scores than the 526 ER−/HER2− and ER+/HER2− non-*BRCA1/2/PALB2*-associated breast cancers for which LST scores could be determined (*P* *<* 0.0001, Mann–Whitney *U* test; *P* *=* 0.0001, bootstrapping-corrected; Fig. 4c). By contrast, the four *PALB2*-associated breast cancers with mono-allelic inactivation (one ER−/HER2− and three ER+, sequenced by WES) displayed comparable LST scores to the 634 ER−/HER2− and ER+ non-*BRCA1/2/PALB2*-associated breast cancers for which LST scores could be defined (*P* *>* 0.05, Mann–Whitney *U* test and bootstrapping-corrected; Fig. 4c). Likewise, the proportion of cases displaying a mutational signature 3 was significantly higher in the 12 *PALB2*-associated breast cancers with bi-allelic inactivation sequenced by WES than in the 491 ER−/HER2− and ER+/HER2− non-*BRCA1/2/PALB2*-associated breast cancers for which mutational signatures could be inferred (67% vs. 17%; *P* = 0.0002, Fisher’s exact test; *P* *=* 0.02, bootstrapping-corrected; Fig. 4c). These results suggest that *PALB2*-associated breast cancers with bi-allelic inactivation are more often HR-deficient than non-*BRCA1/2/PALB2*-associated breast cancers despite displaying a similar prevalence of ER-positive luminal breast cancers, and that *PALB2*-associated breast cancers without bi-allelic inactivation appear to resemble non-*BRCA1/2/PALB2*-associated breast cancers.

### *PALB2*-associated breast cancers with bi-allelic inactivation display similarities with *BRCA1-*associated and *BRCA2-*associated breast cancers with bi-allelic inactivation of *BRCA1*/*2*

**Fig. 5:**
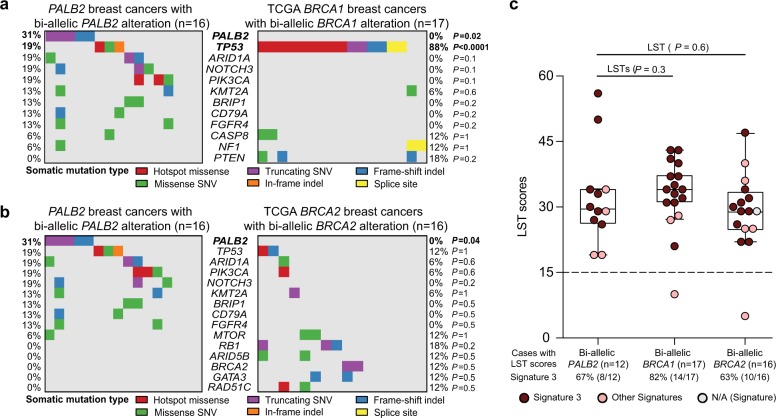
Comparison of *PALB2*-associated breast cancers and *BRCA1* and *BRCA2* breast cancers. **a**, **b** Heatmap depicting the most recurrently mutated genes affecting 410 cancer genes identified in *PALB2*-associated breast cancers and *BRCA1* and *BRCA2* breast cancers from TCGA.^19^ Cases are shown in columns, and genes in rows. Multi-Fisher’s exact test comparisons of mutational frequencies of the recurrently mutated genes were performed between **a** the 16 *PALB2*-associated breast cancers with bi-allelic *PALB2* alterations and 17 *BRCA1* breast cancers bi-allelic *BRCA1* alterations, and **b** the 16 *PALB2*-associated breast cancers with bi-allelic *PALB2* alterations and 16 *BRCA2* breast cancers with bi-allelic *BRCA2* alterations. *P*-value of each comparison is shown on the right side of the heatmap, with statistically significant *P*-values in bold. Indel, small insertion/deletion; SNV, single nucleotide variant. **c** Boxplots showing the large-scale state transition (LST) scores of the 12 *PALB2*-associated breast cancers with bi-allelic *PALB2* alterations, 17 *BRCA1* and 16 *BRCA2* breast cancers with bi-allelic *BRCA1* and *BRCA2* alterations, respectively. The median value of the LST scores, and the 75th and 25th percentiles are displayed at the top and bottom of the boxes, respectively. Each dot corresponds to the LST score and/or mutational signature of one case. Mutational signatures are color-coded according to the legend. *P*-values of the comparisons of LST scores are shown using Fisher’s exact tests. *N/A* signatures not assessable, *LST* large-scale state transition

Finally, we sought to define whether *PALB2*-associated breast cancers with bi-allelic *PALB2* inactivation would differ from breast cancers arising in *BRCA1* and *BRCA2* pathogenic germline mutation carriers with bi-allelic inactivation of *BRCA1* and *BRCA2*, respectively. The 12 *PALB2*-associated breast cancers analyzed by WES were found to harbor a number of somatic mutations (median 139.5, range 63–269) comparable to that of 17 *BRCA1-*associated breast cancers with bi-allelic inactivation from TCGA (median 143, range 54–1223; *P* *>* 0.05, Mann–Whitney *U* test), and higher than that of the 16 *BRCA2-*associated breast cancers with bi-allelic inactivation from TCGA (median 74.5, range 38–209; *P* *=* 0.006, Mann–Whitney *U* test). In regards to the repertoire of somatic mutations, *PALB2* mutations were significantly more frequent in the 16 *PALB2*-associated breast cancers with bi-allelic inactivation (*n* = 5, 31%) than in the 17 *BRCA1*− (*n* = 0) and 16 *BRCA2-*associated (*n* = 0) breast cancers with bi-allelic inactivation from TCGA (*P* *=* 0.02 and *P* *=* 0.04, respectively, Mann–Whitney *U* test; Fig. 5a, b, Supplementary Table 5). In addition, a higher frequency of *TP53* mutations was found in the 17 *BRCA1-*associated breast cancers (*n* = 15, 88%) than in the 16 *PALB2*-associated breast cancers with bi-allelic inactivation (*n* = 3, 19%; *P* *<* 0.0001, Fisher’s exact test; Fig. 5a).

CN analysis revealed that the 17 *BRCA1-*associated breast cancers with bi-allelic inactivation had higher frequencies of gains of 3q and 6p and losses of 17q, among other differences (*P* *<* 0.05, Fisher’s exact test; Supplementary Fig. 5a), as compared to the 16 *PALB2*-associated breast cancers with bi-allelic inactivation. In contrast, the CN profiles of the 16 *BRCA2* breast cancers with bi-allelic inactivation were more similar to those of the *PALB2*-associated breast cancers, albeit more frequently harboring losses of 13q and 22q, among other differences (*P* *<* 0.05; Supplementary Fig. 5b). No significant difference in the frequency of amplifications and homozygous deletions was found between the 16 *PALB2*-associated breast cancers and the 17 *BRCA1-*associated and 16 *BRCA2-*associated breast cancers with bi-allelic inactivation of the respective wild-type allele (Supplementary Fig. 5c, d).

The LST scores of the 12 breast cancers with bi-allelic *PALB2* inactivation analyzed by WES were comparable to those of the breast cancers with bi-allelic *BRCA1* inactivation (*n* = 17) and bi-allelic *BRCA2* inactivation from TCGA (*n* = 16; *P* > 0.05, Mann–Whitney *U* test; Fig. 5c), whereas the proportion of the *PALB2*-associated breast cancers with bi-allelic inactivation displaying signature 3 (67%, 8/12) was not statistically significantly different from that of the *BRCA1-*associated breast cancers with bi-allelic inactivation (82%, 14/17; *P* *=* 0.4, Fisher’s exact test) and *BRCA2-*associated breast cancers with bi-allelic inactivation from TCGA (63%, 10/16; *P* *=* 1, Fisher’s exact test; Fig. 5c). Consistent with these findings, LST score, NtAI score,^26^ which assesses telomeric allelic imbalance, and the Myriad score,^26^ which is the unweighted sum of LOH, telomeric allelic imbalance and LSTs, were higher in breast cancers with *PALB2* (*n* = 12) and *BRCA1/2* (*n* = 33) biallelic inactivation, compared to those with *PALB2* (*n* = 4) and *BRCA1/2* (*n* = 8) monoallelic inactivation, respectively (Supplementary Figs. 6a–6c). Moreover, LST score, mutational signature 3, NtAI score and Myriad score detected bi-allelic inactivation of *PALB2* and of *BRCA1*/*BRCA2* in *PALB2*-associated breast cancers and in *BRCA1/2*-associated breast cancers, respectively, with comparable accuracy (Supplementary Figs. 6d–6g). Taken together, our results suggest that *PALB2*-associated breast cancers with bi-allelic inactivation are similar to breast cancers with *BRCA1* and *BRCA2*− bi-allelic inactivation in terms of genetic instability and genomic features indicative of HRD.

## Discussion

Here we demonstrate that *PALB2*-associated breast cancers constitute a heterogeneous group of tumors at the genetic level and can be stratified according to the bi-allelic inactivation of the *PALB2* wild-type allele. *PALB2*-associated breast cancers display a high mutation burden and a limited number of genes recurrently affected by pathogenic somatic mutations, including *PIK3CA, TP53*, *NOTCH3*, and *PALB2* itself. Loss of the *PALB2* wild-type allele in *PALB2-*associated breast cancers occurred in the form of *PALB2* pathogenic somatic mutations in five (21%) cases, whereas LOH of the wild-type allele of *PALB2* was detected in 11 (46%) cases. Second somatic mutations in *BRCA1*/*2* have been reported as the underlying cause of bi-allelic inactivation in tumors from *BRCA1*/*2* germline mutations carriers.^27^ It should be noted, however, that somatic mutations resulting in the inactivation of the wild-type allele of *BRCA1* or *BRCA2* in *BRCA1*− or *BRCA2-*associated breast cancers, respectively,^15,24,28^ appear to be less frequent than somatic *PALB2* mutations in the context of *PALB2*-associated breast cancers. In the study by Maxwell et al.^28^ bi-allelic *BRCA1* inactivation was due to a *BRCA1* somatic mutation in only one case (1.1%) out of 93 *BRCA1*-associated breast and ovarian tumors. Similarly, out of 67 *BRCA2*-associated tumors with bi-allelic *BRCA2* inactivation, in only one case this was due to a *BRCA2* somatic mutation (1.5%). In contrast, *PALB2* somatic mutations as a mechanism of bi-allelic inactivation were significantly more frequent in the *PALB2*-associated breast cancers from this series (31%; 5/16; *P* = 0.00006, Fisher’s exact test).

Consistent with the findings of Lee et al.,^12^ our study demonstrates that *PALB2* follows the Knudson two-hit model, given that in a large proportion of *PALB2*-associated breast cancers, a second hit in the form of a somatic *PALB2* mutation or LOH of the wild-type allele of *PALB2* was detected. Contrary to that study,^12^ in which *PALB2*-associated breast cancers with either mono-allelic or bi-allelic *PALB2* alterations were found to display genomic features of HRD, based on targeted massively parallel sequencing of 487 genes, our WES analysis of 16 *PALB2*-associated breast cancers revealed that tumors with *PALB2* bi-allelic alterations displayed significantly higher LST scores and average deletion lengths than *PALB2-*associated breast cancers with mono-allelic *PALB2* alterations. In addition, only one out of the four *PALB2-*associated breast cancers with mono-allelic *PALB2* alterations displayed a dominant mutational signature 3, whereas eight out of 12 *PALB2*-associated breast cancers with bi-allelic *PALB2* inactivation harbored a dominant mutational signature 3. Our WES findings are consistent with the pan-cancer WES analysis performed by Riaz et al.,^15^ whereby HR-related genes with bi-allelic inactivation but not those with mono-allelic alterations were found to display genomic features of HRD, and the analyses performed by Polak et al.,^24^ where bi-allelic, but not mono-allelic, alterations affecting *BRCA1, BRCA2*, and *PALB2* were found to be associated with HRD in breast cancers. Conversely, 8/24 *PALB2*-associated breast cancers included in this study lacked bi-allelic *PALB2* inactivation and 2/16 *PALB2*-associated breast cancers sequenced by WES lacked both bi-allelic *PALB2* inactivation and genomic features of HRD. In this context, one could posit that this subset of *PALB2*-associated breast cancers may retain competent HR repair of DNA double-strand breaks and would unlikely benefit from HRD-directed therapies. Interestingly, the proportion of *PALB2*-associated breast cancers displaying mono-allelic *PALB2* inactivation was comparable to the one of *BRCA1*-associated and *BRCA2*-associated breast cancers from TCGA harboring *BRCA1* or *BRCA2* mono-allelic inactivation, respectively. Although *PALB2* mono-allelic inactivation is not associated with genomic features of HRD, its role in tumorigenesis is yet to be determined.

In agreement with previous studies showing that most breast cancers with HRD features are underpinned by bi-allelic inactivation of HR-related genes,^15,29^ we identified the genetic basis of HRD in 12 out of 14 (86%) *PALB2*-associated breast cancers with genomic features of HRD. It should be noted that of the *PALB2*-associated breast cancers with mono-allelic *PALB2* alterations studied here, one displayed a high LST score and another one harbored a dominant mutational signature 3. This observation suggests that other mechanisms of HRD may be operative in these tumors. First, no bi-allelic inactivation of another DNA repair related gene was detected in these cancers. Second, albeit *PALB2* gene promoter methylation was reported in two of eight inherited breast cancers and four of 60 sporadic breast cancers,^13^ this phenomenon appears to be vanishingly rare in *PALB2*-associated breast cancers with mono-allelic *PALB2* alterations.^12^ Further analyses are warranted to define whether other mechanisms of inactivation of the wild-type allele of *PALB2* may play a role in *PALB2-*associated breast cancers with mono-allelic *PALB2* alterations but with genomic features of HRD.

Our study has important limitations. First, as a result of the rarity of *PALB2*-associated breast cancers, the small sample size may have limited the detection of significant differences in the exploratory analyses comparing *PALB2*-associated breast cancers with non-*BRCA1/2/PALB2*-associated breast cancers from TCGA. Importantly, however, these analyses revealed that *PALB2*-associated breast cancers with bi-allelic inactivation differ from non-*BRCA1/2/PALB2*-associated breast cancers but are similar to *BRCA1* and *BRCA2* breast cancers with bi-allelic inactivation. Second, genomic features of HRD were investigated here based on WES; although our approach for the detection of genomic features of HRD is more robust than that based on targeted massively parallel sequencing,^12^ whole-genome sequencing analysis still remains the optimal approach. Therefore, the proportion of *PALB2*-associated breast cancers with HRD may be even higher than that reported here and in previous analyses.^12^

Despite these limitations, our data demonstrate that *PALB2*-associated breast cancers harbor complex and heterogeneous genomes. Notably, *PALB2* bi-allelic inactivation is present in a large proportion of *PALB2*-associated breast cancers, and the mechanisms leading to this include both LOH of the wild-type allele or pathogenic somatic mutations affecting *PALB2*. Importantly, the 12 *PALB2*-associated breast cancers with *PALB2* bi-allelic inactivation displayed genomic features consistent with HRD, and shared similarities in terms of genetic instability and genomic features of HRD with *BRCA1-*associated and *BRCA2-*associated breast cancers with bi-allelic inactivation. Two of the 16 *PALB2*-associated breast cancers subjected to WES, however, lacked both bi-allelic inactivation of *PALB2* and genomic features of HRD. Hence, we posit that molecular assays to identify bi-allelic inactivation of *PALB2* and/or genomic features of HRD may aid in the selection of patients likely to benefit from HRD-directed therapies, including platinum-based chemotherapy and/or PARP inhibitors.

## Methods

### Cases

We included 24 invasive breast cancers from women with pathogenic *PALB2* germline mutations. This study was approved by Memorial Sloan Kettering Cancer Center’s institutional review board (IRB) and by the local ethics committees/IRBs of the authors’ institutions. Written informed consents were obtained as required by the protocols approved by the IRBs/local ethics committees of the respective authors’ institutions. This study is in compliance with the Declaration of Helsinki. For 22 breast cancers, tissue samples were retrieved from the McGill University (Canada, *n* = 6), Cancer Research Malaysia/University Malaya (Malaysia, *n* = 5), the Kathleen Cuningham Foundation Consortium for research into Familial Breast Cancer (kConFab, Australia, *n* = 5), Memorial Sloan Kettering Cancer Center (MSKCC; New York, USA, *n* = 3), University of Eastern Finland (Finland, *n* = 2) and Charles University (Czech Republic, *n* = 1). Hematoxylin and eosin-stained tissue sections of the 22 breast cancers were reviewed by three pathologists (FCG, FP, and JSR-F). The genomics data of IDC53 were in part previously reported in Foo et al.^9^ In addition, the WES-derived mutational and clinico-pathologic data of two *PALB2*-associated breast cancers (IDC60 and IDC61; Table 1) were retrieved from the provisional TCGA breast cancer dataset at the Broad’s Institute firehose on 01/28/16.

### Immunohistochemistry and fluorescence in situ hybridization (FISH)

ER and HER2 status were assessed by immunohistochemistry following American Society of Clinical Oncology (ASCO)/College of American Pathologists (CAP) guidelines.^30^ In addition, *HER2* amplification was assessed in selected cases by fluorescence in situ hybridization (FISH) using PathVysion (Abbott) and/or *HER2* IQFISH pharmDx (Dako), following the ASCO/CAP guidelines.^31,32^

### DNA extraction

Eight-micrometer-thick sections from representative FFPE blocks were microdissected with a sterile needle under a stereomicroscope (Olympus) to ensure >80% of tumor cells. Genomic DNA was extracted from tumor and matched normal blood or saliva samples using the DNeasy Blood and Tissue Kit (Qiagen), and quantified using the Qubit Fluorometer (Life Technologies).

### Massively parallel sequencing and bioinformatics analysis

DNA of tumor and matched normal samples was subjected to WES (*n* = 14) or MSK-IMPACT^20^ (*n* = 8), which targets all exons and selected introns of 410 cancer genes. Sequencing data analysis was performed as described previously.^33,34^ Read alignment and mutation calling was performed as described in the Supplementary Methods.^35^ CNAs and regions of LOH were defined using FACETS.^36^ In brief, homozygous deletions correspond to regions with an absolute copy number of zero, hemizygous losses are regions with absolute copy number lower than the average ploidy rounded to the nearest integer value. Low copy number gains are regions with absolute copy number greater than the average ploidy rounded to the nearest integer value, and high-level copy number amplifications are regions with absolute copy number in excess of four relative to the average ploidy rounded to the nearest integer value. Regions of LOH and homozygous deletions were manually reviewed using plots of Log_2_ ratios and B allele frequencies. The cancer cell fraction (CCF) of each mutation was inferred using ABSOLUTE (v1.0.6)^37^ and manually reviewed^33,37,38^ (Supplementary Methods).

### Large-scale state transitions, indel length and mutational signatures

The presence of LSTs, representative of genomic scars indicative of HRD,^39^ was assessed in breast cancers subjected to WES. A LST score cut-off of 15 was adopted to classify breast cancers as LST-high (≥15) or LST-low (<15), as previously described.^9,22,39^ The length of indels was assessed in *PALB2*-associated breast cancers analyzed by WES, given that deletion sizes of ≥5 bp have been associated with defective HR-based repair.^25^ Mutational signatures^40^ were inferred from non-synonymous and silent somatic exonic SNVs (i) using deconstructSigs^41^ based on the set of 30 mutational signatures represented in COSMIC^27^ or (ii) on the 12 mutational signatures known to occur in breast cancers^27^ and (iii) using a non-negative matrix factorization algorithm (NMF)^42^ based on the 30 signatures from COSMIC,^27^ in samples with at least 30 somatic mutations, as previously described.^15,22^ The dominant mutational signature in each case was defined based on the consensus of at least two of the three methods.

### Sanger sequencing validation

Selected somatic mutations with MAFs > 10%, including mutations affecting *PIK3CA* (*n* = 7), *PALB2* (*n* = 4), *TP53* (*n* = 4), and *NOTCH3* (*n* = 2), were validated by Sanger sequencing (primer sequences in Supplementary Table 3). PCR amplification of genomic DNA and analyses were performed in duplicate.

### Comparisons with breast cancers from TCGA

The mutation burden, mutation frequencies, CNAs and genomic features indicative of HRD of the *PALB2*-associated breast cancers were compared to those of non*-BRCA1/2/PALB2*-associated breast cancers with matched ER and HER2 status (*n* = 683), and to those of *BRCA1* (*n* = 17) and *BRCA2* (*n* = 16) breast cancers with bi-allelic inactivation from TCGA^43^ **(**Supplementary Methods**)**.

### Statistical analysis

Comparisons of the number of somatic mutations and LST scores, gene-level copy number states and mutational signatures between *PALB2*-associated breast cancers and non-*BRCA1/2/PALB2-*associated, *BRCA1-*associated and *BRCA2-*associated breast cancers were performed using the Mann–Whitney *U* test and Fisher’s exact test, respectively. To account for differences in sample sizes, a bootstrap resampling analysis was performed (Supplementary Methods).

### Reporting summary

Further information on experimental design is available in the Nature Research Reporting Summary linked to this paper.

## Supplementary information

Supplementary material.

Reporting Summary Checklist

## Data Availability

WES sequencing data (supporting Fig. 1–5, Table 1, Supplementary Figs. 2–6 and supplementary tables 1–5) and MSK-IMPACT sequencing data (supporting Figs. 1, 2, 4 and 5, Table 1, supplementary Figs. 2, 4 and 5 and supplementary tables 1–5) generated during this study, can be accessed from cBioPortal (https://identifiers.org/cbioportal:brca_msk_li_2019). TCGA Breast Cancer sequencing data (supporting Figs. 4 and 5, supplementary Figs. 4–6 and supplementary table 5) used in this study, can be accessed from cBioPortal (https://identifiers.org/cbioportal:brca_tcga_pan_can_atlas_2018) or from the related publication 10.1016/j.cell.2018.02.060. Additional data supporting supplementary table 4 can be accessed from table 2 and supplementary table 1 of the related publication: 10.1002/path.5055. The data generated and analyzed during this study are described in the following data record: 10.6084/m9.figshare.8138912.

## Supplementary information

**Supplementary information** accompanies the paper on the *npj Breast Cancer* website (10.1038/s41523-019-0115-9).

## References

[1] Xia B (2006). Control of BRCA2 cellular and clinical functions by a nuclear partner, PALB2. Mol. Cell..

[2] Reid S (2007). Biallelic mutations in PALB2 cause Fanconi anemia subtype FA-N and predispose to childhood cancer. Nat. Genet..

[3] Kanchi KL (2014). Integrated analysis of germline and somatic variants in ovarian cancer. Nat. Commun..

[4] Antoniou AC (2014). Breast-cancer risk in families with mutations in PALB2. N. Engl. J. Med..

[5] Takeuchi S, Doi M, Ikari N, Yamamoto M, Furukawa T (2018). Mutations in BRCA1, BRCA2, and PALB2, and a panel of 50 cancer-associated genes in pancreatic ductal adenocarcinoma. Sci. Rep..

[6] Chen S, Parmigiani G (2007). Meta-analysis of BRCA1 and BRCA2 penetrance. J. Clin. Oncol..

[7] Cybulski C (2015). Clinical outcomes in women with breast cancer and a PALB2 mutation: a prospective cohort analysis. Lancet Oncol..

[8] Nikkila J (2013). Heterozygous mutations in PALB2 cause DNA replication and damage response defects. Nat. Commun..

[9] Foo TK (2017). Compromised BRCA1-PALB2 interaction is associated with breast cancer risk. Oncogene.

[10] Isaac, D., Karapetyan, L., Tamkus, D. Association of germline PALB2 mutation and response to platinum-based chemotherapy in metastatic breast cancer: a case series. *JCO Precision Oncol.* **2**, 1–5 (2018). 10.1200/PO.17.00258 35135128

[11] Knudson AG (2001). Two genetic hits (more or less) to cancer. Nat. Rev. Cancer.

[12] Lee JEA (2018). Molecular analysis of PALB2-associated breast cancers. J. Pathol..

[13] Potapova A, Hoffman AM, Godwin AK, Al-Saleem T, Cairns P (2008). Promoter hypermethylation of the PALB2 susceptibility gene in inherited and sporadic breast and ovarian cancer. Cancer Res..

[14] Poumpouridou N (2016). Development and validation of molecular methodologies to assess PALB2 expression in sporadic breast cancer. Clin. Biochem..

[15] Riaz N (2017). Pan-cancer analysis of bi-allelic alterations in homologous recombination DNA repair genes. Nat. Commun..

[16] Foulkes WD (2007). Identification of a novel truncating PALB2 mutation and analysis of its contribution to early-onset breast cancer in French-Canadian women. Breast Cancer Res..

[17] Erkko H (2007). A recurrent mutation in PALB2 in Finnish cancer families. Nature.

[18] Ramus S. J. et al. Germline mutations in the BRIP1, BARD1, PALB2, and NBN genes in women with ovarian cancer. *J. Natl Cancer Inst*. **107**, pii: djv214 (2015). 10.1093/jnci/djv214 PMC4643629 26315354

[19] Cancer Genome Atlas Network. (2012). Comprehensive molecular portraits of human breast tumours. Nature.

[20] Cheng DT (2015). Memorial Sloan Kettering-integrated mutation profiling of actionable cancer targets (MSK-IMPACT): a hybridization capture-based next-generation sequencing clinical assay for solid tumor molecular oncology. J. Mol. Diagn..

[21] Honrado E, Osorio A, Palacios J, Benitez J (2006). Pathology and gene expression of hereditary breast tumors associated with BRCA1, BRCA2 and CHEK2 gene mutations. Oncogene.

[22] Weigelt B (2018). The landscape of somatic genetic alterations in breast cancers from ATM germline mutation carriers. J. Natl. Cancer Inst..

[23] Turner NC, Reis-Filho JS (2013). Tackling the diversity of triple-negative breast cancer. Clin. Cancer Res..

[24] Polak Paz, Kim Jaegil, Braunstein Lior Z, Karlic Rosa, Haradhavala Nicholas J, Tiao Grace, Rosebrock Daniel, Livitz Dimitri, Kübler Kirsten, Mouw Kent W, Kamburov Atanas, Maruvka Yosef E, Leshchiner Ignaty, Lander Eric S, Golub Todd R, Zick Aviad, Orthwein Alexandre, Lawrence Michael S, Batra Rajbir N, Caldas Carlos, Haber Daniel A, Laird Peter W, Shen Hui, Ellisen Leif W, D'Andrea Alan D, Chanock Stephen J, Foulkes William D, Getz Gad (2017). A mutational signature reveals alterations underlying deficient homologous recombination repair in breast cancer. Nature Genetics.

[25] Alexandrov, L. et al. The repertoire of mutational signatures in human cancer. *bioRxiv*. 10.1101/322859 (2018).

[26] Telli ML (2016). Homologous recombination deficiency (HRD) score predicts response to platinum-containing neoadjuvant chemotherapy in patients with triple-negative breast cancer. Clin. Cancer Res..

[27] Nik-Zainal S (2016). Landscape of somatic mutations in 560 breast cancer whole-genome sequences. Nature.

[28] Maxwell KN (2017). BRCA locus-specific loss of heterozygosity in germline BRCA1 and BRCA2 carriers. Nat. Commun..

[29] Mutter RW (2017). Bi-allelic alterations in DNA repair genes underpin homologous recombination DNA repair defects in breast cancer. J. Pathol..

[30] Hammond ME (2010). American Society of Clinical Oncology/College Of American Pathologists guideline recommendations for immunohistochemical testing of estrogen and progesterone receptors in breast cancer. J. Clin. Oncol..

[31] Wolff AC (2013). Recommendations for human epidermal growth factor receptor 2 testing in breast cancer: American Society of Clinical Oncology/College of American Pathologists clinical practice guideline update. J. Clin. Oncol..

[32] Wolff AC (2014). Recommendations for human epidermal growth factor receptor 2 testing in breast cancer: American Society of Clinical Oncology/College of American Pathologists clinical practice guideline update. Arch. Pathol. Lab Med..

[33] Ng CKY (2017). The landscape of somatic genetic alterations in metaplastic breast carcinomas. Clin. Cancer Res..

[34] Geyer FC (2018). Recurrent hotspot mutations in HRAS Q61 and PI3K-AKT pathway genes as drivers of breast adenomyoepitheliomas. Nat. Commun..

[35] Li, A. et al. Metadata supporting data files of the related manuscript: homologous recombination DNA repair defects in PALB2-associated breast cancers. figshare 10.6084/m9.figshare.8138912 (2019).

[36] Shen R, Seshan VE (2016). FACETS: allele-specific copy number and clonal heterogeneity analysis tool for high-throughput DNA sequencing. Nucleic Acids Res.

[37] Carter SL (2012). Absolute quantification of somatic DNA alterations in human cancer. Nat. Biotechnol..

[38] Landau DA (2013). Evolution and impact of subclonal mutations in chronic lymphocytic leukemia. Cell.

[39] Popova T (2012). Ploidy and large-scale genomic instability consistently identify basal-like breast carcinomas with BRCA1/2 inactivation. Cancer Res..

[40] Alexandrov LB (2013). Signatures of mutational processes in human cancer. Nature.

[41] Rosenthal R, McGranahan N, Herrero J, Taylor BS, Swanton C (2016). DeconstructSigs: delineating mutational processes in single tumors distinguishes DNA repair deficiencies and patterns of carcinoma evolution. Genome Biol..

[42] Gaujoux R, Seoighe C (2010). A flexible R package for nonnegative matrix factorization. BMC Bioinforma..

[43] Bailey MH (2018). Comprehensive characterization of cancer driver genes and mutations. Cell.

