# Publisher Correction: Homologous recombination DNA repair defects in *PALB2*-associated breast cancers

**DOI:** 10.1038/s41523-019-0140-8

**Published:** 2019-11-19

**Authors:** Anqi Li, Felipe C. Geyer, Pedro Blecua, Ju Youn Lee, Pier Selenica, David N. Brown, Fresia Pareja, Simon S. K. Lee, Rahul Kumar, Barbara Rivera, Rui Bi, Salvatore Piscuoglio, Hannah Y. Wen, John R. Lozada, Rodrigo Gularte-Mérida, Luca Cavallone, kConFab Investigators, Morteza Aghmesheh, David Amor, Leslie Andrews, Yoland Antill, Rosemary Balleine, Jonathan Beesley, Anneke Blackburn, Michael Bogwitz, Melissa Brown, Matthew Burgess, Jo Burke, Phyllis Butow, Liz Caldon, Ian Campbell, Alice Christian, Christine Clarke, Paul Cohen, Ashley Crook, James Cui, Margaret Cummings, Sarah-Jane Dawson, Anna De Fazio, Martin Delatycki, Alex Dobrovic, Tracy Dudding, Pascal Duijf, Edward Edkins, Stacey Edwards, Gelareh Farshid, Andrew Fellows, Michael Field, James Flanagan, Peter Fong, John Forbes, Laura Forrest, Stephen Fox, Juliet French, Michael Friedlander, David Gallego Ortega, Michael Gattas, Graham Giles, Grantley Gill, Margaret Gleeson, Sian Greening, Eric Haan, Marion Harris, Nick Hayward, Ian Hickie, John Hopper, Clare Hunt, Paul James, Mark Jenkins, Rick Kefford, Maira Kentwell, Judy Kirk, James Kollias, Sunil Lakhani, Geoff Lindeman, Lara Lipton, Lizz Lobb, Sheau Lok, Finlay Macrea, Graham Mann, Deb Marsh, Sue-Anne McLachlan, Bettina Meiser, Roger Milne, Sophie Nightingale, Shona O’Connell, Nick Pachter, Briony Patterson, Kelly Phillips, Mona Saleh, Elizabeth Salisbury, Christobel Saunders, Jodi Saunus, Clare Scott, Rodney Scott, Adrienne Sexton, Andrew Shelling, Peter Simpson, Allan Spigelman, Mandy Spurdle, Jennifer Stone, Jessica Taylor, Heather Thorne, Alison Trainer, Georgia Trench, Kathy Tucker, Jane Visvader, Logan Walker, Mathew Wallis, Rachael Williams, Ingrid Winship, Kathy Wu, Mary Anne Young, Zoulikha Rezoug, Tu Nguyen-Dumont, Paolo Peterlongo, Carlo Tondini, Thorkild Terkelsen, Karina Rønlund, Susanne E. Boonen, Arto Mannerma, Robert Winqvist, Marketa Janatova, Pathmanathan Rajadurai, Bing Xia, Larry Norton, Mark E. Robson, Pei-Sze Ng, Lai-Meng Looi, Melissa C. Southey, Britta Weigelt, Teo Soo-Hwang, Marc Tischkowitz, William D. Foulkes, Jorge S. Reis-Filho

**Affiliations:** 1 Department of Pathology, Memorial Sloan Kettering Cancer Center, New York, NY USA; 2 Department of Pathology, Fudan University Shanghai Cancer Center and Shanghai Medical College, Fudan University, Shanghai, P.R. China; 3 Radiation Oncology, Memorial Sloan Kettering Cancer Center, New York, NY USA; 4 Departments of Oncology and Human Genetics, McGill University, Montreal, Quebec Canada; 5 Cancer Axis, Lady Davis Institute, Jewish General Hospital, Montreal, Quebec Canada; 6 Institute of Pathology, University Hospital Basel, Basel, Switzerland; 7 Cancer Prevention Center, Jewish General Hospital, Montreal, Quebec Canada; 8 Genetic Epidemiology Laboratory, Department of Clinical Pathology, University of Melbourne, Parkville, Victoria, Australia; 9 Precision Medicine, School of Clinical Sciences at Monash Health, Monash University, Victoria, Australia; 10 IFOM, The Italian Foundation for Cancer Research Institute of Molecular Oncology, Milan, Italy; 11 Ospedale Papa Giovanni XXIII, Bergamo, Italy; 12 Department of Clinical Genetics, Aarhus University Hospital, Aarhus, Denmark; 13 Department of Clinical Genetics, Vejle Hospital, Vejle, Denmark; 14 Clinical Genetics Unit, Department of Pediatrics, Zealand University Hospital, Roskilde, Denmark; 15 Biocenter Kuopio and Cancer Center of Easter Finland, University of Eastern Finland, Kuopio, Finland; 16 Laboratory of Cancer Genetics and Tumor Biology, Cancer and Translational Medicine Research Unit, Biocenter Oulu, University of Oulu, Oulu, Finland; 17 Institute of Biochemistry and Experimental Oncology, First Faculty of Medicine, Charles University, Prague, Czech Republic; 18 Department of Pathology, Subang Jaya Medical Centre, Subang Jaya, Selangor Malaysia; 19 Department of Radiation Oncology, Rutgers Cancer Institute of New Jersey, New Brunswick, NJ USA; 20 Department of Medicine, Memorial Sloan Kettering Cancer Center, New York, NY USA; 21 Cancer Research Malaysia, Subang Jaya, Malaysia; 22 Department of Pathology, Faculty of Medicine, University Malaya, Kuala Lumpur, Malaysia; 23 University Malaya Cancer Research Institute, Faculty of Medicine, University Malaya, Kuala Lumpur, Malaysia; 24 Department of Medical Genetics, University of Cambridge, Cambridge, UK; 25 Cancer Program, Research Institute McGill University Health Centre, Montreal, Quebec Canada; 26 Illawarra Cancer Care Centre, Wollongong Hospital, Wollongong, NSW 2500 Australia; 27 Genetic Health Services Victoria, Royal Children’s Hospital, Melbourne, VIC, 3050 Australia; 28 Prince of Wales Hospital, Randwick, NSW 2031 Australia; 29 The Family Cancer Clinic, Cabrini Hospital, Malvern, VIC 3144 Australia; 30 Department of Medical Oncology, Westmead Hospital, Westmead, NSW 2145 Australia; 31 Queensland Institute of Medical Research, Herston Qld, 4002 Australia; 32 Australian National University, Canberra ACT, Canberra, 2601 Australia; 33 Familial Cancer Centre, The Royal Melbourne Hospital, Parkville, VIC 3050 Australia; 34 University of Queensland, St. Lucia, QLD 4072 Australia; 35 Clinical Genetics Service, Austin Health, Vic, 3084 Australia; 36 Royal Hobart Hospital, Hobart, Tasmania 7001 Australia; 37 Medical Psychology Unit, Royal Prince Alfred Hospital, Camperdown NSW, 2204 Australia; 38 Garvan Institute of Medical Research, Darlinghurst, NSW 2010 Australia; 39 Cancer Genetics Laboratory, Peter MacCallum Cancer Centre, Melbourne, VIC 3000 Australia; 40 Genetics Department, Central Region Genetics Service, Wellington Hospital, Wellington, 6021 New Zealand; 41 Westmead Institute for Cancer Research, University of Sydney, Westmead Hospital, Westmead, NSW 2145 Australia; 42 Gynaecological Cancer Research, St John of God Subiaco Hospital, Subiaco, WA 6008 Australia; 43 Department of Clinical Genetics, Royal North Shore Hospital, St Leonards, NSW 2065 Australia; 44 Epidemiology and Preventive Medicine, Monash University, Prahan, Vic 3004 Australia; 45 Department of Pathology, University of Queensland Medical School, Herston, Qld 4006 Australia; 46 Sir Peter MacCallum Department of Oncology, Melbourne, VIC 3000 Australia; 47 Department of Gynaecological Oncology, Westmead Institute for Cancer Research, Westmead Hospital, Westmead, NSW 2145 Australia; 48 Hunter Genetics, Hunter Area Health Service, Waratah, 2298 NSW Australia; 49 The University of Queensland Diamantina Institute, Brisbane, QLD 4102 Australia; 50 Clinical Chemistry, Princess Margaret Hospital for Children, Perth, WA 6001 Australia; 51 SA Tissue Pathology, IMVS, Adelaide, SA 5000 Australia; 52 Epigenetics Unit, Department of Surgery and Oncology, Imperial College London, London, W12 0NN UK; 53 Regional Cancer and Blood Services, Auckland City Hospital, Auckland, 1023 New Zealand; 54 Surgical Oncology, University of Newcastle, Newcastle Mater Hospital, Waratah, NSW 2298 Australia; 55 Psychosocial Cancer Genetics Research Group, Parkville Familial Cancer Centre, Melbourne, Vic 3000 Australia; 56 School of Molecular and Microbial Sciences, University of Queensland, St Lucia, Qld 4072 Australia; 57 Department of Medical Oncology, Prince of Wales Hospital, Randwick, NSW 2031 Australia; 58 Tumour Development Group, Garvan Institute of Medical Research, The Kinghorn Cancer Centre, Darlinghurst, NSDW 2010 Australia; 59 Queensland Clinical Genetic Service, Royal Children’s Hospital, Herston, QLD 4020 Australia; 60 Anti-Cancer Council of Victoria, Melbourne, VIC 3004 Australia; 61 Department of Surgery, Royal Adelaide Hospital, Adelaide, 5000 Australia; 62 Hunter Family Cancer Service, Waratah, NSW 2298 Australia; 63 Department of Medical Genetics, Women’s and Children’s Hospital, North Adelaide, SA 5006 Australia; 64 Family Cancer Clinic, Monash Medical Centre, Clayton, 3168 Australia; 65 Brain and Mind Centre, Camperdown, NSW 2050 Australia; 66 Centre for M.E.G.A. Epidemiology, University of Melbourne, Carlton, VIC 3010 Australia; 67 Familial Cancer Service, Department of Medicine, Westmead Hospital, Westmead, NSW 2145 Australia; 68 Breast Endocrine and Surgical Unit, Royal Adelaide Hospital, North Terrace, SA 5000 Australia; 69 University of Queensland, The Royal Brisbane & Women’s Hospital, Herston, QLD 4029 Australia; 70 Walter and Eliza Hall Institute, PO Royal Melbourne, Hospital, Parkville, VIC 3050 Australia; 71 Medical Oncology and Clinical Haematology Unit, Western Hospital, Footscray, VIC 3011 Australia; 72 School of Medicine, University of Notre Dame, Kogarah, NSW 2217 Australia; 73 Department of Medical Oncology, The Royal Melbourne Hospital, Parkville, Vic 3050 Australia; 74 Kolling Institute of Medical Research, Royal North Shore Hospital, St Leonards, NSW 2065 Australia; 75 Department of Oncology, St Vincent’s Hospital, Fitzroy, VIC 3065 Australia; 76 Department of Surgery, Peter MacCallum Cancer Centre, Melbourne, VIC 3000 Australia; 77 Genetic Services of WA, King Edward Memorial Hospital, Subiaco, WA 6008 Australia; 78 Centre for Genetic Education, Prince of Wales Hospital, Randwick, NSW 2031 Australia; 79 Anatomical Pathology, Prince of Wales Hospital, Randwick, 2031 NSW Australia; 80 School of Surgery and Pathology, QE11 Medical Centre, Nedlands, WA 6907 Australia; 81 Breast Pathology, University of Queensland Centre for Clinical Research, Royal Brisbane and Women’s Hospital, Herston, Qld 4029 Australia; 82 Hunter Area Pathology Service, John Hunter Hospital, NSW, 2310 Australia; 83 Obstetrics and Gynaecology, University of Auckland, Auckland, 1023 New Zealand; 84 Family Cancer Clinic, St Vincent’s Hospital, Darlinghurst, NSW 2010 Australia; 85 Centre for Genetic Origins of Health and Disease, University of Western Australia, Crawley, WA 6009 Australia; 86 Department of Pathology, University of Otago, Christchurch, 8011 New Zealand; 87 Department of Medicine, Royal Melbourne Hospital, Parkville, VIC 3050 Australia

**Keywords:** Breast cancer, Cancer genetics, Cancer genomics

**Correction to:** *npj Breast Cancer* 10.1038/s41523-019-0115-9, published online 08 August 2019

In the original version of this paper, the link to the data record in the Data Availability Statement was incorrectly listed as 10.6084/m9.figshare.8138912.44. The link has been corrected to 10.6084/m9.figshare.8138912. This has been corrected in the HTML and PDF versions of this article.

