# Supplementary material for: Homologous recombination DNA repair defects in PALB2-associated breast cancers
Source: NPJ Breast Cancer. 2019 Aug 8;5:23. doi: 10.1038/s41523-019-0115-9 (PMC6687719; doi:10.1038/s41523-019-0115-9)
Supplement: Supplementary file 1 — Supplementary material. [file 41523_2019_115_MOESM1_ESM.pdf]

SUPPLEMENTARY MATERIALS

Homologous recombination DNA repair defects in *PALB2*-associated breast cancers

Li et al.

Supplementary Methods
Supplementary Figures 1-6
Supplementary Tables 1-5

SUPPLEMENTARY METHODS

Massively parallel sequencing and bioinformatics analysis

DNA samples derived from microdissected tumor and normal tissue were subjected to WES (n=14) or MSK-IMPACT[1] (n=8), which targets all exons and selected introns of 410 cancer genes. Reads were aligned to the reference human genome GRCh37 using the Burrows-Wheeler Aligner (BWA, v0.7.10).[2] Local realignment, duplicate removal and base quality recalibration were performed using the Genome Analysis Toolkit (GATK, v3.1.1).[3] *PALB2* and *BRCA1/2* germline variants were detected by HaplotypeCaller from GATK in the gvcf mode with default settings, and were retained if the germline variants were present in both normal and tumor samples.[4] Somatic single nucleotide variants (SNVs) were detected by MuTect (v1.0),[5] small insertions and deletions (indels) by Strelka (v2.0.15)[6], VarScan 2 (v2.3.7)[7], Lancet (v1.0.0)[8] and Scalpel (v0.5.3)[9], and further curated by manual inspection. SNVs and indels located outside of the target regions were disregarded.[10] We excluded SNVs and indels for which the tumor mutant allele fraction (MAF) was <5 times that of the paired normal MAF, as well as SNVs and indels found at >5% global minor allele frequency of dbSNP (build 137) as previously described.[10, 11]

The cancer cell fraction (CCF) of each mutation was inferred using ABSOLUTE (v1.0.6)[12] and manually reviewed[11-13] A mutation was classified as clonal if its probability of being clonal was >50%[13] or if the lower bound of the 95% confidence interval of its CCF was >90%;[11, 14]. Somatic LOH of the *PALB2* germline mutations was classified as subclonal if the CCF was lower than the tumor purity (delta ≥0.2) as determined by FACETS,[15] as previously described.[14] We employed a combination of mutation function predictors[16] to infer whether a mutation was potentially pathogenic, as previously described.[11] Mutation hotspots were defined according to Chang et al.[15]

Comparisons with breast cancers from TCGA

Comparisons of mutation burden, mutation frequencies, CNAs and genomic features indicative of HRD were conducted between the *PALB2*-associated breast cancers, non-*BRCA1/2/PALB2*-

associated breast cancers with matched ER and HER2 status (n=683), and *BRCA1*- (n=17) and *BRCA2*-associated (n=16) breast cancers with bi-allelic inactivation from TCGA[17]. The bi-allelic inactivation status of *BRCA1* and *BRCA2* was retrieved from Riaz et al.[4] TCGA breast cancers with available data to assess LST scores and numbers of somatic mutations adequate for mutational signature assessment were employed for comparative analyses of genomic features indicative of HRD. The recently updated publicly available MC3 dataset from the TCGA was obtained at https://gdc.cancer.gov/about-data/publications/mc3-2017 and https://gdac.broadinstitute.org (01/28/2016).

Statistical analysis

Comparisons of the tumor mutation burden, LST scores, gene-level copy number states and mutational signatures between *PALB2*-associated breast cancers and non-*BRCA1/2/PALB2*-, *BRCA1*- and *BRCA2*-associated breast cancers were performed using the Mann-Whitney *U* test and Fisher's exact test, respectively. To account for differences in sample sizes, a bootstrap resampling analysis was performed for the comparisons of mutation burden, mutation frequencies, gene-level copy number states, LSTs and mutational signatures between the *PALB2*-associated and TCGA non-*BRCA1/2/PALB2*-associated breast cancers at a 1:3 ratio. In brief, 3 non-*BRCA1/2/PALB2*-associated breast cancers were randomly selected for each *PALB2*-associated breast cancer, allowing for a given non-*BRCA1/2/PALB2*-associated sample to be included in the distinct random subsets. We iterated this process 100 times and two-tailed *P*-values were calculated using either Mann-Whitney *U* test or Fisher's exact test for each iteration, as appropriate. A bootstrapping-corrected *P*-value was determined as the mean *P*-value of all iterations for a given comparison. Two-tailed *P*-values<0.05 were considered statistically significant. Statistical analyses were performed with R v3.1.2.

SUPPLEMENTARY REFERENCES

1. Cheng D. T., Mitchell T. N., Zehir A., Shah R. H., Benayed R., Syed A., et al. Memorial Sloan Kettering-Integrated Mutation Profiling of Actionable Cancer Targets (MSK-IMPACT): A Hybridization Capture-Based Next-Generation Sequencing Clinical Assay for Solid Tumor Molecular Oncology. *J Mol Diagn.* **17**, 251-264 (2015).

2. Li H., Durbin R. Fast and accurate short read alignment with Burrows-Wheeler transform. *Bioinformatics.* **25**, 1754-1760 (2009).

3. McKenna A., Hanna M., Banks E., Sivachenko A., Cibulskis K., Kernytsky A., et al. The Genome Analysis Toolkit: a MapReduce framework for analyzing next-generation DNA sequencing data. *Genome Res.* **20**, 1297-1303 (2010).

4. Riaz N., Blecua P., Lim R. S., Shen R., Higginson D. S., Weinhold N., et al. Pan-cancer analysis of bi-allelic alterations in homologous recombination DNA repair genes. *Nat Commun.* **8**, 857 (2017).

5. Cibulskis K., Lawrence M. S., Carter S. L., Sivachenko A., Jaffe D., Sougnez C., et al. Sensitive detection of somatic point mutations in impure and heterogeneous cancer samples. *Nat Biotechnol.* **31**, 213-219 (2013).

6. Saunders C. T., Wong W. S., Swamy S., Becq J., Murray L. J., Cheetham R. K. Strelka: accurate somatic small-variant calling from sequenced tumor-normal sample pairs. *Bioinformatics.* **28**, 1811-1817 (2012).

7. Koboldt D. C., Zhang Q., Larson D. E., Shen D., McLellan M. D., Lin L., et al. VarScan 2: somatic mutation and copy number alteration discovery in cancer by exome sequencing. *Genome Res.* **22**, 568-576 (2012).

8. Narzisi G., Corvelo A., Arora K., Bergmann E. A., Shah M., Musunuri R., et al. Genome-wide somatic variant calling using localized colored de Bruijn graphs. *Comms Bio.* **1**, 1-9 (2018).

9. Narzisi G., O'Rawe J. A., Iossifov I., Fang H., Lee Y. H., Wang Z., et al. Accurate de novo and transmitted indel detection in exome-capture data using microassembly. *Nature methods.* **11**, 1033-1036 (2014).

10. Geyer F. C., Li A., Papanastasiou A. D., Smith A., Selenica P., Burke K. A., et al. Recurrent hotspot mutations in HRAS Q61 and PI3K-AKT pathway genes as drivers of breast adenomyoepitheliomas. *Nat Commun.* **9**, 1816 (2018).

11. Ng C. K. Y., Piscuoglio S., Geyer F. C., Burke K. A., Pareja F., Eberle C. A., et al. The Landscape of Somatic Genetic Alterations in Metaplastic Breast Carcinomas. *Clin Cancer Res.* **23**, 3859-3870 (2017).

12. Carter S. L., Cibulskis K., Helman E., McKenna A., Shen H., Zack T., et al. Absolute quantification of somatic DNA alterations in human cancer. *Nature biotechnology.* **30**, 413-421 (2012).

13. Landau D. A., Carter S. L., Stojanov P., McKenna A., Stevenson K., Lawrence M. S., et al. Evolution and impact of subclonal mutations in chronic lymphocytic leukemia. *Cell.* **152**, 714-726 (2013).

14. Weigelt B., Bi R., Kumar R., Blecua P., Mandelker D. L., Geyer F. C., et al. The Landscape of Somatic Genetic Alterations in Breast Cancers From ATM Germline Mutation Carriers. *J Natl Cancer Inst.* (2018).

15. Chang M. T., Asthana S., Gao S. P., Lee B. H., Chapman J. S., Kandoth C., et al. Identifying recurrent mutations in cancer reveals widespread lineage diversity and mutational specificity. *Nature biotechnology.* **34**, 155-163 (2016).

16. Martelotto L. G., Ng C. K., De Filippo M. R., Zhang Y., Piscuoglio S., Lim R. S., et al. Benchmarking mutation effect prediction algorithms using functionally validated cancer-related missense mutations. *Genome Biol.* **15**, 484 (2014).

17. Bailey M. H., Tokheim C., Porta-Pardo E., Sengupta S., Bertrand D., Weerasinghe A., et al. Comprehensive Characterization of Cancer Driver Genes and Mutations. *Cell.* **173**, 371-385 e318 (2018).

Supplementary Figure 1

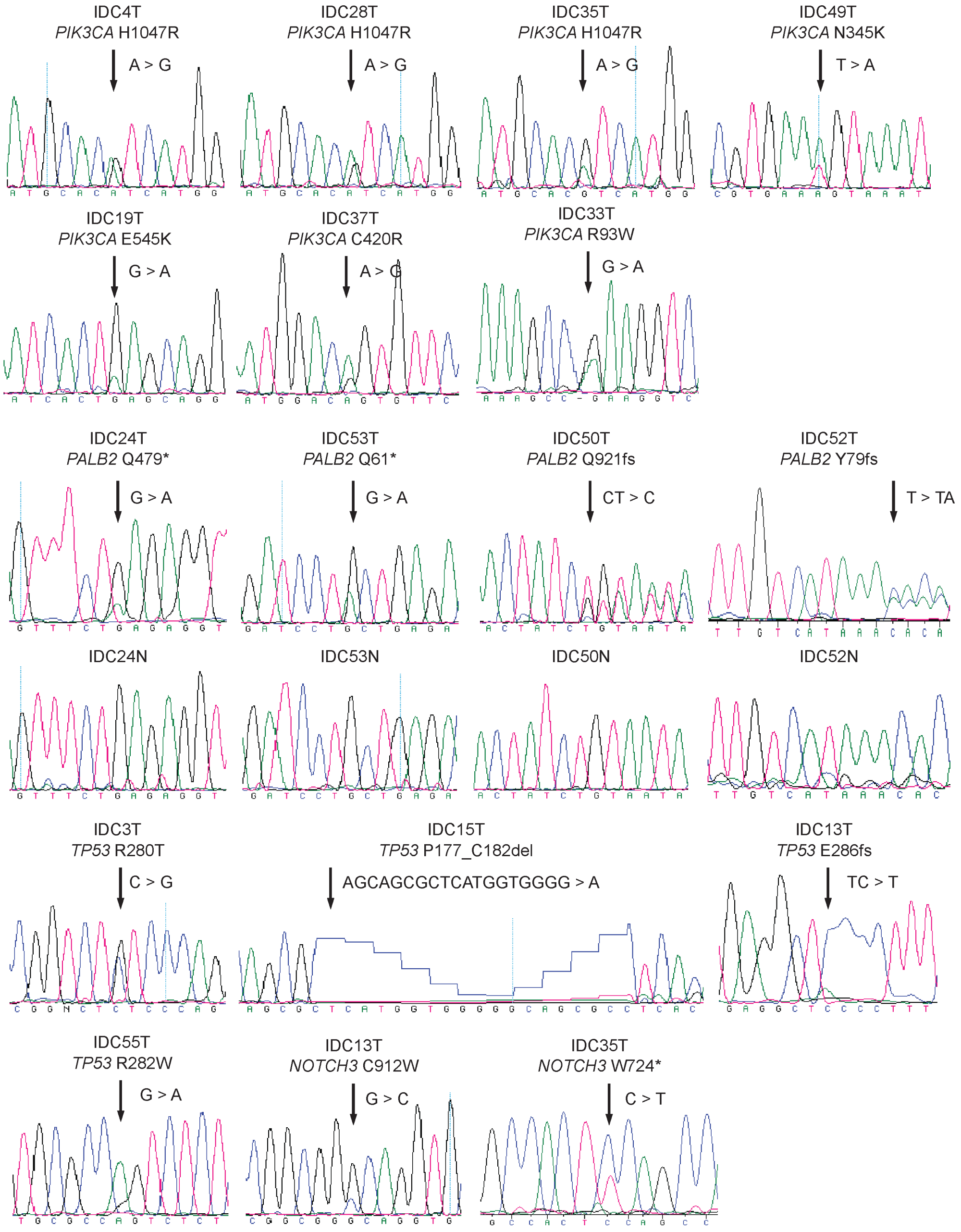

Supplementary Figure 1. Validation of *PIK3CA*, *TP53*, *PALB2* and *NOTCH3* somatic mutations using Sanger sequencing.

Representative Sanger sequence electropherograms of *PIK3CA* (n=7), *TP53* (n=4) and *NOTCH3* (n=2) somatic mutations in index tumor samples, and somatic *PALB2* (n=4) mutations in both index tumor and matched normal samples. The presence of the mutation is indicated by a black arrow.

Supplementary Figure 2

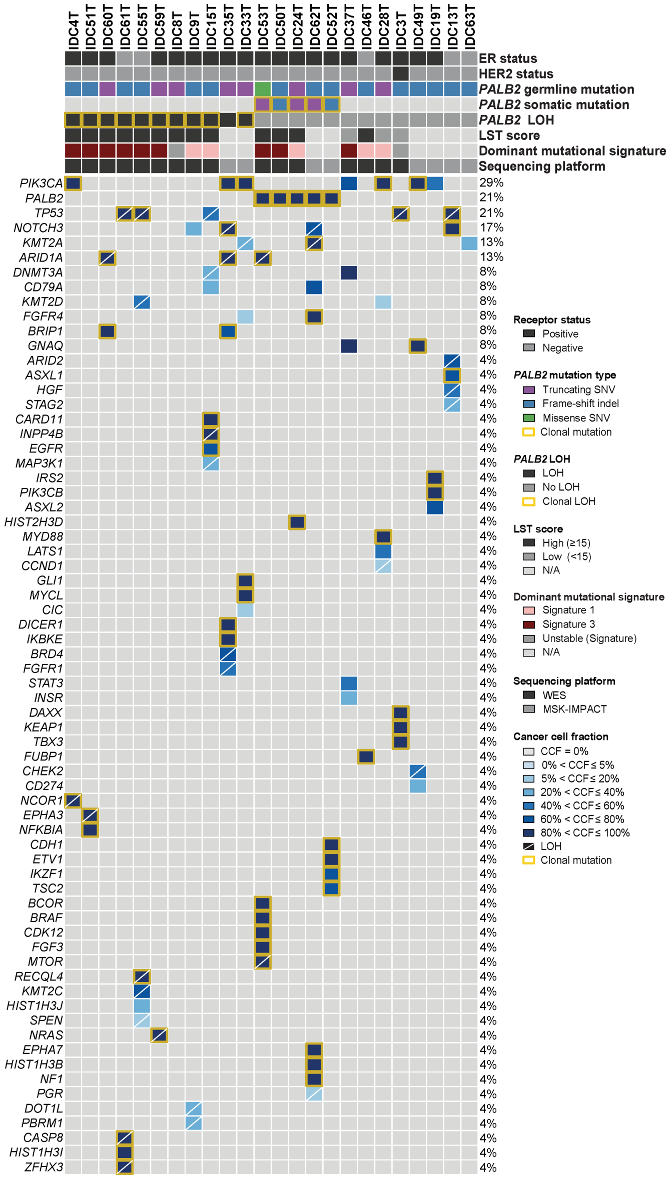

Supplementary Figure 2. Cancer cell fractions of somatic mutations affecting 410 cancer genes in *PALB2*-associated breast cancers.

Cancer cell fractions (i.e. bioinformatically inferred percentage of cancer cells harboring a given somatic mutation) of non-synonymous somatic mutations affecting 410 cancer genes as defined by ABSOLUTE in the 24 *PALB2*-associated breast cancers sequenced by whole-exome (n=16) or MSK-IMPACT (n=8) sequencing. Immunohistochemical features, *PALB2* germline mutation type, presence of a second somatic *PALB2* mutation or loss of heterozygosity (LOH) of the *PALB2* wild-type allele, LST score, dominant mutational signature and sequencing platform are shown in the phenobar (top), color-coded according to the legend. Clonal somatic mutations or clonal LOH of the *PALB2* wild-type allele are indicated by a yellow box. Cancer cell fractions are color-coded according to the legend; the presence of LOH of the wild-type allele of mutated genes other than *PALB2* is represented by a diagonal bar. CCF, cancer cell fraction; indel, small insertion/deletion; LOH, loss of heterozygosity; LST, large-scale transition; N/A, not assessed; SNV, single nucleotide variant; WES, whole-exome sequencing.

Supplementary Figure 3

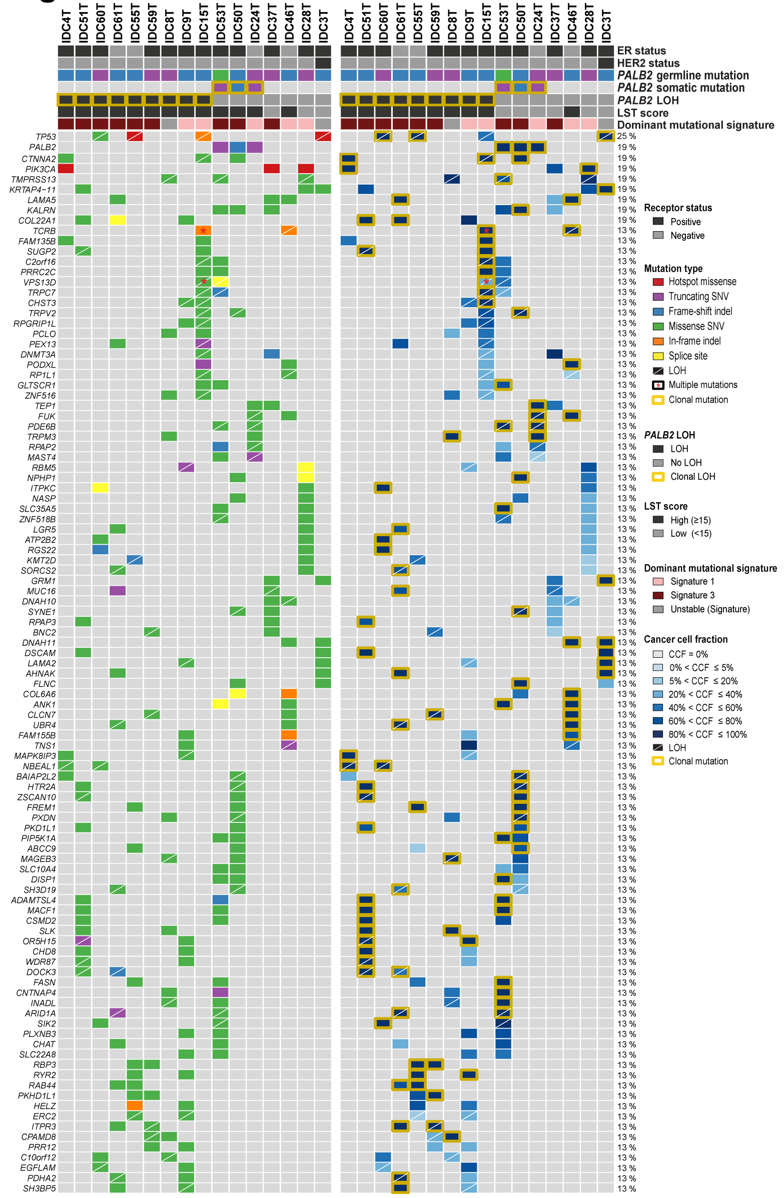

Supplementary Figure 3. Repertoire of recurrent somatic mutations and corresponding cancer cell fractions in *PALB2*-associated breast cancer subjected to whole-exome sequencing.

Repertoire of recurrent (n≥2) non-synonymous somatic mutations (left), and their corresponding cancer cell fraction (i.e. bioinformatically inferred percentage of cancer cells harboring a given somatic mutation; right) identified in the 16 *PALB2*-associated breast cancer profiled by whole-exome sequencing. Immunohistochemical features, *PALB2* germline mutation type, presence of a second somatic *PALB2* mutation or loss of heterozygosity (LOH) of the *PALB2* wild-type allele, LST score and dominant mutational signature are shown in the phenobar (top), color-coded according to the legend. Clonal somatic mutations or clonal LOH of the *PALB2* wild-type allele are indicated by a yellow box. The presence of multiple somatic mutations affecting the same gene is indicated by a red asterisk. CCF, cancer cell fraction; indel, small insertion/deletion; LOH, loss of heterozygosity; LST, large-scale state transition; SNV, single nucleotide variant.

Supplementary Figure 4

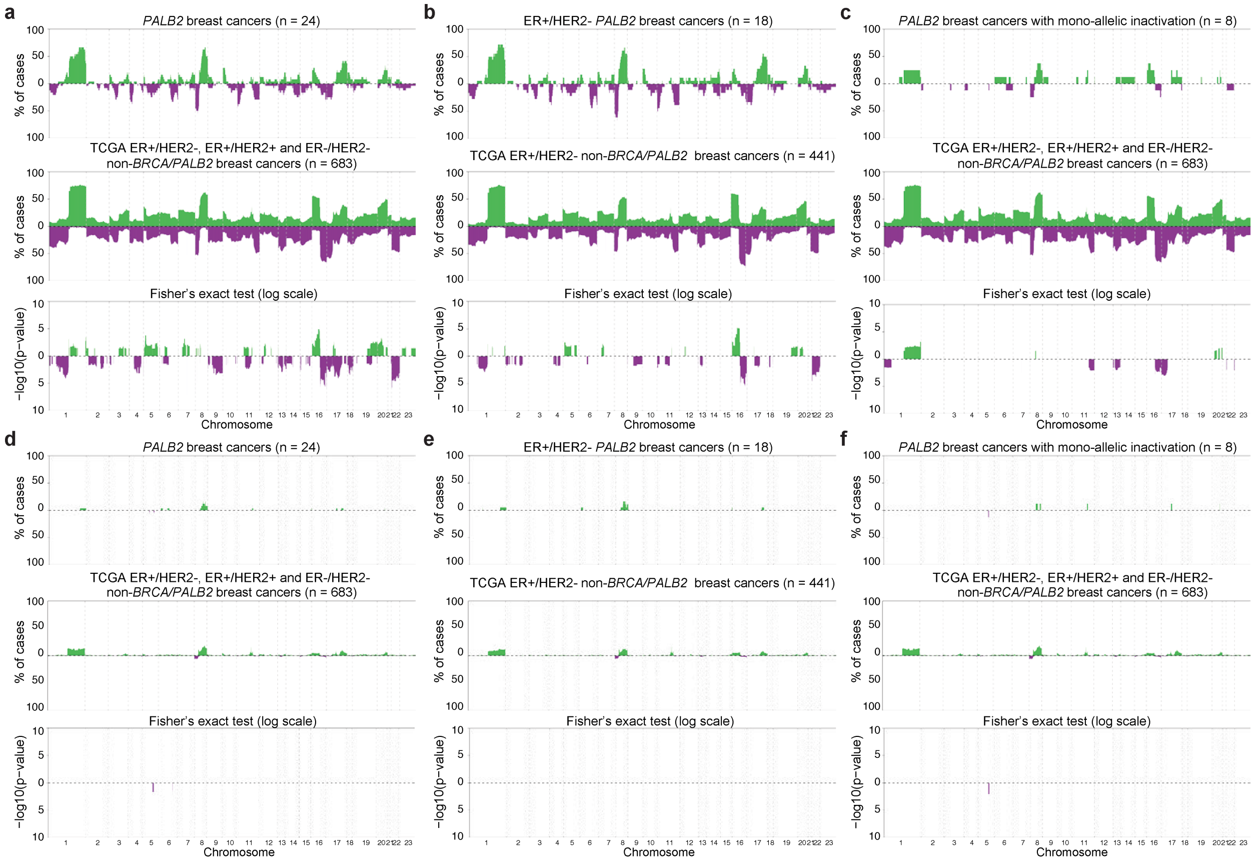

Supplementary Figure 4. Comparisons of the frequencies of copy number alterations in *PALB2*-associated breast cancers and non-*BRCA1/2/PALB2* breast cancers from TCGA.

Frequency plots and multi-Fisher's exact test comparisons of gains and losses **(a-c)**, amplifications and homozygous deletions **(d-f)** were performed between **(a, d)** the 24 *PALB2*-associated breast cancers and 683 non-*BRCA1/2/PALB2*-associated breast cancers (ER+/HER2-, ER+/HER2+ and ER-/HER2-), **(b, e)** between the 18 ER+/HER2- *PALB2*-associated breast cancers and 441 ER+/HER2- non-*BRCA1/2/PALB2*-associated breast cancers, **(c, f)** between the eight *PALB2*-associated breast cancers with mono-allelic *PALB2* alterations and 683 non-*BRCA1/2/PALB2*-associated breast cancers (ER+/HER2-, ER+/HER2+ and ER-/HER2-) from TCGA. The frequency of gains/amplifications (green bars) or losses/homozygous deletions (purple bars) for each gene is plotted on the y-axis, according to their genomic position on the x-axis. Inverse Log_{10} values of the Fisher's exact test *P*-values are plotted according to genomic location (x-axis).

Supplementary Figure 5

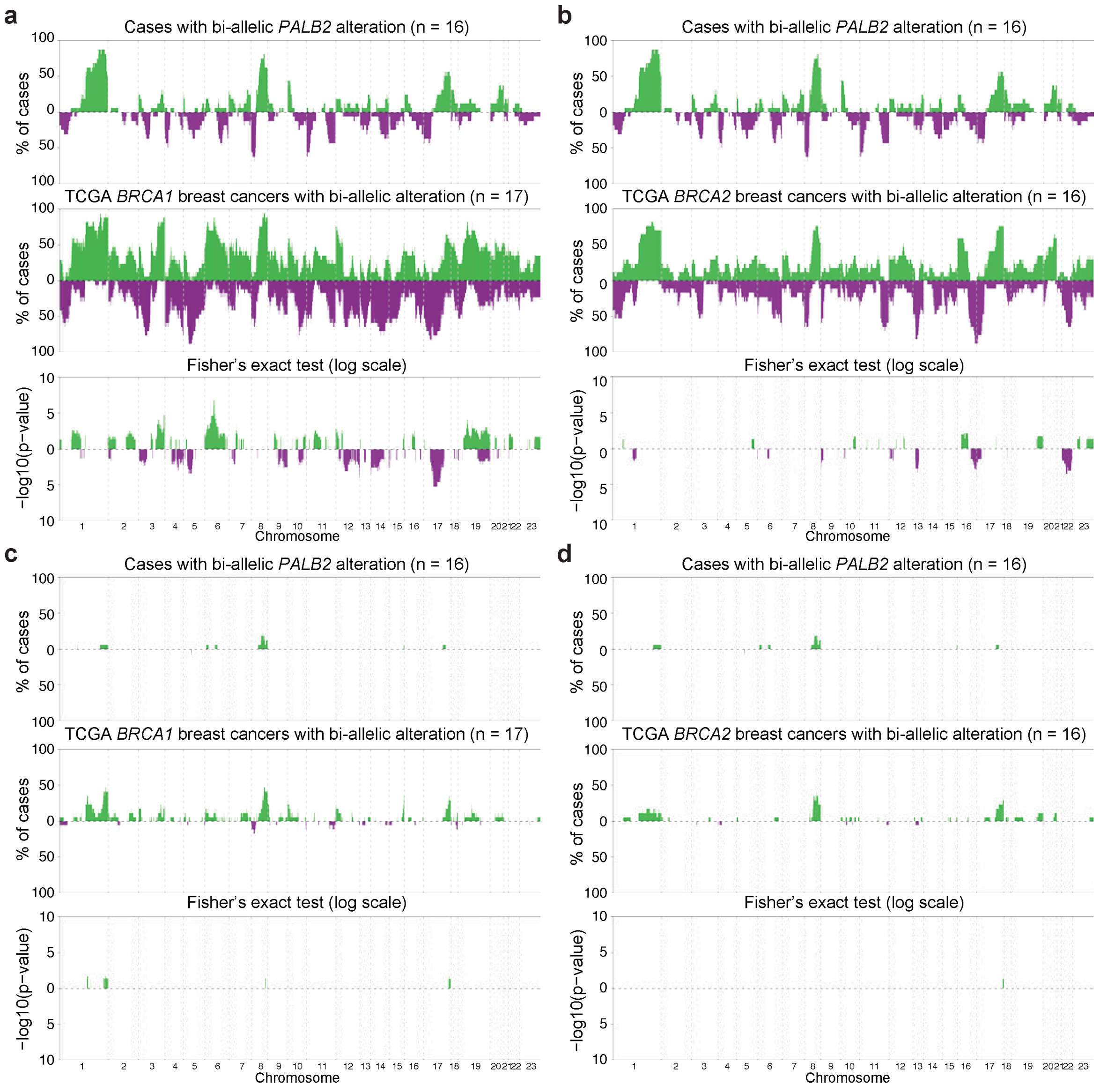

Supplementary Figure 5. Comparisons of the frequencies of copy number gains and losses in *PALB2*-associated breast cancers with bi-allelic *PALB2* alterations and *BRCA1*- and *BRCA2*-associated breast cancers with bi-allelic alterations from TCGA.
(a-d) Frequency plots and multi-Fisher's exact test comparisons of copy number gains and losses between **(a)** the 16 *PALB2*-associated breast cancers with bi-allelic *PALB2* alterations and the 17 *BRCA1*-associated breast cancers with bi-allelic *BRCA1* alterations, and **(b)** the 16 *BRCA2*-associated breast cancers with bi-allelic *BRCA2* alterations from TCGA. **(c-d)** Frequency plots and multi-Fisher's exact test comparisons of high-level gains/amplifications and homozygous deletions between **(c)** the 16 *PALB2*-associated breast cancers with bi-allelic *PALB2* alterations and the 17 *BRCA1*-associated breast cancers with bi-allelic *BRCA1* alterations, and **(d)** the 16 *BRCA2*-associated breast cancers with bi-allelic *BRCA2* alterations. The frequency of gains/amplifications (green bars) or losses/homozygous deletions (purple bars) for each gene is plotted on the y-axis, according to their genomic position on the x-axis. Inverse Log_{10} values of the Fisher's exact test *P*-values are plotted according to genomic location (x-axis).

Supplementary Figure 6

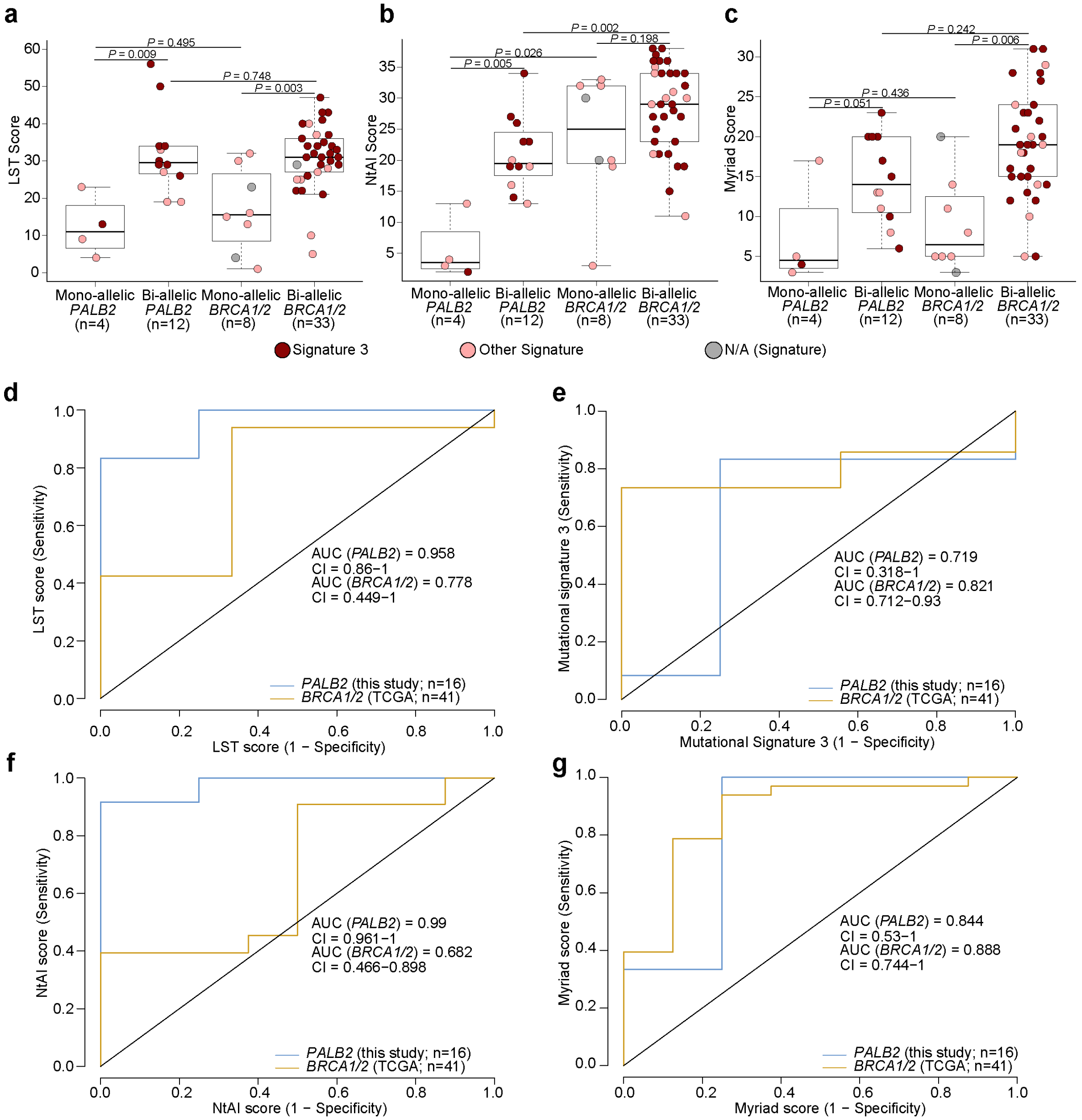

Supplementary Figure 6. Comparisons of measurements of homologous recombination deficiency in *PALB2*-associated breast cancers and *BRCA1/2*-associated breast cancers with mono-allelic and bi-allelic *PALB2* or *BRCA1/2* inactivation.

(a-c) Comparisons of **(a)** Large-scale transition (LST) score, **(b)** NtAI scores and **(c)** Myriad scores of the four *PALB2*-associated breast cancers with mono-allelic *PALB2* alterations, the 12 *PALB2*-associated breast cancers with bi-allelic *PALB2* alterations, the eight *BRCA1/2*-associated breast cancers with mono-allelic *BRCA1* or *BRCA2* alterations and the 33 *BRCA1/2*-associated breast cancers with bi-allelic *BRCA1* or *BRCA2* alterations. **(d-g)** Receiver operating characteristic (ROC) curves comparing performance of **(d)** LST scores, **(e)** mutational signature 3 (%), **(f)** NtAI scores and **(g)** Myriad scores in *PALB2*-associated breast cancers and *BRCA1/2*-associated breast cancers for the identification of *PALB2* and *BRCA1/2* bi-allelic inactivation. AUC, area under the ROC curve; CI, confidence interval.

Supplementary Table 1. Sequencing statistics of tumor and paired normal samples of *PALB2*-associated breast cancers sequenced by whole-exome or MSK-IMPACT targeted massively parallel sequencing.

Case ID	Sequencing type	Target Territory	Total Reads	Percent Selected Bases	Mean Target Coverage	Percent Target Bases 2X	Percent Target Bases 10X	Percent Target Bases 20X	Percent Target Bases 30X	Percent Target Bases 40X	Percent Target Bases 50X	Percent Target Bases 100X
IDC13N	MSK-IMPACT	1362579	17,587,599	67.86%	316	99.50%	99.25%	99.04%	98.79%	98.49%	98.04%	92.95%
IDC13T	MSK-IMPACT	1362579	21,890,639	54.60%	128	98.95%	98.25%	97.65%	96.58%	94.91%	92.47%	65.62%
IDC19N	MSK-IMPACT	1362579	104,539,241	75.41%	789	99.53%	99.43%	99.35%	99.28%	99.19%	99.12%	98.77%
IDC19T	MSK-IMPACT	1362579	38,378,568	51.96%	227	99.25%	98.77%	98.34%	97.75%	96.73%	95.05%	78.22%
IDC33N	MSK-IMPACT	1522367	22,171,814	84.58%	585	99.46%	99.34%	99.23%	99.12%	98.98%	98.83%	97.67%
IDC33T	MSK-IMPACT	1522367	15,369,766	82.02%	324	99.16%	98.76%	98.50%	98.29%	98.04%	97.66%	93.05%
IDC35N	MSK-IMPACT	1362579	14,260,504	67.93%	172	99.19%	98.58%	96.96%	93.67%	89.14%	83.72%	53.91%
IDC35T	MSK-IMPACT	1362579	6,712,966	55.84%	124	99.02%	97.84%	94.32%	88.10%	80.93%	73.46%	42.67%
IDC49N	MSK-IMPACT	1362579	54,791,577	77.98%	1452	99.51%	99.45%	99.40%	99.35%	99.31%	99.25%	99.02%
IDC49T	MSK-IMPACT	1362579	36,941,697	62.09%	236	98.87%	98.26%	97.89%	97.39%	96.65%	95.65%	87.28%
IDC52N	MSK-IMPACT	1362579	30,113,913	68.94%	572	99.53%	99.44%	99.33%	99.21%	99.11%	99.00%	97.77%
IDC52T	MSK-IMPACT	1362579	9,519,069	62.84%	73	98.35%	93.74%	83.48%	71.71%	60.98%	51.78%	24.03%
IDC62N	MSK-IMPACT	1522367	15,173,050	84.93%	502	99.42%	99.15%	98.95%	98.75%	98.55%	98.35%	97.58%
IDC62T	MSK-IMPACT	1522367	32,271,761	84.50%	904	99.46%	99.30%	99.16%	99.06%	98.96%	98.86%	98.41%
IDC63N	MSK-IMPACT	1522367	15,767,985	89.13%	518	99.46%	99.26%	99.09%	98.96%	98.82%	98.65%	97.89%
IDC63T	MSK-IMPACT	1522367	21,077,959	89.66%	678	99.28%	99.05%	98.85%	98.67%	98.53%	98.40%	97.72%
IDC15N	Whole exome	51542852	133,574,121	85.80%	146	93.49%	91.44%	89.75%	87.45%	84.53%	81.20%	60.98%
IDC15T	Whole exome	51542852	67,920,645	81.58%	40	87.47%	74.55%	61.01%	47.91%	36.45%	27.26%	6.62%
IDC24N	Whole exome	51542852	75,039,053	85.83%	88	91.64%	85.64%	79.57%	73.71%	67.56%	60.98%	30.49%
IDC24T	Whole exome	51542852	109,782,207	87.43%	130	92.15%	87.81%	83.63%	79.78%	76.02%	72.20%	50.30%
IDC28N	Whole exome	51542852	88,885,131	89.73%	106	92.14%	88.21%	83.49%	78.94%	74.43%	69.75%	43.19%
IDC28T	Whole exome	51542852	100,553,535	87.98%	105	91.69%	86.60%	81.46%	76.63%	71.71%	66.44%	38.35%
IDC37N	Whole exome	51189318	233,784,412	82.85%	247	99.67%	99.28%	98.92%	98.52%	97.98%	97.22%	88.66%
IDC37T	Whole exome	51189318	250,533,924	78.34%	268	99.79%	99.57%	99.24%	98.70%	97.80%	96.52%	85.10%
IDC3N	Whole exome	51542852	86,590,460	82.29%	85	92.30%	86.29%	77.82%	68.33%	58.12%	48.31%	21.15%
IDC3T	Whole exome	51542852	110,163,811	90.09%	136	92.18%	88.46%	84.66%	81.29%	78.07%	74.87%	57.27%
IDC46N	Whole exome	51542852	139,890,230	83.78%	155	93.59%	91.31%	89.46%	86.99%	84.05%	80.79%	61.75%
IDC46T	Whole exome	51542852	44,683,165	88.43%	33	85.88%	70.18%	52.42%	37.15%	26.07%	18.72%	5.14%
IDC4N	Whole exome	51542852	107,232,015	85.31%	122	93.31%	91.13%	88.67%	85.40%	81.61%	77.58%	54.44%
IDC4T	Whole exome	51542852	114,347,035	89.63%	139	92.32%	88.89%	85.18%	81.81%	78.57%	75.34%	57.72%
IDC50N	Whole exome	51542852	123,171,969	85.09%	136	93.25%	90.97%	88.94%	86.21%	82.96%	79.38%	58.75%
IDC50T	Whole exome	51542852	72,671,924	84.91%	77	91.18%	83.69%	75.58%	67.74%	60.03%	52.73%	26.06%
IDC51N	Whole exome	51542852	112,888,954	81.16%	118	93.54%	90.84%	88.22%	84.66%	80.34%	75.45%	48.79%
IDC51T	Whole exome	51542852	66,148,714	87.59%	70	90.09%	81.77%	73.48%	64.98%	56.15%	47.52%	19.19%
IDC53N	Whole exome	51542852	186,199,465	84.58%	193	93.87%	91.33%	89.56%	87.52%	85.14%	82.40%	66.03%
IDC53T	Whole exome	51542852	68,290,444	80.92%	69	91.90%	85.02%	75.96%	66.47%	57.10%	48.43%	20.94%
IDC55N	Whole exome	51189318	165,293,498	62.27%	139	99.69%	99.17%	98.38%	96.97%	94.63%	91.24%	64.21%
IDC55T	Whole exome	51189318	267,310,420	81.28%	289	99.77%	99.53%	99.18%	98.69%	97.94%	96.88%	87.42%
IDC59N	Whole exome	51189318	185,081,923	84.63%	173	99.80%	99.49%	98.86%	97.81%	96.22%	94.06%	75.86%
IDC59T	Whole exome	51189318	175,236,053	91.76%	213	99.80%	99.51%	99.00%	98.20%	97.04%	95.45%	81.82%
IDC60N	Whole exome	51189318	73,994,558	65.70%	46	96.57%	91.44%	81.89%	68.30%	52.88%	38.47%	4.96%
IDC60T	Whole exome	51189318	89,242,346	57.28%	46	96.68%	92.05%	83.10%	69.18%	52.84%	37.56%	4.33%
IDC61N	Whole exome	51189318	152,388,926	57.82%	86	94.27%	86.65%	81.91%	76.44%	70.40%	64.04%	33.92%
IDC61T	Whole exome	51189318	143,124,548	50.78%	77	94.81%	85.35%	78.76%	71.65%	64.33%	57.05%	27.50%
IDC8N	Whole exome	51542852	101,656,174	86.68%	121	93.09%	90.79%	87.94%	84.45%	80.75%	76.96%	55.65%
IDC8T	Whole exome	51542852	124,287,664	89.61%	151	92.46%	89.37%	86.03%	83.02%	80.17%	77.37%	62.44%
IDC9N	Whole exome	51542852	122,188,395	85.89%	142	93.35%	91.31%	89.22%	86.56%	83.52%	80.31%	62.76%
IDC9T	Whole exome	51542852	107,445,459	86.92%	119	91.72%	86.91%	82.76%	79.16%	75.63%	72.00%	51.24%

Supplementary Table 2. Clonal and subclonal variants, variant allele fraction range and tumor purity of the *PALB2*-associated breast cancers in this study

Sample ID	# Clonal Variants	# Subclonal Variants	# Total Variants	VAF Min	VAF Max	Purity	*PALB2* Status
IDC13T	3	3	6	6.38%	29.63%	0.47	Monoallelic
IDC15T	73	82	155	4.17%	75%	0.76	Biallelic
IDC19T	2	2	4	10.48%	26.09%	0.47	Monoallelic
IDC24T	32	22	54	4.94%	38.46%	0.43	Biallelic
IDC28T	9	58	67	4.11%	23.33%	0.52	Monoallelic
IDC33T	3	3	6	4.64%	40.93%	0.71	Biallelic
IDC35T	6	2	8	17.02%	64.52%	0.25	Biallelic
IDC37T	9	70	79	2.92%	23.08%	0.41	Monoallelic
IDC3T	27	10	37	3.45%	43.71%	0.45	Monoallelic
IDC46T	55	18	73	5.75%	77.65%	0.69	Monoallelic
IDC49T	2	2	4	5.71%	35%	0.51	Monoallelic
IDC4T	41	35	76	3.55%	76.09%	0.68	Biallelic
IDC50T	110	37	147	3.07%	81.82%	0.7	Biallelic
IDC51T	109	22	131	5.68%	64.71%	0.55	Biallelic
IDC52T	5	0	5	14.29%	41.94%	0.56	Biallelic
IDC53T	124	71	195	3.28%	67.57%	0.55	Biallelic
IDC55T	65	40	105	2.84%	73.51%	0.79	Biallelic
IDC59T	33	39	72	3.72%	77.78%	0.68	Biallelic
IDC60T	64	39	103	4.96%	47.69%	0.44	Biallelic
IDC61T	47	39	86	2.84%	86.67%	0.68	Biallelic
IDC62T	7	3	10	3.41%	53.45%	0.67	Biallelic
IDC63T	0	1	1	6.99%	6.99%	0.3	Monoallelic
IDC8T	15	26	41	5.14%	40%	0.4	Biallelic
IDC9T	25	136	161	3.55%	83.33%	0.6	Biallelic

VAF, variant allele fraction

Supplementary Table 3. List of non-synonymous somatic mutations identified in the *PALB2*- associated breast cancers sequenced by whole-exome sequencing or MSK-IMPACT targeted massively parallel sequencing.

Case ID	Gene	Amino Acid Change	Mutation Type	Chromosome	Genomic Position	Reference Allele	Alternate Allele	Depth at Mutation	Mutant Allele Fraction	Mutation Taster	FATHMM	CHASM (Breast)	Pathogenicity	Hotspot Residue	Loss of Heterozygosity (LOH)	Cancer Cell Fraction (CCF) (ABSOLUTE)	Probability of mutation being clonal	Lower Bound of 95% Confidence Interval	Clonal/ Subclonal Mutation	Cancer Gene Census	127 Significantly Mutated Genes (Kandoth et al)	Cancer5000-S genes (Lawrence et al)	MSK-IMPACT 410 Cancer Genes	Sequencing platform	Forward Primer	Reverse Primer
IDC13T	*ASXL1*	p.G645Vfs*58	Frame_Shift_Del	20	31022441	AG	A	58	10.34%	.	.	.	likely_pathogenic	.	.	0.78	0.51879	34%	Clonal				YES	MSK-IMPACT		
IDC13T	*TP53*	p.E286Kfs*59	Frame_Shift_Del	17	7577081	TC	T	89	29.21%				likely_pathogenic	.	loh	1	0.95348	81%	Clonal	TRUE	TRUE	TRUE	YES	MSK-IMPACT	GCTTCTTGTCCTGCTTGCTT	CTACTGGGACGGAACAGCTT
IDC13T	*ARID2*	p.T1002I	Missense_Mutation	12	46244911	C	T	43	11.63%	Deleterious	PASSENGER/OTHER	Passenger	passenger	.	loh	0.65	0.39466	27%	Subclonal	TRUE		TRUE	YES	MSK-IMPACT		
IDC13T	*HGF*	p.P23S	Missense_Mutation	7	81399221	G	A	56	8.93%	Neutral	PASSENGER/OTHER	Passenger	passenger	.	loh	0.59	0.31956	25%	Subclonal		TRUE		YES	MSK-IMPACT		
IDC13T	*NOTCH3*	p.C912W	Missense_Mutation	19	15292443	G	C	81	29.63%	Deleterious	CANCER	Passenger	likely_pathogenic	.		1	0.8704	82%	Clonal	.	TRUE	.	YES	MSK-IMPACT	CAGTGGAAACCCATTCCATC	CCCTTCTCAGCCTCAGACAC
IDC13T	*STAG2*	p.G719R	Missense_Mutation	X	123200083	G	A	141	6.38%	Deleterious	PASSENGER/OTHER	Passenger	passenger	.	loh	0.36	0.00074	19%	Subclonal	TRUE	TRUE	TRUE	YES	MSK-IMPACT		
IDC15T	*DST*	p.K2570Lfs*6	Frame_Shift_Del	6	56418009	AATTTT	A	33	15.15%	.	.	.	passenger	.	.	0.79	0.51928	32%	Clonal	.	.	.	.	WES		
IDC15T	*KRT74*	p.L125Sfs*23	Frame_Shift_Del	12	52967188	AG	A	48	20.83%	.	.	.	likely_pathogenic	.	loh	0.67	0.31031	37%	Subclonal	.	.	.	.	WES		
IDC15T	*TADA1*	p.G98Efs*39	Frame_Shift_Del	1	166833097	TC	T	63	11.11%	.	.	.	passenger	.	.	1	0.69084	45%	Clonal	.	.	.	.	WES		
IDC15T	*TBC1D10A*	p.S177Pfs*43	Frame_Shift_Del	22	30691741	GAC	G	61	18.03%	.	.	.	likely_pathogenic	.	loh	0.58	0.10881	33%	Subclonal	.	.	.	.	WES		
IDC15T	*EIF2B5*	p.W111Vfs*27	Frame_Shift_Ins	3	183855413	C	CA	20	15.00%	.	.	.	passenger	.	.	0.78	0.5057	24%	Clonal	.	.	.	.	WES		
IDC15T	*NLE1*	p.D425Gfs*11	Frame_Shift_Ins	17	33460458	T	TC	56	28.57%	.	.	.	likely_pathogenic	.	loh	0.92	0.67725	56%	Clonal	.	.	.	.	WES		
IDC15T	*RNF38*	p.P327Hfs*21	Frame_Shift_Ins	9	36353258	G	GT	13	15.38%	.	.	.	likely_pathogenic	.	loh	0.49	0.30789	14%	Subclonal	.	.	.	.	WES		
IDC15T	*MATN1*	p.L18del	In_Frame_Del	1	31196345	GAGC	G	26	11.54%	.	.	.	passenger	.	.	0.49	0.25064	17%	Subclonal	.	.	.	.	WES		
IDC15T	*NR2F2*	p.Q75del	In_Frame_Del	15	96875542	AAGC	A	24	16.67%	.	.	.	likely_pathogenic	.	loh	0.37	0.0274	15%	Subclonal	.	.	.	.	WES		
IDC15T	*TCRB*	p.P2017del	In_Frame_Del	7	141920357	ACCC	A	43	41.86%	.	.	.	passenger	.	.	1	0.60904	79%	Clonal	.	.	.	.	WES		
IDC15T	*TP53*	p.P177_C182del	In_Frame_Del	17	7578383	AGCAGCGCTCATGGTGGGG	A	35	14.29%	.	.	.	likely_pathogenic	.	loh	0.6	0.32597	26%	Subclonal	TRUE	TRUE	TRUE	YES	WES	CACTCGGATAAGATGCTGAGG	CACATGACGGAGGTTGTGAG
IDC15T	*TCRB*	p.P1866_S1867insT	In_Frame_Ins	7	141919907	C	CCAA	55	18.18%	.	.	.	passenger	.	.	1	0.7747	56%	Clonal	.	.	.	.	WES		
IDC15T	*A2ML1*	p.P217L	Missense_Mutation	12	8988857	C	T	47	8.51%	Deleterious	PASSENGER/OTHER	Passenger	passenger	.	loh	0.53	0.2705	20%	Subclonal	.	.	.	.	WES		
IDC15T	*ABCA2*	p.R224W	Missense_Mutation	9	139916351	G	A	42	9.52%	Neutral	PASSENGER/OTHER	Passenger	passenger	.	loh	0.31	0.0069	12%	Subclonal	.	.	.	.	WES		
IDC15T	*ABCA2*	p.I626V	Missense_Mutation	9	139912747	T	C	68	63.24%	Deleterious	PASSENGER/OTHER	Passenger	passenger	.	loh	1	0.96249	90%	Clonal	.	.	.	.	WES		
IDC15T	*ABCA9*	p.L362F	Missense_Mutation	17	67031429	C	A	5	60.00%	Neutral	PASSENGER/OTHER	Passenger	passenger	.	loh	1	0.78066	37%	Clonal	.	.	.	.	WES		
IDC15T	*ADCK5*	p.R560C	Missense_Mutation	8	145618224	C	T	142	4.23%	Neutral	PASSENGER/OTHER	Passenger	passenger	.	.	0.22	0	10%	Subclonal	.	.	.	.	WES		
IDC15T	*AKAP12*	p.D1277E	Missense_Mutation	6	151673357	C	A	44	38.64%	Neutral	PASSENGER/OTHER	Passenger	passenger	.	.	1	0.92556	75%	Clonal	.	.	.	.	WES		
IDC15T	*ANKS6*	p.R686W	Missense_Mutation	9	101530449	G	A	17	23.53%	Neutral	CANCER	Passenger	passenger	.	loh	0.76	0.49371	28%	Subclonal	.	.	.	.	WES		
IDC15T	*ARHGEF10L*	p.E541K	Missense_Mutation	1	17958852	G	A	19	36.84%	Deleterious	PASSENGER/OTHER	Passenger	passenger	.	loh	1	0.72557	49%	Clonal	.	.	.	.	WES		
IDC15T	*ASCC2*	p.R362M	Missense_Mutation	22	30202474	C	A	11	36.36%	Deleterious	PASSENGER/OTHER	Passenger	passenger	.	loh	1	0.66857	36%	Clonal	.	.	.	.	WES		
IDC15T	*C2CD4D*	p.D178N	Missense_Mutation	1	151810934	C	T	92	5.43%	Neutral	PASSENGER/OTHER	Passenger	passenger	.	.	0.56	0.27809	23%	Subclonal	.	.	.	.	WES		
IDC15T	*C2orf16*	p.T44N	Missense_Mutation	2	27799570	C	A	33	21.21%	Neutral	PASSENGER/OTHER	Passenger	passenger	.	loh	0.89	0.60394	41%	Clonal	.	.	.	.	WES		
IDC15T	*C2orf78*	p.E632D	Missense_Mutation	2	74043246	G	T	32	12.50%	Neutral	.	Passenger	passenger	.	loh	0.53	0.25557	21%	Subclonal	.	.	.	.	WES		
IDC15T	*CARD11*	p.I778T	Missense_Mutation	7	2959183	A	G	41	21.95%	Deleterious	PASSENGER/OTHER	Passenger	passenger	.	.	0.92	0.63977	46%	Clonal	TRUE	.	TRUE	YES	WES		
IDC15T	*CC2D1A*	p.P194A	Missense_Mutation	19	14024283	C	G	168	8.33%	Deleterious	PASSENGER/OTHER	Passenger	passenger	.	.	0.68	0.31563	41%	Subclonal	.	.	.	.	WES		
IDC15T	*CCDC33*	p.V164I	Missense_Mutation	15	74560743	G	A	46	8.70%	Neutral	PASSENGER/OTHER	Passenger	passenger	.	loh	0.37	0.04428	14%	Subclonal	.	.	.	.	WES		
IDC15T	*CCDC88A*	p.E1812K	Missense_Mutation	2	55522847	C	T	29	13.79%	Deleterious	PASSENGER/OTHER	Passenger	passenger	.	loh	0.58	0.32869	22%	Subclonal	.	.	.	.	WES		
IDC15T	*CD79A*	p.R143Q	Missense_Mutation	19	42383653	G	A	47	8.51%	Neutral	CANCER	Passenger	passenger	.	.	0.27	0.00191	11%	Subclonal	TRUE	.	.	YES	WES		
IDC15T	*CDC6*	p.G196R	Missense_Mutation	17	38447846	G	A	14	21.43%	Deleterious	PASSENGER/OTHER	Passenger	passenger	.	loh	0.69	0.44706	22%	Subclonal	.	.	.	.	WES		
IDC15T	*CHIT1*	p.H410Y	Missense_Mutation	1	203186190	G	A	14	57.14%	Neutral	PASSENGER/OTHER	Passenger	passenger	.	.	1	0.87311	62%	Clonal	.	.	.	.	WES		
IDC15T	*CHST3*	p.V202L	Missense_Mutation	10	73767393	G	C	46	69.57%	Neutral	PASSENGER/OTHER	Passenger	passenger	.	loh	1	0.92923	87%	Clonal	.	.	.	.	WES		
IDC15T	*CLPTM1*	p.R568W	Missense_Mutation	19	45495637	C	T	24	12.50%	Deleterious	CANCER	Driver	likely_pathogenic	.	.	0.4	0.11982	14%	Subclonal	.	.	.	.	WES		
IDC15T	*CPN2*	p.G530S	Missense_Mutation	3	194061844	C	T	56	8.93%	Deleterious	PASSENGER/OTHER	Passenger	passenger	.	.	0.47	0.12848	20%	Subclonal	.	.	.	.	WES		
IDC15T	*CTNNA2*	p.Q686H	Missense_Mutation	2	80816479	G	C	26	26.92%	Deleterious	PASSENGER/OTHER	Passenger	passenger	.	loh	1	0.70528	47%	Clonal	.	.	.	.	WES		
IDC15T	*DCAF8L2*	p.L448H	Missense_Mutation	X	27766355	T	A	29	44.83%	.	PASSENGER/OTHER	Passenger	passenger	.	loh	1	0.82834	67%	Clonal	.	.	.	.	WES		
IDC15T	*DLGAP1*	p.L481M	Missense_Mutation	18	3729285	G	T	58	25.86%	Deleterious	CANCER	Passenger	likely_pathogenic	.	loh	0.83	0.56817	51%	Clonal	.	.	.	.	WES		
IDC15T	*DNAH2*	p.E1917K	Missense_Mutation	17	7683501	G	A	29	13.79%	Deleterious	PASSENGER/OTHER	Passenger	passenger	.	loh	0.58	0.32869	22%	Subclonal	.	.	.	.	WES		
IDC15T	*DNAH9*	p.H460Q	Missense_Mutation	17	11532763	C	G	14	42.86%	Deleterious	PASSENGER/OTHER	Passenger	passenger	.	loh	1	0.83629	51%	Clonal	.	.	.	.	WES		
IDC15T	*DNAJB2*	p.R178H	Missense_Mutation	2	220147942	G	A	44	11.36%	Deleterious	PASSENGER/OTHER	Passenger	passenger	.	loh	0.37	0.0149	16%	Subclonal	.	.	.	.	WES		
IDC15T	*DNMT3A*	p.R318W	Missense_Mutation	2	25470522	G	A	49	8.16%	Deleterious	PASSENGER/OTHER	Passenger	passenger	.	loh	0.34	0.02752	13%	Subclonal	TRUE	TRUE	TRUE	YES	WES		
IDC15T	*DOCK6*	p.D523Y	Missense_Mutation	19	11352797	C	A	18	38.89%	Deleterious	PASSENGER/OTHER	Passenger	passenger	.	.	1	0.71243	59%	Clonal	.	.	.	.	WES		
IDC15T	*DUSP27*	p.S891C	Missense_Mutation	1	167097039	A	T	82	14.63%	Neutral	PASSENGER/OTHER	Passenger	passenger	.	.	1	0.82741	62%	Clonal	.	.	.	.	WES		
IDC15T	*DYRK1B*	p.D239N	Missense_Mutation	19	40319029	C	T	44	9.09%	Deleterious	CANCER	Passenger	likely_pathogenic	.	.	0.66	0.41635	24%	Subclonal	.	.	.	.	WES		
IDC15T	*EFCAB4B*	p.R544W	Missense_Mutation	12	3742875	G	A	40	12.50%	Deleterious	CANCER	Passenger	likely_pathogenic	.	loh	0.78	0.50937	31%	Clonal	.	.	.	.	WES		
IDC15T	*EGFR*	p.N206T	Missense_Mutation	7	55219044	A	C	26	15.38%	Deleterious	CANCER	Passenger	likely_pathogenic	.	.	0.8	0.52237	29%	Clonal	TRUE	TRUE	TRUE	YES	WES		
IDC15T	*EHD2*	p.R30H	Missense_Mutation	19	48219958	G	A	25	16.00%	Deleterious	PASSENGER/OTHER	Passenger	passenger	.	.	0.51	0.22162	20%	Subclonal	.	.	.	.	WES		
IDC15T	*EHMT2*	p.S451T	Missense_Mutation	6	31856389	C	G	47	23.40%	Deleterious	CANCER	Passenger	likely_pathogenic	.	loh	0.99	0.69136	51%	Clonal	.	.	.	.	WES		
IDC15T	*ELF2*	p.R326C	Missense_Mutation	4	139981623	G	A	45	8.89%	Deleterious	PASSENGER/OTHER	Passenger	passenger	.	loh	0.46	0.17169	18%	Subclonal	.	.	.	.	WES		
IDC15T	*FAM135B*	p.V1308I	Missense_Mutation	8	139149483	C	T	54	29.63%	Deleterious	PASSENGER/OTHER	Passenger	passenger	.	.	1	0.90991	74%	Clonal	.	.	.	.	WES		
IDC15T	*FGL2*	p.D259N	Missense_Mutation	7	76826141	C	T	46	17.39%	Deleterious	PASSENGER/OTHER	Passenger	passenger	.	.	0.91	0.6159	43%	Clonal	.	.	.	.	WES		
IDC15T	*FREM3*	p.G1612S	Missense_Mutation	4	144616995	C	T	35	11.43%	Deleterious	PASSENGER/OTHER	Passenger	passenger	.	loh	0.6	0.35094	23%	Subclonal	.	.	.	.	WES		
IDC15T	*FUT11*	p.I324T	Missense_Mutation	10	75533210	T	C	40	30.00%	Deleterious	PASSENGER/OTHER	Passenger	passenger	.	loh	1	0.78913	60%	Clonal	.	.	.	.	WES		
IDC15T	*GATAD2B*	p.P542L	Missense_Mutation	1	153784230	G	A	5	60.00%	Deleterious	PASSENGER/OTHER	Passenger	passenger	.	.	1	0.41038	38%	Subclonal	.	.	.	.	WES		
IDC15T	*GLTSCR1*	p.T1496M	Missense_Mutation	19	48205476	C	T	43	9.30%	Neutral	PASSENGER/OTHER	Passenger	passenger	.	.	0.3	0.00537	12%	Subclonal	.	.	.	.	WES		
IDC15T	*GMEB1*	p.D315Y	Missense_Mutation	1	29037076	G	T	26	15.38%	Neutral	PASSENGER/OTHER	Passenger	passenger	.	.	0.65	0.40436	25%	Subclonal	.	.	.	.	WES		
IDC15T	*GNA13*	p.R356H	Missense_Mutation	17	63010442	C	T	40	10.00%	Deleterious	CANCER	Passenger	likely_pathogenic	.	loh	0.42	0.10445	17%	Subclonal	.	.	TRUE	.	WES		
IDC15T	*GPRASP2*	p.R655S	Missense_Mutation	X	101971762	G	C	28	28.57%	Deleterious	PASSENGER/OTHER	Driver	likely_pathogenic	.	loh	0.92	0.63288	44%	Clonal	.	.	.	.	WES		
IDC15T	*GRM2*	p.E555K	Missense_Mutation	3	51749452	G	A	38	10.53%	Deleterious	PASSENGER/OTHER	Passenger	passenger	.	loh	0.44	0.13452	17%	Subclonal	.	.	.	.	WES		
IDC15T	*H6PD*	p.A612V	Missense_Mutation	1	9324387	C	T	123	5.69%	Neutral	PASSENGER/OTHER	Passenger	passenger	.	.	0.24	0	11%	Subclonal	.	.	.	.	WES		
IDC15T	*HELZ2*	p.R1494W	Missense_Mutation	20	62195695	G	A	86	5.81%	Neutral	PASSENGER/OTHER	Passenger	passenger	.	.	0.42	0.07744	18%	Subclonal	.	.	.	.	WES		
IDC15T	*hsa-mir-150*	p.G85S	Missense_Mutation	19	50004033	G	A	18	16.67%	.	.	.	passenger	.	.	0.54	0.29981	18%	Subclonal	.	.	.	.	WES		
IDC15T	*IL13RA2*	p.S218L	Missense_Mutation	X	114245260	G	A	12	33.33%	Deleterious	PASSENGER/OTHER	Passenger	passenger	.	loh	1	0.64063	35%	Clonal	.	.	.	.	WES		
IDC15T	*INPP4B*	p.W293C	Missense_Mutation	4	143130137	C	A	24	33.33%	Deleterious	PASSENGER/OTHER	Passenger	passenger	.	loh	1	0.84984	57%	Clonal	.	.	.	YES	WES		
IDC15T	*ITSN1*	p.A1096T	Missense_Mutation	21	35201984	G	A	26	15.38%	Deleterious	PASSENGER/OTHER	Driver	likely_pathogenic	.	.	1	0.64139	34%	Clonal	.	.	.	.	WES		
IDC15T	*KCNH5*	p.R813Q	Missense_Mutation	14	63174755	C	T	24	16.67%	Deleterious	PASSENGER/OTHER	Passenger	passenger	.	loh	0.54	0.25321	21%	Subclonal	.	.	.	.	WES		
IDC15T	*KCTD3*	p.F99L	Missense_Mutation	1	215751052	T	G	45	51.11%	Deleterious	PASSENGER/OTHER	Passenger	passenger	.	.	1	0.93249	82%	Clonal	.	.	.	.	WES		
IDC15T	*KDM3A*	p.G1021E	Missense_Mutation	2	86711249	G	A	23	17.39%	Deleterious	PASSENGER/OTHER	Passenger	passenger	.	loh	0.73	0.47804	27%	Subclonal	.	.	.	.	WES		
IDC15T	*KIFAP3*	p.R72M	Missense_Mutation	1	170015957	C	A	35	25.71%	Deleterious	PASSENGER/OTHER	Passenger	passenger	.	.	1	0.8196	63%	Clonal	.	.	.	.	WES		
IDC15T	*KLHL6*	p.R515W	Missense_Mutation	3	183210303	G	A	59	8.47%	Deleterious	PASSENGER/OTHER	Passenger	passenger	.	.	0.44	0.0962	19%	Subclonal	.	.	.	.	WES		
IDC15T	*LMAN2L*	p.R173K	Missense_Mutation	2	97378866	C	T	18	22.22%	Deleterious	PASSENGER/OTHER	Passenger	passenger	.	loh	0.94	0.58979	32%	Clonal	.	.	.	.	WES		
IDC15T	*LPCAT2*	p.A525T	Missense_Mutation	16	55616948	G	A	21	19.05%	Neutral	PASSENGER/OTHER	Passenger	passenger	.	loh	0.61	0.35598	24%	Subclonal	.	.	.	.	WES		
IDC15T	*MAP3K1*	p.P1019L	Missense_Mutation	5	56178083	C	T	17	17.65%	Deleterious	PASSENGER/OTHER	Passenger	passenger	.	loh	0.39	0.0966	14%	Subclonal	.	TRUE	TRUE	YES	WES		
IDC15T	*MAP3K10*	p.P727L	Missense_Mutation	19	40719766	C	T	18	22.22%	Neutral	PASSENGER/OTHER	Passenger	passenger	.	.	1	0.74035	39%	Clonal	.	.	.	.	WES		
IDC15T	*MDN1*	p.R878C	Missense_Mutation	6	90468044	G	A	16	18.75%	Deleterious	PASSENGER/OTHER	Passenger	passenger	.	.	0.79	0.51095	24%	Clonal	.	.	.	.	WES		
IDC15T	*MKNK2*	p.P373L	Missense_Mutation	19	2040169	G	A	36	11.11%	Deleterious	PASSENGER/OTHER	Passenger	passenger	.	.	0.47	0.17	18%	Subclonal	.	.	.	.	WES		
IDC15T	*MTMR4*	p.S383L	Missense_Mutation	17	56582291	G	A	37	10.81%	Deleterious	PASSENGER/OTHER	Passenger	passenger	.	loh	0.35	0.02287	14%	Subclonal	.	.	.	.	WES		
IDC15T	*MUM1*	p.P350A	Missense_Mutation	19	1360965	C	G	32	40.63%	Neutral	PASSENGER/OTHER	Passenger	passenger	.	.	1	0.89391	68%	Clonal	.	.	.	.	WES		
IDC15T	*MYADM*	p.A140T	Missense_Mutation	19	54377201	G	A	144	4.17%	Deleterious	PASSENGER/OTHER	Passenger	passenger	.	.	0.13	0	6%	Subclonal	.	.	.	.	WES		
IDC15T	*MYOCD*	p.G721R	Missense_Mutation	17	12659833	G	A	44	9.09%	Neutral	PASSENGER/OTHER	Passenger	passenger	.	loh	0.56	0.31737	22%	Subclonal	.	.	TRUE	.	WES		
IDC15T	*NCAM2*	p.P804L	Missense_Mutation	21	22910175	C	T	5	60.00%	Deleterious	PASSENGER/OTHER	Passenger	passenger	.	.	1	0.61805	38%	Clonal	.	.	.	.	WES		
IDC15T	*NFIC*	p.R185C	Missense_Mutation	19	3382232	C	T	85	49.41%	Deleterious	PASSENGER/OTHER	Passenger	passenger	.	.	1	0.33112	91%	Clonal	.	.	.	.	WES		
IDC15T	*NKTR*	p.G174R	Missense_Mutation	3	42672778	G	A	34	29.41%	Deleterious	PASSENGER/OTHER	Passenger	passenger	.	loh	1	0.76712	56%	Clonal	.	.	.	.	WES		
IDC15T	*NOX1*	p.N122K	Missense_Mutation	X	100117781	G	T	16	62.50%	Deleterious	PASSENGER/OTHER	Passenger	passenger	.	loh	1	0.92153	67%	Clonal	.	.	.	.	WES		
IDC15T	*NTN1*	p.D98V	Missense_Mutation	17	8925983	A	T	30	56.67%	Deleterious	PASSENGER/OTHER	Driver	likely_pathogenic	.	loh	1	0.88716	78%	Clonal	.	.	.	.	WES		
IDC15T	*PARP11*	p.R246C	Missense_Mutation	12	3921570	G	A	33	12.12%	Deleterious	PASSENGER/OTHER	Passenger	passenger	.	loh	0.75	0.49174	27%	Subclonal	.	.	.	.	WES		
IDC15T	*PARP3*	p.R137Q	Missense_Mutation	3	51978482	G	A	73	6.85%	Neutral	PASSENGER/OTHER	Passenger	passenger	.	loh	0.29	0.00138	12%	Subclonal	.	.	.	.	WES		
IDC15T	*PCLO*	p.T2908I	Missense_Mutation	7	82581546	G	A	47	8.51%	Deleterious	PASSENGER/OTHER	Passenger	passenger	.	.	0.44	0.14334	17%	Subclonal	.	.	.	.	WES		
IDC15T	*PEX11B*	p.V211M	Missense_Mutation	1	145522770	G	A	120	5.83%	Deleterious	PASSENGER/OTHER	Passenger	passenger	.	.	0.6	0.28233	29%	Subclonal	.	.	.	.	WES		
IDC15T	*PHACTR1*	p.R156S	Missense_Mutation	6	12934082	G	C	42	16.67%	Neutral	.	Passenger	passenger	.	loh	0.7	0.42579	34%	Subclonal	.	.	.	.	WES		
IDC15T	*PKDCC*	p.E373Q	Missense_Mutation	2	42282428	G	C	12	25.00%	Deleterious	.	Passenger	passenger	.	loh	1	0.61065	28%	Clonal	.	.	.	.	WES		
IDC15T	*PLEKHF1*	p.G240V	Missense_Mutation	19	30165465	G	T	96	8.33%	Neutral	PASSENGER/OTHER	Passenger	passenger	.	.	0.68	0.3978	34%	Subclonal	.	.	.	.	WES		
IDC15T	*POLR1A*	p.D40H	Missense_Mutation	2	86327255	C	G	36	30.56%	Deleterious	PASSENGER/OTHER	Passenger	passenger	.	loh	1	0.78624	59%	Clonal	.	.	.	.	WES		
IDC15T	*PPP1R3A*	p.I365L	Missense_Mutation	7	113520054	T	G	38	28.95%	Neutral	PASSENGER/OTHER	Passenger	passenger	.	.	1	0.77163	58%	Clonal	.	.	.	.	WES		
IDC15T	*PRRC2C*	p.L60F	Missense_Mutation	1	171482205	C	T	30	13.33%	Deleterious	PASSENGER/OTHER	Passenger	passenger	.	.	1	0.69512	36%	Clonal	.	.	.	.	WES		
IDC15T	*PTCD1*	p.P220S	Missense_Mutation	7	99030984	G	A	14	21.43%	Deleterious	PASSENGER/OTHER	Driver	likely_pathogenic	.	.	0.9	0.56077	26%	Clonal	.	.	.	.	WES		
IDC15T	*PTPRK*	p.A155P	Missense_Mutation	6	128643216	C	G	34	35.29%	Deleterious	PASSENGER/OTHER	Passenger	passenger	.	.	1	0.89644	69%	Clonal	TRUE	.	.	.	WES		
IDC15T	*PUF60*	p.S396L	Missense_Mutation	8	144899273	G	A	48	8.33%	Neutral	PASSENGER/OTHER	Passenger	passenger	.	.	0.43	0.13038	17%	Subclonal	.	.	.	.	WES		
IDC15T	*PXN*	p.R21W	Missense_Mutation	12	120662133	G	A	35	11.43%	Deleterious	PASSENGER/OTHER	Passenger	passenger	.	loh	0.37	0.03581	14%	Subclonal	.	.	.	.	WES		
IDC15T	*RBBP6*	p.E461K	Missense_Mutation	16	24574611	G	A	25	24.00%	Deleterious	PASSENGER/OTHER	Passenger	passenger	.	loh	0.77	0.50598	35%	Clonal	.	.	.	.	WES		
IDC15T	*RELB*	p.P286L	Missense_Mutation	19	45528966	C	T	36	33.33%	Deleterious	PASSENGER/OTHER	Passenger	passenger	.	.	1	0.74701	57%	Clonal	.	.	.	.	WES		
IDC15T	*RENBP*	p.R106Q	Missense_Mutation	X	153209143	C	T	43	9.30%	Deleterious	PASSENGER/OTHER	Passenger	passenger	.	loh	0.3	0.00537	12%	Subclonal	.	.	.	.	WES		
IDC15T	*RGMA*	p.Y107C	Missense_Mutation	15	93595572	T	C	26	57.69%	Deleterious	PASSENGER/OTHER	Passenger	passenger	.	loh	1	0.8699	69%	Clonal	.	.	.	.	WES		
IDC15T	*RP11-849H4.2*	p.T169M	Missense_Mutation	11	71627229	G	A	13	30.77%	.	PASSENGER/OTHER	Passenger	passenger	.	loh	0.99	0.61314	33%	Clonal	.	.	.	.	WES		
IDC15T	*RP1L1*	p.S951L	Missense_Mutation	8	10468756	G	A	49	8.16%	Neutral	PASSENGER/OTHER	Passenger	passenger	.	loh	0.26	0.00112	10%	Subclonal	.	.	.	.	WES		
IDC15T	*RPGRIP1L*	p.S1195N	Missense_Mutation	16	53652969	C	T	13	23.08%	Neutral	PASSENGER/OTHER	Passenger	passenger	.	loh	0.74	0.48275	24%	Subclonal	.	.	.	.	WES		
IDC15T	*SCML2*	p.G697R	Missense_Mutation	X	18259385	C	T	20	15.00%	Deleterious	PASSENGER/OTHER	Passenger	passenger	.	loh	0.48	0.23055	17%	Subclonal	.	.	.	.	WES		
IDC15T	*SCN4A*	p.K566E	Missense_Mutation	17	62038702	T	C	77	31.17%	Neutral	PASSENGER/OTHER	Passenger	passenger	.	loh	1	0.84637	74%	Clonal	.	.	.	.	WES		
IDC15T	*SELE*	p.Y608C	Missense_Mutation	1	169694895	T	C	45	17.78%	Neutral	PASSENGER/OTHER	Passenger	passenger	.	.	1	0.84222	56%	Clonal	.	.	.	.	WES		
IDC15T	*SEPHS2*	p.I317M	Missense_Mutation	16	30456098	G	C	9	33.33%	Deleterious	PASSENGER/OTHER	Driver	likely_pathogenic	.	loh	1	0.6178	29%	Clonal	.	.	.	.	WES		
IDC15T	*SHANK3*	p.G1156R	Missense_Mutation	22	51159679	G	A	43	9.30%	Neutral	PASSENGER/OTHER	Passenger	passenger	.	loh	0.21	0.00001	8%	Subclonal	.	.	.	.	WES		
IDC15T	*SIK1*	p.T485S	Missense_Mutation	21	44838909	G	C	28	14.29%	Neutral	PASSENGER/OTHER	Passenger	passenger	.	.	1	0.61749	32%	Clonal	.	.	.	.	WES		
IDC15T	*SLC16A8*	p.L386F	Missense_Mutation	22	38476889	G	A	41	53.66%	Deleterious	PASSENGER/OTHER	Passenger	passenger	.	loh	1	0.91608	81%	Clonal	.	.	.	.	WES		
IDC15T	*SLC22A23*	p.R425Q	Missense_Mutation	6	3290037	C	T	47	8.51%	Deleterious	PASSENGER/OTHER	Passenger	passenger	.	loh	0.36	0.0379	14%	Subclonal	.	.	.	.	WES		
IDC15T	*SLC22A3*	p.G235D	Missense_Mutation	6	160829800	G	A	40	12.50%	Deleterious	PASSENGER/OTHER	Passenger	passenger	.	.	0.65	0.39373	27%	Subclonal	.	.	.	.	WES		
IDC15T	*SLC45A1*	p.E550K	Missense_Mutation	1	8397926	G	A	43	9.30%	Deleterious	PASSENGER/OTHER	Passenger	passenger	.	.	0.39	0.06919	15%	Subclonal	.	.	.	.	WES		
IDC15T	*STAB2*	p.Y1172F	Missense_Mutation	12	104089555	A	T	21	42.86%	Deleterious	PASSENGER/OTHER	Passenger	passenger	.	loh	1	0.79284	58%	Clonal	.	.	.	.	WES		
IDC15T	*STXBP5*	p.S943L	Missense_Mutation	6	147684753	C	T	35	11.43%	Deleterious	PASSENGER/OTHER	Passenger	passenger	.	.	0.71	0.4611	26%	Subclonal	.	.	.	.	WES		
IDC15T	*STXBP6*	p.D101Y	Missense_Mutation	14	25325292	C	A	11	63.64%	Deleterious	.	Passenger	passenger	.	loh	1	0.89576	58%	Clonal	.	.	.	.	WES		
IDC15T	*SUGP2*	p.G239V	Missense_Mutation	19	19136441	C	A	108	54.63%	Neutral	PASSENGER/OTHER	Passenger	passenger	.	.	1	0.25692	93%	Clonal	.	.	.	.	WES		
IDC15T	*SUMO3*	p.G9D	Missense_Mutation	21	46234015	C	T	64	9.38%	Deleterious	PASSENGER/OTHER	Passenger	passenger	.	.	0.68	0.41151	30%	Subclonal	.	.	.	.	WES		
IDC15T	*SVIL*	p.G1741R	Missense_Mutation	10	29769622	C	T	89	6.74%	Deleterious	PASSENGER/OTHER	Passenger	passenger	.	.	0.35	0.00721	16%	Subclonal	.	.	.	.	WES		
IDC15T	*TCEAL4*	p.I209T	Missense_Mutation	X	102842229	T	C	26	11.54%	Neutral	PASSENGER/OTHER	Passenger	passenger	.	loh	0.37	0.08143	13%	Subclonal	.	.	.	.	WES		
IDC15T	*TMEM206*	p.N125K	Missense_Mutation	1	212560384	G	C	17	41.18%	Deleterious	.	Passenger	passenger	.	.	1	0.84358	55%	Clonal	.	.	.	.	WES		
IDC15T	*TRIM47*	p.V378L	Missense_Mutation	17	73872051	C	G	76	13.16%	Neutral	PASSENGER/OTHER	Passenger	passenger	.	loh	0.55	0.10705	30%	Subclonal	.	.	.	.	WES		
IDC15T	*TRPC7*	p.I555N	Missense_Mutation	5	135583339	A	T	16	75.00%	Deleterious	PASSENGER/OTHER	Passenger	passenger	.	loh	1	0.54161	73%	Clonal	.	.	.	.	WES		
IDC15T	*TRPV2*	p.L625M	Missense_Mutation	17	16335498	C	A	67	10.45%	Deleterious	PASSENGER/OTHER	Passenger	passenger	.	loh	0.65	0.35802	31%	Subclonal	.	.	.	.	WES		
IDC15T	*TRPV4*	p.I473M	Missense_Mutation	12	110232206	G	C	32	31.25%	Deleterious	PASSENGER/OTHER	Passenger	passenger	.	loh	1	0.69992	51%	Clonal	.	.	.	.	WES		
IDC15T	*TYRP1*	p.R55C	Missense_Mutation	9	12694159	C	T	71	50.70%	Deleterious	PASSENGER/OTHER	Passenger	passenger	.	.	1	0.3136	89%	Subclonal	.	.	.	.	WES		
IDC15T	*UBA7*	p.R501Q	Missense_Mutation	3	49847827	C	T	25	12.00%	Neutral	PASSENGER/OTHER	Passenger	passenger	.	loh	0.51	0.27552	17%	Subclonal	.	.	.	.	WES		
IDC15T	*UBN1*	p.K189N	Missense_Mutation	16	4909160	G	T	21	19.05%	Deleterious	PASSENGER/OTHER	Passenger	passenger	.	loh	0.8	0.52451	29%	Clonal	.	.	.	.	WES		
IDC15T	*VAV1*	p.E288D	Missense_Mutation	19	6826659	G	T	62	11.29%	Deleterious	PASSENGER/OTHER	Driver	likely_pathogenic	.	.	0.93	0.61297	40%	Clonal	.	.	.	.	WES		
IDC15T	*VPS13D*	p.R2272T	Missense_Mutation	1	12371675	G	C	8	62.50%	Deleterious	PASSENGER/OTHER	Passenger	passenger	.	loh	1	0.85536	49%	Clonal	.	.	.	.	WES		
IDC15T	*VPS13D*	p.A4140T	Missense_Mutation	1	12516138	G	A	37	10.81%	Deleterious	PASSENGER/OTHER	Passenger	passenger	.	loh	0.35	0.02287	14%	Subclonal	.	.	.	.	WES		
IDC15T	*WDR86*	p.W42R	Missense_Mutation	7	151106552	A	T	31	29.03%	Deleterious	CANCER	Passenger	likely_pathogenic	.	.	1	0.81735	57%	Clonal	.	.	.	.	WES		
IDC15T	*YTHDF1*	p.R404H	Missense_Mutation	20	61834081	C	T	101	5.94%	Deleterious	PASSENGER/OTHER	Passenger	passenger	.	.	0.43	0.05762	20%	Subclonal	.	.	.	.	WES		
IDC15T	*ZBTB37*	p.L416V	Missense_Mutation	1	173854996	C	G	161	9.94%	Deleterious	PASSENGER/OTHER	Passenger	passenger	.	.	1	0.72226	57%	Clonal	.	.	.	.	WES		
IDC15T	*ZFR*	p.T472I	Missense_Mutation	5	32403312	G	A	22	18.18%	Deleterious	PASSENGER/OTHER	Passenger	passenger	.	loh	0.4	0.05622	16%	Subclonal	.	.	.	.	WES		
IDC15T	*ZMYM4*	p.R1175Q	Missense_Mutation	1	35865173	G	A	38	10.53%	Deleterious	PASSENGER/OTHER	Passenger	passenger	.	.	0.44	0.13452	17%	Subclonal	.	.	.	.	WES		
IDC15T	*ZNF516*	p.G893S	Missense_Mutation	18	74091393	C	T	18	16.67%	Neutral	PASSENGER/OTHER	Passenger	passenger	.	loh	0.37	0.06884	13%	Subclonal	.	.	.	.	WES		
IDC15T	*ZNF70*	p.A141V	Missense_Mutation	22	24086906	G	A	47	8.51%	Neutral	PASSENGER/OTHER	Passenger	passenger	.	loh	0.27	0.00191	11%	Subclonal	.	.	.	.	WES		
IDC15T	*ZNF835*	p.T439M	Missense_Mutation	19	57175251	G	A	48	8.33%	Neutral	PASSENGER/OTHER	Passenger	passenger	.	.	0.27	0.00147	10%	Subclonal	.	.	.	.	WES		
IDC15T	*ZNF865*	p.R1005C	Missense_Mutation	19	56127997	C	T	54	7.41%	.	PASSENGER/OTHER	Passenger	passenger	.	.	0.24	0.00029	9%	Subclonal	.	.	.	.	WES		
IDC15T	*CATSPERG*	p.K1064*	Nonsense_Mutation	19	38860875	A	T	36	13.89%	Neutral	.	.	passenger	.	.	1	0.62329	36%	Clonal	.	.	.	.	WES		
IDC15T	*EFTUD1*	p.R998*	Nonsense_Mutation	15	82431181	G	A	44	11.36%	Deleterious	.	.	likely_pathogenic	.	loh	0.48	0.13894	21%	Subclonal	.	.	.	.	WES		
IDC15T	*MTA1*	p.Q235*	Nonsense_Mutation	14	105926744	C	T	126	5.56%	Deleterious	.	.	passenger	.	.	0.29	0.00013	14%	Subclonal	.	.	.	.	WES		
IDC15T	*MUM1*	p.S290*	Nonsense_Mutation	19	1360786	C	A	53	33.96%	Deleterious	.	.	passenger	.	.	1	0.83921	71%	Clonal	.	.	.	.	WES		
IDC15T	*MUM1*	p.S162*	Nonsense_Mutation	19	1360402	C	G	77	18.18%	Deleterious	.	.	passenger	.	.	0.77	0.46689	46%	Subclonal	.	.	.	.	WES		
IDC15T	*PEX13*	p.R170*	Nonsense_Mutation	2	61258969	C	T	36	11.11%	Deleterious	.	.	likely_pathogenic	.	loh	0.47	0.17	18%	Subclonal	.	.	.	.	WES		
IDC15T	*PODXL*	p.Q297*	Nonsense_Mutation	7	131194258	G	A	43	9.30%	Deleterious	.	.	passenger	.	.	0.39	0.06919	15%	Subclonal	.	.	.	.	WES		
IDC15T	*PTPN21*	p.R808*	Nonsense_Mutation	14	88945353	G	A	46	8.70%	Deleterious	.	.	passenger	.	loh	0.28	0.00248	11%	Subclonal	.	.	.	.	WES		
IDC15T	*RNF149*	p.R304*	Nonsense_Mutation	2	101902593	G	A	16	18.75%	Deleterious	.	.	likely_pathogenic	.	loh	0.79	0.51095	24%	Clonal	.	.	.	.	WES		
IDC15T	*TTLL7*	p.R352*	Nonsense_Mutation	1	84394907	G	A	17	17.65%	Deleterious	.	.	passenger	.	.	0.74	0.48559	23%	Subclonal	.	.	.	.	WES		
IDC15T	*CA9*	p.X303_splice	Splice_Site	9	35677854	G	A	28	35.71%	Deleterious	.	.	passenger	.	loh	1	0.75261	55%	Clonal	.	.	.	.	WES		
IDC15T	*MAML1*	p.X658_splice	Splice_Site	5	179198145	TAGG	T	41	48.78%			.	likely_pathogenic	.	loh	1	0.93601	79%	Clonal	.	.	.	.	WES		
IDC19T	*IRS2*	p.S499C	Missense_Mutation	13	110436905	G	C	161	26.09%	Deleterious	PASSENGER/OTHER	Passenger	passenger	.	.	1	0.84035	75%	Clonal	.	.	.	YES	MSK-IMPACT		
IDC37T	*PIK3CA*	p.C420R	Missense_Mutation	3	178927980	T	C	96	13.54%	Deleterious	PASSENGER/OTHER	Driver	likely_pathogenic	TRUE	.	0.64	0.05594	38%	Clonal	TRUE	TRUE	TRUE	YES	WES	TGTTGAATTTTCCTTTTGGGGAAGA	GGGTTCAGCAAATCTTCTAATCCA
IDC19T	*PIK3CB*	p.Y622H	Missense_Mutation	3	138413656	A	G	63	23.81%	Deleterious	CANCER	Passenger	likely_pathogenic	.	.	0.96	0.69569	56%	Clonal	.	.	.	YES	MSK-IMPACT		
IDC19T	*ASXL2*	p.E347*	Nonsense_Mutation	2	25976506	C	A	73	17.81%	Deleterious	.	.	passenger	.	.	0.72	0.37512	42%	Subclonal	.	.	.	YES	MSK-IMPACT		
IDC24T	*BSN*	p.P3389Lfs*96	Frame_Shift_Del	3	49699440	TACCCCCC	T	151	5.30%	.	.	.	likely_pathogenic	.	loh	0.25	0	12%	Subclonal	.	.	.	.	WES		
IDC24T	*ANKRD54*	p.D278N	Missense_Mutation	22	38228021	C	T	50	32.00%	Deleterious	CANCER	Passenger	likely_pathogenic	.	loh	1	0.85922	64%	Clonal	.	.	.	.	WES		
IDC24T	*DBX2*	p.R116K	Missense_Mutation	12	45444364	C	T	149	18.79%	Neutral	PASSENGER/OTHER	Passenger	passenger	.	.	0.88	0.66137	60%	Clonal	.	.	.	.	WES		
IDC24T	*DNALI1*	p.R176Q	Missense_Mutation	1	38027221	G	A	56	8.93%	Deleterious	CANCER	Passenger	likely_pathogenic	.	loh	0.33	0.00815	14%	Subclonal	.	.	.	.	WES		
IDC24T	*DRD5*	p.G110E	Missense_Mutation	4	9783982	G	A	14	21.43%	Deleterious	PASSENGER/OTHER	Passenger	passenger	.	loh	0.78	0.60702	24%	Clonal	.	.	.	.	WES		
IDC24T	*FBXL16*	p.V127M	Missense_Mutation	16	747027	C	T	214	16.36%	Deleterious	PASSENGER/OTHER	Passenger	passenger	.	.	0.93	0.73792	66%	Clonal	.	.	.	.	WES		
IDC24T	*FUK*	p.V113E	Missense_Mutation	16	70500087	T	A	115	23.48%	Neutral	PASSENGER/OTHER	Passenger	passenger	.	loh	0.86	0.70021	59%	Clonal	.	.	.	.	WES		
IDC24T	*GUK1*	p.G54D	Missense_Mutation	1	228333311	G	A	98	25.51%	Deleterious	PASSENGER/OTHER	Passenger	passenger	.	.	1	0.94186	80%	Clonal	.	.	.	.	WES		
IDC24T	*HIST2H3D*	p.E95K	Missense_Mutation	1	149784954	C	T	80	27.50%	Deleterious	PASSENGER/OTHER	Passenger	passenger	.	.	1	0.85183	72%	Clonal	.	.	.	YES	WES		
IDC24T	*HSBP1L1*	p.K68E	Missense_Mutation	18	77728172	A	G	66	13.64%	Neutral	.	Passenger	passenger	.	.	0.77	0.53063	40%	Clonal	.	.	.	.	WES		
IDC24T	*IFT81*	p.Q363K	Missense_Mutation	12	110600769	C	A	121	18.18%	Deleterious	PASSENGER/OTHER	Passenger	passenger	.	.	0.85	0.61602	56%	Clonal	.	.	.	.	WES		
IDC24T	*IL18*	p.D146H	Missense_Mutation	11	112014465	C	G	81	4.94%	Neutral	PASSENGER/OTHER	Passenger	passenger	.	loh	0.18	0	7%	Subclonal	.	.	.	.	WES		
IDC24T	*IL23R*	p.K43N	Missense_Mutation	1	67635083	G	C	44	25.00%	Deleterious	PASSENGER/OTHER	Passenger	passenger	.	loh	1	0.77508	56%	Clonal	.	.	.	.	WES		
IDC24T	*ISM2*	p.D413N	Missense_Mutation	14	77942417	C	T	161	11.80%	Deleterious	PASSENGER/OTHER	Passenger	passenger	.	.	0.67	0.21081	43%	Subclonal	.	.	.	.	WES		
IDC24T	*KIAA1244*	p.R757Q	Missense_Mutation	6	138599729	G	A	84	5.95%	Deleterious	PASSENGER/OTHER	Passenger	passenger	.	loh	0.28	0.00102	12%	Subclonal	.	.	.	.	WES		
IDC24T	*KLC4*	p.E246A	Missense_Mutation	6	43034155	A	C	202	20.30%	Deleterious	PASSENGER/OTHER	Driver	likely_pathogenic	.	.	0.95	0.77206	69%	Clonal	.	.	.	.	WES		
IDC24T	*KRT2*	p.L435F	Missense_Mutation	12	53040690	G	A	210	20.95%	Neutral	PASSENGER/OTHER	Passenger	passenger	.	.	0.98	0.80782	71%	Clonal	.	.	.	.	WES		
IDC24T	*LLGL1*	p.S250T	Missense_Mutation	17	18137621	G	C	149	16.78%	Neutral	PASSENGER/OTHER	Passenger	passenger	.	loh	0.61	0.03639	42%	Subclonal	.	.	.	.	WES		
IDC24T	*LRP1*	p.G2084C	Missense_Mutation	12	57578685	G	T	113	18.58%	Deleterious	PASSENGER/OTHER	Passenger	passenger	.	.	0.87	0.64545	56%	Clonal	.	.	.	.	WES		
IDC24T	*LRRC1*	p.R62Q	Missense_Mutation	6	53706933	G	A	39	10.26%	Deleterious	PASSENGER/OTHER	Passenger	passenger	.	.	0.48	0.2064	19%	Subclonal	.	.	.	.	WES		
IDC24T	*MATN4*	p.P290S	Missense_Mutation	20	43929763	G	A	158	21.52%	Neutral	PASSENGER/OTHER	Passenger	passenger	.	.	1	0.81121	69%	Clonal	.	.	.	.	WES		
IDC24T	*MIIP*	p.I289F	Missense_Mutation	1	12090104	A	T	256	28.52%	Neutral	PASSENGER/OTHER	Passenger	passenger	.	loh	1	0.91847	81%	Clonal	.	.	.	.	WES		
IDC24T	*MYO9A*	p.T493S	Missense_Mutation	15	72286920	T	A	118	14.41%	Deleterious	PASSENGER/OTHER	Passenger	passenger	.	.	0.67	0.23922	42%	Subclonal	.	.	.	.	WES		
IDC24T	*NAT2*	p.S127I	Missense_Mutation	8	18257893	G	T	117	38.46%	Deleterious	PASSENGER/OTHER	Passenger	passenger	.	loh	1	0.94311	88%	Clonal	.	.	.	.	WES		
IDC24T	*NKD1*	p.R15M	Missense_Mutation	16	50582646	G	T	48	12.50%	Deleterious	PASSENGER/OTHER	Passenger	passenger	.	.	0.58	0.29296	27%	Subclonal	.	.	.	.	WES		
IDC24T	*NTN5*	p.P112T	Missense_Mutation	19	49173910	G	T	136	25.00%	Neutral	PASSENGER/OTHER	Passenger	passenger	.	.	1	0.87195	75%	Clonal	.	.	.	.	WES		
IDC24T	*OR10C1*	p.L143P	Missense_Mutation	6	29408220	T	C	181	20.99%	Deleterious	PASSENGER/OTHER	Passenger	passenger	.	.	0.98	0.80032	70%	Clonal	.	.	.	.	WES		
IDC24T	*OR6V1*	p.G94A	Missense_Mutation	7	142749718	G	C	124	22.58%	Neutral	PASSENGER/OTHER	Passenger	passenger	.	.	1	0.82343	69%	Clonal	.	.	.	.	WES		
IDC24T	*PDE6B*	p.R676S	Missense_Mutation	4	657909	A	C	43	30.23%	Deleterious	PASSENGER/OTHER	Passenger	passenger	.	loh	1	0.82956	59%	Clonal	.	.	.	.	WES		
IDC24T	*PLEKHA6*	p.D936N	Missense_Mutation	1	204197935	C	T	210	7.62%	Deleterious	PASSENGER/OTHER	Passenger	passenger	.	.	0.58	0.08582	36%	Subclonal	.	.	.	.	WES		
IDC24T	*PLSCR5*	p.D228H	Missense_Mutation	3	146307535	C	G	44	9.09%	Neutral	PASSENGER/OTHER	Passenger	passenger	.	.	0.42	0.12311	17%	Subclonal	.	.	.	.	WES		
IDC24T	*PTPRN2*	p.P218L	Missense_Mutation	7	157959880	G	A	198	18.69%	Neutral	PASSENGER/OTHER	Passenger	passenger	.	.	0.87	0.6479	63%	Clonal	.	.	.	.	WES		
IDC24T	*RPAP2*	p.A334G	Missense_Mutation	1	92789478	C	G	74	9.46%	Deleterious	.	Passenger	passenger	.	loh	0.44	0.04235	21%	Subclonal	.	.	.	.	WES		
IDC24T	*SEC63*	p.T537A	Missense_Mutation	6	108214751	T	C	40	7.50%	Neutral	PASSENGER/OTHER	Passenger	passenger	.	loh	0.35	0.08155	12%	Subclonal	.	.	.	.	WES		
IDC24T	*SEZ6L*	p.T381P	Missense_Mutation	22	26693025	A	C	120	16.67%	Deleterious	PASSENGER/OTHER	Passenger	passenger	.	.	0.78	0.47979	51%	Subclonal	.	.	.	.	WES		
IDC24T	*SLC44A5*	p.D495E	Missense_Mutation	1	75684219	G	C	66	16.67%	Deleterious	PASSENGER/OTHER	Passenger	passenger	.	loh	0.78	0.52885	43%	Clonal	.	.	.	.	WES		
IDC24T	*SPACA1*	p.V76I	Missense_Mutation	6	88763681	G	A	29	34.48%	Neutral	PASSENGER/OTHER	Passenger	passenger	.	loh	1	0.86686	61%	Clonal	.	.	.	.	WES		
IDC24T	*SREBF1*	p.R616H	Missense_Mutation	17	17720300	C	T	67	22.39%	Deleterious	PASSENGER/OTHER	Passenger	passenger	.	loh	0.82	0.65148	50%	Clonal	.	.	.	.	WES		
IDC24T	*TEP1*	p.L2624V	Missense_Mutation	14	20836610	G	C	72	23.61%	Deleterious	PASSENGER/OTHER	Passenger	passenger	.	.	1	0.79791	63%	Clonal	.	.	.	.	WES		
IDC24T	*TROAP*	p.R239W	Missense_Mutation	12	49719940	C	T	93	19.35%	Deleterious	.	Passenger	passenger	.	.	0.9	0.68479	56%	Clonal	.	.	.	.	WES		
IDC24T	*TRPM3*	p.L922V	Missense_Mutation	9	73213583	G	C	18	22.22%	Deleterious	PASSENGER/OTHER	Passenger	passenger	.	.	1	0.65102	33%	Clonal	.	.	.	.	WES		
IDC24T	*TUBAL3*	p.R327I	Missense_Mutation	10	5435841	C	A	164	13.41%	Deleterious	PASSENGER/OTHER	Passenger	passenger	.	.	0.62	0.06966	42%	Subclonal	.	.	.	.	WES		
IDC24T	*UNC13A*	p.I1042V	Missense_Mutation	19	17746924	T	C	30	13.33%	Deleterious	PASSENGER/OTHER	Passenger	passenger	.	.	0.62	0.40426	24%	Subclonal	.	.	.	.	WES		
IDC24T	*UQCRC1*	p.T35M	Missense_Mutation	3	48646701	G	A	75	6.67%	Neutral	PASSENGER/OTHER	Passenger	passenger	.	loh	0.31	0.00448	13%	Subclonal	.	.	.	.	WES		
IDC24T	*USP53*	p.H766R	Missense_Mutation	4	120212365	A	G	87	14.94%	Neutral	PASSENGER/OTHER	Passenger	passenger	.	.	0.7	0.36399	41%	Subclonal	.	.	.	.	WES		

IDC24T	*VEGFB*	p.G62D	Missense_Mutation	11	64003366	G	A	104	5.77%	Deleterious	.	Passenger	passenger	.	.	0.27	0.00014	12%	Subclonal	.	.	.	.	WES		
IDC24T	*ZFYVE28*	p.R143H	Missense_Mutation	4	2341273	C	T	229	33.62%	Deleterious	PASSENGER/OTHER	Passenger	passenger	.	loh	1	0.91904	87%	Clonal	.	.	.	.	WES		
IDC24T	*BRAT1*	p.R177*	Nonsense_Mutation	7	2583498	G	A	247	16.60%	Deleterious	.	.	passenger	.	.	0.77	0.31508	58%	Subclonal	.	.	.	.	WES		
IDC24T	*KIF24*	p.R1296*	Nonsense_Mutation	9	34255150	G	A	73	6.85%	Deleterious	.	.	passenger	.	.	0.32	0.00615	14%	Subclonal	.	.	.	.	WES		
IDC24T	*MAL*	p.Y91*	Nonsense_Mutation	2	95715337	C	A	74	29.73%	Deleterious	.	.	passenger	.	.	1	0.85315	74%	Clonal	.	.	.	.	WES		
IDC24T	*MAST4*	p.S38*	Nonsense_Mutation	5	65892596	C	A	143	5.59%	Deleterious	.	.	likely_pathogenic	.	loh	0.2	0	10%	Subclonal	.	.	.	.	WES		
IDC24T	*PALB2*	p.Q479*	Nonsense_Mutation	16	23646432	G	A	85	20.00%	Deleterious	.	.	likely_pathogenic	.	.	0.93	0.7093	56%	Clonal	TRUE	.	.	YES	WES	GCAGGCTGATTTTCTTTTTCCTG	TCCAATGAGGAAACTGACCAA
IDC24T	*PI16*	p.Q73*	Nonsense_Mutation	6	36926966	C	T	213	25.82%	Deleterious	.	.	passenger	.	.	1	0.90118	83%	Clonal	.	.	.	.	WES		
IDC24T	*ANKDD1B*	p.X65_splice	Splice_Site	5	74912628	G	C	49	22.45%	.	.	.	likely_pathogenic	.	loh	0.82	0.65813	45%	Clonal	.	.	.	.	WES		
IDC28T	*SCN2A*	p.A841Qfs*27	Frame_Shift_Del	2	166198936	TG	T	86	29.07%	.	.	.	passenger	.	.	1	0.66933	71%	Clonal	.	.	.	.	WES		
IDC28T	*ASH1L*	p.E1654K	Missense_Mutation	1	155447701	C	T	31	12.90%	Deleterious	PASSENGER/OTHER	Passenger	passenger	.	.	0.64	0.20551	24%	Subclonal	.	.	.	.	WES		
IDC28T	*ATP2B2*	p.E696K	Missense_Mutation	3	10400425	C	T	65	9.23%	Deleterious	PASSENGER/OTHER	Passenger	passenger	.	.	0.37	0.00359	17%	Subclonal	.	.	.	.	WES		
IDC28T	*C1orf172*	p.S276L	Missense_Mutation	1	27278045	G	A	186	4.84%	Deleterious	PASSENGER/OTHER	Passenger	passenger	.	.	0.19	0	10%	Subclonal	.	.	.	.	WES		
IDC28T	*C5orf22*	p.D172N	Missense_Mutation	5	31538503	G	A	44	27.27%	Neutral	PASSENGER/OTHER	Passenger	passenger	.	.	1	0.5316	56%	Clonal	.	.	.	.	WES		
IDC28T	*CCND1*	p.E252K	Missense_Mutation	11	69465916	G	A	199	4.52%	Deleterious	PASSENGER/OTHER	Passenger	passenger	.	loh	0.18	0	9%	Subclonal	TRUE	TRUE	TRUE	YES	WES		
IDC28T	*CDHR3*	p.P401A	Missense_Mutation	7	105653454	C	G	30	23.33%	Deleterious	PASSENGER/OTHER	Passenger	passenger	.	.	0.93	0.3921	42%	Subclonal	.	.	.	.	WES		
IDC28T	*CNGA4*	p.T226M	Missense_Mutation	11	6261701	C	T	194	7.73%	Deleterious	PASSENGER/OTHER	Passenger	passenger	.	.	0.31	0	18%	Subclonal	.	.	.	.	WES		
IDC28T	*CNGB3*	p.R355Q	Missense_Mutation	8	87656093	C	T	141	4.26%	Deleterious	PASSENGER/OTHER	Passenger	passenger	.	.	0.34	0.00251	15%	Subclonal	.	.	.	.	WES		
IDC28T	*COPA*	p.R1183Q	Missense_Mutation	1	160260376	C	T	52	9.62%	Deleterious	PASSENGER/OTHER	Passenger	passenger	.	.	0.48	0.06226	21%	Subclonal	.	.	.	.	WES		
IDC28T	*DHX16*	p.R736L	Missense_Mutation	6	30624478	C	A	96	9.38%	Deleterious	PASSENGER/OTHER	Passenger	passenger	.	.	0.37	0.0004	20%	Subclonal	.	.	.	.	WES		
IDC28T	*DHX57*	p.P1028L	Missense_Mutation	2	39050343	G	A	55	36.36%	Deleterious	PASSENGER/OTHER	Passenger	passenger	.	.	1	0.70408	74%	Clonal	.	.	.	.	WES		
IDC28T	*ERLEC1*	p.G379R	Missense_Mutation	2	54040119	G	A	47	8.51%	Deleterious	PASSENGER/OTHER	Passenger	passenger	.	.	0.34	0.00911	13%	Subclonal	.	.	.	.	WES		
IDC28T	*FAM13C*	p.V79I	Missense_Mutation	10	61112119	C	T	55	9.09%	Neutral	PASSENGER/OTHER	Passenger	passenger	.	.	0.36	0.00688	16%	Subclonal	.	.	.	.	WES		
IDC28T	*FBXO32*	p.R199W	Missense_Mutation	8	124525494	G	A	72	13.89%	Deleterious	PASSENGER/OTHER	Passenger	passenger	.	.	1	0.49989	52%	Subclonal	.	.	.	.	WES		
IDC28T	*GTF3C2*	p.S379L	Missense_Mutation	2	27559284	G	A	50	10.00%	Deleterious	PASSENGER/OTHER	Passenger	passenger	.	.	0.4	0.01611	17%	Subclonal	.	.	.	.	WES		
IDC28T	*GTSE1*	p.R276Q	Missense_Mutation	22	46708102	G	A	97	7.22%	Neutral	PASSENGER/OTHER	Passenger	passenger	.	.	0.29	0.00003	14%	Subclonal	.	.	.	.	WES		
IDC28T	*GUCY2D*	p.V973M	Missense_Mutation	17	7918793	G	A	46	8.70%	Deleterious	PASSENGER/OTHER	Passenger	passenger	.	.	0.35	0.01088	14%	Subclonal	.	.	.	.	WES		
IDC28T	*GUCY2D*	p.R438H	Missense_Mutation	17	7909967	G	A	121	5.79%	Neutral	PASSENGER/OTHER	Passenger	passenger	.	.	0.23	0	11%	Subclonal	.	.	.	.	WES		
IDC28T	*HCFC1*	p.T1627M	Missense_Mutation	X	153218027	G	A	67	7.46%	Deleterious	PASSENGER/OTHER	Passenger	passenger	.	.	0.3	0.00073	13%	Subclonal	.	.	.	.	WES		
IDC28T	*HMOX2*	p.E145K	Missense_Mutation	16	4557942	G	A	160	14.38%	Deleterious	PASSENGER/OTHER	Passenger	passenger	.	.	0.86	0.37642	57%	Subclonal	.	.	.	.	WES		
IDC28T	*HOXD12*	p.V223I	Missense_Mutation	2	176965342	G	A	133	4.51%	Neutral	PASSENGER/OTHER	Passenger	passenger	.	.	0.18	0	8%	Subclonal	.	.	.	.	WES		
IDC28T	*IL13RA1*	p.E292D	Missense_Mutation	X	117900540	G	T	37	21.62%	Deleterious	PASSENGER/OTHER	Passenger	passenger	.	.	0.86	0.35589	42%	Subclonal	.	.	.	.	WES		
IDC28T	*ITPKC*	p.R553Q	Missense_Mutation	19	41239253	G	A	29	13.79%	Deleterious	PASSENGER/OTHER	Passenger	passenger	.	.	0.55	0.13276	21%	Subclonal	.	.	.	.	WES		
IDC28T	*IZUMO2*	p.R171C	Missense_Mutation	19	50657969	G	A	81	6.17%	Deleterious	PASSENGER/OTHER	Passenger	passenger	.	.	0.25	0.00004	10%	Subclonal	.	.	.	.	WES		
IDC28T	*KIAA0100*	p.R1663H	Missense_Mutation	17	26948488	C	T	72	6.94%	Deleterious	PASSENGER/OTHER	Driver	likely_pathogenic	.	.	0.28	0.00027	12%	Subclonal	.	.	.	.	WES		
IDC28T	*KIAA1109*	p.V4869I	Missense_Mutation	4	123277880	G	A	34	14.71%	Deleterious	PASSENGER/OTHER	Passenger	passenger	.	.	0.59	0.14177	25%	Subclonal	.	.	.	.	WES		
IDC28T	*KMT2D*	p.R1326Q	Missense_Mutation	12	49442931	C	T	137	4.38%	Deleterious	PASSENGER/OTHER	Passenger	passenger	.	.	0.17	0	8%	Subclonal	TRUE	TRUE	TRUE	.	WES		
IDC28T	*KRTAP4-11*	p.S83R	Missense_Mutation	17	39274319	G	C	16	18.75%	Neutral	PASSENGER/OTHER	Passenger	passenger	.	.	0.75	0.26848	23%	Subclonal	.	.	.	.	WES		
IDC28T	*LATS1*	p.R660H	Missense_Mutation	6	150004246	C	T	35	14.29%	Deleterious	PASSENGER/OTHER	Driver	likely_pathogenic	.	.	0.57	0.12758	24%	Subclonal	.	.	.	YES	WES		
IDC28T	*LGR5*	p.E858K	Missense_Mutation	12	71978362	G	A	93	5.38%	Deleterious	PASSENGER/OTHER	Passenger	passenger	.	.	0.21	0	9%	Subclonal	.	.	.	.	WES		
IDC28T	*LRFN1*	p.R508Q	Missense_Mutation	19	39799066	C	T	134	4.48%	Deleterious	PASSENGER/OTHER	Passenger	passenger	.	.	0.18	0	8%	Subclonal	.	.	.	.	WES		
IDC28T	*LRP12*	p.R295C	Missense_Mutation	8	105509897	G	A	102	6.86%	Deleterious	PASSENGER/OTHER	Passenger	passenger	.	.	0.55	0.08701	26%	Subclonal	.	.	.	.	WES		
IDC28T	*MEX3C*	p.R304C	Missense_Mutation	18	48703791	G	A	74	8.11%	Deleterious	PASSENGER/OTHER	Passenger	passenger	.	.	0.32	0.00068	15%	Subclonal	.	.	.	.	WES		
IDC28T	*MYD88*	p.G214D	Missense_Mutation	3	38182017	G	A	185	25.41%	Deleterious	PASSENGER/OTHER	Passenger	passenger	.	.	1	0.65191	74%	Clonal	TRUE	.	TRUE	YES	WES		
IDC28T	*NASP*	p.A239V	Missense_Mutation	1	46073299	C	T	42	9.52%	Neutral	PASSENGER/OTHER	Passenger	passenger	.	.	0.38	0.02156	15%	Subclonal	.	.	.	.	WES		
IDC28T	*P2RY4*	p.R145C	Missense_Mutation	X	69479042	G	A	231	30.74%	Neutral	PASSENGER/OTHER	Passenger	passenger	.	.	1	0.78543	86%	Clonal	.	.	.	.	WES		
IDC28T	*PER3*	p.S247L	Missense_Mutation	1	7858683	C	T	90	6.67%	Neutral	PASSENGER/OTHER	Passenger	passenger	.	.	0.27	0.00003	12%	Subclonal	.	.	.	.	WES		
IDC28T	*PFKFB3*	p.E335K	Missense_Mutation	10	6263628	G	A	67	7.46%	Deleterious	PASSENGER/OTHER	Passenger	passenger	.	.	0.3	0.00073	13%	Subclonal	.	.	.	.	WES		
IDC19T	*PIK3CA*	p.E545K	Missense_Mutation	3	178936091	G	A	124	10.48%	Deleterious	CANCER	Driver	likely_pathogenic	TRUE	.	0.42	0.00076	25%	Subclonal	TRUE	TRUE	TRUE	YES	MSK-IMPACT	GAGTAACAGACTAGCTAGAGACAATGA	CTGAGATCAGCCAAATTCAGTTAT
IDC28T	*PRRC2B*	p.P652L	Missense_Mutation	9	134346218	C	T	80	6.25%	Deleterious	PASSENGER/OTHER	Passenger	passenger	.	.	0.25	0.00005	11%	Subclonal	.	.	.	.	WES		
IDC28T	*PRUNE2*	p.C55R	Missense_Mutation	9	79465560	A	G	45	20.00%	Deleterious	PASSENGER/OTHER	Passenger	passenger	.	.	0.8	0.30331	41%	Subclonal	.	.	.	.	WES		
IDC28T	*PTPRN*	p.V907M	Missense_Mutation	2	220156202	C	T	70	7.14%	Deleterious	CANCER	Passenger	likely_pathogenic	.	.	0.28	0.0004	12%	Subclonal	.	.	.	.	WES		
IDC28T	*RBCK1*	p.D425N	Missense_Mutation	20	409198	G	A	82	6.10%	Deleterious	PASSENGER/OTHER	Passenger	passenger	.	.	0.24	0.00003	10%	Subclonal	.	.	.	.	WES		
IDC28T	*RFPL3S*	p.R65W	Missense_Mutation	22	32758977	G	A	49	8.16%	Neutral	.	Passenger	passenger	.	.	0.33	0.00633	13%	Subclonal	.	.	.	.	WES		
IDC28T	*RGS22*	p.R78Q	Missense_Mutation	8	101092468	C	T	123	4.88%	Neutral	PASSENGER/OTHER	Passenger	passenger	.	.	0.39	0.01052	18%	Subclonal	.	.	.	.	WES		
IDC28T	*SCAF11*	p.R82H	Missense_Mutation	12	46345485	C	T	72	6.94%	Deleterious	PASSENGER/OTHER	Passenger	passenger	.	.	0.28	0.00027	12%	Subclonal	.	.	.	.	WES		
IDC28T	*SIPA1L1*	p.G1403S	Missense_Mutation	14	72176317	G	A	46	8.70%	Neutral	CANCER	Driver	likely_pathogenic	.	.	0.35	0.01088	14%	Subclonal	.	.	.	.	WES		
IDC28T	*SLC35A5*	p.D183N	Missense_Mutation	3	112299511	G	A	67	7.46%	Neutral	PASSENGER/OTHER	Passenger	passenger	.	.	0.3	0.00073	13%	Subclonal	.	.	.	.	WES		
IDC28T	*SLC45A4*	p.G155S	Missense_Mutation	8	142229123	C	T	57	21.05%	Deleterious	PASSENGER/OTHER	Passenger	passenger	.	.	1	0.76541	64%	Clonal	.	.	.	.	WES		
IDC28T	*SORCS2*	p.V1111M	Missense_Mutation	4	7738815	G	A	146	4.11%	Deleterious	PASSENGER/OTHER	Passenger	passenger	.	.	0.16	0	7%	Subclonal	.	.	.	.	WES		
IDC28T	*ST6GALNAC5*	p.Q218E	Missense_Mutation	1	77510279	C	G	116	26.72%	Deleterious	PASSENGER/OTHER	Passenger	passenger	.	.	1	0.64837	71%	Clonal	.	.	.	.	WES		
IDC28T	*THRAP3*	p.E938K	Missense_Mutation	1	36769562	G	A	78	7.69%	Deleterious	PASSENGER/OTHER	Passenger	passenger	.	.	0.31	0.00031	14%	Subclonal	TRUE	.	.	.	WES		
IDC28T	*TMPRSS13*	p.A77G	Missense_Mutation	11	117789345	G	C	13	23.08%	Neutral	PASSENGER/OTHER	Passenger	passenger	.	loh	0.92	0.33809	26%	Subclonal	.	.	.	.	WES		
IDC28T	*TSHZ3*	p.V831I	Missense_Mutation	19	31768208	C	T	145	4.83%	Deleterious	PASSENGER/OTHER	Passenger	passenger	.	.	0.19	0	9%	Subclonal	.	TRUE	.	.	WES		
IDC28T	*TTC37*	p.R374C	Missense_Mutation	5	94863731	G	A	62	8.06%	Neutral	PASSENGER/OTHER	Passenger	passenger	.	.	0.32	0.00191	14%	Subclonal	.	.	.	.	WES		
IDC28T	*TTLL6*	p.R464W	Missense_Mutation	17	46867303	G	A	146	4.11%	Neutral	PASSENGER/OTHER	Passenger	passenger	.	.	0.16	0	7%	Subclonal	.	.	.	.	WES		
IDC28T	*UFD1L*	p.R275C	Missense_Mutation	22	19442298	G	A	53	9.43%	Deleterious	PASSENGER/OTHER	Passenger	passenger	.	.	0.38	0.00974	16%	Subclonal	.	.	.	.	WES		
IDC28T	*UGGT2*	p.R1288Q	Missense_Mutation	13	96508557	C	T	48	8.33%	Deleterious	PASSENGER/OTHER	Passenger	passenger	.	.	0.33	0.0076	13%	Subclonal	.	.	.	.	WES		
IDC28T	*VWC2L*	p.G203R	Missense_Mutation	2	215440482	G	A	114	5.26%	Deleterious	PASSENGER/OTHER	Passenger	passenger	.	.	0.21	0	9%	Subclonal	.	.	.	.	WES		
IDC28T	*ZMIZ1*	p.T298P	Missense_Mutation	10	81052048	A	C	110	23.64%	Deleterious	PASSENGER/OTHER	Passenger	passenger	.	.	0.94	0.50391	63%	Clonal	.	.	.	.	WES		
IDC28T	*ZNF518B*	p.P679L	Missense_Mutation	4	10445917	G	A	86	5.81%	Neutral	PASSENGER/OTHER	Passenger	passenger	.	.	0.23	0.00001	10%	Subclonal	.	.	.	.	WES		
IDC28T	*ZNF76*	p.V49I	Missense_Mutation	6	35253976	G	A	111	5.41%	Deleterious	PASSENGER/OTHER	Passenger	passenger	.	.	0.22	0	10%	Subclonal	.	.	.	.	WES		
IDC28T	*ENPP1*	p.X265_splice	Splice_Site	6	132179888	G	C	65	20.00%	Deleterious	.	.	passenger	.	.	0.8	0.2961	46%	Subclonal	.	.	.	.	WES		
IDC28T	*GUCY2C*	p.X536_splice	Splice_Site	12	14804447	T	C	35	22.86%	Deleterious	.	.	passenger	.	.	0.91	0.38996	44%	Subclonal	.	.	.	.	WES		
IDC28T	*NPHP1*	p.X417_splice	Splice_Site	2	110917703	C	T	34	14.71%	Deleterious	.	.	passenger	.	.	0.59	0.14177	25%	Subclonal	.	.	.	.	WES		
IDC28T	*RBM5*	p.X113_splice	Splice_Site	3	50131309	G	A	23	17.39%	Deleterious	.	.	passenger	.	.	0.69	0.23598	26%	Subclonal	.	.	.	.	WES		
IDC33T	*KMT2A*	p.H2176Lfs*38	Frame_Shift_Del	11	118373129	CACTCATGAAATAGTCACAGTAGGTGATCCTTT	C	52	15.38%	Deleterious	.	.	likely_pathogenic	.	loh	0.32	0.00001	16%	subclonal	TRUE	.	TRUE	YES	MSK-IMPACT		
IDC33T	*CIC*	p.A1545T	Missense_Mutation	19	42799149	G	A	431	4.64%	Neutral	.	Passenger	passenger	.	.	0.14	0	9%	subclonal	TRUE	.	.	YES	MSK-IMPACT		
IDC33T	*FGFR4*	p.E61D	Missense_Mutation	5	176517482	G	C	355	6.20%	Neutral	PASSENGER/OTHER	Passenger	passenger	.	.	0.19	0	12%	subclonal	TRUE	.	.	YES	MSK-IMPACT		
IDC33T	*GLI1*	p.R805G	Missense_Mutation	12	57864936	C	G	193	40.93%	Neutral	PASSENGER/OTHER	Passenger	passenger	.	.	1	0.81958	89%	Clonal	.	.	.	YES	MSK-IMPACT		
IDC33T	*MYCL*	p.E92V	Missense_Mutation	1	40366922	T	A	445	30.34%	Neutral	PASSENGER/OTHER	Passenger	passenger	.	.	0.93	0.71752	80%	Clonal	TRUE	.	.	.	MSK-IMPACT		
IDC28T	*PIK3CA*	p.H1047R	Missense_Mutation	3	178952085	A	G	51	27.45%	Deleterious	CANCER	Driver	likely_pathogenic	TRUE	.	1	0.5559	59%	Clonal	TRUE	TRUE	TRUE	YES	WES	TGAGCAAGAGGCTTTGGAGT	TGTGGAATCCAGAGTGAGCTT
IDC35T	*ARID1A*	p.T377Pfs*14	Frame_Shift_Del	1	27024022	GA	G	31	64.52%	.	.	.	likely_pathogenic	.	loh	1	0.91751	81%	Clonal	TRUE	TRUE	TRUE	YES	MSK-IMPACT		
IDC35T	*BRD4*	p.D655A	Missense_Mutation	19	15366191	T	G	151	20.53%	Deleterious	PASSENGER/OTHER	Passenger	passenger	.	loh	0.76	0.32247	54%	Subclonal	TRUE	.	.	YES	MSK-IMPACT		
IDC35T	*BRIP1*	p.G11V	Missense_Mutation	17	59938869	C	A	47	17.02%	Deleterious	PASSENGER/OTHER	Passenger	passenger	.	.	0.8	0.55692	39%	Clonal	TRUE	.	.	YES	MSK-IMPACT		
IDC35T	*DICER1*	p.R848T	Missense_Mutation	14	95574324	C	G	34	23.53%	Deleterious	CANCER	Passenger	likely_pathogenic	.	.	0.87	0.61901	42%	Clonal	TRUE	.	.	YES	MSK-IMPACT		
IDC35T	*FGFR1*	p.V298L	Missense_Mutation	8	38282164	C	G	49	20.41%	Deleterious	CANCER	Passenger	likely_pathogenic	.	loh	0.55	0.11095	31%	Subclonal	TRUE	.	.	YES	MSK-IMPACT		
IDC35T	*IKBKE*	p.V480E	Missense_Mutation	1	206658345	T	A	148	21.62%	Neutral	CANCER	Passenger	passenger	.	.	1	0.86732	76%	Clonal	.	.	.	YES	MSK-IMPACT		
IDC35T	*PIK3CA*	p.H1047R	Missense_Mutation	3	178952085	A	G	82	54.88%	Deleterious	CANCER	Driver	likely_pathogenic	TRUE	.	1	0.3563	91%	Clonal	TRUE	TRUE	TRUE	YES	MSK-IMPACT	TGAGCAAGAGGCTTTGGAGT	TGTGGAATCCAGAGTGAGCTT
IDC35T	*NOTCH3*	p.W724*	Nonsense_Mutation	19	15296192	C	T	149	26.85%	Deleterious	.	.	passenger	.	loh	0.99	0.81844	72%	Clonal	.	.	.	YES	MSK-IMPACT	CAGTGGAAACCCATTCCATC	CCCTTCTCAGCCTCAGACAC
IDC37T	*DNMT3A*	p.N230Tfs*86	Frame_Shift_Del	2	25471071	GT	G	176	21.02%	Deleterious	.	.	likely_pathogenic	.	.	0.99	0.45801	70%	Clonal	TRUE	TRUE	TRUE	YES	WES		
IDC37T	*ASCL2*	p.N67Kfs*134	Frame_Shift_Ins	11	2291362	G	GT	250	10.80%	Deleterious	.	.	likely_pathogenic	.	loh	0.51	0.00005	35%	.	.	.	.	.	WES		
IDC37T	*STAT3*	p.T54_V56delinsM	In_Frame_Del	17	40498693	ACCAAAG	A	107	9.35%	Deleterious	.	.	likely_pathogenic	.	.	0.44	0.00206	24%	subclonal	TRUE	.	.	YES	WES		
IDC37T	*ADRA1D*	p.K520M	Missense_Mutation	20	4202330	T	A	397	5.04%	Deleterious	PASSENGER/OTHER	Passenger	passenger	.	.	0.24	0	15%	subclonal	.	.	.	.	WES		
IDC37T	*AFMID*	p.G8D	Missense_Mutation	17	76183474	G	A	242	6.61%	Neutral	.	Passenger	passenger	.	.	0.31	0	19%	subclonal	.	.	.	.	WES		
IDC37T	*APOBR*	p.E361D	Missense_Mutation	16	28507445	G	C	122	13.93%	Neutral	PASSENGER/OTHER	Passenger	passenger	.	.	0.66	0.04807	41%	subclonal	.	.	.	.	WES		
IDC37T	*ATG12*	p.E4D	Missense_Mutation	5	115177238	C	A	320	3.75%	Deleterious	.	Passenger	passenger	.	.	0.18	0	10%	subclonal	.	.	.	.	WES		
IDC37T	*BNC2*	p.P605T	Missense_Mutation	9	16436379	G	T	334	3.89%	Deleterious	PASSENGER/OTHER	Passenger	passenger	.	.	0.18	0	10%	subclonal	.	.	.	.	WES		
IDC37T	*C19orf55*	p.K104N	Missense_Mutation	19	36252920	G	C	293	3.75%	Deleterious	PASSENGER/OTHER	Passenger	passenger	.	.	0.18	0	9%	subclonal	.	.	.	.	WES		
IDC37T	*CCNI*	p.G286R	Missense_Mutation	4	77969650	C	G	205	6.34%	Deleterious	PASSENGER/OTHER	Passenger	passenger	.	.	0.3	0	17%	subclonal	.	.	.	.	WES		
IDC37T	*CELSR3*	p.Q2106K	Missense_Mutation	3	48688379	G	T	109	12.84%	Deleterious	PASSENGER/OTHER	Driver	likely_pathogenic	.	.	0.61	0.03078	36%	subclonal	.	.	.	.	WES		
IDC37T	*CHRNA3*	p.P119T	Missense_Mutation	15	78909388	G	T	116	6.90%	Deleterious	CANCER	Passenger	likely_pathogenic	.	.	0.33	0.00006	16%	subclonal	.	.	.	.	WES		
IDC37T	*CYP1A2*	p.M68T	Missense_Mutation	15	75042282	T	C	391	5.63%	Deleterious	PASSENGER/OTHER	Passenger	passenger	.	.	0.27	0	17%	subclonal	.	.	.	.	WES		
IDC37T	*CYP26A1*	p.T157A	Missense_Mutation	10	94834590	A	G	245	21.63%	Neutral	PASSENGER/OTHER	Passenger	passenger	.	.	1	0.53827	76%	Clonal	.	.	.	.	WES		
IDC37T	*DNAH10*	p.G2017S	Missense_Mutation	12	124337864	G	A	73	5.48%	Deleterious	PASSENGER/OTHER	Passenger	passenger	.	.	0.26	0.00038	10%	subclonal	.	.	.	.	WES		
IDC37T	*FAM65B*	p.D504E	Missense_Mutation	6	24843498	G	C	182	3.85%	Neutral	PASSENGER/OTHER	Passenger	passenger	.	.	0.18	0	8%	subclonal	.	.	.	.	WES		
IDC37T	*FAT3*	p.Q4492H	Missense_Mutation	11	92623985	G	C	433	6.47%	Deleterious	PASSENGER/OTHER	Passenger	passenger	.	loh	0.24	0	16%	subclonal	.	.	.	.	WES		
IDC37T	*GAB2*	p.R59Q	Missense_Mutation	11	77991847	C	T	311	6.11%	Deleterious	PASSENGER/OTHER	Passenger	passenger	.	.	0.29	0	18%	subclonal	.	.	.	.	WES		
IDC37T	*GAREML*	p.L598P	Missense_Mutation	2	26410294	T	C	544	5.51%	Deleterious	PASSENGER/OTHER	Passenger	passenger	.	.	0.26	0	18%	subclonal	.	.	.	.	WES		
IDC37T	*GNAQ*	p.T96S	Missense_Mutation	9	80537112	T	A	78	23.08%	Deleterious	CANCER	Driver	likely_pathogenic	TRUE	.	1	0.44523	63%	Clonal	TRUE	.	.	.	WES		
IDC37T	*GREB1*	p.T1302S	Missense_Mutation	2	11758905	A	T	571	4.20%	Neutral	PASSENGER/OTHER	Passenger	passenger	.	.	0.2	0	13%	subclonal	.	.	.	.	WES		
IDC37T	*GRM1*	p.S146F	Missense_Mutation	6	146351090	C	T	496	10.69%	Deleterious	CANCER	Passenger	likely_pathogenic	.	.	0.5	0	38%	subclonal	.	.	.	.	WES		
IDC37T	*HOXA1*	p.D31E	Missense_Mutation	7	27135439	G	T	509	10.02%	Deleterious	PASSENGER/OTHER	Passenger	passenger	.	.	0.47	0	36%	subclonal	.	.	.	.	WES		
IDC37T	*HPSE2*	p.Q124H	Missense_Mutation	10	100992181	C	G	179	5.03%	Neutral	PASSENGER/OTHER	Passenger	passenger	.	.	0.24	0	12%	subclonal	.	.	.	.	WES		
IDC37T	*IFITM10*	p.P105A	Missense_Mutation	11	1769121	G	C	338	8.58%	Neutral	PASSENGER/OTHER	Passenger	passenger	.	loh	0.4	0	28%	.	.	.	.	.	WES		
IDC37T	*IL5RA*	p.T189P	Missense_Mutation	3	3139698	T	G	196	9.18%	Neutral	PASSENGER/OTHER	Passenger	passenger	.	loh	0.43	0.00002	27%	subclonal	.	.	.	.	WES		
IDC37T	*INSR*	p.H1358R	Missense_Mutation	19	7117143	T	C	507	4.54%	Deleterious	PASSENGER/OTHER	Passenger	passenger	.	.	0.21	0	14%	subclonal	.	.	.	YES	WES		
IDC37T	*KALRN*	p.L1257I	Missense_Mutation	3	124175496	C	A	264	7.95%	Deleterious	PASSENGER/OTHER	Passenger	passenger	.	.	0.37	0	24%	subclonal	.	.	.	.	WES		
IDC37T	*KCNH1*	p.S854F	Missense_Mutation	1	210857032	G	A	706	5.24%	Deleterious	CANCER	Passenger	likely_pathogenic	.	.	0.3	0	21%	subclonal	.	.	.	.	WES		
IDC37T	*KCNJ10*	p.F165L	Missense_Mutation	1	160011828	G	T	301	8.97%	Deleterious	CANCER	Passenger	likely_pathogenic	.	.	0.51	0.00009	35%	subclonal	.	.	.	.	WES		
IDC37T	*KCNJ10*	p.L166V	Missense_Mutation	1	160011827	G	C	309	8.41%	Deleterious	CANCER	Passenger	likely_pathogenic	.	.	0.48	0.00002	33%	subclonal	.	.	.	.	WES		
IDC37T	*KCNJ3*	p.Q344K	Missense_Mutation	2	155711349	C	A	215	9.77%	Deleterious	CANCER	Passenger	likely_pathogenic	.	.	0.46	0.00003	30%	subclonal	.	.	.	.	WES		
IDC37T	*KCNK10*	p.Q210E	Missense_Mutation	14	88693772	G	C	65	7.69%	Deleterious	PASSENGER/OTHER	Passenger	passenger	.	.	0.36	0.00467	16%	subclonal	.	.	.	.	WES		
IDC37T	*KCNQ2*	p.P57T	Missense_Mutation	20	62103648	G	T	210	4.29%	Deleterious	CANCER	Passenger	likely_pathogenic	.	.	0.2	0	10%	subclonal	.	.	.	.	WES		
IDC37T	*KCTD6*	p.F117S	Missense_Mutation	3	58486995	T	C	172	22.09%	Deleterious	PASSENGER/OTHER	Passenger	passenger	.	.	1	0.51734	73%	Clonal	.	.	.	.	WES		
IDC37T	*KIFC2*	p.D30E	Missense_Mutation	8	145691892	C	A	241	14.94%	Deleterious	PASSENGER/OTHER	Passenger	passenger	.	.	0.7	0.02543	51%	subclonal	.	.	.	.	WES		
IDC37T	*KLHL30*	p.L200Q	Missense_Mutation	2	239049994	T	A	330	3.03%	Neutral	PASSENGER/OTHER	Passenger	passenger	.	.	0.14	0	7%	subclonal	.	.	.	.	WES		
IDC37T	*LAMA5*	p.G87S	Missense_Mutation	20	60942043	C	T	183	5.46%	Deleterious	PASSENGER/OTHER	Passenger	passenger	.	.	0.26	0	14%	subclonal	.	.	.	.	WES		
IDC37T	*LAMB1*	p.Q1236H	Missense_Mutation	7	107580487	C	G	316	18.67%	Deleterious	PASSENGER/OTHER	Passenger	passenger	.	loh	0.88	0.26922	69%	.	.	.	.	.	WES		
IDC37T	*MBD1*	p.G446S	Missense_Mutation	18	47800044	C	T	450	4.22%	Neutral	CANCER	Passenger	passenger	.	.	0.2	0	12%	subclonal	.	.	.	.	WES		
IDC37T	*MRGPRX2*	p.S177Y	Missense_Mutation	11	19077420	G	T	196	8.67%	Neutral	PASSENGER/OTHER	Passenger	passenger	.	.	0.41	0.00001	25%	subclonal	.	.	.	.	WES		
IDC37T	*MRPL4*	p.E205Q	Missense_Mutation	19	10369149	G	C	550	4.36%	Deleterious	.	Passenger	passenger	.	.	0.21	0	13%	subclonal	.	.	.	.	WES		
IDC37T	*MTMR8*	p.V540F	Missense_Mutation	23	63488914	C	A	123	10.57%	Neutral	CANCER	Passenger	passenger	.	loh	0.5	0.00355	29%	subclonal	.	.	.	.	WES		
IDC37T	*MUC16*	p.G10633R	Missense_Mutation	19	9049734	C	T	206	10.19%	.	PASSENGER/OTHER	Passenger	passenger	.	loh	0.48	0.00009	31%	subclonal	.	.	.	.	WES		
IDC37T	*NFATC4*	p.E796K	Missense_Mutation	14	24845640	G	A	513	18.52%	Neutral	PASSENGER/OTHER	Passenger	passenger	.	.	0.87	0.20461	72%	Clonal	.	.	.	.	WES		
IDC37T	*NHLH1*	p.T82M	Missense_Mutation	1	160340766	C	T	452	9.07%	Deleterious	CANCER	Passenger	likely_pathogenic	.	.	0.52	0	38%	subclonal	.	.	.	.	WES		
IDC37T	*NMUR1*	p.G53R	Missense_Mutation	2	232393575	C	T	282	4.96%	Deleterious	PASSENGER/OTHER	Passenger	passenger	.	.	0.23	0	14%	subclonal	.	.	.	.	WES		
IDC37T	*NSMCE2*	p.H88L	Missense_Mutation	8	126163518	A	T	126	7.94%	Deleterious	PASSENGER/OTHER	Passenger	passenger	.	.	0.37	0.00014	20%	subclonal	.	.	.	.	WES		
IDC37T	*OR52B1P*	p.P90R	Missense_Mutation	11	6173550	G	C	273	4.03%	.	PASSENGER/OTHER	Passenger	passenger	.	loh	0.19	0	10%	.	.	.	.	.	WES		
IDC37T	*P2RY12*	p.G225S	Missense_Mutation	3	151055961	C	T	220	4.55%	Neutral	PASSENGER/OTHER	Passenger	passenger	.	.	0.21	0	11%	subclonal	.	.	.	.	WES		
IDC4T	*PIK3CA*	p.H1047R	Missense_Mutation	3	178952085	A	G	188	50.53%	Deleterious	CANCER	Driver	likely_pathogenic	TRUE	.	1	0.94789	95%	Clonal	TRUE	TRUE	TRUE	YES	WES	TGAGCAAGAGGCTTTGGAGT	TGTGGAATCCAGAGTGAGCTT
IDC37T	*PLCH1*	p.K1684N	Missense_Mutation	3	155198787	T	G	176	6.25%	Deleterious	PASSENGER/OTHER	Passenger	passenger	.	.	0.29	0	16%	subclonal	.	.	.	.	WES		
IDC37T	*PQLC1*	p.A22V	Missense_Mutation	18	77710862	G	A	203	19.21%	Deleterious	CANCER	Passenger	likely_pathogenic	.	.	0.91	0.33176	66%	Clonal	.	.	.	.	WES		
IDC37T	*RPAP3*	p.S205A	Missense_Mutation	12	48084355	A	C	117	6.84%	Neutral	PASSENGER/OTHER	Passenger	passenger	.	.	0.32	0.00006	16%	subclonal	.	.	.	.	WES		
IDC37T	*RTF1*	p.R421I	Missense_Mutation	15	41766876	G	T	74	6.76%	Deleterious	.	Passenger	passenger	.	.	0.32	0.00121	14%	subclonal	.	.	.	.	WES		
IDC37T	*SERAC1*	p.A364S	Missense_Mutation	6	158541533	C	A	162	5.56%	Deleterious	PASSENGER/OTHER	Passenger	passenger	.	.	0.26	0	14%	subclonal	.	.	.	.	WES		
IDC37T	*SLC5A1*	p.F36L	Missense_Mutation	22	32439376	C	G	145	4.83%	Deleterious	CANCER	Passenger	likely_pathogenic	.	.	0.23	0	11%	subclonal	.	.	.	.	WES		
IDC37T	*SLCO5A1*	p.E247Q	Missense_Mutation	8	70744170	C	G	356	16.57%	Neutral	PASSENGER/OTHER	Passenger	passenger	.	.	0.95	0.4244	73%	Clonal	.	.	.	.	WES		
IDC37T	*SPPL2C*	p.R215W	Missense_Mutation	17	43922915	C	T	292	14.04%	Deleterious	PASSENGER/OTHER	Passenger	passenger	.	.	0.66	0.0045	49%	subclonal	.	.	.	.	WES		
IDC37T	*STAG1*	p.E634K	Missense_Mutation	3	136141389	C	T	176	8.52%	Deleterious	PASSENGER/OTHER	Passenger	passenger	.	.	0.4	0.00002	24%	subclonal	.	.	.	.	WES		
IDC37T	*STX7*	p.D164N	Missense_Mutation	6	132791137	C	T	123	8.13%	Deleterious	PASSENGER/OTHER	Passenger	passenger	.	.	0.38	0.00022	21%	subclonal	.	.	.	.	WES		
IDC37T	*SYNE1*	p.G7942A	Missense_Mutation	6	152477198	C	G	147	4.76%	Deleterious	PASSENGER/OTHER	Passenger	passenger	.	.	0.22	0	11%	subclonal	.	.	.	.	WES		
IDC37T	*TBC1D14*	p.E418K	Missense_Mutation	4	7002960	G	A	92	5.43%	Deleterious	PASSENGER/OTHER	Passenger	passenger	.	.	0.26	0.00006	11%	subclonal	.	.	.	.	WES		
IDC37T	*TEP1*	p.I934M	Missense_Mutation	14	20854665	G	C	140	10.00%	Deleterious	PASSENGER/OTHER	Passenger	passenger	.	.	0.47	0.001	28%	subclonal	.	.	.	.	WES		
IDC37T	*TET3*	p.R829Q	Missense_Mutation	2	74317026	G	A	68	5.88%	Deleterious	PASSENGER/OTHER	Passenger	passenger	.	.	0.28	0.00087	11%	subclonal	.	.	.	.	WES		
IDC37T	*TGIF2LX*	p.E209G	Missense_Mutation	23	89177710	A	G	440	4.77%	Neutral	PASSENGER/OTHER	Passenger	passenger	.	loh	0.22	0	14%	subclonal	.	.	.	.	WES		
IDC37T	*TLX2*	p.P248L	Missense_Mutation	2	74743204	C	T	213	8.92%	Deleterious	CANCER	Passenger	likely_pathogenic	.	.	0.42	0.00001	27%	subclonal	.	.	.	.	WES		
IDC37T	*TMEM132C*	p.A17V	Missense_Mutation	12	128751997	C	T	30	10.00%	Neutral	PASSENGER/OTHER	Passenger	passenger	.	.	0.47	0.06394	16%	subclonal	.	.	.	.	WES		
IDC37T	*TNFSF9*	p.R71H	Missense_Mutation	19	6531259	G	A	77	7.79%	Neutral	PASSENGER/OTHER	Passenger	passenger	.	.	0.37	0.00244	17%	subclonal	.	.	.	.	WES		
IDC37T	*TRPM8*	p.K822E	Missense_Mutation	2	234890434	A	G	60	8.33%	Neutral	PASSENGER/OTHER	Passenger	passenger	.	.	0.39	0.00938	17%	subclonal	.	.	.	.	WES		
IDC37T	*WDR24*	p.G12S	Missense_Mutation	16	739607	C	T	651	2.92%	Neutral	PASSENGER/OTHER	Passenger	passenger	.	.	0.14	0	8%	subclonal	.	.	.	.	WES		
IDC37T	*ZBTB17*	p.I523F	Missense_Mutation	1	16270103	T	A	434	5.53%	Deleterious	PASSENGER/OTHER	Passenger	passenger	.	.	0.26	0	17%	subclonal	.	.	.	.	WES		
IDC37T	*ATN1*	p.Q492*	Nonsense_Mutation	12	7045904	C	T	319	5.02%	Deleterious	.	.	passenger	.	.	0.24	0	14%	subclonal	.	.	.	.	WES		
IDC37T	*GSG1L*	p.Q302*	Nonsense_Mutation	16	27802783	G	A	53	7.55%	Deleterious	.	.	passenger	.	.	0.36	0.00879	14%	subclonal	.	.	.	.	WES		
IDC37T	*PAXBP1*	p.R410*	Nonsense_Mutation	21	34131546	G	A	137	4.38%	Deleterious	.	.	passenger	.	.	0.21	0	9%	subclonal	.	.	.	.	WES		
IDC37T	*TCP11*	p.Q307*	Nonsense_Mutation	6	35088259	G	A	215	5.58%	Deleterious	.	.	passenger	.	.	0.26	0	15%	subclonal	.	.	.	.	WES		
IDC37T	*USP47*	p.R244*	Nonsense_Mutation	11	11927060	C	T	90	21.11%	Deleterious	.	.	passenger	.	.	0.99	0.3901	61%	Clonal	.	.	.	.	WES		
IDC37T	*WBSCR22*	p.X171_splice	Splice_Site	7	73107658	G	T	228	6.58%	Deleterious	.	.	passenger	.	.	0.31	0	19%	subclonal	.	.	.	.	WES		
IDC37T	*CLDN10*	p.M1?	Translation_Start_Site	13	96086090	G	A	325	15.08%	Deleterious	CANCER	Passenger	likely_pathogenic	.	loh	0.71	0.01223	54%	.	.	.	.	.	WES		
IDC3T	*ABCA3*	p.T1173M	Missense_Mutation	16	2334965	G	A	84	5.95%	Deleterious	PASSENGER/OTHER	Passenger	passenger	.	.	0.47	0.20126	20%	Subclonal	.	.	.	.	WES		
IDC3T	*AHNAK*	p.E4573D	Missense_Mutation	11	62288170	T	G	88	17.05%	Neutral	PASSENGER/OTHER	Passenger	passenger	.	.	1	0.79125	56%	Clonal	.	.	.	.	WES		
IDC3T	*ATG4B*	p.T99S	Missense_Mutation	2	242593972	A	T	72	34.72%	Neutral	PASSENGER/OTHER	Passenger	passenger	.	.	1	0.96199	81%	Clonal	.	.	.	.	WES		
IDC3T	*BAI1*	p.P1449S	Missense_Mutation	8	143623940	C	T	27	14.81%	Neutral	PASSENGER/OTHER	Passenger	passenger	.	.	1	0.71037	32%	Clonal	.	.	.	.	WES		
IDC3T	*C1orf61*	p.Y124S	Missense_Mutation	1	156376924	T	G	37	18.92%	Neutral	.	Passenger	passenger	.	.	1	0.85492	50%	Clonal	.	.	.	.	WES		
IDC3T	*COL15A1*	p.E417K	Missense_Mutation	9	101767228	G	A	116	7.76%	Neutral	PASSENGER/OTHER	Passenger	passenger	.	.	0.53	0.16102	28%	Subclonal	.	.	.	.	WES		
IDC3T	*COL19A1*	p.D661N	Missense_Mutation	6	70859794	G	A	125	12.80%	Neutral	PASSENGER/OTHER	Passenger	passenger	.	.	0.75	0.52752	46%	Clonal	.	.	.	.	WES		
IDC3T	*DAXX*	p.S461P	Missense_Mutation	6	33287872	A	G	111	40.54%	Neutral	PASSENGER/OTHER	Passenger	passenger	.	.	1	0.9207	90%	Clonal	TRUE	.	.	YES	WES		
IDC3T	*DNAH11*	p.D4248N	Missense_Mutation	7	21932256	G	A	80	18.75%	Deleterious	PASSENGER/OTHER	Passenger	passenger	.	.	1	0.85885	64%	Clonal	.	.	.	.	WES		
IDC3T	*DOPEY2*	p.S1237F	Missense_Mutation	21	37617988	C	T	232	3.45%	Deleterious	PASSENGER/OTHER	Passenger	passenger	.	.	0.24	0	12%	Subclonal	.	.	.	.	WES		
IDC3T	*DSCAM*	p.R349C	Missense_Mutation	21	41719762	G	A	84	40.48%	Deleterious	PASSENGER/OTHER	Passenger	passenger	.	.	1	0.71312	87%	Clonal	.	.	.	.	WES		
IDC3T	*EP400*	p.R1125H	Missense_Mutation	12	132496112	G	A	98	5.10%	Deleterious	PASSENGER/OTHER	Passenger	passenger	.	.	0.35	0.02779	15%	Subclonal	.	.	.	.	WES		
IDC3T	*FLNC*	p.R2690W	Missense_Mutation	7	128498467	C	T	73	5.48%	Deleterious	PASSENGER/OTHER	Passenger	passenger	.	.	0.38	0.09458	15%	Subclonal	.	.	.	.	WES		
IDC3T	*GRM1*	p.S749I	Missense_Mutation	6	146720421	G	T	149	34.90%	Deleterious	PASSENGER/OTHER	Passenger	passenger	.	.	1	0.9728	90%	Clonal	.	.	.	.	WES		
IDC3T	*GSTP1*	p.K116Q	Missense_Mutation	11	67353584	A	C	49	18.37%	Deleterious	PASSENGER/OTHER	Passenger	passenger	.	.	1	0.916	61%	Clonal	.	.	.	.	WES		
IDC3T	*KEAP1*	p.Y426C	Missense_Mutation	19	10602301	T	C	146	26.71%	Deleterious	PASSENGER/OTHER	Passenger	passenger	.	.	1	0.86899	84%	Clonal	.	TRUE	TRUE	YES	WES		
IDC3T	*KRTAP4-11*	p.R51K	Missense_Mutation	17	39274416	C	T	74	9.46%	Neutral	PASSENGER/OTHER	Passenger	passenger	.	.	1	0.88894	53%	Clonal	.	.	.	.	WES		
IDC3T	*LAMA2*	p.D2498Y	Missense_Mutation	6	129799878	G	T	82	18.29%	Deleterious	PASSENGER/OTHER	Passenger	passenger	.	.	1	0.81801	59%	Clonal	.	.	.	.	WES		
IDC3T	*MID1*	p.R160C	Missense_Mutation	X	10535110	G	A	230	3.48%	Deleterious	PASSENGER/OTHER	Passenger	passenger	.	.	0.24	0	12%	Subclonal	.	.	.	.	WES		
IDC3T	*MOCS1*	p.G28S	Missense_Mutation	6	39895236	C	T	75	18.67%	Neutral	PASSENGER/OTHER	Passenger	passenger	.	.	1	0.8189	58%	Clonal	.	.	.	.	WES		
IDC3T	*MTR*	p.I1124M	Missense_Mutation	1	237057824	C	G	154	24.68%	Deleterious	PASSENGER/OTHER	Driver	likely_pathogenic	.	.	1	0.96706	86%	Clonal	.	.	.	.	WES		
IDC3T	*OR6Y1*	p.C146F	Missense_Mutation	1	158517459	C	A	134	5.97%	Neutral	PASSENGER/OTHER	Passenger	passenger	.	.	0.47	0.08272	24%	Subclonal	.	.	.	.	WES		
IDC3T	*PDZD8*	p.G909E	Missense_Mutation	10	119043518	C	T	124	41.13%	Neutral	PASSENGER/OTHER	Passenger	passenger	.	.	1	0.89888	91%	Clonal	.	.	.	.	WES		
IDC3T	*PODXL2*	p.L142H	Missense_Mutation	3	127379296	T	A	127	28.35%	Neutral	PASSENGER/OTHER	Passenger	passenger	.	.	1	0.96725	85%	Clonal	.	.	.	.	WES		
IDC3T	*RBM47*	p.A207G	Missense_Mutation	4	40440291	G	C	252	29.37%	Deleterious	PASSENGER/OTHER	Passenger	passenger	.	.	1	0.97669	92%	Clonal	.	.	.	.	WES		
IDC3T	*RORB*	p.Q111H	Missense_Mutation	9	77257427	G	C	138	28.26%	Deleterious	PASSENGER/OTHER	Passenger	passenger	.	.	1	0.96834	86%	Clonal	.	.	.	.	WES		
IDC3T	*SCARF2*	p.P11L	Missense_Mutation	22	20792010	G	A	8	37.50%	Deleterious	PASSENGER/OTHER	Passenger	passenger	.	.	1	0.83757	36%	Clonal	.	.	.	.	WES		
IDC3T	*SLC5A10*	p.Y183F	Missense_Mutation	17	18872459	A	T	131	42.75%	Deleterious	PASSENGER/OTHER	Passenger	passenger	.	loh	1	0.97405	91%	Clonal	.	.	.	.	WES		
IDC3T	*TBX3*	p.N297H	Missense_Mutation	12	115115437	T	G	124	27.42%	Deleterious	PASSENGER/OTHER	Passenger	passenger	.	.	1	0.96477	84%	Clonal	.	TRUE	TRUE	YES	WES		
IDC3T	*THYN1*	p.H182Y	Missense_Mutation	11	134118790	G	A	44	9.09%	Deleterious	.	Passenger	passenger	.	.	0.62	0.48502	24%	Subclonal	.	.	.	.	WES		
IDC3T	*TP53*	p.R280T	Missense_Mutation	17	7577099	C	G	75	42.67%	Deleterious	CANCER	Passenger	likely_pathogenic	TRUE	loh	1	0.96712	85%	Clonal	TRUE	TRUE	TRUE	YES	WES	GCTTCTTGTCCTGCTTGCTT	CTACTGGGACGGAACAGCTT
IDC3T	*VPS33A*	p.S402L	Missense_Mutation	12	122723231	G	A	95	5.26%	Deleterious	PASSENGER/OTHER	Passenger	passenger	.	.	0.36	0.03661	15%	Subclonal	.	.	.	.	WES		
IDC3T	*ZNF691*	p.R219W	Missense_Mutation	1	43317191	C	T	182	13.19%	Deleterious	PASSENGER/OTHER	Passenger	passenger	.	.	0.77	0.52132	52%	Clonal	.	.	.	.	WES		
IDC3T	*IGSF11*	p.Q368*	Nonsense_Mutation	3	118621561	G	A	141	9.22%	Deleterious	.	.	passenger	.	.	0.63	0.28915	37%	Subclonal	.	.	.	.	WES		
IDC3T	*RTN4*	p.E187*	Nonsense_Mutation	2	55255354	C	A	67	20.90%	Deleterious	.	.	passenger	.	.	1	0.84634	62%	Clonal	.	.	.	.	WES		
IDC3T	*SLC5A10*	p.Y183*	Nonsense_Mutation	17	18872460	C	G	132	42.42%	Deleterious	.	.	likely_pathogenic	.	loh	1	0.97475	91%	Clonal	.	.	.	.	WES		
IDC3T	*CST1*	p.X76_splice	Splice_Site	20	23731275	C	T	167	43.71%	Neutral	.	.	passenger	.	.	1	0.26029	94%	Clonal	.	.	.	.	WES		
IDC46T	*AARS*	p.S886Lfs*29	Frame_Shift_Del	16	70287234	AG	A	37	59.46%	.	.	.	passenger	.	.	1	0.84831	83%	Clonal	.	.	.	.	WES		
IDC46T	*KIF12*	p.P18Lfs*20	Frame_Shift_Del	9	116859929	AG	A	33	12.12%	.	.	.	passenger	.	.	0.66	0.53029	25%	Clonal	.	.	.	.	WES		
IDC46T	*ANKRD12*	p.H653Tfs*2	Frame_Shift_Ins	18	9255214	T	TA	22	63.64%	.	.	.	passenger	.	.	1	0.54983	76%	Clonal	.	.	.	.	WES		
IDC46T	*ZNF664*	p.G223Kfs*45	Frame_Shift_Ins	12	124497356	C	CAA	19	15.79%	.	.	.	likely_pathogenic	.	loh	0.54	0.41554	18%	Subclonal	.	.	.	.	WES		
IDC46T	*ARPC5L*	p.A35del	In_Frame_Del	9	127631655	AGGC	A	27	11.11%	.	.	.	passenger	.	.	0.6	0.49805	20%	Subclonal	.	.	.	.	WES		
IDC46T	*COL6A6*	p.E345del	In_Frame_Del	3	130284207	CAGA	C	26	15.38%	.	.	.	passenger	.	.	0.83	0.65166	29%	Clonal	.	.	.	.	WES		
IDC46T	*FAM155B*	p.K372del	In_Frame_Del	X	68749483	CGAA	C	26	15.38%	.	.	.	passenger	.	.	0.68	0.55087	26%	Clonal	.	.	.	.	WES		
IDC46T	*TCRB*	p.P2017del	In_Frame_Del	7	141920357	ACCC	A	9	44.44%	.	.	.	passenger	.	loh	1	0.83804	40%	Clonal	.	.	.	.	WES		
IDC46T	*AKAP14*	p.Y32F	Missense_Mutation	X	119037281	A	T	27	18.52%	Neutral	.	Passenger	passenger	.	.	0.82	0.65157	33%	Clonal	.	.	.	.	WES		
IDC46T	*ANK1*	p.C992W	Missense_Mutation	8	41553988	G	C	97	38.14%	Deleterious	PASSENGER/OTHER	Passenger	passenger	.	.	1	0.96738	87%	Clonal	.	.	.	.	WES		
IDC46T	*ARHGAP27*	p.V423I	Missense_Mutation	17	43473445	C	T	32	53.13%	Deleterious	PASSENGER/OTHER	Passenger	passenger	.	loh	1	0.95783	76%	Clonal	.	.	.	.	WES		
IDC46T	*ARHGEF2*	p.A331T	Missense_Mutation	1	155932494	C	T	33	57.58%	Deleterious	PASSENGER/OTHER	Passenger	passenger	.	.	1	0.60509	81%	Clonal	.	.	.	.	WES		
IDC46T	*AZI1*	p.E936K	Missense_Mutation	17	79164844	C	T	47	55.32%	Deleterious	PASSENGER/OTHER	Passenger	passenger	.	loh	1	0.9686	83%	Clonal	.	.	.	.	WES		

IDC46T	*C5orf47*	p.R6Q	Missense_Mutation	5	173416283	G	A	76	6.58%	Neutral	.	Passenger	passenger	.	loh	0.36	0.03074	15%	Subclonal	.	.	.	.	WES		
IDC46T	*C8orf17*	p.K80E	Missense_Mutation	8	140944767	A	G	48	10.42%	Neutral	PASSENGER/OTHER	Passenger	passenger	.	loh	1	0.82853	42%	Clonal	.	.	.	.	WES		
IDC46T	*CAPRIN1*	p.Q489E	Missense_Mutation	11	34112136	C	G	9	66.67%	Deleterious	PASSENGER/OTHER	Driver	likely_pathogenic	.	loh	1	0.92702	55%	Clonal	.	.	.	.	WES		
IDC46T	*CCDC13*	p.T177S	Missense_Mutation	3	42793501	G	C	50	46.00%	Neutral	PASSENGER/OTHER	Passenger	passenger	.	.	1	0.9032	82%	Clonal	.	.	.	.	WES		
IDC46T	*CLCN7*	p.R674Q	Missense_Mutation	16	1497708	C	T	21	14.29%	Deleterious	PASSENGER/OTHER	Passenger	passenger	.	.	0.92	0.67653	26%	Clonal	.	.	.	.	WES		
IDC46T	*CLUH*	p.A1289T	Missense_Mutation	17	2593953	C	T	35	11.43%	Neutral	PASSENGER/OTHER	Passenger	passenger	.	loh	0.39	0.09125	15%	Subclonal	.	.	.	.	WES		
IDC46T	*COL9A2*	p.R504Q	Missense_Mutation	1	40769239	C	T	43	9.30%	Deleterious	PASSENGER/OTHER	Passenger	passenger	.	.	0.5	0.32479	20%	Subclonal	.	.	.	.	WES		
IDC46T	*CRISP2*	p.C232W	Missense_Mutation	6	49660522	G	C	5	60.00%	Deleterious	PASSENGER/OTHER	Passenger	passenger	.	loh	1	0.86668	36%	Clonal	.	.	.	.	WES		
IDC46T	*DCAF4*	p.A250E	Missense_Mutation	14	73418526	C	A	18	22.22%	Deleterious	PASSENGER/OTHER	Passenger	passenger	.	.	1	0.77077	35%	Clonal	.	.	.	.	WES		
IDC46T	*DIAPH2*	p.R1070G	Missense_Mutation	X	96684711	A	G	13	46.15%	Deleterious	PASSENGER/OTHER	Passenger	passenger	.	.	1	0.9136	53%	Clonal	.	.	.	.	WES		
IDC46T	*DNAH10*	p.D3337A	Missense_Mutation	12	124398887	A	C	50	6.00%	Deleterious	PASSENGER/OTHER	Passenger	passenger	.	loh	0.21	0.00071	7%	Subclonal	.	.	.	.	WES		
IDC46T	*DNAH11*	p.L278V	Missense_Mutation	7	21599360	C	G	19	31.58%	Deleterious	PASSENGER/OTHER	Passenger	passenger	.	.	1	0.88441	50%	Clonal	.	.	.	.	WES		
IDC46T	*FBXL19*	p.E171K	Missense_Mutation	16	30939108	G	A	40	10.00%	Deleterious	PASSENGER/OTHER	Passenger	passenger	.	.	0.54	0.38756	21%	Subclonal	.	.	.	.	WES		
IDC46T	*FOXO4*	p.P394L	Missense_Mutation	X	70321261	C	T	30	36.67%	Neutral	PASSENGER/OTHER	Passenger	passenger	.	.	1	0.91511	64%	Clonal	TRUE	.	.	.	WES		
IDC46T	*FUBP1*	p.Q548K	Missense_Mutation	1	78422320	G	T	20	30.00%	Deleterious	PASSENGER/OTHER	Passenger	passenger	.	.	1	0.87412	49%	Clonal	TRUE	.	.	YES	WES		
IDC46T	*FUK*	p.P215R	Missense_Mutation	16	70501850	C	G	29	24.14%	Deleterious	PASSENGER/OTHER	Passenger	passenger	.	.	1	0.77366	45%	Clonal	.	.	.	.	WES		
IDC46T	*HCRTR2*	p.D415H	Missense_Mutation	6	55147160	G	C	12	33.33%	Deleterious	PASSENGER/OTHER	Passenger	passenger	.	loh	1	0.75935	36%	Clonal	.	.	.	.	WES		
IDC46T	*INSIG1*	p.S43Y	Missense_Mutation	7	155090123	C	A	25	48.00%	Neutral	PASSENGER/OTHER	Passenger	passenger	.	loh	1	0.92487	67%	Clonal	.	.	.	.	WES		
IDC46T	*INSIG2*	p.I63S	Missense_Mutation	2	118854320	T	G	7	42.86%	Deleterious	.	Passenger	passenger	.	loh	1	0.80682	33%	Clonal	.	.	.	.	WES		
IDC46T	*KLHL21*	p.V451L	Missense_Mutation	1	6659183	C	G	84	35.71%	Deleterious	PASSENGER/OTHER	Passenger	passenger	.	.	1	0.96573	83%	Clonal	.	.	.	.	WES		
IDC46T	*LAMA5*	p.T2836I	Missense_Mutation	20	60888856	G	A	20	35.00%	Deleterious	PASSENGER/OTHER	Passenger	passenger	.	.	1	0.9116	55%	Clonal	.	.	.	.	WES		
IDC46T	*LRRTM1*	p.N123I	Missense_Mutation	2	80530577	T	A	34	26.47%	Deleterious	PASSENGER/OTHER	Driver	likely_pathogenic	.	.	1	0.86825	56%	Clonal	.	.	.	.	WES		
IDC46T	*MEF2A*	p.R405C	Missense_Mutation	15	100252689	C	T	46	8.70%	Deleterious	PASSENGER/OTHER	Passenger	passenger	.	.	0.47	0.26469	18%	Subclonal	.	.	.	.	WES		
IDC46T	*METTL13*	p.F223S	Missense_Mutation	1	171753394	T	C	47	12.77%	Deleterious	PASSENGER/OTHER	Passenger	passenger	.	.	0.69	0.54206	31%	Clonal	.	.	.	.	WES		
IDC46T	*METTL22*	p.E350K	Missense_Mutation	16	8738451	G	A	33	27.27%	Deleterious	PASSENGER/OTHER	Passenger	passenger	.	.	1	0.87665	57%	Clonal	.	.	.	.	WES		
IDC46T	*MYEF2*	p.R31Q	Missense_Mutation	15	48470343	C	T	21	57.14%	Neutral	PASSENGER/OTHER	Passenger	passenger	.	loh	1	0.95609	70%	Clonal	.	.	.	.	WES		
IDC46T	*NCKAP5L*	p.P235H	Missense_Mutation	12	50190939	G	T	23	30.43%	Neutral	PASSENGER/OTHER	Passenger	passenger	.	.	1	0.88867	53%	Clonal	.	.	.	.	WES		
IDC46T	*NKX6-2*	p.G139S	Missense_Mutation	10	134598948	C	T	26	69.23%	Deleterious	PASSENGER/OTHER	Passenger	passenger	.	loh	1	0.93548	80%	Clonal	.	.	.	.	WES		
IDC46T	*PCDHA13*	p.F622Y	Missense_Mutation	5	140263718	T	A	142	14.79%	Neutral	PASSENGER/OTHER	Passenger	passenger	.	loh	0.8	0.61861	52%	Clonal	.	.	.	.	WES		
IDC46T	*PCDHB7*	p.N789K	Missense_Mutation	5	140554783	T	A	9	55.56%	Neutral	PASSENGER/OTHER	Passenger	passenger	.	loh	1	0.84902	51%	Clonal	.	.	.	.	WES		
IDC46T	*PKLR*	p.T88A	Missense_Mutation	1	155269910	T	C	28	64.29%	Deleterious	PASSENGER/OTHER	Passenger	passenger	.	.	1	0.46129	81%	Subclonal	.	.	.	.	WES		
IDC46T	*PODXL*	p.Q149H	Missense_Mutation	7	131195846	C	A	67	28.36%	Neutral	PASSENGER/OTHER	Passenger	passenger	.	.	1	0.95178	75%	Clonal	.	.	.	.	WES		
IDC46T	*POTEE*	p.K913T	Missense_Mutation	2	132021766	A	C	19	21.05%	Deleterious	PASSENGER/OTHER	Passenger	passenger	.	loh	0.72	0.58199	27%	Clonal	.	.	.	.	WES		
IDC46T	*PRTFDC1*	p.G119V	Missense_Mutation	10	25160976	C	A	85	77.65%	Deleterious	PASSENGER/OTHER	Passenger	passenger	.	.	1	0.46889	94%	Clonal	.	.	.	.	WES		
IDC46T	*REXO1*	p.S505T	Missense_Mutation	19	1827275	A	T	45	11.11%	Neutral	PASSENGER/OTHER	Passenger	passenger	.	loh	0.38	0.03725	16%	Subclonal	.	.	.	.	WES		
IDC46T	*RNF215*	p.D376N	Missense_Mutation	22	30775597	C	T	16	25.00%	Deleterious	PASSENGER/OTHER	Passenger	passenger	.	loh	0.86	0.66501	30%	Clonal	.	.	.	.	WES		
IDC46T	*RP1L1*	p.R526Q	Missense_Mutation	8	10470031	C	T	87	5.75%	Neutral	PASSENGER/OTHER	Passenger	passenger	.	loh	0.2	0	8%	Subclonal	.	.	.	.	WES		
IDC46T	*SLC5A9*	p.F553C	Missense_Mutation	1	48705115	T	G	27	14.81%	Deleterious	PASSENGER/OTHER	Passenger	passenger	.	.	0.8	0.63581	28%	Clonal	.	.	.	.	WES		
IDC46T	*SOX3*	p.A234V	Missense_Mutation	X	139586525	G	A	25	16.00%	Neutral	PASSENGER/OTHER	Passenger	passenger	.	.	0.71	0.57395	26%	Clonal	.	.	.	.	WES		
IDC46T	*SRRM1*	p.P566H	Missense_Mutation	1	24993374	C	A	31	12.90%	Deleterious	PASSENGER/OTHER	Driver	likely_pathogenic	.	.	0.7	0.56754	26%	Clonal	.	.	.	.	WES		
IDC46T	*SRRM1*	p.R570L	Missense_Mutation	1	24993386	G	T	35	11.43%	Deleterious	PASSENGER/OTHER	Driver	likely_pathogenic	.	.	0.62	0.49112	24%	Subclonal	.	.	.	.	WES		
IDC46T	*STOX2*	p.S547L	Missense_Mutation	4	184931631	C	T	22	18.18%	Neutral	PASSENGER/OTHER	Passenger	passenger	.	.	1	0.76097	35%	Clonal	.	.	.	.	WES		
IDC46T	*TCEB3B*	p.E336D	Missense_Mutation	18	44560628	C	A	46	39.13%	Neutral	PASSENGER/OTHER	Passenger	passenger	.	loh	1	0.88254	71%	Clonal	.	.	.	.	WES		
IDC46T	*THRB*	p.T157M	Missense_Mutation	3	24188228	G	A	11	27.27%	Deleterious	PASSENGER/OTHER	Passenger	passenger	.	.	1	0.79833	32%	Clonal	.	.	.	.	WES		
IDC46T	*TRANK1*	p.T892M	Missense_Mutation	3	36898406	G	A	45	8.89%	Deleterious	PASSENGER/OTHER	Passenger	passenger	.	.	0.48	0.28427	19%	Subclonal	.	.	.	.	WES		
IDC46T	*TXLNB*	p.G81R	Missense_Mutation	6	139609796	C	G	19	63.16%	Neutral	PASSENGER/OTHER	Passenger	passenger	.	loh	1	0.9536	71%	Clonal	.	.	.	.	WES		
IDC46T	*UBR4*	p.A1126V	Missense_Mutation	1	19499502	G	A	32	15.63%	Deleterious	PASSENGER/OTHER	Passenger	passenger	.	.	0.85	0.66493	33%	Clonal	.	.	.	.	WES		
IDC46T	*VCL*	p.R759Q	Missense_Mutation	10	75864953	G	A	16	18.75%	Deleterious	PASSENGER/OTHER	Passenger	passenger	.	.	1	0.70785	27%	Clonal	.	.	.	.	WES		
IDC46T	*ZC3H13*	p.R1343P	Missense_Mutation	13	46541932	C	G	41	9.76%	Deleterious	PASSENGER/OTHER	Passenger	passenger	.	loh	0.33	0.02747	13%	Subclonal	.	.	.	.	WES		
IDC46T	*ZNF589*	p.A15T	Missense_Mutation	3	48282704	G	A	38	13.16%	Neutral	PASSENGER/OTHER	Passenger	passenger	.	loh	0.45	0.133	19%	Subclonal	.	.	.	.	WES		
IDC46T	*ZNF707*	p.V57I	Missense_Mutation	8	144773796	G	A	24	16.67%	Neutral	PASSENGER/OTHER	Passenger	passenger	.	loh	1	0.77509	35%	Clonal	.	.	.	.	WES		
IDC46T	*ZNF747*	p.P105H	Missense_Mutation	16	30545687	G	T	61	26.23%	Neutral	PASSENGER/OTHER	Passenger	passenger	.	.	1	0.88747	68%	Clonal	.	.	.	.	WES		
IDC46T	*ZSWIM3*	p.D113N	Missense_Mutation	20	44505534	G	A	31	12.90%	Neutral	PASSENGER/OTHER	Passenger	passenger	.	loh	0.44	0.17829	17%	Subclonal	.	.	.	.	WES		
IDC46T	*ZXDB*	p.K289T	Missense_Mutation	X	57619347	A	C	65	32.31%	Deleterious	PASSENGER/OTHER	Passenger	passenger	.	.	1	0.88707	74%	Clonal	.	.	.	.	WES		
IDC46T	*KAT6B*	p.Q1999*	Nonsense_Mutation	10	76790577	C	T	35	17.14%	Deleterious	.	.	likely_pathogenic	.	.	0.93	0.71189	38%	Clonal	TRUE	.	.	.	WES		
IDC46T	*PDZRN4*	p.R563*	Nonsense_Mutation	12	41966268	C	T	42	38.10%	Deleterious	.	.	passenger	.	.	1	0.95212	74%	Clonal	.	.	.	.	WES		
IDC46T	*SLC29A2*	p.W291*	Nonsense_Mutation	11	66133697	C	T	29	10.34%	Deleterious	PASSENGER/OTHER	Passenger	passenger	.	.	0.56	0.45499	19%	Subclonal	.	.	.	.	WES		
IDC46T	*TNS1*	p.R346*	Nonsense_Mutation	2	218745639	G	A	30	13.33%	Deleterious	.	.	likely_pathogenic	.	loh	0.46	0.20651	18%	Subclonal	.	.	.	.	WES		
IDC46T	*ATP4A*	p.X501_splice	Splice_Site	19	36048750	C	T	33	39.39%	Deleterious	.	.	passenger	.	.	1	0.94434	71%	Clonal	.	.	.	.	WES		
IDC46T	*TMPRSS6*	p.X623_splice	Splice_Site	22	37465385	CTGGGG	C	14	28.57%	.	.	.	likely_pathogenic	.	loh	0.98	0.71302	33%	Clonal	.	.	.	.	WES		
IDC49T	*CD274*	p.E31Afs*3	Frame_Shift_Del	9	5457111	GTGGTAGAGTA	G	70	5.71%	.	.	.	likely_pathogenic	.	.	0.23	0.00024	9%	Subclonal	TRUE	.	.	YES	MSK-IMPACT		
IDC49T	*CHEK2*	p.K416E	Missense_Mutation	22	29091840	T	C	83	15.66%	Deleterious	PASSENGER/OTHER	Passenger	passenger	.	loh	0.47	0.00432	28%	Subclonal	TRUE	TRUE	.	.	MSK-IMPACT		
IDC49T	*GNAQ*	p.T96S	Missense_Mutation	9	80537112	T	A	118	23.73%	Deleterious	CANCER	Driver	likely_pathogenic	TRUE	.	0.95	0.71987	65%	Clonal	TRUE	.	.	YES	MSK-IMPACT		
IDC49T	*PIK3CA*	p.N345K	Missense_Mutation	3	178921553	T	A	40	35.00%	Deleterious	CANCER	Driver	likely_pathogenic	TRUE	.	1	0.82038	66%	Clonal	TRUE	TRUE	TRUE	YES	MSK-IMPACT	CAGACGCATTTCCACAGCTA	GCATCAGCATTTGACTTTACCTT
IDC4T	*NCOR1*	p.T1361Lfs*17	Frame_Shift_Del	17	15974793	GT	G	161	67.08%	.	.	.	likely_pathogenic	.	loh	1	0.87262	96%	Clonal	.	TRUE	TRUE	YES	WES		
IDC4T	*PLEKHH3*	p.C406Sfs*63	Frame_Shift_Del	17	40823422	GACAGTGCACGGTAC	G	40	22.50%	.	.	.	likely_pathogenic	.	loh	0.76	0.5123	40%	Clonal	.	.	.	.	WES		
IDC4T	*PLEKHS1*	p.H192Qfs*10	Frame_Shift_Del	10	115531768	CACCTACA	C	107	7.48%	.	.	.	likely_pathogenic	.	loh	0.33	0.00033	16%	Subclonal	.	.	.	.	WES		
IDC4T	*ZNF354A*	p.V173Gfs*87	Frame_Shift_Del	5	178140351	CCTAATAAGCA	C	113	10.62%	.	.	.	passenger	.	.	0.36	0.00002	20%	Subclonal	.	.	.	.	WES		
IDC4T	*OR5M8*	p.S229_G232del	In_Frame_Del	11	56258150	TGCCCTCTGTAGA	T	88	5.68%	.	.	.	passenger	.	.	0.25	0.00017	10%	Subclonal	.	.	.	.	WES		
IDC4T	*AADACL2*	p.S63F	Missense_Mutation	3	151458483	C	T	64	15.63%	Neutral	PASSENGER/OTHER	Passenger	passenger	.	.	0.84	0.60447	44%	Clonal	.	.	.	.	WES		
IDC4T	*ABCG1*	p.S85L	Missense_Mutation	21	43645992	C	T	69	15.94%	Deleterious	PASSENGER/OTHER	Passenger	passenger	.	loh	0.7	0.39666	39%	Subclonal	.	.	.	.	WES		
IDC4T	*ANO5*	p.A479S	Missense_Mutation	11	22281092	G	T	242	18.60%	Deleterious	PASSENGER/OTHER	Passenger	passenger	.	.	1	0.83654	85%	Clonal	.	.	.	.	WES		
IDC4T	*ATP6AP1*	p.H459L	Missense_Mutation	X	153664200	A	T	175	26.29%	Deleterious	.	Passenger	passenger	.	.	1	0.89157	79%	Clonal	.	.	.	.	WES		
IDC4T	*BAIAP2L2*	p.R523C	Missense_Mutation	22	38481330	G	A	46	8.70%	Deleterious	PASSENGER/OTHER	Passenger	passenger	.	.	0.38	0.06583	15%	Subclonal	.	.	.	.	WES		
IDC4T	*BDNF*	p.V208I	Missense_Mutation	11	27679736	C	T	180	43.89%	Deleterious	PASSENGER/OTHER	Passenger	passenger	.	.	1	0.8659	94%	Clonal	.	.	.	.	WES		
IDC4T	*BIVM-ERCC5*	p.S103C	Missense_Mutation	13	103460009	C	G	101	18.81%	Deleterious	.	Passenger	passenger	.	loh	0.64	0.10018	41%	Subclonal	.	.	.	.	WES		
IDC4T	*C1orf131*	p.E43K	Missense_Mutation	1	231374926	C	T	71	19.72%	Neutral	PASSENGER/OTHER	Passenger	passenger	.	.	1	0.8578	65%	Clonal	.	.	.	.	WES		
IDC4T	*C1orf228*	p.H431Y	Missense_Mutation	1	45191043	C	T	166	40.96%	Neutral	.	Passenger	passenger	.	.	1	0.95372	93%	Clonal	.	.	.	.	WES		
IDC4T	*C1orf51*	p.E368Q	Missense_Mutation	1	150259310	G	C	168	29.76%	Neutral	.	Passenger	passenger	.	.	1	0.82873	87%	Clonal	.	.	.	.	WES		
IDC4T	*CABP4*	p.R54H	Missense_Mutation	11	67223055	G	A	81	7.41%	Neutral	PASSENGER/OTHER	Passenger	passenger	.	.	0.4	0.03083	18%	Subclonal	.	.	.	.	WES		
IDC4T	*CHPF*	p.S682T	Missense_Mutation	2	220404389	A	T	57	21.05%	Deleterious	PASSENGER/OTHER	Passenger	passenger	.	loh	0.71	0.39566	41%	Subclonal	.	.	.	.	WES		
IDC4T	*CHST11*	p.R219C	Missense_Mutation	12	105151177	C	T	212	44.81%	Deleterious	PASSENGER/OTHER	Passenger	passenger	.	.	1	0.74348	95%	Clonal	.	.	.	.	WES		
IDC4T	*CLP1*	p.Q259E	Missense_Mutation	11	57428405	C	G	195	5.13%	Deleterious	PASSENGER/OTHER	Passenger	passenger	.	.	0.28	0	15%	Subclonal	.	.	.	.	WES		
IDC4T	*CNGA3*	p.A619V	Missense_Mutation	2	99013489	C	T	86	26.74%	Neutral	PASSENGER/OTHER	Passenger	passenger	.	.	1	0.84515	70%	Clonal	.	.	.	.	WES		
IDC4T	*CNTROB*	p.Q545H	Missense_Mutation	17	7847857	G	C	46	76.09%	Deleterious	PASSENGER/OTHER	Passenger	passenger	.	loh	1	0.20854	89%	Subclonal	.	.	.	.	WES		
IDC4T	*COL6A5*	p.L1009V	Missense_Mutation	3	130113765	C	G	33	12.12%	Neutral	PASSENGER/OTHER	Passenger	passenger	.	loh	0.41	0.08755	16%	Subclonal	.	.	.	.	WES		
IDC4T	*CSGALNACT1*	p.S503F	Missense_Mutation	8	19263382	G	A	118	10.17%	Deleterious	PASSENGER/OTHER	Passenger	passenger	.	loh	0.34	0.00001	20%	Subclonal	.	.	.	.	WES		
IDC4T	*CTNNA2*	p.N351H	Missense_Mutation	2	80136918	A	C	38	15.79%	Deleterious	PASSENGER/OTHER	Passenger	passenger	.	.	0.85	0.59412	36%	Clonal	.	.	.	.	WES		
IDC4T	*CTNNBL1*	p.S524R	Missense_Mutation	20	36488715	C	A	124	10.48%	Deleterious	PASSENGER/OTHER	Passenger	passenger	.	.	0.56	0.08776	33%	Subclonal	.	.	.	.	WES		
IDC4T	*DDX19A*	p.V411M	Missense_Mutation	16	70405322	G	A	34	8.82%	Deleterious	PASSENGER/OTHER	Passenger	passenger	.	loh	0.3	0.02336	10%	Subclonal	.	.	.	.	WES		
IDC4T	*DMD*	p.E1384V	Missense_Mutation	X	32429951	T	A	108	37.04%	Deleterious	PASSENGER/OTHER	Driver	likely_pathogenic	.	.	1	0.96539	87%	Clonal	.	.	.	.	WES		
IDC4T	*DPRX*	p.T24S	Missense_Mutation	19	54137827	C	G	34	29.41%	Neutral	PASSENGER/OTHER	Passenger	passenger	.	.	0.99	0.71714	51%	Clonal	.	.	.	.	WES		
IDC4T	*EIF4EBP2*	p.N97Y	Missense_Mutation	10	72179813	A	T	73	9.59%	Deleterious	.	Passenger	passenger	.	loh	0.32	0.00049	16%	Subclonal	.	.	.	.	WES		
IDC4T	*EIF4EBP2*	p.N96H	Missense_Mutation	10	72179810	A	C	75	10.67%	Deleterious	.	Passenger	passenger	.	loh	0.36	0.00107	18%	Subclonal	.	.	.	.	WES		
IDC4T	*EVX2*	p.R323C	Missense_Mutation	2	176945299	G	A	33	9.09%	Deleterious	PASSENGER/OTHER	Passenger	passenger	.	.	0.4	0.14637	14%	Subclonal	.	.	.	.	WES		
IDC4T	*FAM135B*	p.Q1295E	Missense_Mutation	8	139151247	G	C	157	5.10%	Deleterious	PASSENGER/OTHER	Passenger	passenger	.	.	0.53	0.15799	27%	Subclonal	.	.	.	.	WES		
IDC4T	*FAM160A1*	p.E603K	Missense_Mutation	4	152571000	G	A	169	17.75%	Neutral	PASSENGER/OTHER	Passenger	passenger	.	.	0.78	0.41036	55%	Subclonal	.	.	.	.	WES		
IDC4T	*FAM47B*	p.R224H	Missense_Mutation	X	34961619	G	A	170	33.53%	Neutral	PASSENGER/OTHER	Passenger	passenger	.	.	1	0.95605	90%	Clonal	.	.	.	.	WES		
IDC4T	*FBLN2*	p.G1008V	Missense_Mutation	3	13672253	G	T	70	18.57%	Deleterious	PASSENGER/OTHER	Passenger	passenger	.	.	1	0.79094	59%	Clonal	.	.	.	.	WES		
IDC4T	*GCAT*	p.S321C	Missense_Mutation	22	38211739	C	G	152	19.74%	Deleterious	PASSENGER/OTHER	Passenger	passenger	.	.	0.86	0.64212	61%	Clonal	.	.	.	.	WES		
IDC4T	*GRIA2*	p.D477E	Missense_Mutation	4	158256987	C	A	74	13.51%	Deleterious	PASSENGER/OTHER	Passenger	passenger	.	loh	0.46	0.01125	25%	Subclonal	.	.	.	.	WES		
IDC4T	*JMJD1C*	p.Q436E	Missense_Mutation	10	64974621	G	C	103	34.95%	Neutral	PASSENGER/OTHER	Passenger	passenger	.	.	1	0.96106	85%	Clonal	.	.	.	.	WES		
IDC4T	*JMJD1C*	p.S501Y	Missense_Mutation	10	64974425	G	T	139	35.97%	Neutral	PASSENGER/OTHER	Passenger	passenger	.	.	1	0.96817	89%	Clonal	.	.	.	.	WES		
IDC4T	*LARGE*	p.R398G	Missense_Mutation	22	33733727	G	C	181	4.97%	Deleterious	PASSENGER/OTHER	Passenger	passenger	.	.	0.22	0	11%	Subclonal	.	.	.	.	WES		
IDC4T	*LRP1B*	p.R3026S	Missense_Mutation	2	141250219	C	G	70	7.14%	Deleterious	PASSENGER/OTHER	Passenger	passenger	.	.	0.31	0.00456	13%	Subclonal	.	.	.	.	WES		
IDC4T	*MAPK8IP3*	p.V371L	Missense_Mutation	16	1798619	G	T	136	16.91%	Deleterious	PASSENGER/OTHER	Passenger	passenger	.	.	0.91	0.70339	59%	Clonal	.	.	.	.	WES		
IDC4T	*MEAF6*	p.R171Q	Missense_Mutation	1	37967426	C	T	42	9.52%	Deleterious	.	Passenger	passenger	.	.	0.51	0.2666	20%	Subclonal	.	.	.	.	WES		
IDC4T	*MTNR1B*	p.E111Q	Missense_Mutation	11	92714720	G	C	171	23.39%	Neutral	PASSENGER/OTHER	Passenger	passenger	.	.	1	0.83913	73%	Clonal	.	.	.	.	WES		
IDC4T	*NBEAL1*	p.K896N	Missense_Mutation	2	203990167	G	C	102	32.35%	Neutral	PASSENGER/OTHER	Passenger	passenger	.	loh	1	0.86165	74%	Clonal	.	.	.	.	WES		
IDC4T	*NLRP2*	p.L842P	Missense_Mutation	19	55501548	T	C	135	40.74%	Deleterious	PASSENGER/OTHER	Passenger	passenger	.	.	1	0.67549	87%	Clonal	.	.	.	.	WES		
IDC4T	*NPC1*	p.Y423N	Missense_Mutation	18	21136266	A	T	180	40.00%	Deleterious	PASSENGER/OTHER	Passenger	passenger	.	.	1	0.9633	93%	Clonal	.	.	.	.	WES		
IDC4T	*OR2T3*	p.A48V	Missense_Mutation	1	248636794	C	T	32	50.00%	Neutral	PASSENGER/OTHER	Passenger	passenger	.	.	1	0.35414	78%	Subclonal	.	.	.	.	WES		
IDC4T	*P2RY2*	p.L112R	Missense_Mutation	11	72945539	T	G	132	7.58%	Deleterious	PASSENGER/OTHER	Passenger	passenger	.	.	0.41	0.00372	22%	Subclonal	.	.	.	.	WES		
IDC4T	*PCDHGB6*	p.M291K	Missense_Mutation	5	140788641	T	A	112	29.46%	Neutral	PASSENGER/OTHER	Passenger	passenger	.	.	0.99	0.81282	70%	Clonal	.	.	.	.	WES		
IDC33T	*PIK3CA*	p.R93W	Missense_Mutation	3	178916890	C	T	230	36.09%	Deleterious	PASSENGER/OTHER	Driver	likely_pathogenic	.	.	1	0.9037	85%	Clonal	TRUE	TRUE	TRUE	YES	MSK-IMPACT	AAGAAGCAAGAAAATACCCCCT	ACCAATTTCTCGATTGAGGATCT
IDC4T	*PLCB2*	p.N255K	Missense_Mutation	15	40591084	G	C	95	4.21%	Deleterious	PASSENGER/OTHER	Passenger	passenger	.	.	0.23	0.00039	9%	Subclonal	.	.	.	.	WES		
IDC4T	*PRPF8*	p.R1384W	Missense_Mutation	17	1564957	G	A	78	5.13%	Deleterious	PASSENGER/OTHER	Passenger	passenger	.	loh	0.22	0.00025	9%	Subclonal	.	.	.	.	WES		
IDC4T	*SEMA6D*	p.S957Y	Missense_Mutation	15	48063630	C	A	168	5.36%	Deleterious	PASSENGER/OTHER	Passenger	passenger	.	.	0.29	0.00001	15%	Subclonal	.	.	.	.	WES		
IDC4T	*SERINC4*	p.P284T	Missense_Mutation	15	44089133	G	T	102	19.61%	Deleterious	PASSENGER/OTHER	Passenger	passenger	.	.	1	0.79366	63%	Clonal	.	.	.	.	WES		
IDC4T	*SLC27A3*	p.G592A	Missense_Mutation	1	153751349	G	C	174	20.11%	Deleterious	PASSENGER/OTHER	Passenger	passenger	.	.	1	0.85083	73%	Clonal	.	.	.	.	WES		
IDC4T	*SLC38A10*	p.E999K	Missense_Mutation	17	79219721	C	T	128	41.41%	Neutral	PASSENGER/OTHER	Passenger	passenger	.	.	1	0.94856	91%	Clonal	.	.	.	.	WES		
IDC4T	*SLC39A12*	p.T67A	Missense_Mutation	10	18242404	A	G	93	15.05%	Neutral	PASSENGER/OTHER	Passenger	passenger	.	.	0.81	0.57011	48%	Clonal	.	.	.	.	WES		
IDC4T	*SUSD5*	p.Q247E	Missense_Mutation	3	33195385	G	C	183	10.93%	Neutral	PASSENGER/OTHER	Passenger	passenger	.	.	0.48	0.00054	31%	Subclonal	.	.	.	.	WES		
IDC4T	*SZT2*	p.I2934M	Missense_Mutation	1	43911906	C	G	105	26.67%	Neutral	.	Passenger	passenger	.	.	1	0.85083	78%	Clonal	.	.	.	.	WES		
IDC4T	*TAS1R3*	p.A694V	Missense_Mutation	1	1269366	C	T	49	22.45%	Neutral	PASSENGER/OTHER	Passenger	passenger	.	loh	0.76	0.49674	43%	Subclonal	.	.	.	.	WES		
IDC4T	*TFDP3*	p.T343K	Missense_Mutation	X	132351260	G	T	128	53.91%	Neutral	PASSENGER/OTHER	Passenger	passenger	.	.	1	0.86601	93%	Clonal	.	.	.	.	WES		
IDC4T	*TIMELESS*	p.R434H	Missense_Mutation	12	56822670	C	T	44	9.09%	Deleterious	PASSENGER/OTHER	Passenger	passenger	.	.	0.4	0.08648	16%	Subclonal	.	.	.	.	WES		
IDC4T	*TMEM229A*	p.Q236L	Missense_Mutation	7	123672351	T	A	43	32.56%	Neutral	.	Passenger	passenger	.	loh	1	0.79054	60%	Clonal	.	.	.	.	WES		
IDC4T	*UBR1*	p.E1697K	Missense_Mutation	15	43242479	C	T	71	23.94%	Deleterious	PASSENGER/OTHER	Passenger	passenger	.	.	1	0.83446	67%	Clonal	.	.	.	.	WES		
IDC4T	*WEE2*	p.P157H	Missense_Mutation	7	141414136	C	A	45	31.11%	Deleterious	PASSENGER/OTHER	Passenger	passenger	.	loh	1	0.77304	59%	Clonal	.	.	.	.	WES		
IDC4T	*WLS*	p.I405S	Missense_Mutation	1	68611610	A	C	163	23.31%	Deleterious	PASSENGER/OTHER	Passenger	passenger	.	.	1	0.87934	79%	Clonal	.	.	.	.	WES		
IDC4T	*ZC3H18*	p.D107H	Missense_Mutation	16	88643850	G	C	78	73.08%	Deleterious	PASSENGER/OTHER	Passenger	passenger	.	loh	1	0.61545	93%	Clonal	.	.	.	.	WES		
IDC4T	*ZFP69*	p.E515K	Missense_Mutation	1	40961693	G	A	127	9.45%	Neutral	PASSENGER/OTHER	Passenger	passenger	.	.	0.51	0.03497	29%	Subclonal	.	.	.	.	WES		
IDC4T	*ZFPM1*	p.R337W	Missense_Mutation	16	88599084	C	T	63	6.35%	Deleterious	PASSENGER/OTHER	Passenger	passenger	.	loh	0.21	0.00007	8%	Subclonal	.	.	.	.	WES		
IDC4T	*ZMYND12*	p.E346Q	Missense_Mutation	1	42896469	C	G	150	16.67%	Neutral	PASSENGER/OTHER	Passenger	passenger	.	.	0.9	0.69071	60%	Clonal	.	.	.	.	WES		
IDC4T	*GANAB*	p.Q692*	Nonsense_Mutation	11	62396413	G	A	103	4.85%	Deleterious	.	.	passenger	.	.	0.26	0.00051	11%	Subclonal	.	.	.	.	WES		
IDC4T	*SLC4A2*	p.S856*	Nonsense_Mutation	7	150771157	C	G	14	21.43%	Deleterious	.	.	likely_pathogenic	.	loh	0.72	0.50221	23%	Clonal	.	.	.	.	WES		
IDC4T	*TCF20*	p.E1394*	Nonsense_Mutation	22	42607132	C	A	218	26.61%	Deleterious	.	.	passenger	.	.	1	0.90539	82%	Clonal	.	.	.	.	WES		
IDC4T	*WDR90*	p.S886*	Nonsense_Mutation	16	708235	C	G	141	3.55%	Deleterious	.	.	passenger	.	.	0.19	0	8%	Subclonal	.	.	.	.	WES		
IDC4T	*TULP3*	p.X309_splice	Splice_Site	12	3046796	G	A	43	18.60%	Deleterious	.	.	passenger	.	.	0.81	0.57819	40%	Clonal	.	.	.	.	WES		
IDC50T	*ALMS1*	p.M2669Wfs*8	Frame_Shift_Del	2	73717091	CGAATGCCATTCGATGAAAA	C	46	6.52%	.	.	.	passenger	.	.	0.27	0.01479	9%	Subclonal	.	.	.	.	WES		
IDC50T	*CTSB*	p.N16Gfs*33	Frame_Shift_Del	8	11710906	TGCTCCGGGCATTG	T	34	23.53%	.	.	.	likely_pathogenic	.	loh	0.97	0.71956	45%	Clonal	.	.	.	.	WES		
IDC50T	*PALB2*	p.Q921Rfs*2	Frame_Shift_Del	16	23635401	CT	C	53	32.08%	.	.	.	likely_pathogenic	.	loh	1	0.91575	72%	Clonal	TRUE	.	.	YES	WES	GCTGACAGAGACAAAGATGAAGG	TCCTTGTACAGTGAGAATACAAAAGA
IDC50T	*TIFA*	p.Q140Lfs*68	Frame_Shift_Del	4	113199150	AAATT	A	59	28.81%	.	.	.	passenger	.	.	1	0.88037	70%	Clonal	.	.	.	.	WES		
IDC50T	*BMP2K*	p.A315Cfs*3	Frame_Shift_Ins	4	79780792	A	AT	52	21.15%	.	.	.	passenger	.	.	1	0.78283	54%	Clonal	.	.	.	.	WES		
IDC50T	*PRKCG*	p.C586Yfs*19	Frame_Shift_Ins	19	54407984	C	CATCT	256	13.67%	.	.	.	likely_pathogenic	.	loh	0.7	0.13987	51%	Subclonal	.	.	.	.	WES		
IDC50T	*PVRL2*	p.R258Pfs*5	Frame_Shift_Ins	19	45375402	A	AC	92	26.09%	.	.	.	likely_pathogenic	.	loh	0.81	0.58159	56%	Clonal	.	.	.	.	WES		
IDC50T	*MICAL3*	p.E1000del	In_Frame_Del	22	18314678	TTCC	T	33	15.15%	.	.	.	passenger	.	loh	0.62	0.42663	26%	Subclonal	.	.	.	.	WES		
IDC50T	*MARCH4*	p.Q363K	Missense_Mutation	2	217124181	G	T	209	16.75%	Neutral	PASSENGER/OTHER	Passenger	passenger	.	.	0.86	0.66521	62%	Clonal	.	.	.	.	WES		
IDC50T	*ABCA7*	p.S262N	Missense_Mutation	19	1043245	G	A	164	18.90%	Neutral	PASSENGER/OTHER	Passenger	passenger	.	.	0.78	0.44858	56%	Subclonal	.	.	.	.	WES		
IDC50T	*ABCC9*	p.H69Q	Missense_Mutation	12	22086793	A	T	51	13.73%	Deleterious	PASSENGER/OTHER	Passenger	passenger	.	.	0.7	0.50467	33%	Clonal	.	.	.	.	WES		
IDC50T	*ANK3*	p.E4281G	Missense_Mutation	10	61815639	T	C	33	24.24%	Deleterious	CANCER	Passenger	likely_pathogenic	.	.	0.76	0.55745	38%	Clonal	.	.	.	.	WES		
IDC50T	*ARRDC4*	p.D167N	Missense_Mutation	15	98509249	G	A	27	11.11%	Deleterious	PASSENGER/OTHER	Passenger	passenger	.	.	0.35	0.07237	12%	Subclonal	.	.	.	.	WES		
IDC50T	*ASIC2*	p.E388K	Missense_Mutation	17	31352977	C	T	67	58.21%	Deleterious	PASSENGER/OTHER	Passenger	passenger	.	.	1	0.88355	89%	Clonal	.	.	.	.	WES		
IDC50T	*ATF7IP*	p.A949V	Missense_Mutation	12	14619508	C	T	28	67.86%	Deleterious	PASSENGER/OTHER	Driver	likely_pathogenic	.	.	1	0.3846	82%	Subclonal	.	.	.	.	WES		
IDC50T	*ATP5B*	p.Q469E	Missense_Mutation	12	57032974	G	C	37	16.22%	Deleterious	PASSENGER/OTHER	Driver	likely_pathogenic	.	loh	0.83	0.62558	36%	Clonal	.	.	.	.	WES		
IDC50T	*B3GNT9*	p.C219R	Missense_Mutation	16	67183734	A	G	129	26.36%	Deleterious	PASSENGER/OTHER	Passenger	passenger	.	loh	1	0.87656	73%	Clonal	.	.	.	.	WES		
IDC50T	*BACH1*	p.L218V	Missense_Mutation	21	30698797	C	G	29	68.97%	Neutral	PASSENGER/OTHER	Passenger	passenger	.	loh	1	0.95571	81%	Clonal	.	.	.	.	WES		
IDC50T	*BAIAP2L2*	p.L194R	Missense_Mutation	22	38493070	A	C	27	66.67%	Deleterious	CANCER	Passenger	likely_pathogenic	.	loh	1	0.78015	80%	Clonal	.	.	.	.	WES		
IDC50T	*BRICD5*	p.S5T	Missense_Mutation	16	2260821	C	G	147	40.14%	Neutral	PASSENGER/OTHER	Passenger	passenger	.	.	1	0.96805	91%	Clonal	.	.	.	.	WES		
IDC50T	*C15orf27*	p.E152Q	Missense_Mutation	15	76452507	G	C	44	27.27%	Deleterious	PASSENGER/OTHER	Passenger	passenger	.	.	0.85	0.66504	49%	Clonal	.	.	.	.	WES		
IDC50T	*C1orf65*	p.A173G	Missense_Mutation	1	223567335	C	G	69	15.94%	Deleterious	PASSENGER/OTHER	Passenger	passenger	.	.	0.82	0.62509	45%	Clonal	.	.	.	.	WES		
IDC50T	*CD177*	p.R90W	Missense_Mutation	19	43858433	C	T	41	7.32%	Neutral	PASSENGER/OTHER	Passenger	passenger	.	loh	0.23	0.00189	8%	Subclonal	.	.	.	.	WES		
IDC50T	*CDCP1*	p.Q94H	Missense_Mutation	3	45159914	C	G	55	30.91%	Neutral	PASSENGER/OTHER	Passenger	passenger	.	.	1	0.90309	72%	Clonal	.	.	.	.	WES		
IDC50T	*CDH15*	p.A4P	Missense_Mutation	16	89238249	G	C	55	10.91%	Neutral	PASSENGER/OTHER	Passenger	passenger	.	loh	0.34	0.0032	16%	Subclonal	.	.	.	.	WES		
IDC50T	*CDK5RAP2*	p.S391R	Missense_Mutation	9	123280843	A	C	144	5.56%	Neutral	PASSENGER/OTHER	Passenger	passenger	.	.	0.28	0.00003	14%	Subclonal	.	.	.	.	WES		
IDC50T	*CEP350*	p.T2685I	Missense_Mutation	1	180063294	C	T	43	13.95%	Neutral	PASSENGER/OTHER	Passenger	passenger	.	.	0.71	0.52767	32%	Clonal	.	.	.	.	WES		
IDC50T	*CILP*	p.Q1123H	Missense_Mutation	15	65489255	T	A	60	45.00%	Deleterious	PASSENGER/OTHER	Passenger	passenger	.	.	1	0.85089	79%	Clonal	.	.	.	.	WES		
IDC50T	*CIPC*	p.A82T	Missense_Mutation	14	77576262	G	A	45	28.89%	Neutral	PASSENGER/OTHER	Passenger	passenger	.	loh	0.9	0.71587	52%	Clonal	.	.	.	.	WES		
IDC50T	*CLNK*	p.R206M	Missense_Mutation	4	10533893	C	A	33	36.36%	Neutral	PASSENGER/OTHER	Passenger	passenger	.	.	1	0.9281	67%	Clonal	.	.	.	.	WES		
IDC50T	*CRISPLD2*	p.S437A	Missense_Mutation	16	84922839	T	G	15	53.33%	Deleterious	PASSENGER/OTHER	Passenger	passenger	.	loh	1	0.89275	59%	Clonal	.	.	.	.	WES		
IDC50T	*CROCC*	p.D1168E	Missense_Mutation	1	17281845	T	A	21	52.38%	Neutral	PASSENGER/OTHER	Passenger	passenger	.	.	1	0.93209	68%	Clonal	.	.	.	.	WES		
IDC50T	*CSNK2A3*	p.E114Q	Missense_Mutation	11	11374327	C	G	82	25.61%	.	PASSENGER/OTHER	Passenger	passenger	.	.	1	0.83133	65%	Clonal	.	.	.	.	WES		
IDC50T	*CTNNA2*	p.N351H	Missense_Mutation	2	80136918	A	C	29	20.69%	Deleterious	PASSENGER/OTHER	Passenger	passenger	.	.	0.85	0.64096	37%	Clonal	.	.	.	.	WES		
IDC50T	*DIO2*	p.R48S	Missense_Mutation	14	80677674	G	T	103	31.07%	Deleterious	PASSENGER/OTHER	Passenger	passenger	.	loh	0.97	0.82076	68%	Clonal	.	.	.	.	WES		
IDC50T	*DISP1*	p.P1314L	Missense_Mutation	1	223178680	C	T	182	4.95%	Neutral	PASSENGER/OTHER	Passenger	passenger	.	.	0.25	0	13%	Subclonal	.	.	.	.	WES		
IDC50T	*DNM2*	p.R199W	Missense_Mutation	19	10887799	C	T	44	9.09%	Deleterious	PASSENGER/OTHER	Passenger	passenger	.	.	0.37	0.06744	15%	Subclonal	TRUE	.	.	.	WES		
IDC50T	*DOCK2*	p.R1800Q	Missense_Mutation	5	169508957	G	A	30	10.00%	Neutral	PASSENGER/OTHER	Passenger	passenger	.	.	0.31	0.03607	11%	Subclonal	.	.	.	.	WES		
IDC50T	*DPY19L3*	p.E560Q	Missense_Mutation	19	32959700	G	C	38	7.89%	Deleterious	PASSENGER/OTHER	Passenger	passenger	.	.	0.4	0.18852	14%	Subclonal	.	.	.	.	WES		
IDC50T	*EML2*	p.G492S	Missense_Mutation	19	46124866	C	T	40	32.50%	Deleterious	PASSENGER/OTHER	Passenger	passenger	.	loh	1	0.78537	56%	Clonal	.	.	.	.	WES		
IDC50T	*EML6*	p.S379C	Missense_Mutation	2	55074709	C	G	62	20.97%	Deleterious	PASSENGER/OTHER	Passenger	passenger	.	.	0.86	0.67751	50%	Clonal	.	.	.	.	WES		
IDC50T	*FANCM*	p.P1670L	Missense_Mutation	14	45658234	C	T	24	16.67%	Deleterious	PASSENGER/OTHER	Passenger	passenger	.	.	0.85	0.62419	30%	Clonal	.	.	.	.	WES		
IDC50T	*FDFT1*	p.E191Q	Missense_Mutation	8	11683593	G	C	24	20.83%	Deleterious	PASSENGER/OTHER	Passenger	passenger	.	loh	0.86	0.63668	34%	Clonal	.	.	.	.	WES		
IDC50T	*FKBPL*	p.H180R	Missense_Mutation	6	32097019	T	C	283	22.97%	Neutral	PASSENGER/OTHER	Passenger	passenger	.	.	1	0.92099	84%	Clonal	.	.	.	.	WES		
IDC50T	*FLNC*	p.E25G	Missense_Mutation	7	128470765	A	G	101	15.84%	Deleterious	PASSENGER/OTHER	Passenger	passenger	.	.	0.97	0.76345	56%	Clonal	.	.	.	.	WES		
IDC50T	*FRAS1*	p.Q1429H	Missense_Mutation	4	79328974	G	C	25	12.00%	Deleterious	PASSENGER/OTHER	Passenger	passenger	.	.	0.61	0.46895	20%	Subclonal	.	.	.	.	WES		
IDC50T	*FREM1*	p.E170Q	Missense_Mutation	9	14859304	C	G	89	41.57%	Deleterious	PASSENGER/OTHER	Passenger	passenger	.	.	1	0.88905	81%	Clonal	.	.	.	.	WES		
IDC50T	*GGT7*	p.S440T	Missense_Mutation	20	33442334	C	G	111	15.32%	Deleterious	PASSENGER/OTHER	Passenger	passenger	.	.	0.94	0.74707	56%	Clonal	.	.	.	.	WES		
IDC50T	*GLDC*	p.G674R	Missense_Mutation	9	6558591	C	T	7	42.86%	Deleterious	.	Passenger	passenger	.	.	1	0.81921	35%	Clonal	.	.	.	.	WES		
IDC50T	*GOLGB1*	p.K1832N	Missense_Mutation	3	121413874	C	A	42	21.43%	Neutral	PASSENGER/OTHER	Passenger	passenger	.	.	0.88	0.67904	44%	Clonal	.	.	.	.	WES		
IDC50T	*GRIP1*	p.D115H	Missense_Mutation	12	66932933	C	G	44	20.45%	Deleterious	PASSENGER/OTHER	Passenger	passenger	.	.	1	0.8058	53%	Clonal	.	.	.	.	WES		
IDC50T	*GZF1*	p.K68N	Missense_Mutation	20	23345224	G	C	119	9.24%	Neutral	PASSENGER/OTHER	Passenger	passenger	.	.	0.57	0.1573	32%	Subclonal	.	.	.	.	WES		
IDC50T	*HK1*	p.L503M	Missense_Mutation	10	71142472	C	A	54	22.22%	Deleterious	PASSENGER/OTHER	Passenger	passenger	.	.	0.92	0.71695	50%	Clonal	.	.	.	.	WES		
IDC50T	*HMHA1*	p.C763F	Missense_Mutation	19	1081598	G	T	68	19.12%	Deleterious	PASSENGER/OTHER	Passenger	passenger	.	.	0.79	0.58278	46%	Clonal	.	.	.	.	WES		
IDC50T	*HSPB1*	p.Q31K	Missense_Mutation	7	75932120	C	A	99	17.17%	Deleterious	PASSENGER/OTHER	Passenger	passenger	.	.	1	0.80531	60%	Clonal	.	.	.	.	WES		
IDC50T	*HTR2A*	p.V375G	Missense_Mutation	13	47409264	A	C	44	65.91%	Deleterious	PASSENGER/OTHER	Passenger	passenger	.	loh	1	0.96673	86%	Clonal	.	.	.	.	WES		
IDC50T	*HTR2C*	p.S380C	Missense_Mutation	X	114141740	C	G	30	40.00%	Deleterious	PASSENGER/OTHER	Passenger	passenger	.	loh	1	0.83767	62%	Clonal	.	.	.	.	WES		
IDC50T	*IGFN1*	p.L2545Q	Missense_Mutation	1	201181655	T	A	84	5.95%	Neutral	PASSENGER/OTHER	Passenger	passenger	.	.	0.3	0.00476	13%	Subclonal	.	.	.	.	WES		
IDC50T	*JAG1*	p.A891P	Missense_Mutation	20	10622442	C	G	154	12.34%	Deleterious	PASSENGER/OTHER	Passenger	passenger	.	.	0.76	0.49153	48%	Subclonal	.	.	.	.	WES		
IDC50T	*KALRN*	p.A1659V	Missense_Mutation	3	124281736	C	T	64	48.44%	Deleterious	CANCER	Passenger	likely_pathogenic	.	.	1	0.96231	85%	Clonal	.	.	.	.	WES		
IDC50T	*KCNH6*	p.P2S	Missense_Mutation	17	61600778	C	T	104	13.46%	Deleterious	PASSENGER/OTHER	Passenger	passenger	.	.	0.82	0.63326	48%	Clonal	.	.	.	.	WES		
IDC50T	*KCNV2*	p.T314M	Missense_Mutation	9	2718680	C	T	456	3.07%	Deleterious	PASSENGER/OTHER	Passenger	passenger	.	.	0.16	0	9%	Subclonal	.	.	.	.	WES		
IDC50T	*KIAA0368*	p.G385R	Missense_Mutation	9	114199309	C	T	77	20.78%	Deleterious	PASSENGER/OTHER	Passenger	passenger	.	.	1	0.80807	60%	Clonal	.	.	.	.	WES		
IDC50T	*KIAA1875*	p.S1241N	Missense_Mutation	8	145169308	G	A	365	42.74%	Neutral	PASSENGER/OTHER	Passenger	passenger	.	.	1	0.10973	97%	Clonal	.	.	.	.	WES		
IDC50T	*KMT2E*	p.Y884C	Missense_Mutation	7	104747023	A	G	95	20.00%	Deleterious	PASSENGER/OTHER	Passenger	passenger	.	.	1	0.80642	62%	Clonal	.	.	.	.	WES		
IDC50T	*KPNA6*	p.E87D	Missense_Mutation	1	32622976	G	C	15	40.00%	Deleterious	PASSENGER/OTHER	Passenger	passenger	.	.	1	0.8584	50%	Clonal	.	.	.	.	WES		
IDC50T	*KSR1*	p.C539Y	Missense_Mutation	17	25932806	G	A	127	13.39%	Deleterious	CANCER	Passenger	likely_pathogenic	.	.	0.82	0.62388	51%	Clonal	.	.	.	.	WES		
IDC50T	*LIN28A*	p.A20V	Missense_Mutation	1	26737904	C	T	29	10.34%	Neutral	PASSENGER/OTHER	Passenger	passenger	.	.	0.43	0.21396	15%	Subclonal	.	.	.	.	WES		
IDC50T	*LMTK2*	p.V1364G	Missense_Mutation	7	97823868	T	G	87	14.94%	Deleterious	CANCER	Passenger	likely_pathogenic	.	.	0.76	0.55015	44%	Clonal	.	.	.	.	WES		
IDC50T	*MAGEB3*	p.M286L	Missense_Mutation	X	30254897	A	T	53	15.09%	Neutral	PASSENGER/OTHER	Passenger	passenger	.	.	0.62	0.34205	32%	Subclonal	.	.	.	.	WES		
IDC50T	*MDGA2*	p.W454C	Missense_Mutation	14	47351407	C	A	47	8.51%	Deleterious	PASSENGER/OTHER	Passenger	passenger	.	.	0.44	0.17057	17%	Subclonal	.	.	.	.	WES		
IDC50T	*MDGA2*	p.W454L	Missense_Mutation	14	47351408	C	A	48	10.42%	Deleterious	PASSENGER/OTHER	Passenger	passenger	.	.	0.53	0.28963	23%	Subclonal	.	.	.	.	WES		
IDC50T	*MMP11*	p.A160D	Missense_Mutation	22	24122686	C	A	162	27.78%	Neutral	PASSENGER/OTHER	Passenger	passenger	.	loh	1	0.90609	79%	Clonal	.	.	.	.	WES		
IDC50T	*MMP8*	p.N170S	Missense_Mutation	11	102592245	T	C	44	27.27%	Deleterious	PASSENGER/OTHER	Passenger	passenger	.	.	0.85	0.66504	49%	Clonal	.	.	.	.	WES		
IDC50T	*MYO18B*	p.V340L	Missense_Mutation	22	26164901	G	T	69	13.04%	Neutral	PASSENGER/OTHER	Passenger	passenger	.	loh	0.54	0.13277	29%	Subclonal	.	.	.	.	WES		
IDC50T	*MYOM3*	p.S39N	Missense_Mutation	1	24435011	C	T	41	48.78%	Neutral	PASSENGER/OTHER	Passenger	passenger	.	.	1	0.95347	79%	Clonal	.	.	.	.	WES		
IDC50T	*NAA11*	p.E200K	Missense_Mutation	4	80246434	C	T	172	4.07%	Neutral	PASSENGER/OTHER	Passenger	passenger	.	.	0.21	0	10%	Subclonal	.	.	.	.	WES		
IDC50T	*NAA20*	p.L92V	Missense_Mutation	20	20007532	C	G	107	17.76%	Deleterious	PASSENGER/OTHER	Passenger	passenger	.	.	1	0.82618	63%	Clonal	.	.	.	.	WES		
IDC50T	*NAF1*	p.Y396C	Missense_Mutation	4	164050347	T	C	48	31.25%	Neutral	PASSENGER/OTHER	Passenger	passenger	.	loh	0.97	0.77645	57%	Clonal	.	.	.	.	WES		

IDC50T	NASP	p.A239V	Missense_Mutation	1	46073299	C	T	28	10.71%	Neutral	PASSENGER/OTHER	Passenger	passenger	.	.	0.44	0.23926	15%	Subclonal	.	.	.	.	WES		
IDC50T	NCK2	p.M222T	Missense_Mutation	2	106498222	T	C	90	27.78%	Deleterious	PASSENGER/OTHER	Passenger	passenger	.	.	1	0.86923	71%	Clonal	.	.	.	.	WES		
IDC50T	NPHP1	p.Q345K	Missense_Mutation	2	110919269	G	T	27	22.22%	Deleterious	PASSENGER/OTHER	Passenger	passenger	.	.	0.92	0.67529	39%	Clonal	.	.	.	.	WES		
IDC50T	NR2C2	p.S171T	Missense_Mutation	3	15062338	G	C	16	18.75%	Deleterious	PASSENGER/OTHER	Passenger	passenger	.	.	0.77	0.57646	24%	Clonal	.	.	.	.	WES		
IDC50T	NUDT3		Missense_Mutation	6	34256514	TACAGGAAGTCTTCAGTCATCTGATGCCTG	T	42	11.90%	.	.	.	passenger	.	.	0.61	0.41284	26%	Subclonal	.	.	.	.	WES		
IDC50T	OIT3	p.G522R	Missense_Mutation	10	74692208	G	A	223	21.52%	Neutral	PASSENGER/OTHER	Passenger	passenger	.	.	0.89	0.71598	67%	Clonal	.	.	.	.	WES		
IDC50T	PALD1	p.T732S	Missense_Mutation	10	72307134	A	T	77	20.78%	Neutral	PASSENGER/OTHER	Passenger	passenger	.	.	0.86	0.67307	52%	Clonal	.	.	.	.	WES		
IDC50T	PANK2	p.S244I	Missense_Mutation	20	3888675	G	T	67	20.90%	Deleterious	PASSENGER/OTHER	Passenger	passenger	.	.	1	0.79897	58%	Clonal	.	.	.	.	WES		
IDC50T	PCDHAC2	p.S297R	Missense_Mutation	5	140347242	C	A	112	35.71%	Deleterious	PASSENGER/OTHER	Passenger	passenger	.	.	1	0.95553	86%	Clonal	.	.	.	.	WES		
IDC50T	PDIA2	p.G113A	Missense_Mutation	16	334525	G	C	128	49.22%	Neutral	PASSENGER/OTHER	Passenger	passenger	.	.	1	0.67162	93%	Clonal	.	.	.	.	WES		
IDC50T	PGAP1	p.P54R	Missense_Mutation	2	197784861	G	C	24	50.00%	Deleterious		Passenger	passenger	.	.	1	0.88239	71%	Clonal	.	.	.	.	WES		
IDC50T	PHF21A	p.A442V	Missense_Mutation	11	45967515	G	A	27	29.63%	Deleterious	PASSENGER/OTHER	Passenger	passenger	.	.	1	0.79493	51%	Clonal	.	.	.	.	WES		
IDC50T	PIK3R6	p.D339Y	Missense_Mutation	17	8732182	C	A	91	62.64%	.	.	Passenger	passenger	.	loh	1	0.9757	92%	Clonal	.	.	.	.	WES		
IDC50T	PIP5K1A	p.G435S	Missense_Mutation	1	151212455	G	A	20	15.00%	Deleterious	PASSENGER/OTHER	Passenger	passenger	.	.	0.62	0.46811	20%	Subclonal	.	.	.	.	WES		
IDC50T	PKD1L1	p.D34N	Missense_Mutation	7	47983053	C	T	34	11.76%	Neutral	PASSENGER/OTHER	Passenger	passenger	.	.	0.72	0.54517	26%	Clonal	.	.	.	.	WES		
IDC50T	PKD2L2	p.S152N	Missense_Mutation	5	137230229	G	A	30	16.67%	Neutral	PASSENGER/OTHER	Passenger	passenger	.	.	0.85	0.63267	33%	Clonal	.	.	.	.	WES		
IDC50T	PPP2R2B	p.Y336S	Missense_Mutation	5	145972588	T	G	62	41.94%	Deleterious	PASSENGER/OTHER	Passenger	passenger	.	.	1	0.93665	81%	Clonal	.	.	.	.	WES		
IDC50T	PSMD14	p.D289H	Missense_Mutation	2	162267843	G	C	52	42.31%	Deleterious	.	Passenger	passenger	.	.	1	0.9436	80%	Clonal	.	.	.	.	WES		
IDC50T	PXDN	p.E965Q	Missense_Mutation	2	1652659	C	G	56	57.14%	Deleterious	PASSENGER/OTHER	Passenger	passenger	.	loh	1	0.94778	85%	Clonal	.	.	.	.	WES		
IDC50T	RAG2	p.I142V	Missense_Mutation	11	36615295	T	C	75	22.67%	Deleterious	PASSENGER/OTHER	Passenger	passenger	.	.	0.93	0.75013	57%	Clonal	.	.	.	.	WES		
IDC50T	RASGRP3	p.E104D	Missense_Mutation	2	33745695	A	C	26	19.23%	Deleterious	PASSENGER/OTHER	Passenger	passenger	.	.	0.79	0.5963	32%	Clonal	.	.	.	.	WES		
IDC50T	RBM20	p.S988C	Missense_Mutation	10	112581340	C	G	90	4.44%	Neutral	PASSENGER/OTHER	Passenger	passenger	.	loh	0.14	0	5%	Subclonal	.	.	.	.	WES		
IDC50T	RPL18	p.R146Q	Missense_Mutation	19	49119188	C	T	58	32.76%	Deleterious	.	Passenger	passenger	.	loh	1	0.92462	75%	Clonal	.	.	.	.	WES		
IDC50T	SCN3A	p.D1725H	Missense_Mutation	2	165947490	C	G	64	21.88%	Deleterious	PASSENGER/OTHER	Passenger	passenger	.	.	1	0.81331	59%	Clonal	.	.	.	.	WES		
IDC50T	SEC24B	p.L1202R	Missense_Mutation	4	110459665	T	G	32	34.38%	Deleterious	PASSENGER/OTHER	Passenger	passenger	.	.	1	0.91435	65%	Clonal	.	.	.	.	WES		
IDC50T	SEMA3G	p.G567R	Missense_Mutation	3	52472026	C	G	55	34.55%	Deleterious	PASSENGER/OTHER	Passenger	passenger	.	.	1	0.9382	75%	Clonal	.	.	.	.	WES		
IDC50T	SFT2D3	p.A23V	Missense_Mutation	2	128459170	C	T	9	33.33%	Neutral	PASSENGER/OTHER	Passenger	passenger	.	.	1	0.75169	32%	Clonal	.	.	.	.	WES		
IDC50T	SH3D19	p.R687W	Missense_Mutation	4	152049421	G	A	24	12.50%	Deleterious	PASSENGER/OTHER	Passenger	passenger	.	loh	0.39	0.13454	14%	Subclonal	.	.	.	.	WES		
IDC50T	SLC10A4	p.D403N	Missense_Mutation	4	48490849	G	A	71	8.45%	Deleterious	PASSENGER/OTHER	Passenger	passenger	.	.	0.43	0.07197	20%	Subclonal	.	.	.	.	WES		
IDC50T	SLC27A2	p.G423V	Missense_Mutation	15	50519186	G	T	16	25.00%	Deleterious	PASSENGER/OTHER	Passenger	passenger	.	.	0.78	0.58556	29%	Clonal	.	.	.	.	WES		
IDC50T	SLC2A1	p.A392S	Missense_Mutation	1	43393380	C	A	74	22.97%	Deleterious	PASSENGER/OTHER	Passenger	passenger	.	.	0.95	0.7593	57%	Clonal	.	.	.	.	WES		
IDC50T	SMEK1	p.S730N	Missense_Mutation	14	91927888	C	T	28	53.57%	Deleterious	PASSENGER/OTHER	Driver	likely_pathogenic	.	loh	1	0.92016	72%	Clonal	.	.	.	.	WES		
IDC50T	SOGA2	p.E1510Q	Missense_Mutation	18	8826036	G	C	53	49.06%	Deleterious	PASSENGER/OTHER	Passenger	passenger	.	loh	1	0.82007	80%	Clonal	.	.	.	.	WES		
IDC50T	SPEG	p.A2112S	Missense_Mutation	2	220348519	G	T	40	17.50%	Deleterious	PASSENGER/OTHER	Passenger	passenger	.	.	0.9	0.67161	40%	Clonal	.	.	.	.	WES		
IDC50T	SPIN3	p.R46W	Missense_Mutation	X	57021245	G	A	152	14.47%	Neutral	PASSENGER/OTHER	Passenger	passenger	.	.	0.74	0.41929	49%	Subclonal	.	.	.	.	WES		
IDC50T	SRCAP	p.G2940R	Missense_Mutation	16	30750179	G	A	221	10.41%	Deleterious	CANCER	Passenger	likely_pathogenic	.	.	0.53	0.00399	36%	Subclonal	.	.	.	.	WES		
IDC50T	ST14	p.Q528K	Missense_Mutation	11	130068414	C	A	101	32.67%	Neutral	PASSENGER/OTHER	Passenger	passenger	.	.	1	0.85553	71%	Clonal	.	.	.	.	WES		
IDC50T	SUPT6H	p.R409H	Missense_Mutation	17	27005858	G	A	71	29.58%	Deleterious	PASSENGER/OTHER	Passenger	passenger	.	.	1	0.94259	77%	Clonal	.	.	.	.	WES		
IDC50T	SYCP2L	p.D132H	Missense_Mutation	6	10898301	G	C	63	9.52%	Neutral	PASSENGER/OTHER	Passenger	passenger	.	.	0.68	0.48943	30%	Subclonal	.	.	.	.	WES		
IDC50T	SYNE1	p.L2203Q	Missense_Mutation	6	152728264	A	T	11	81.82%	Deleterious	PASSENGER/OTHER	Passenger	passenger	.	loh	1	0.60419	68%	Clonal	.	.	.	.	WES		
IDC50T	TAS1R2	p.G224A	Missense_Mutation	1	19181293	C	G	47	38.30%	Neutral	PASSENGER/OTHER	Passenger	passenger	.	.	1	0.9002	73%	Clonal	.	.	.	.	WES		
IDC50T	TMEM2	p.P1212T	Missense_Mutation	9	74309487	G	T	44	34.09%	Deleterious	PASSENGER/OTHER	Passenger	passenger	.	.	1	0.81646	61%	Clonal	.	.	.	.	WES		
IDC50T	TMEM253	p.G30A	Missense_Mutation	14	21569591	G	C	49	24.49%	Neutral	.	Passenger	passenger	.	.	1	0.82867	60%	Clonal	.	.	.	.	WES		
IDC50T	TONSL	p.T477A	Missense_Mutation	8	145665455	T	C	105	25.71%	Neutral	PASSENGER/OTHER	Passenger	passenger	.	.	1	0.94738	82%	Clonal	.	.	.	.	WES		
IDC50T	TRDV1	p.Q76H	Missense_Mutation	14	22564777	G	C	61	37.70%	.	PASSENGER/OTHER	Passenger	passenger	.	.	1	0.95245	80%	Clonal	.	.	.	.	WES		
IDC50T	TREML1	p.R76G	Missense_Mutation	6	41121646	G	C	149	14.09%	Neutral	PASSENGER/OTHER	Passenger	passenger	.	.	0.72	0.37196	48%	Subclonal	.	.	.	.	WES		
IDC50T	TRIP12	p.A439G	Missense_Mutation	2	230683219	G	C	42	45.24%	Deleterious	PASSENGER/OTHER	Passenger	passenger	.	.	1	0.94936	77%	Clonal	.	.	.	.	WES		
IDC50T	TRPV2	p.E351D	Missense_Mutation	17	16329541	G	T	64	26.56%	Deleterious	PASSENGER/OTHER	Passenger	passenger	.	loh	0.83	0.63386	52%	Clonal	.	.	.	.	WES		
IDC50T	VPS13C	p.E1106D	Missense_Mutation	15	62256047	T	A	17	23.53%	Deleterious	PASSENGER/OTHER	Passenger	passenger	.	.	0.73	0.55244	27%	Clonal	.	.	.	.	WES		
IDC50T	WDR13	p.W320C	Missense_Mutation	X	48460300	G	C	100	19.00%	Deleterious	PASSENGER/OTHER	Passenger	passenger	.	.	0.97	0.77994	60%	Clonal	.	.	.	.	WES		
IDC50T	WDR54	p.T240S	Missense_Mutation	2	74652285	C	G	130	25.38%	Neutral	PASSENGER/OTHER	Passenger	passenger	.	.	1	0.86034	71%	Clonal	.	.	.	.	WES		
IDC50T	WDR7	p.G934A	Missense_Mutation	18	54426137	G	C	34	14.71%	Neutral	PASSENGER/OTHER	Passenger	passenger	.	loh	0.46	0.12191	20%	Subclonal	.	.	.	.	WES		
IDC50T	ZBTB16	p.K95T	Missense_Mutation	11	113934306	A	C	74	27.03%	Deleterious	PASSENGER/OTHER	Passenger	passenger	.	.	0.84	0.65331	55%	Clonal	TRUE	.	.	.	WES		
IDC50T	ZFR2	p.S587F	Missense_Mutation	19	3819214	G	A	19	31.58%	Deleterious	PASSENGER/OTHER	Passenger	passenger	.	.	1	0.79154	46%	Clonal	.	.	.	.	WES		
IDC50T	ZNF454	p.Q169E	Missense_Mutation	5	178391910	C	G	19	42.11%	Neutral	PASSENGER/OTHER	Passenger	passenger	.	.	1	0.8186	54%	Clonal	.	.	.	.	WES		
IDC50T	ZNF81	p.R104I	Missense_Mutation	X	47774356	G	T	18	50.00%	Neutral	PASSENGER/OTHER	Passenger	passenger	.	.	1	0.88324	65%	Clonal	.	.	.	.	WES		
IDC50T	ZSCAN10	p.P3T	Missense_Mutation	16	3142767	G	T	165	21.21%	Deleterious	PASSENGER/OTHER	Passenger	passenger	.	.	1	0.87441	73%	Clonal	.	.	.	.	WES		
IDC50T	C10orf71	p.K1185*	Nonsense_Mutation	10	50534143	A	T	53	30.19%	Deleterious	.	.	passenger	.	.	0.94	0.75906	57%	Clonal	.	.	.	.	WES		
IDC50T	CEP112	p.Q670*	Nonsense_Mutation	17	63898425	G	A	91	17.58%	Deleterious	.	.	passenger	.	.	1	0.80886	60%	Clonal	.	.	.	.	WES		
IDC50T	CORO6	p.Q433*	Nonsense_Mutation	17	27942872	G	A	152	15.79%	Deleterious	.	.	passenger	.	.	0.97	0.78854	62%	Clonal	.	.	.	.	WES		
IDC50T	CSMD3	p.L1427*	Nonsense_Mutation	8	113564904	A	T	42	9.52%	Deleterious	.	.	passenger	.	.	0.87	0.62843	29%	Clonal	.	.	.	.	WES		
IDC50T	HTATSF1	p.Q246*	Nonsense_Mutation	X	135586524	C	T	44	22.73%	Deleterious	.	.	likely_pathogenic	.	.	0.94	0.71878	48%	Clonal	.	.	.	.	WES		
IDC50T	NFATC2	p.Y708*	Nonsense_Mutation	20	50049202	G	C	59	13.56%	Deleterious	.	.	likely_pathogenic	.	.	0.83	0.63257	40%	Clonal	TRUE	.	.	.	WES		
IDC50T	TTC39B	p.K427*	Nonsense_Mutation	9	15188085	T	A	37	21.62%	Deleterious	.	.	passenger	.	.	0.67	0.42872	35%	Subclonal	.	.	.	.	WES		
IDC50T	WWOX	p.R264*	Nonsense_Mutation	16	78458951	C	T	17	76.47%	Deleterious	.	.	likely_pathogenic	.	loh	1	0.62119	75%	Clonal	.	.	.	.	WES		
IDC50T	COL6A6	p.X1512_splice	Splice_Site	3	130317208	G	T	29	13.79%	Deleterious	.	.	passenger	.	.	0.57	0.37901	22%	Subclonal	.	.	.	.	WES		
IDC50T	FRRS1	p.X1_splice	Splice_Site	1	100214325	C	A	13	46.15%	.	.	.	passenger	.	.	1	0.82522	49%	Clonal	.	.	.	.	WES		
IDC50T	XPC	p.X100_splice	Splice_Site	3	14212052	T	A	42	28.57%	Deleterious	.	.	likely_pathogenic	.	.	1	0.8179	59%	Clonal	TRUE	.	.	.	WES		
IDC51T	BOC	p.E607Kfs*18	Frame_Shift_Del	3	112998099	CA	C	9	33.33%	.	.	.	passenger	.	.	1	0.8349	34%	Clonal	.	.	.	.	WES		
IDC51T	BRWD3	p.E747Nfs*9	Frame_Shift_Del	X	79971738	TCTTC	T	14	28.57%	.	.	.	passenger	.	.	1	0.76546	35%	Clonal	.	.	.	.	WES		
IDC51T	MAP1A	p.L2466*	Frame_Shift_Del	15	43821064	GACCTCTCA	G	141	7.09%	.	.	.	likely_pathogenic	.	loh	0.28	0	15%	Subclonal	.	.	.	.	WES		
IDC51T	CACNA1B	p.P34Sfs*87	Frame_Shift_Ins	9	140772471	C	CG	74	14.86%	.	.	.	passenger	.	.	0.58	0.20877	33%	Subclonal	.	.	.	.	WES		
IDC51T	CLASRP	p.K181_E183del	In_Frame_Del	19	45561078	GCAGAAAAGC	G	96	6.25%	.	.	.	passenger	.	loh	0.31	0.0021	14%	Subclonal	.	.	.	.	WES		
IDC51T	MAGED1	p.D563del	In_Frame_Del	X	51640673	CTGA	C	29	17.24%	.	.	.	passenger	.	.	0.85	0.68488	33%	Clonal	.	.	.	.	WES		
IDC51T	SFTPC	p.V44del	In_Frame_Del	8	22020158	CGTG	C	30	10.00%	.	.	.	passenger	.	loh	0.29	0.03585	10%	Subclonal	.	.	.	.	WES		
IDC51T	ACAD9	p.S171Y	Missense_Mutation	3	128615337	C	A	35	14.29%	Neutral	PASSENGER/OTHER	Passenger	passenger	.	.	0.7	0.58416	29%	Clonal	.	.	.	.	WES		
IDC51T	ADAMTSL4	p.P163T	Missense_Mutation	1	150525954	C	A	56	17.86%	Deleterious	CANCER	Passenger	likely_pathogenic	.	.	0.88	0.73009	46%	Clonal	.	.	.	.	WES		
IDC51T	AGAP2	p.G1114R	Missense_Mutation	12	58120574	C	G	66	15.15%	Deleterious	CANCER	Passenger	likely_pathogenic	.	loh	0.75	0.59916	40%	Clonal	.	.	.	.	WES		
IDC51T	ARHGEF18	p.E1018K	Missense_Mutation	19	7533846	G	A	159	27.04%	Deleterious	PASSENGER/OTHER	Passenger	passenger	.	loh	1	0.90747	76%	Clonal	.	.	.	.	WES		
IDC51T	ATXN7L3B	p.P73R	Missense_Mutation	12	74932110	C	G	45	13.33%	Deleterious	.	Passenger	passenger	.	.	0.92	0.72271	38%	Clonal	.	.	.	.	WES		
IDC51T	B3GALTL	p.E376V	Missense_Mutation	13	31891765	A	T	36	19.44%	Deleterious	PASSENGER/OTHER	Passenger	passenger	.	.	1	0.80245	46%	Clonal	.	.	.	.	WES		
IDC51T	BMP2	p.V303M	Missense_Mutation	20	6759452	G	A	52	7.69%	Deleterious	PASSENGER/OTHER	Passenger	passenger	.	loh	0.3	0.01388	12%	Subclonal	.	.	.	.	WES		
IDC51T	BOD1L1	p.T2297N	Missense_Mutation	4	13601634	G	T	53	13.21%	Neutral	PASSENGER/OTHER	Passenger	passenger	.	.	0.78	0.64755	36%	Clonal	.	.	.	.	WES		
IDC51T	BTAF1	p.L1823V	Missense_Mutation	10	93788607	C	G	25	32.00%	Deleterious	PASSENGER/OTHER	Passenger	passenger	.	.	1	0.88785	56%	Clonal	.	.	.	.	WES		
IDC51T	C10orf91	p.D113H	Missense_Mutation	10	134261464	G	C	66	12.12%	Neutral	PASSENGER/OTHER	Passenger	passenger	.	.	0.6	0.3554	30%	Subclonal	.	.	.	.	WES		
IDC51T	C20orf144	p.L51V	Missense_Mutation	20	32251362	C	G	214	12.62%	Neutral	PASSENGER/OTHER	Passenger	passenger	.	.	0.75	0.45719	52%	Subclonal	.	.	.	.	WES		
IDC51T	CCNE2	p.V262G	Missense_Mutation	8	95897341	A	C	89	8.99%	Deleterious	PASSENGER/OTHER	Passenger	passenger	.	.	0.89	0.71939	42%	Clonal	.	.	.	.	WES		
IDC51T	CEACAM4	p.N144K	Missense_Mutation	19	42128134	G	T	65	24.62%	Neutral	PASSENGER/OTHER	Passenger	passenger	.	loh	1	0.87109	64%	Clonal	.	.	.	.	WES		
IDC51T	CHD8	p.R574T	Missense_Mutation	14	21884062	C	G	15	40.00%	Deleterious	PASSENGER/OTHER	Driver	likely_pathogenic	.	.	1	0.89321	53%	Clonal	.	.	TRUE	.	WES		
IDC51T	CLVS1	p.W283S	Missense_Mutation	8	62370972	G	C	34	8.82%	Deleterious	PASSENGER/OTHER	Passenger	passenger	.	.	0.44	0.28481	15%	Subclonal	.	.	.	.	WES		
IDC51T	CNNM1	p.G810E	Missense_Mutation	10	101147665	G	A	88	25.00%	Deleterious	PASSENGER/OTHER	Passenger	passenger	.	.	1	0.89142	70%	Clonal	.	.	.	.	WES		
IDC51T	CNST	p.D301N	Missense_Mutation	1	246805303	G	A	15	20.00%	Deleterious	PASSENGER/OTHER	Passenger	passenger	.	.	0.99	0.71247	27%	Clonal	.	.	.	.	WES		
IDC51T	COL18A1	p.G374D	Missense_Mutation	21	46888630	G	A	90	16.67%	Neutral	PASSENGER/OTHER	Passenger	passenger	.	.	0.82	0.68376	49%	Clonal	.	.	.	.	WES		
IDC51T	COL22A1	p.G1288V	Missense_Mutation	8	139629164	C	A	48	8.33%	Deleterious	PASSENGER/OTHER	Passenger	passenger	.	.	0.83	0.66237	29%	Clonal	.	.	.	.	WES		
IDC51T	COPS7B	p.V42G	Missense_Mutation	2	232653405	T	G	22	27.27%	Deleterious	PASSENGER/OTHER	Passenger	passenger	.	loh	1	0.77926	42%	Clonal	.	.	.	.	WES		
IDC51T	CRLF1	p.I43T	Missense_Mutation	19	18710644	A	G	25	24.00%	Deleterious	PASSENGER/OTHER	Passenger	passenger	.	loh	0.94	0.73862	39%	Clonal	.	.	.	.	WES		
IDC51T	CSMD2	p.N1121H	Missense_Mutation	1	34174784	T	G	26	26.92%	Deleterious	PASSENGER/OTHER	Passenger	passenger	.	.	1	0.83942	49%	Clonal	.	.	.	.	WES		
IDC51T	CSNK1G2	p.V166L	Missense_Mutation	19	1978906	G	T	203	48.28%	Deleterious	PASSENGER/OTHER	Passenger	passenger	.	loh	1	0.77833	95%	Clonal	.	.	.	.	WES		
IDC51T	CYP27C1	p.E67Q	Missense_Mutation	2	127958887	C	G	25	36.00%	Deleterious	PASSENGER/OTHER	Passenger	passenger	.	.	1	0.92201	62%	Clonal	.	.	.	.	WES		
IDC51T	DBH	p.D526Y	Missense_Mutation	9	136522205	G	T	66	16.67%	Deleterious	PASSENGER/OTHER	Passenger	passenger	.	.	0.66	0.3969	37%	Subclonal	.	.	.	.	WES		
IDC51T	DBH	p.D526V	Missense_Mutation	9	136522206	A	T	66	16.67%	Deleterious	PASSENGER/OTHER	Passenger	passenger	.	.	0.66	0.3969	37%	Subclonal	.	.	.	.	WES		
IDC51T	DDX17	p.N579K	Missense_Mutation	22	38882399	A	T	39	35.90%	Deleterious	PASSENGER/OTHER	Passenger	passenger	.	.	1	0.93737	70%	Clonal	.	.	.	.	WES		
IDC51T	DDX54	p.Q606E	Missense_Mutation	12	113601994	G	C	118	16.10%	Neutral	PASSENGER/OTHER	Passenger	passenger	.	loh	0.96	0.80561	59%	Clonal	.	.	.	.	WES		
IDC51T	DENND5A	p.H118Q	Missense_Mutation	11	9225802	A	C	45	17.78%	Deleterious	PASSENGER/OTHER	Passenger	passenger	.	.	0.88	0.71816	42%	Clonal	.	.	.	.	WES		
IDC51T	DGKH	p.L464F	Missense_Mutation	13	42748218	C	T	78	10.26%	Deleterious	CANCER	Passenger	likely_pathogenic	.	.	0.61	0.38639	31%	Subclonal	.	.	.	.	WES		
IDC51T	DGKQ	p.S166C	Missense_Mutation	4	962308	T	A	132	20.45%	Deleterious	PASSENGER/OTHER	Passenger	passenger	.	.	1	0.89831	73%	Clonal	.	.	.	.	WES		
IDC51T	DND1	p.V142F	Missense_Mutation	5	140052210	C	A	52	28.85%	Deleterious	PASSENGER/OTHER	Passenger	passenger	.	loh	1	0.85967	62%	Clonal	.	.	.	.	WES		
IDC51T	DOCK3	p.D1988N	Missense_Mutation	3	51418859	G	A	56	35.71%	Deleterious	PASSENGER/OTHER	Passenger	passenger	.	loh	1	0.89953	65%	Clonal	.	.	.	.	WES		
IDC51T	DSCAM	p.D1661E	Missense_Mutation	21	41427704	G	T	64	23.44%	Neutral	PASSENGER/OTHER	Passenger	passenger	.	.	1	0.85839	62%	Clonal	.	.	.	.	WES		
IDC51T	DYNLT3	p.W86G	Missense_Mutation	X	37700299	A	C	21	23.81%	Deleterious	PASSENGER/OTHER	Passenger	passenger	.	.	0.94	0.72516	36%	Clonal	.	.	.	.	WES		
IDC51T	EPB41L4A	p.P563S	Missense_Mutation	5	111506050	G	A	17	64.71%	Deleterious	PASSENGER/OTHER	Driver	likely_pathogenic	.	loh	1	0.89553	70%	Clonal	.	.	.	.	WES		
IDC51T	EPHA3	p.F533L	Missense_Mutation	3	89456423	C	G	19	21.05%	Deleterious	PASSENGER/OTHER	Passenger	passenger	.	loh	0.83	0.66859	29%	Clonal	.	TRUE	.	YES	WES		
IDC51T	ETNK1	p.D112Y	Missense_Mutation	12	22778431	G	T	26	19.23%	Deleterious	PASSENGER/OTHER	Passenger	passenger	.	.	1	0.81455	41%	Clonal	.	.	.	.	WES		
IDC51T	ETV3	p.A486G	Missense_Mutation	1	157094715	G	C	75	17.33%	Deleterious	PASSENGER/OTHER	Passenger	passenger	.	.	0.85	0.71847	49%	Clonal	.	.	.	.	WES		
IDC51T	FAM214A	p.K144N	Missense_Mutation	15	52903425	C	G	35	62.86%	Deleterious	PASSENGER/OTHER	Driver	likely_pathogenic	.	loh	1	0.8925	82%	Clonal	.	.	.	.	WES		
IDC51T	FBF1	p.E722K	Missense_Mutation	17	73914280	C	T	326	25.46%	Deleterious	PASSENGER/OTHER	Passenger	passenger	.	.	1	0.9734	93%	Clonal	.	.	.	.	WES		
IDC51T	FKRP	p.D109N	Missense_Mutation	19	47259032	G	A	119	15.13%	Deleterious	.	Passenger	passenger	.	loh	0.75	0.53144	47%	Clonal	.	.	.	.	WES		
IDC51T	FTO	p.Q66E	Missense_Mutation	16	53859848	C	G	34	17.65%	Deleterious	PASSENGER/OTHER	Passenger	passenger	.	.	0.87	0.70219	37%	Clonal	.	.	.	.	WES		
IDC51T	GDPD4	p.W27S	Missense_Mutation	11	76990418	C	G	16	37.50%	Deleterious	PASSENGER/OTHER	Passenger	passenger	.	.	1	0.89765	53%	Clonal	.	.	.	.	WES		
IDC51T	GPBP1	p.P229R	Missense_Mutation	5	56542903	C	G	18	33.33%	Deleterious	PASSENGER/OTHER	Passenger	passenger	.	.	1	0.89598	51%	Clonal	.	.	.	.	WES		
IDC51T	GPR112	p.L374R	Missense_Mutation	X	135426986	T	G	16	18.75%	Neutral	PASSENGER/OTHER	Passenger	passenger	.	.	0.74	0.61592	23%	Clonal	.	.	.	.	WES		
IDC51T	GRM7	p.I45M	Missense_Mutation	3	6903210	C	G	255	11.76%	Deleterious	PASSENGER/OTHER	Passenger	passenger	.	loh	0.82	0.63865	57%	Clonal	.	.	.	.	WES		
IDC51T	GUCA1B	p.M177I	Missense_Mutation	6	42152625	C	G	68	17.65%	Deleterious	PASSENGER/OTHER	Passenger	passenger	.	.	0.87	0.72942	48%	Clonal	.	.	.	.	WES		
IDC51T	HECW2	p.F472L	Missense_Mutation	2	197184198	G	C	65	20.00%	Neutral	PASSENGER/OTHER	Passenger	passenger	.	.	1	0.85046	59%	Clonal	.	.	.	.	WES		
IDC51T	HTR2A	p.D23Y	Missense_Mutation	13	47469975	C	A	12	25.00%	Neutral	PASSENGER/OTHER	Passenger	passenger	.	.	0.98	0.71343	27%	Clonal	.	.	.	.	WES		
IDC51T	IFNGR2	p.V253M	Missense_Mutation	21	34805056	G	A	46	8.70%	Neutral	PASSENGER/OTHER	Passenger	passenger	.	.	0.43	0.19164	17%	Subclonal	.	.	.	.	WES		
IDC51T	ITIH1	p.P92R	Missense_Mutation	3	52812492	C	G	33	36.36%	Deleterious	PASSENGER/OTHER	Driver	likely_pathogenic	.	loh	1	0.87653	57%	Clonal	.	.	.	.	WES		
IDC51T	JARID2	p.G889R	Missense_Mutation	6	15507581	G	C	141	9.93%	Deleterious	PASSENGER/OTHER	Driver	likely_pathogenic	.	.	0.59	0.1693	35%	Subclonal	.	.	.	.	WES		
IDC51T	KRTAP4-11	p.R51K	Missense_Mutation	17	39274416	C	T	46	13.04%	Neutral	PASSENGER/OTHER	Passenger	passenger	.	.	0.64	0.49656	29%	Subclonal	.	.	.	.	WES		
IDC51T	LAT	p.E255D	Missense_Mutation	16	29001288	G	C	35	37.14%	Neutral	.	Passenger	passenger	.	loh	1	0.93209	71%	Clonal	.	.	.	.	WES		
IDC51T	LGALS4	p.S229F	Missense_Mutation	19	39292771	G	A	27	37.04%	Neutral	PASSENGER/OTHER	Passenger	passenger	.	loh	1	0.92712	63%	Clonal	.	.	.	.	WES		
IDC51T	LGR6	p.L121M	Missense_Mutation	1	202205054	C	A	56	19.64%	Neutral	PASSENGER/OTHER	Passenger	passenger	.	.	0.97	0.78261	50%	Clonal	.	.	.	.	WES		
IDC51T	LRRC36	p.D315N	Missense_Mutation	16	67401108	G	A	32	28.13%	Deleterious	PASSENGER/OTHER	Passenger	passenger	.	.	1	0.86399	56%	Clonal	.	.	.	.	WES		
IDC51T	LYST	p.E2176K	Missense_Mutation	1	235922627	C	T	54	14.81%	Deleterious	PASSENGER/OTHER	Passenger	passenger	.	.	0.73	0.59056	36%	Clonal	.	.	.	.	WES		
IDC51T	MACF1	p.L1689R	Missense_Mutation	1	39818731	T	G	48	20.83%	Deleterious	PASSENGER/OTHER	Passenger	passenger	.	.	1	0.84207	55%	Clonal	.	.	.	.	WES		
IDC51T	MAP4	p.E608Q	Missense_Mutation	3	47957495	C	G	17	23.53%	Neutral	PASSENGER/OTHER	Passenger	passenger	.	loh	1	0.77095	35%	Clonal	.	.	.	.	WES		
IDC51T	METTL2B	p.C124R	Missense_Mutation	7	128119379	T	C	9	33.33%	Neutral	PASSENGER/OTHER	Passenger	passenger	.	loh	1	0.78375	31%	Clonal	.	.	.	.	WES		
IDC51T	METTL2B	p.C124S	Missense_Mutation	7	128119380	G	C	9	33.33%	Neutral	PASSENGER/OTHER	Passenger	passenger	.	loh	1	0.78375	31%	Clonal	.	.	.	.	WES		
IDC51T	MLXIP	p.R679Q	Missense_Mutation	12	122622019	G	A	16	25.00%	Deleterious	PASSENGER/OTHER	Passenger	passenger	.	loh	1	0.82249	38%	Clonal	.	.	.	.	WES		
IDC51T	MMRN1	p.E475D	Missense_Mutation	4	90856256	G	C	30	10.00%	Deleterious	PASSENGER/OTHER	Passenger	passenger	.	.	0.49	0.3832	17%	Subclonal	.	.	.	.	WES		
IDC51T	MSANTD3-TMEFF1	p.A26D	Missense_Mutation	9	103204627	C	A	38	42.11%	Neutral	PASSENGER/OTHER	Passenger	passenger	.	loh	1	0.92844	72%	Clonal	.	.	.	.	WES		
IDC51T	MURC	p.A283V	Missense_Mutation	9	103348486	C	T	46	19.57%	Deleterious	PASSENGER/OTHER	Passenger	passenger	.	loh	0.77	0.63107	40%	Clonal	.	.	.	.	WES		
IDC51T	NDUFAB1	p.P14S	Missense_Mutation	16	23607572	G	A	88	5.68%	Neutral	.	Passenger	passenger	.	loh	0.34	0.02056	14%	Subclonal	.	.	.	.	WES		
IDC51T	NEDD4L	p.R467H	Missense_Mutation	18	56010160	G	A	10	40.00%	Deleterious	PASSENGER/OTHER	Passenger	passenger	.	.	1	0.84318	40%	Clonal	.	.	.	.	WES		
IDC51T	NELFCD	p.E391D	Missense_Mutation	20	57566965	G	C	44	15.91%	Deleterious	.	Passenger	passenger	.	.	1	0.82223	47%	Clonal	.	.	.	.	WES		
IDC51T	NFKBIA	p.E43K	Missense_Mutation	14	35873724	C	T	136	30.88%	Deleterious	PASSENGER/OTHER	Passenger	passenger	.	.	1	0.95943	87%	Clonal	.	.	.	YES	WES		
IDC51T	NOL9	p.Q160E	Missense_Mutation	1	6610594	G	C	42	23.81%	Deleterious	PASSENGER/OTHER	Passenger	passenger	.	.	1	0.83368	54%	Clonal	.	.	.	.	WES		
IDC51T	NRAP	p.L833V	Missense_Mutation	10	115381900	G	C	56	21.43%	Neutral	PASSENGER/OTHER	Passenger	passenger	.	.	1	0.8199	55%	Clonal	.	.	.	.	WES		
IDC51T	NUMBL	p.P561A	Missense_Mutation	19	41173522	G	C	67	23.88%	Neutral	PASSENGER/OTHER	Passenger	passenger	.	loh	1	0.86625	63%	Clonal	.	.	.	.	WES		
IDC51T	OR11L1	p.E269K	Missense_Mutation	1	248004394	C	T	40	12.50%	Neutral	PASSENGER/OTHER	Passenger	passenger	.	.	0.62	0.48111	26%	Subclonal	.	.	.	.	WES		
IDC51T	OR9Q2	p.Q94E	Missense_Mutation	11	57958242	C	G	102	25.49%	Neutral	PASSENGER/OTHER	Passenger	passenger	.	.	1	0.89313	77%	Clonal	.	.	.	.	WES		
IDC51T	PABPC1	p.R370H	Missense_Mutation	8	101721823	C	T	30	10.00%	Deleterious	PASSENGER/OTHER	Passenger	passenger	.	.	0.99	0.70944	27%	Clonal	.	.	.	.	WES		
IDC51T	PCMTD1	p.G88E	Missense_Mutation	8	52773449	C	T	22	40.91%	Deleterious	CANCER	Passenger	likely_pathogenic	.	.	1	0.92814	62%	Clonal	.	.	.	.	WES		
IDC51T	PDS5B	p.H772N	Missense_Mutation	13	33309375	C	A	49	18.37%	Deleterious	PASSENGER/OTHER	Passenger	passenger	.	.	1	0.8044	50%	Clonal	.	.	.	.	WES		
IDC51T	PFKM	p.S145T	Missense_Mutation	12	48525159	T	A	95	6.32%	Deleterious	PASSENGER/OTHER	Passenger	passenger	.	.	0.44	0.10942	20%	Subclonal	.	.	.	.	WES		
IDC51T	PKD1L1	p.S2149I	Missense_Mutation	7	47870842	C	A	67	8.96%	Neutral	PASSENGER/OTHER	Passenger	passenger	.	.	0.71	0.58363	31%	Clonal	.	.	.	.	WES		
IDC51T	PLCXD3	p.L116F	Missense_Mutation	5	41382392	C	A	30	36.67%	Deleterious	PASSENGER/OTHER	Passenger	passenger	.	.	1	0.92863	67%	Clonal	.	.	.	.	WES		
IDC51T	PLXNB1	p.H1205N	Missense_Mutation	3	48457444	G	T	63	14.29%	Deleterious	PASSENGER/OTHER	Passenger	passenger	.	loh	0.7	0.54367	37%	Clonal	.	.	.	.	WES		
IDC51T	POU6F2	p.P394H	Missense_Mutation	7	39472830	C	A	49	14.29%	Deleterious	PASSENGER/OTHER	Passenger	passenger	.	.	1	0.79494	45%	Clonal	.	.	.	.	WES		
IDC51T	RBM27	p.P679L	Missense_Mutation	5	145641215	C	T	28	32.14%	Deleterious	CANCER	Passenger	likely_pathogenic	.	loh	1	0.84634	55%	Clonal	.	.	.	.	WES		
IDC51T	RGSL1	p.R623C	Missense_Mutation	1	182491180	C	T	29	20.69%		PASSENGER/OTHER	Passenger	passenger	.	.	1	0.76076	41%	Clonal	.	.	.	.	WES		
IDC51T	RP11-766F14.2	p.L296V	Missense_Mutation	4	100574920	G	C	78	19.23%	.	PASSENGER/OTHER	Passenger	passenger	.	.	0.95	0.79168	55%	Clonal	.	.	.	.	WES		
IDC51T	RPAP3	p.S428L	Missense_Mutation	12	48073280	G	A	41	9.76%	Deleterious	PASSENGER/OTHER	Passenger	passenger	.	.	0.68	0.56861	25%	Clonal	.	.	.	.	WES		
IDC51T	RPS6KC1	p.L251V	Missense_Mutation	1	213303148	T	G	41	58.54%	Deleterious	PASSENGER/OTHER	Passenger	passenger	.	.	1	0.67264	84%	Clonal	.	.	.	.	WES		
IDC51T	SAMD4A	p.E61Q	Missense_Mutation	14	55034815	G	C	19	31.58%	Deleterious	CANCER	Passenger	likely_pathogenic	.	.	1	0.89152	51%	Clonal	.	.	.	.	WES		
IDC51T	SH3RF2	p.V436I	Missense_Mutation	5	145428792	G	A	58	8.62%	Deleterious	PASSENGER/OTHER	Passenger	passenger	.	loh	0.34	0.01531	15%	Subclonal	.	.	.	.	WES		
IDC51T	SIAH1	p.Q248E	Missense_Mutation	16	48395691	G	C	30	36.67%	Deleterious	PASSENGER/OTHER	Passenger	passenger	.	.	1	0.93059	65%	Clonal	.	.	.	.	WES		
IDC51T	SLK	p.D680N	Missense_Mutation	10	105762974	G	A	29	55.17%	Deleterious	PASSENGER/OTHER	Passenger	passenger	.	.	1	0.8369	77%	Clonal	.	.	.	.	WES		
IDC51T	SNAP91	p.L567S	Missense_Mutation	6	84302975	A	G	41	7.32%	Deleterious	PASSENGER/OTHER	Passenger	passenger	.	loh	0.29	0.03153	10%	Subclonal	.	.	.	.	WES		
IDC51T	SOGA3	p.K111R	Missense_Mutation	6	127837428	T	C	32	31.25%	Neutral	PASSENGER/OTHER	Passenger	passenger	.	loh	1	0.92449	63%	Clonal	.	.	.	.	WES		
IDC51T	SUCNR1	p.A257D	Missense_Mutation	3	151599101	C	A	67	20.90%	Deleterious	PASSENGER/OTHER	Passenger	passenger	.	.	1	0.82277	57%	Clonal	.	.	.	.	WES		
IDC51T	SUGP2	p.Y450H	Missense_Mutation	19	19135809	A	G	54	27.78%	Deleterious	PASSENGER/OTHER	Passenger	passenger	.	loh	1	0.85071	61%	Clonal	.	.	.	.	WES		
IDC51T	TAS2R10	p.W201C	Missense_Mutation	12	10978266	C	A	34	17.65%	Deleterious	PASSENGER/OTHER	Passenger	passenger	.	.	1	0.80597	44%	Clonal	.	.	.	.	WES		
IDC51T	TAS2R10	p.W201L	Missense_Mutation	12	10978267	C	A	35	14.29%	Deleterious	PASSENGER/OTHER	Passenger	passenger	.	.	0.99	0.73642	36%	Clonal	.	.	.	.	WES		
IDC51T	TCHH	p.Q618H	Missense_Mutation	1	152083839	C	G	227	33.04%	Neutral	PASSENGER/OTHER	Passenger	passenger	.	.	1	0.75381	91%	Clonal	.	.	.	.	WES		
IDC51T	TGFB2	p.S170Y	Missense_Mutation	1	218578589	C	A	63	14.29%	Deleterious	PASSENGER/OTHER	Passenger	passenger	.	.	0.7	0.54367	37%	Clonal	.	.	.	.	WES		
IDC51T	TMEM214	p.E65Q	Missense_Mutation	2	27256976	G	C	55	18.18%	Deleterious	PASSENGER/OTHER	Passenger	passenger	.	.	0.9	0.74017	46%	Clonal	.	.	.	.	WES		
IDC51T	TMPRSS15	p.N273S	Missense_Mutation	21	19732136	T	C	24	29.17%	Neutral	PASSENGER/OTHER	Passenger	passenger	.	.	1	0.85842	51%	Clonal	.	.	.	.	WES		
IDC51T	TRPM5	p.D177H	Missense_Mutation	11	2441572	C	G	70	42.86%	Deleterious	PASSENGER/OTHER	Passenger	passenger	.	.	1	0.95827	85%	Clonal	.	.	.	.	WES		
IDC51T	TRPT1	p.G92W	Missense_Mutation	11	63992326	C	A	121	12.40%	Neutral	PASSENGER/OTHER	Passenger	passenger	.	.	0.74	0.54	44%	Clonal	.	.	.	.	WES		
IDC51T	TTN	p.H3112Y	Missense_Mutation	2	179632623	G	A	22	18.18%	Deleterious	PASSENGER/OTHER	Passenger	passenger	.	loh	0.71	0.59866	27%	Clonal	.	.	.	.	WES		
IDC51T	TXNDC15	p.P315L	Missense_Mutation	5	134235236	C	T	16	18.75%	Deleterious	PASSENGER/OTHER	Passenger	passenger	.	loh	0.74	0.61592	23%	Clonal	.	.	.	.	WES		
IDC51T	WDR19	p.D1287Y	Missense_Mutation	4	39279769	G	T	30	30.00%	Deleterious	PASSENGER/OTHER	Passenger	passenger	.	.	1	0.91371	60%	Clonal	.	.	.	.	WES		
IDC51T	WDR87	p.S2593A	Missense_Mutation	19	38376417	A	C	48	37.50%	Neutral	PASSENGER/OTHER	Passenger	passenger	.	loh	1	0.94964	75%	Clonal	.	.	.	.	WES		
IDC51T	ZNF251	p.R179T	Missense_Mutation	8	145948509	C	G	90	8.89%	Neutral	PASSENGER/OTHER	Passenger	passenger	.	.	0.62	0.40783	31%	Subclonal	.	.	.	.	WES		
IDC51T	ZNF280D	p.P96T	Missense_Mutation	15	56993226	G	T	71	14.08%	Deleterious	PASSENGER/OTHER	Passenger	passenger	.	.	0.84	0.69443	44%	Clonal	.	.	.	.	WES		
IDC51T	ZNF33B	p.H265Y	Missense_Mutation	10	43089605	G	A	26	15.38%	Neutral	PASSENGER/OTHER	Passenger	passenger	.	.	0.76	0.62887	28%	Clonal	.	.	.	.	WES		
IDC51T	ZNF517	p.R152W	Missense_Mutation	8	146032755	C	T	204	60.78%	Neutral	PASSENGER/OTHER	Passenger	passenger	.	.	1	0.40308	97%	Clonal	.	.	.	.	WES		
IDC51T	ZSCAN10	p.T723P	Missense_Mutation	16	3139103	T	G	77	42.86%	Deleterious	PASSENGER/OTHER	Passenger	passenger	.	loh	1	0.85048	87%	Clonal	.	.	.	.	WES		
IDC51T	BOC	p.E607*	Nonsense_Mutation	3	112998101	G	T	8	37.50%	Deleterious	.	.	passenger	.	.	1	0.84092	35%	Clonal	.	.	.	.	WES		
IDC51T	C19orf24	p.Q99*	Nonsense_Mutation	19	1277195	C	T	85	57.65%	.	.	.	likely_pathogenic	.	loh	1	0.52482	91%	Clonal	.	.	.	.	WES		
IDC51T	C1orf112	p.Y385*	Nonsense_Mutation	1	169798431	C	A	18	16.67%	Neutral	PASSENGER/OTHER	Passenger	passenger	.	.	0.82	0.65663	25%	Clonal	.	.	.	.	WES		
IDC51T	NRAP	p.Q840*	Nonsense_Mutation	10	115381879	G	A	52	21.15%	Deleterious	.	.	passenger	.	.	1	0.80962	53%	Clonal	.	.	.	.	WES		
IDC51T	OR5H15	p.Y313*	Nonsense_Mutation	3	97888482	C	A	14	57.14%	Neutral	.	.	passenger	.	loh	1	0.92596	62%	Clonal	.	.	.	.	WES		
IDC51T	RBBP8	p.G621*	Nonsense_Mutation	18	20573732	G	T	32	34.38%	Deleterious	.	.	passenger	.	.	1	0.93258	66%	Clonal	.	.	.	.	WES		
IDC51T	SLC19A1	p.W363*	Nonsense_Mutation	21	46950746	C	T	134	12.69%	Deleterious	.	.	passenger	.	.	0.63	0.19152	39%	Subclonal	.	.	.	.	WES		
IDC51T	SLC43A2	p.W229*	Nonsense_Mutation	17	1494901	C	T	42	45.24%	Deleterious	.	.	likely_pathogenic	.	loh	1	0.94865	77%	Clonal	.	.	.	.	WES		
IDC51T	ASAP2	p.X341_splice	Splice_Site	2	9484930	G	A	22	13.64%	Deleterious	.	.	passenger	.	.	0.67	0.57505	22%	Clonal	.	.	.	.	WES		
IDC51T	EMP3	p.X61_splice	Splice_Site	19	48830881	G	T	59	30.51%	Deleterious	.	.	likely_pathogenic	.	loh	1	0.90223	72%	Clonal	.	.	.	.	WES		
IDC52T	PALB2	p.Y79Lfs*2	Frame_Shift_Ins	16	23647631	T	TA	31	41.94%	.	.	.	likely_pathogenic	.	.	1	0.93962	70%	Clonal	TRUE	.	.	YES	MSK-IMPACT	GTAATCCTCCTGGGCCATCT	TTTTCCTCCTCAGAACCTAAAA
IDC52T	CDH1	p.S131F	Missense_Mutation	16	68842331	C	T	24	33.33%	Neutral	PASSENGER/OTHER	Passenger	passenger	.	.	1	0.84167	55%	Clonal	TRUE	TRUE	TRUE	YES	MSK-IMPACT		
IDC52T	ETV1	p.S196F	Missense_Mutation	7	13971342	G	A	18	27.78%	Deleterious	PASSENGER/OTHER	Passenger	passenger	.	.	1	0.83817	46%	Clonal	TRUE	.	.	YES	MSK-IMPACT		
IDC52T	IKZF1	p.E170Q	Missense_Mutation	7	50450324	G	C	30	16.67%	Deleterious	PASSENGER/OTHER	Passenger	passenger	.	.	0.75	0.52409	31%	Clonal	TRUE	.	.	YES	MSK-IMPACT		
IDC52T	TSC2	p.C811F	Missense_Mutation	16	2124277	G	T	140	14.29%	Deleterious	PASSENGER/OTHER	Passenger	passenger	.	.	0.79	0.51481	51%	Clonal	TRUE	.	.	YES	MSK-IMPACT		
IDC53T	ADAMTSL4	p.S674Cfs*21	Frame_Shift_Del	1	150529782	ACT	A	271	45.76%	.	.	.	passenger	.	.	1	0.95553	96%	Clonal	.	.	.	.	WES		
IDC53T	AMMECR1	p.G57Efs*102	Frame_Shift_Del	X	109561117	TCCGGTTAGACCTC	T	63	11.11%	.	.	.	passenger	.	.	0.53	0.20198	26%	Subclonal	.	.	.	.	WES		
IDC53T	AP2A1	p.D473Qfs*5	Frame_Shift_Del	19	50303364	ACCGTGATGACGTCCAGGGCTATG	A	66	10.61%	.	.	.	passenger	.	.	0.51	0.1542	25%	Subclonal	.	.	.	.	WES		
IDC53T	LRP4	p.D91Efs*306	Frame_Shift_Del	11	46921855	CG	C	24	16.67%	.	.	.	passenger	.	.	0.79	0.60557	28%	Clonal	.	.	.	.	WES		
IDC53T	LRRC8B	p.N619*	Frame_Shift_Del	1	90050062	ATAACC	A	56	5.36%	.	.	.	passenger	.	.	0.26	0.01136	9%	Subclonal	.	.	.	.	WES		
IDC53T	NEDD4	p.G480Dfs*7	Frame_Shift_Del	15	56207590	TC	T	61	19.67%	.	.	.	passenger	.	.	0.94	0.73878	51%	Clonal	.	.	.	.	WES		
IDC53T	NINL	p.E705Sfs*37	Frame_Shift_Del	20	25459647	CG	C	122	42.62%	.	.	.	passenger	.	.	1	0.96954	90%	Clonal	.	.	.	.	WES		
IDC53T	PCDH15	p.G1883Afs*9	Frame_Shift_Del	10	55581845	CT	C	74	10.81%	.	.	.	passenger	.	.	0.52	0.13702	26%	Subclonal	.	.	.	.	WES		
IDC53T	PIP5K1B	p.T139Qfs*19	Frame_Shift_Del	9	71503989	CA	C	32	21.88%	.	.	.	likely_pathogenic	.	loh	1	0.74425	44%	Clonal	.	.	.	.	WES		
IDC53T	RPAP2	p.V472Hfs*13	Frame_Shift_Del	1	92789888	TATGTTTTGGGTGAAGAAACCACCAA	T	42	7.14%	.	.	.	passenger	.	.	0.34	0.08942	12%	Subclonal	.	.	.	.	WES		
IDC53T	TDRD12	p.T824Wfs*17	Frame_Shift_Del	19	33293959	CGAGTTCACCGCAG	C	107	9.35%	.	.	.	passenger	.	.	0.45	0.01538	24%	Subclonal	.	.	.	.	WES		
IDC53T	TRPC7	p.V358Afs*7	Frame_Shift_Del	5	135610406	GCCTATGGAGA	G	52	5.77%	.	.	.	likely_pathogenic	.	loh	0.39	0.18288	14%	Subclonal	.	.	.	.	WES		
IDC53T	NEFM	p.S142Efs*52	Frame_Shift_Ins	8	24771715	C	CGGCAGAAGCA	127	15.75%	.	.	.	passenger	.	.	0.75	0.47525	49%	Subclonal	.	.	.	.	WES		
IDC53T	ARHGEF10	p.G1227_G1229del	In_Frame_Del	8	1905068	GTGGAGGAGC	G	51	7.84%	.	.	.	passenger	.	.	0.37	0.07592	15%	Subclonal	.	.	.	.	WES		
IDC53T	C2orf54	p.L329_L337del	In_Frame_Del	2	241827947	GGCAGCTTCCGCGTGGCCAGGCAACAGA	G	98	9.18%	.	.	.	passenger	.	.	0.44	0.01972	23%	Subclonal	.	.	.	.	WES		

IDC53T	*FANCF*	p.V207_P212del	In_Frame_Del	11	22646720	GCGGCTGCAACAGCGCCAC	G	155	5.16%	.	.	.	likely_pathogenic	.	.	0.25	0	12%	Subclonal	TRUE	.	.	.	WES		
IDC53T	*FBN2*	p.C184_G187del	In_Frame_Del	5	127855039	CTGACATCCATTT	C	41	17.07%	.	.	.	passenger	.	.	0.81	0.62977	38%	Clonal	.	.	.	.	WES		
IDC53T	*NPC1L1*	p.I361del	In_Frame_Del	7	44578912	GGAT	G	124	25.81%	.	.	.	passenger	.	.	1	0.89509	76%	Clonal	.	.	.	.	WES		
IDC53T	*TENM4*	p.L286_F287del	In_Frame_Del	11	78601052	TGAAGAG	T	23	21.74%	.	.	.	passenger	.	.	1	0.71843	38%	Clonal	.	.	.	.	WES		
IDC53T	*AMMECR1*	p.L70_P72dup	In_Frame_Ins	X	109561082	G	GGGGGAGAGA	47	6.38%	.	.	.	passenger	.	.	0.3	0.04521	11%	Subclonal	.	.	.	.	WES		
IDC53T	*SEPT3*	p.R301Q	Missense_Mutation	22	42390330	G	A	33	30.30%	Deleterious	PASSENGER/OTHER	Passenger	passenger	.	loh	1	0.80466	54%	Clonal	.	.	.	.	WES		
IDC53T	*AAK1*	p.A570S	Missense_Mutation	2	69741671	C	A	17	17.65%	Neutral	PASSENGER/OTHER	Passenger	passenger	.	.	0.84	0.62155	25%	Clonal	.	.	.	.	WES		
IDC53T	*ABCA6*	p.V566L	Missense_Mutation	17	67110989	C	G	50	20.00%	Deleterious	PASSENGER/OTHER	Passenger	passenger	.	.	1	0.83997	57%	Clonal	.	.	.	.	WES		
IDC53T	*ADAMTS12*	p.R1395G	Missense_Mutation	5	33549431	G	C	213	43.66%	Deleterious	PASSENGER/OTHER	Passenger	passenger	.	.	1	0.9739	94%	Clonal	.	.	.	.	WES		
IDC53T	*ADAMTS4*	p.A286S	Missense_Mutation	1	161166448	C	A	131	39.69%	Deleterious	PASSENGER/OTHER	Passenger	passenger	.	.	1	0.96698	90%	Clonal	.	.	.	.	WES		
IDC53T	*ANKRD31*	p.G727C	Missense_Mutation	5	74443057	C	A	65	12.31%	Neutral	CANCER	Passenger	passenger	.	.	0.59	0.28693	30%	Subclonal	.	.	.	.	WES		
IDC53T	*AOC1*	p.S560P	Missense_Mutation	7	150555958	T	C	96	50.00%	Neutral	PASSENGER/OTHER	Passenger	passenger	.	loh	1	0.96758	89%	Clonal	.	.	.	.	WES		
IDC53T	*AQP4*	p.I96T	Missense_Mutation	18	24442306	A	G	58	50.00%	Deleterious	PASSENGER/OTHER	Passenger	passenger	.	loh	1	0.96167	84%	Clonal	.	.	.	.	WES		
IDC53T	*ARID1A*	p.G794E	Missense_Mutation	1	27088772	G	A	12	41.67%	Deleterious	PASSENGER/OTHER	Passenger	passenger	.	loh	1	0.8084	41%	Clonal	TRUE	TRUE	TRUE	YES	WES		
IDC53T	*BCOR*	p.T581N	Missense_Mutation	X	39932857	G	T	104	21.15%	Neutral	PASSENGER/OTHER	Passenger	passenger	.	.	1	0.81874	63%	Clonal	TRUE	.	TRUE	YES	WES		
IDC53T	*BMPR1B*	p.P508A	Missense_Mutation	4	96075747	C	G	60	35.00%	Deleterious	PASSENGER/OTHER	Passenger	passenger	.	.	1	0.92739	76%	Clonal	.	.	.	.	WES		
IDC53T	*BRD2*	p.R519C	Missense_Mutation	6	32945759	C	T	85	34.12%	Deleterious	PASSENGER/OTHER	Driver	likely_pathogenic	.	loh	1	0.90808	81%	Clonal	.	.	.	.	WES		
IDC53T	*C12orf71*	p.E122V	Missense_Mutation	12	27235052	T	A	72	6.94%	Neutral	PASSENGER/OTHER	Passenger	passenger	.	.	0.33	0.01195	14%	Subclonal	.	.	.	.	WES		
IDC53T	*C2orf16*	p.E64V	Missense_Mutation	2	27799630	A	T	47	8.51%	Neutral	PASSENGER/OTHER	Passenger	passenger	.	.	0.41	0.12279	16%	Subclonal	.	.	.	.	WES		
IDC53T	*CACNA1A*	p.I1381V	Missense_Mutation	19	13372373	T	C	38	36.84%	Deleterious	PASSENGER/OTHER	Passenger	passenger	.	.	1	0.93132	70%	Clonal	.	.	.	.	WES		
IDC53T	*CDH2*	p.E393Q	Missense_Mutation	18	25572786	C	G	38	23.68%	Deleterious	PASSENGER/OTHER	Passenger	passenger	.	loh	0.89	0.69713	45%	Clonal	.	.	.	.	WES		
IDC53T	*CDH22*	p.I340L	Missense_Mutation	20	44841648	T	G	112	10.71%	Deleterious	PASSENGER/OTHER	Passenger	passenger	.	.	0.94	0.73157	50%	Clonal	.	.	.	.	WES		
IDC53T	*CDH4*	p.A630T	Missense_Mutation	20	60503364	G	A	514	6.81%	Neutral	PASSENGER/OTHER	Passenger	passenger	.	.	0.66	0.08219	48%	Subclonal	.	.	.	.	WES		
IDC53T	*CDK12*	p.P670S	Missense_Mutation	17	37646886	C	T	49	30.61%	Deleterious	CANCER	Passenger	likely_pathogenic	.	.	1	0.88002	68%	Clonal	TRUE	TRUE	TRUE	YES	WES		
IDC53T	*CDK20*	p.R158H	Missense_Mutation	9	90585718	C	T	73	27.40%	Deleterious	PASSENGER/OTHER	Passenger	passenger	.	loh	1	0.87277	70%	Clonal	.	.	.	.	WES		
IDC53T	*CDYL2*	p.S34R	Missense_Mutation	16	80718949	G	T	114	12.28%	Deleterious	PASSENGER/OTHER	Passenger	passenger	.	.	0.71	0.44293	42%	Subclonal	.	.	.	.	WES		
IDC53T	*CELF3*	p.A216T	Missense_Mutation	1	151680109	C	T	61	27.87%	Deleterious	PASSENGER/OTHER	Passenger	passenger	.	.	1	0.86678	68%	Clonal	.	.	.	.	WES		
IDC53T	*CEP72*	p.G5A	Missense_Mutation	5	612490	G	C	5	60.00%	Neutral	PASSENGER/OTHER	Passenger	passenger	.	.	1	0.82342	37%	Clonal	.	.	.	.	WES		
IDC53T	*CHAT*	p.E576K	Missense_Mutation	10	50863232	G	A	93	15.05%	Deleterious	PASSENGER/OTHER	Passenger	passenger	.	.	0.72	0.45791	43%	Subclonal	.	.	.	.	WES		
IDC53T	*CHRNA4*	p.A563D	Missense_Mutation	20	61981075	G	T	313	26.52%	Deleterious	PASSENGER/OTHER	Passenger	passenger	.	.	1	0.53741	94%	Clonal	.	.	.	.	WES		
IDC53T	*CHST7*	p.P280L	Missense_Mutation	X	46434205	C	T	179	15.64%	Deleterious	PASSENGER/OTHER	Passenger	passenger	.	.	0.75	0.37897	52%	Subclonal	.	.	.	.	WES		
IDC53T	*COIL*	p.F51L	Missense_Mutation	17	55038228	G	C	58	46.55%	Deleterious	PASSENGER/OTHER	Passenger	passenger	.	.	1	0.94534	84%	Clonal	.	.	.	.	WES		
IDC53T	*COPG1*	p.Q78E	Missense_Mutation	3	128971526	C	G	44	45.45%	Deleterious	PASSENGER/OTHER	Passenger	passenger	.	.	1	0.94567	79%	Clonal	.	.	.	.	WES		
IDC53T	*CRNKL1*	p.R127K	Missense_Mutation	20	20033090	C	T	74	13.51%	Neutral	PASSENGER/OTHER	Passenger	passenger	.	.	0.64	0.35926	35%	Subclonal	.	.	.	.	WES		
IDC53T	*CSMD2*	p.L167I	Missense_Mutation	1	34401454	G	T	109	15.60%	Deleterious	PASSENGER/OTHER	Passenger	passenger	.	.	0.74	0.48555	47%	Subclonal	.	.	.	.	WES		
IDC53T	*CXorf36*	p.R187H	Missense_Mutation	X	45017072	C	T	43	11.63%	Deleterious	PASSENGER/OTHER	Passenger	passenger	.	.	0.55	0.33408	24%	Subclonal	.	.	.	.	WES		
IDC53T	*DAAM1*	p.S88T	Missense_Mutation	14	59758014	G	C	28	14.29%	Deleterious	PASSENGER/OTHER	Passenger	passenger	.	.	0.82	0.62065	29%	Clonal	.	.	.	.	WES		
IDC53T	*DACT1*	p.A798G	Missense_Mutation	14	59113734	C	G	135	36.30%	Neutral	PASSENGER/OTHER	Passenger	passenger	.	.	1	0.96358	89%	Clonal	.	.	.	.	WES		
IDC53T	*DAPK1*	p.Q1197H	Missense_Mutation	9	90321577	G	T	94	5.32%	Deleterious	PASSENGER/OTHER	Passenger	passenger	.	loh	0.25	0.00035	11%	Subclonal	.	.	.	.	WES		
IDC53T	*DDX43*	p.D227V	Missense_Mutation	6	74115431	A	T	25	16.00%	Deleterious	PASSENGER/OTHER	Passenger	passenger	.	.	0.76	0.58623	28%	Clonal	.	.	.	.	WES		
IDC53T	*DISP1*	p.M988L	Missense_Mutation	1	223177701	A	T	101	16.83%	Deleterious	PASSENGER/OTHER	Passenger	passenger	.	.	0.97	0.77742	57%	Clonal	.	.	.	.	WES		
IDC53T	*DMPK*	p.A49S	Missense_Mutation	19	46283173	C	A	100	45.00%	Neutral	PASSENGER/OTHER	Passenger	likely_pathogenic	.	.	1	0.96006	89%	Clonal	.	.	.	.	WES		
IDC53T	*DMRT1*	p.H18R	Missense_Mutation	9	841891	A	G	140	35.71%	Neutral	PASSENGER/OTHER	Passenger	passenger	.	loh	1	0.91897	88%	Clonal	.	.	.	.	WES		
IDC53T	*DMRTA2*	p.R87C	Missense_Mutation	1	50886950	G	A	89	11.24%	Deleterious	PASSENGER/OTHER	Passenger	passenger	.	.	0.54	0.11657	29%	Subclonal	.	.	.	.	WES		
IDC53T	*DNAH14*	p.M1212R	Missense_Mutation	1	225284881	T	G	35	34.29%	Deleterious	PASSENGER/OTHER	Passenger	passenger	.	.	1	0.93255	68%	Clonal	.	.	.	.	WES		
IDC53T	*DNAH17*	p.G1851S	Missense_Mutation	17	76496434	C	T	92	32.61%	Deleterious	PASSENGER/OTHER	Passenger	passenger	.	.	1	0.94396	84%	Clonal	.	.	.	.	WES		
IDC53T	*DNAH8*	p.L1919F	Missense_Mutation	6	38831746	G	C	27	40.74%	Deleterious	PASSENGER/OTHER	Passenger	passenger	.	loh	1	0.88504	63%	Clonal	.	.	.	.	WES		
IDC53T	*DNAJC27*	p.W152C	Missense_Mutation	2	25179984	C	A	34	8.82%	Deleterious	CANCER	Passenger	likely_pathogenic	.	.	0.42	0.22101	15%	Subclonal	.	.	.	.	WES		
IDC53T	*DOLK*	p.R322Q	Missense_Mutation	9	131708618	C	T	58	24.14%	Deleterious	PASSENGER/OTHER	Passenger	passenger	.	loh	1	0.82894	60%	Clonal	.	.	.	.	WES		
IDC53T	*DPH1*	p.F172L	Missense_Mutation	17	1939921	T	C	66	25.76%	Deleterious	PASSENGER/OTHER	Passenger	passenger	.	loh	0.71	0.43734	46%	Subclonal	.	.	.	.	WES		
IDC53T	*DRC1*	p.R51T	Missense_Mutation	2	26625009	G	C	93	22.58%	Deleterious	PASSENGER/OTHER	Passenger	passenger	.	.	1	0.84209	65%	Clonal	.	.	.	.	WES		
IDC53T	*ELOVL4*	p.F156L	Missense_Mutation	6	80631415	G	C	57	22.81%	Deleterious	PASSENGER/OTHER	Passenger	passenger	.	.	1	0.80727	57%	Clonal	.	.	.	.	WES		
IDC53T	*ENPP2*	p.E626G	Missense_Mutation	8	120594665	T	C	92	5.43%	Neutral	PASSENGER/OTHER	Passenger	passenger	.	.	0.64	0.47288	26%	Subclonal	.	.	.	.	WES		
IDC53T	*ESRRA*	p.G62V	Missense_Mutation	11	64074836	G	T	46	17.39%	Deleterious	PASSENGER/OTHER	Passenger	passenger	.	.	0.83	0.64458	40%	Clonal	.	.	.	.	WES		
IDC53T	*FAM120C*	p.A27T	Missense_Mutation	X	54209553	C	T	94	25.53%	Deleterious	PASSENGER/OTHER	Passenger	passenger	.	.	1	0.8783	72%	Clonal	.	.	.	.	WES		
IDC53T	*FAM172A*	p.K210N	Missense_Mutation	5	93217332	C	G	40	22.50%	Deleterious	PASSENGER/OTHER	Passenger	passenger	.	.	1	0.77304	50%	Clonal	.	.	.	.	WES		
IDC53T	*FAM222B*	p.N64D	Missense_Mutation	17	27086787	T	C	129	19.38%	Deleterious	PASSENGER/OTHER	Passenger	passenger	.	.	0.92	0.76811	61%	Clonal	.	.	.	.	WES		
IDC53T	*FAM43B*	p.D46N	Missense_Mutation	1	20879602	G	A	132	67.42%	Deleterious	.	Passenger	passenger	.	loh	1	0	95%	Clonal	.	.	.	.	WES		
IDC53T	*FASN*	p.A2116T	Missense_Mutation	17	80039537	C	T	85	54.12%	Neutral	PASSENGER/OTHER	Passenger	passenger	.	.	1	0.25531	91%	Clonal	.	.	.	.	WES		
IDC53T	*FASTK*	p.A228V	Missense_Mutation	7	150775931	G	A	99	51.52%	Deleterious	PASSENGER/OTHER	Passenger	passenger	.	loh	1	0.97201	90%	Clonal	.	.	.	.	WES		
IDC53T	*FBXO34*	p.S134F	Missense_Mutation	14	55817509	C	T	51	45.10%	Deleterious	PASSENGER/OTHER	Passenger	passenger	.	.	1	0.84566	82%	Clonal	.	.	.	.	WES		
IDC53T	*FGF3*	p.R144H	Missense_Mutation	11	69625362	C	T	190	18.95%	Neutral	PASSENGER/OTHER	Passenger	passenger	.	.	0.9	0.75547	65%	Clonal	.	.	.	YES	WES		
IDC53T	*FLVCR1*	p.Y319D	Missense_Mutation	1	213046091	T	G	76	6.58%	Deleterious	PASSENGER/OTHER	Passenger	passenger	.	.	0.38	0.04863	16%	Subclonal	.	.	.	.	WES		
IDC53T	*FLYWCH1*	p.E128K	Missense_Mutation	16	2980470	G	A	15	26.67%	Neutral	.	Passenger	passenger	.	.	1	0.80535	38%	Clonal	.	.	.	.	WES		
IDC53T	*GFY*	p.R351W	Missense_Mutation	19	49930738	C	T	138	7.25%	.	.	Passenger	passenger	.	.	0.35	0.00019	19%	Subclonal	.	.	.	.	WES		
IDC53T	*GLTSCR1*	p.G168W	Missense_Mutation	19	48182929	G	T	78	16.67%	Deleterious	PASSENGER/OTHER	Passenger	passenger	.	.	0.79	0.60663	46%	Clonal	.	.	.	.	WES		
IDC53T	*GSDMA*	p.Q347R	Missense_Mutation	17	38132195	A	G	42	47.62%	Neutral	PASSENGER/OTHER	Passenger	passenger	.	.	1	0.93526	80%	Clonal	.	.	.	.	WES		
IDC53T	*GSK3A*	p.G57C	Missense_Mutation	19	42746449	C	A	13	23.08%	Deleterious	PASSENGER/OTHER	Passenger	passenger	.	.	1	0.70449	29%	Clonal	.	.	.	.	WES		
IDC53T	*HBP1*	p.M10I	Missense_Mutation	7	106820368	G	T	42	11.90%	Deleterious	CANCER	Passenger	likely_pathogenic	.	.	0.57	0.35675	24%	Subclonal	.	.	.	.	WES		
IDC53T	*HOMER3*	p.Q216H	Missense_Mutation	19	19042827	C	G	45	26.67%	Deleterious	PASSENGER/OTHER	Passenger	passenger	.	.	1	0.83935	60%	Clonal	.	.	.	.	WES		
IDC53T	*HRNR*	p.Y2849F	Missense_Mutation	1	152185559	T	A	35	48.57%	Neutral	PASSENGER/OTHER	Passenger	passenger	.	.	1	0.92739	77%	Clonal	.	.	.	.	WES		
IDC53T	*HSPA4*	p.A426V	Missense_Mutation	5	132425286	C	T	42	9.52%	Deleterious	PASSENGER/OTHER	Passenger	passenger	.	.	0.45	0.20673	18%	Subclonal	.	.	.	.	WES		
IDC53T	*INADL*	p.S1338N	Missense_Mutation	1	62503702	G	A	18	50.00%	Neutral	PASSENGER/OTHER	Passenger	passenger	.	.	1	0.90858	64%	Clonal	.	.	.	.	WES		
IDC53T	*KALRN*	p.Q1618H	Missense_Mutation	3	124215185	G	T	47	12.77%	Deleterious	PASSENGER/OTHER	Passenger	passenger	.	.	0.61	0.39256	28%	Subclonal	.	.	.	.	WES		
IDC53T	*KCNB1*	p.P566L	Missense_Mutation	20	47990400	G	A	173	30.64%	Deleterious	PASSENGER/OTHER	Passenger	passenger	.	.	1	0.61083	91%	Clonal	.	.	.	.	WES		
IDC53T	*KCNJ9*	p.A177T	Missense_Mutation	1	160054349	G	A	174	18.39%	Deleterious	PASSENGER/OTHER	Passenger	passenger	.	.	0.88	0.71396	62%	Clonal	.	.	.	.	WES		
IDC53T	*KCNN3*	p.T627S	Missense_Mutation	1	154685960	T	A	95	29.47%	Deleterious	.	Passenger	passenger	.	.	1	0.86932	78%	Clonal	.	.	.	.	WES		
IDC53T	*KCNU1*	p.Y1056H	Missense_Mutation	8	36793154	T	C	78	6.41%	Neutral	PASSENGER/OTHER	Passenger	passenger	.	.	0.31	0.00478	13%	Subclonal	.	.	.	.	WES		
IDC53T	*KIAA1045*	p.G342V	Missense_Mutation	9	34977557	G	T	57	21.05%	Deleterious	.	Passenger	passenger	.	loh	1	0.77168	53%	Clonal	.	.	.	.	WES		
IDC53T	*KIF23*	p.R253C	Missense_Mutation	15	69718431	C	T	12	25.00%	Deleterious	PASSENGER/OTHER	Passenger	passenger	.	loh	0.69	0.61587	23%	Clonal	.	.	.	.	WES		
IDC53T	*KLHL14*	p.C490F	Missense_Mutation	18	30260251	C	A	49	20.41%	Deleterious	PASSENGER/OTHER	Passenger	passenger	.	loh	0.77	0.57837	42%	Clonal	.	.	.	.	WES		
IDC53T	*KLRF1*	p.M183K	Missense_Mutation	12	9994990	T	A	41	31.71%	Neutral	PASSENGER/OTHER	Passenger	passenger	.	.	1	0.88611	66%	Clonal	.	.	.	.	WES		
IDC53T	*KRT84*	p.S49R	Missense_Mutation	12	52779223	G	T	122	18.85%	Neutral	PASSENGER/OTHER	Passenger	passenger	.	.	0.9	0.73931	59%	Clonal	.	.	.	.	WES		
IDC53T	*LHX5*	p.A24T	Missense_Mutation	12	113909234	C	T	162	16.05%	Deleterious	PASSENGER/OTHER	Passenger	passenger	.	loh	0.93	0.76903	62%	Clonal	.	.	.	.	WES		
IDC53T	*LRRC45*	p.Q349H	Missense_Mutation	17	79986022	G	C	78	12.82%	Deleterious	PASSENGER/OTHER	Passenger	passenger	.	.	0.87	0.67832	45%	Clonal	.	.	.	.	WES		
IDC53T	*LRRC56*	p.A217E	Missense_Mutation	11	551156	C	A	48	60.42%	Deleterious	PASSENGER/OTHER	Passenger	passenger	.	loh	1	0.26546	84%	Subclonal	.	.	.	.	WES		
IDC53T	*MACF1*	p.E3649G	Missense_Mutation	1	39896375	A	G	9	44.44%	Deleterious	PASSENGER/OTHER	Passenger	passenger	.	.	1	0.85824	42%	Clonal	.	.	.	.	WES		
IDC53T	*MAST4*	p.V1524L	Missense_Mutation	5	66459577	G	T	161	9.32%	Neutral	PASSENGER/OTHER	Passenger	passenger	.	.	0.44	0.0013	27%	Subclonal	.	.	.	.	WES		
IDC53T	*MCIDAS*	p.C291R	Missense_Mutation	5	54516481	A	G	127	49.61%	Deleterious	.	Passenger	passenger	.	.	1	0.87862	93%	Clonal	.	.	.	.	WES		
IDC53T	*MCM4*	p.A540T	Missense_Mutation	8	48883254	G	A	40	37.50%	Deleterious	PASSENGER/OTHER	Passenger	passenger	.	.	1	0.93712	71%	Clonal	.	.	.	.	WES		
IDC53T	*MECP2*	p.H379P	Missense_Mutation	X	153296179	T	G	76	15.79%	Deleterious	PASSENGER/OTHER	Passenger	passenger	.	.	0.75	0.54676	43%	Clonal	.	.	.	.	WES		
IDC53T	*MTOR*	p.S1785N	Missense_Mutation	1	11193147	C	T	40	32.50%	Deleterious	CANCER	Passenger	likely_pathogenic	.	loh	0.9	0.78728	52%	Clonal	.	TRUE	TRUE	YES	WES		
IDC53T	*MUC5B*	p.Q709H	Missense_Mutation	11	1254304	G	T	197	27.92%	Neutral	PASSENGER/OTHER	Passenger	passenger	.	loh	0.77	0.23945	61%	Subclonal	.	.	.	.	WES		
IDC53T	*MYH7*	p.P151S	Missense_Mutation	14	23901899	G	A	42	7.14%	Deleterious	PASSENGER/OTHER	Passenger	passenger	.	.	0.34	0.08942	12%	Subclonal	.	.	.	.	WES		
IDC53T	*MYOC*	p.V222G	Missense_Mutation	1	171607802	A	C	41	19.51%	Deleterious	PASSENGER/OTHER	Passenger	passenger	.	.	0.93	0.70888	44%	Clonal	.	.	.	.	WES		
IDC53T	*NDN*	p.K233M	Missense_Mutation	15	23931667	T	A	68	19.12%	Neutral	PASSENGER/OTHER	Passenger	passenger	.	.	0.91	0.72627	51%	Clonal	.	.	.	.	WES		
IDC53T	*NEK10*	p.E203K	Missense_Mutation	3	27352469	C	T	18	33.33%	Deleterious	PASSENGER/OTHER	Passenger	passenger	.	.	1	0.85256	49%	Clonal	.	.	.	.	WES		
IDC53T	*NKX2-4*	p.L228Q	Missense_Mutation	20	21376931	A	T	154	20.13%	Deleterious	CANCER	Passenger	likely_pathogenic	.	.	0.96	0.8099	66%	Clonal	.	.	.	.	WES		
IDC53T	*NLRC4*	p.I20T	Missense_Mutation	2	32477691	A	G	34	38.24%	Neutral	PASSENGER/OTHER	Passenger	passenger	.	.	1	0.93435	69%	Clonal	.	.	.	.	WES		
IDC53T	*NLRP4*	p.D80V	Missense_Mutation	19	56363685	A	T	16	62.50%	Neutral	PASSENGER/OTHER	Passenger	passenger	.	.	1	0.77354	69%	Clonal	.	.	.	.	WES		
IDC53T	*NPY4R*	p.H5Q	Missense_Mutation	10	47086798	C	A	146	10.96%	Neutral	PASSENGER/OTHER	Passenger	passenger	.	.	0.52	0.01854	32%	Subclonal	.	.	.	.	WES		
IDC53T	*OPHN1*	p.H506Q	Missense_Mutation	X	67331704	G	T	55	36.36%	Deleterious	PASSENGER/OTHER	Passenger	passenger	.	.	1	0.93844	76%	Clonal	.	.	.	.	WES		
IDC53T	*OR10W1*	p.H184P	Missense_Mutation	11	58034780	T	G	57	12.28%	Neutral	PASSENGER/OTHER	Passenger	passenger	.	.	0.59	0.31749	28%	Subclonal	.	.	.	.	WES		
IDC53T	*OR1N2*	p.L230M	Missense_Mutation	9	125316136	C	A	106	12.26%	Neutral	PASSENGER/OTHER	Passenger	passenger	.	loh	0.58	0.1439	34%	Subclonal	.	.	.	.	WES		
IDC53T	*OR2F2*	p.E271G	Missense_Mutation	7	143633137	A	G	97	13.40%	Neutral	PASSENGER/OTHER	Passenger	passenger	.	.	0.64	0.2774	38%	Subclonal	.	.	.	.	WES		
IDC53T	*OR5A2*	p.T241P	Missense_Mutation	11	59189706	T	G	61	8.20%	Deleterious	PASSENGER/OTHER	Passenger	passenger	.	.	0.39	0.05532	17%	Subclonal	.	.	.	.	WES		
IDC53T	*OR5D14*	p.E209D	Missense_Mutation	11	55563658	G	T	84	8.33%	Neutral	PASSENGER/OTHER	Passenger	passenger	.	.	0.4	0.0187	19%	Subclonal	.	.	.	.	WES		
IDC53T	*OSBPL2*	p.A344S	Missense_Mutation	20	60861672	G	T	316	8.54%	Neutral	PASSENGER/OTHER	Passenger	passenger	.	.	0.83	0.64446	57%	Clonal	.	.	.	.	WES		
IDC53T	*OTUB2*	p.S59Y	Missense_Mutation	14	94505385	C	A	68	20.59%	Deleterious	PASSENGER/OTHER	Passenger	passenger	.	loh	0.98	0.77303	55%	Clonal	.	.	.	.	WES		
IDC53T	*PAX9*	p.G256R	Missense_Mutation	14	37135801	G	C	77	12.99%	Neutral	PASSENGER/OTHER	Passenger	passenger	.	.	0.75	0.55641	40%	Clonal	.	.	.	.	WES		
IDC53T	*PCDH12*	p.I450M	Missense_Mutation	5	141336067	G	C	114	10.53%	Neutral	PASSENGER/OTHER	Passenger	passenger	.	loh	0.71	0.48048	40%	Subclonal	.	.	.	.	WES		
IDC53T	*PCDH19*	p.V493M	Missense_Mutation	X	99662119	C	T	168	41.67%	Deleterious	PASSENGER/OTHER	Passenger	passenger	.	.	1	0.97415	92%	Clonal	.	.	.	.	WES		
IDC53T	*PCDHA1*	p.A482T	Missense_Mutation	5	140167319	G	A	27	29.63%	Neutral	PASSENGER/OTHER	Passenger	passenger	.	loh	1	0.90499	58%	Clonal	.	.	.	.	WES		
IDC53T	*PCDHB1*	p.Q744H	Missense_Mutation	5	140433287	A	T	64	18.75%	Neutral	PASSENGER/OTHER	Passenger	passenger	.	loh	1	0.83487	59%	Clonal	.	.	.	.	WES		
IDC53T	*PCDHGA4*	p.V697G	Missense_Mutation	5	140736857	T	G	232	18.97%	.	PASSENGER/OTHER	Passenger	passenger	.	loh	1	0.89575	81%	Clonal	.	.	.	.	WES		
IDC53T	*PDE6B*	p.D375H	Missense_Mutation	4	650678	G	C	101	24.75%	Deleterious	PASSENGER/OTHER	Passenger	passenger	.	loh	1	0.94175	81%	Clonal	.	.	.	.	WES		
IDC53T	*PIP5K1A*	p.Y437C	Missense_Mutation	1	151212462	A	G	31	22.58%	Deleterious	CANCER	Passenger	likely_pathogenic	.	.	1	0.7546	45%	Clonal	.	.	.	.	WES		
IDC53T	*PLCE1*	p.I1298T	Missense_Mutation	10	96022329	T	C	83	13.25%	Deleterious	PASSENGER/OTHER	Passenger	passenger	.	loh	0.5	0.03574	28%	Subclonal	.	.	.	.	WES		
IDC53T	*PLXNA2*	p.D1155G	Missense_Mutation	1	208219254	T	C	62	25.81%	Deleterious	PASSENGER/OTHER	Passenger	passenger	.	.	1	0.88709	69%	Clonal	.	.	.	.	WES		
IDC53T	*PLXNB3*	p.V162M	Missense_Mutation	X	153032697	G	A	105	15.24%	Neutral	PASSENGER/OTHER	Passenger	passenger	.	.	0.73	0.45453	45%	Subclonal	.	.	.	.	WES		
IDC53T	*PPAPDC3*	p.R234H	Missense_Mutation	9	134183559	G	A	148	42.57%	Deleterious	PASSENGER/OTHER	Passenger	passenger	.	loh	1	0.97224	92%	Clonal	.	.	.	.	WES		
IDC53T	*PRRC2C*	p.I1893V	Missense_Mutation	1	171526934	A	G	77	11.69%	Neutral	PASSENGER/OTHER	Passenger	passenger	.	.	0.56	0.18919	30%	Subclonal	.	.	.	.	WES		
IDC53T	*PTH2R*	p.P537S	Missense_Mutation	2	209358340	C	T	73	27.40%	Neutral	PASSENGER/OTHER	Passenger	passenger	.	.	1	0.87277	70%	Clonal	.	.	.	.	WES		
IDC53T	*RIN3*	p.R138T	Missense_Mutation	14	93081797	G	C	128	11.72%	Deleterious	PASSENGER/OTHER	Passenger	passenger	.	loh	0.56	0.06202	34%	Subclonal	.	.	.	.	WES		
IDC53T	*RLTPR*	p.P476T	Missense_Mutation	16	67682392	C	A	25	16.00%	Neutral	PASSENGER/OTHER	Passenger	passenger	.	.	0.92	0.66434	31%	Clonal	.	.	.	.	WES		
IDC53T	*SDPR*	p.V418M	Missense_Mutation	2	192700675	C	T	121	20.66%	Deleterious	PASSENGER/OTHER	Passenger	passenger	.	.	0.98	0.81459	64%	Clonal	.	.	.	.	WES		
IDC53T	*SIK2*	p.P544S	Missense_Mutation	11	111591336	C	T	68	63.24%	Deleterious	PASSENGER/OTHER	Passenger	passenger	.	loh	1	0.0052	89%	Subclonal	.	.	.	.	WES		
IDC53T	*SLC10A4*	p.L245R	Missense_Mutation	4	48487092	T	G	75	5.33%	Deleterious	PASSENGER/OTHER	Passenger	passenger	.	.	0.25	0.00203	10%	Subclonal	.	.	.	.	WES		
IDC53T	*SLC13A1*	p.L375F	Missense_Mutation	7	122768909	G	A	12	50.00%	Deleterious	PASSENGER/OTHER	Passenger	passenger	.	.	1	0.89138	54%	Clonal	.	.	.	.	WES		
IDC53T	*SLC22A8*	p.R319Q	Missense_Mutation	11	62763221	C	T	24	12.50%	Deleterious	PASSENGER/OTHER	Passenger	passenger	.	.	0.6	0.46262	20%	Subclonal	.	.	.	.	WES		
IDC53T	*SLC30A5*	p.A154T	Missense_Mutation	5	68408979	G	A	12	25.00%	Deleterious	PASSENGER/OTHER	Passenger	passenger	.	.	1	0.72556	30%	Clonal	.	.	.	.	WES		
IDC53T	*SLC35A5*	p.D203H	Missense_Mutation	3	112299571	G	C	71	38.03%	Neutral	PASSENGER/OTHER	Passenger	passenger	.	.	1	0.95222	82%	Clonal	.	.	.	.	WES		
IDC53T	*SLC35B1*	p.T268I	Missense_Mutation	17	47780333	G	A	57	38.60%	Deleterious	PASSENGER/OTHER	Passenger	passenger	.	.	1	0.95117	79%	Clonal	.	.	.	.	WES		
IDC53T	*SLC43A3*	p.S78Y	Missense_Mutation	11	57193095	G	T	37	27.03%	Deleterious	PASSENGER/OTHER	Driver	likely_pathogenic	.	.	1	0.83056	57%	Clonal	.	.	.	.	WES		
IDC53T	*SLC47A1*	p.A243D	Missense_Mutation	17	19458992	C	A	71	36.62%	Neutral	PASSENGER/OTHER	Passenger	passenger	.	.	1	0.94303	81%	Clonal	.	.	.	.	WES		
IDC53T	*SLFN13*	p.T197N	Missense_Mutation	17	33772110	G	T	49	16.33%	Neutral	PASSENGER/OTHER	Passenger	passenger	.	.	0.78	0.59849	38%	Clonal	.	.	.	.	WES		
IDC53T	*SLFNL1*	p.L259I	Missense_Mutation	1	41483489	G	T	152	19.08%	Neutral	PASSENGER/OTHER	Passenger	passenger	.	.	0.91	0.7585	63%	Clonal	.	.	.	.	WES		
IDC53T	*SLTM*	p.V954L	Missense_Mutation	15	59175961	C	G	53	49.06%	Deleterious	PASSENGER/OTHER	Passenger	passenger	.	.	1	0.9252	84%	Clonal	.	.	.	.	WES		
IDC53T	*SPINT2*	p.F37V	Missense_Mutation	19	38774269	T	G	84	14.29%	Neutral	PASSENGER/OTHER	Passenger	passenger	.	.	0.68	0.40316	39%	Subclonal	.	.	.	.	WES		
IDC53T	*SRRM4*	p.R209Q	Missense_Mutation	12	119568494	G	A	27	29.63%	Neutral	PASSENGER/OTHER	Passenger	passenger	.	loh	1	0.88815	56%	Clonal	.	.	.	.	WES		
IDC53T	*STAC3*	p.N114K	Missense_Mutation	12	57642579	G	C	53	13.21%	Deleterious	PASSENGER/OTHER	Passenger	passenger	.	.	0.63	0.40221	30%	Subclonal	.	.	.	.	WES		
IDC53T	*STARD9*	p.P2765L	Missense_Mutation	15	42982070	C	T	76	44.74%	Neutral	PASSENGER/OTHER	Passenger	passenger	.	.	1	0.95747	86%	Clonal	.	.	.	.	WES		
IDC53T	*SYNPO2L*	p.P918R	Missense_Mutation	10	75406657	G	C	72	9.72%	Deleterious	PASSENGER/OTHER	Passenger	passenger	.	.	0.46	0.08298	23%	Subclonal	.	.	.	.	WES		
IDC53T	*TCTE3*	p.V151E	Missense_Mutation	6	170143210	A	T	13	30.77%	Deleterious	PASSENGER/OTHER	Passenger	passenger	.	loh	0.85	0.71154	31%	Clonal	.	.	.	.	WES		
IDC53T	*TEX10*	p.H908Y	Missense_Mutation	9	103064541	G	A	37	32.43%	Deleterious	.	Passenger	passenger	.	loh	1	0.89	64%	Clonal	.	.	.	.	WES		
IDC53T	*TGFB1I1*	p.Q104E	Missense_Mutation	16	31485283	C	G	84	10.71%	Deleterious	PASSENGER/OTHER	Passenger	passenger	.	.	0.51	0.09919	27%	Subclonal	.	.	.	.	WES		
IDC53T	*TMEM177*	p.Y80H	Missense_Mutation	2	120438667	T	C	121	34.71%	Deleterious	PASSENGER/OTHER	Passenger	passenger	.	.	1	0.89868	86%	Clonal	.	.	.	.	WES		
IDC53T	*TMEM95*	p.L164P	Missense_Mutation	17	7259768	T	C	74	67.57%	Deleterious	.	Passenger	passenger	.	loh	1	0.00005	91%	Clonal	.	.	.	.	WES		
IDC53T	*TMPRSS13*	p.Q83R	Missense_Mutation	11	117789327	T	C	11	27.27%	Neutral	PASSENGER/OTHER	Passenger	passenger	.	loh	0.75	0.65532	24%	Clonal	.	.	.	.	WES		
IDC53T	*TNR*	p.N72I	Missense_Mutation	1	175375636	T	A	99	6.06%	Deleterious	PASSENGER/OTHER	Driver	likely_pathogenic	.	.	0.29	0.00061	13%	Subclonal	.	.	.	.	WES		
IDC53T	*TTC17*	p.S169R	Missense_Mutation	11	43413102	T	A	30	46.67%	Deleterious	PASSENGER/OTHER	Passenger	passenger	.	.	1	0.93361	73%	Clonal	.	.	.	.	WES		
IDC53T	*TTN*	p.E7228K	Missense_Mutation	2	179588145	C	T	37	8.11%	Deleterious	PASSENGER/OTHER	Passenger	passenger	.	loh	0.31	0.03899	11%	Subclonal	.	.	.	.	WES		
IDC53T	*TTN*	p.S35769T	Missense_Mutation	2	179393072	C	G	46	15.22%	Neutral	PASSENGER/OTHER	Passenger	passenger	.	loh	0.57	0.27667	28%	Subclonal	.	.	.	.	WES		
IDC53T	*TTN*	p.S9166W	Missense_Mutation	2	179577152	G	C	50	16.00%	Deleterious	PASSENGER/OTHER	Passenger	passenger	.	loh	0.6	0.30573	31%	Subclonal	.	.	.	.	WES		
IDC53T	*UNC13C*	p.D1581V	Missense_Mutation	15	54685274	A	T	30	10.00%	Deleterious	PASSENGER/OTHER	Passenger	passenger	.	.	0.48	0.31314	16%	Subclonal	.	.	.	.	WES		
IDC53T	*USP34*	p.R2698H	Missense_Mutation	2	61441784	C	T	35	8.57%	Deleterious	PASSENGER/OTHER	Passenger	passenger	.	.	0.41	0.20029	14%	Subclonal	.	.	.	.	WES		
IDC53T	*VWA5A*	p.K128M	Missense_Mutation	11	123989032	A	T	54	11.11%	Neutral	PASSENGER/OTHER	Passenger	passenger	.	loh	0.31	0.00071	14%	Subclonal	.	.	.	.	WES		
IDC53T	*ZBTB1*	p.G642A	Missense_Mutation	14	64990147	G	C	25	32.00%	Deleterious	PASSENGER/OTHER	Passenger	passenger	.	loh	1	0.8633	55%	Clonal	.	.	.	.	WES		
IDC53T	*ZFPM2*	p.A711D	Missense_Mutation	8	106814442	C	A	233	5.58%	Deleterious	PASSENGER/OTHER	Passenger	passenger	.	.	0.66	0.35303	38%	Subclonal	.	.	.	.	WES		
IDC53T	*ZNF219*	p.D301G	Missense_Mutation	14	21560554	T	C	134	12.69%	Deleterious	PASSENGER/OTHER	Passenger	passenger	.	.	0.6	0.11298	38%	Subclonal	.	.	.	.	WES		
IDC53T	*ZNF287*	p.D178H	Missense_Mutation	17	16467109	C	G	37	21.62%	Deleterious	PASSENGER/OTHER	Passenger	passenger	.	loh	0.6	0.33469	31%	Subclonal	.	.	.	.	WES		
IDC53T	*ZNF518B*	p.N456D	Missense_Mutation	4	10446587	T	C	76	5.26%	Neutral	PASSENGER/OTHER	Passenger	passenger	.	loh	0.46	0.24171	18%	Subclonal	.	.	.	.	WES		
IDC53T	*ZNF831*	p.Q1634E	Missense_Mutation	20	57829664	C	G	121	9.92%	Neutral	PASSENGER/OTHER	Passenger	passenger	.	.	0.97	0.74553	51%	Clonal	.	.	.	.	WES		
IDC53T	*BRAF*	p.S571*	Nonsense_Mutation	7	140454016	G	C	27	18.52%	Deleterious	.	.	likely_pathogenic	.	.	0.88	0.65949	34%	Clonal	TRUE	TRUE	TRUE	YES	WES		
IDC53T	*CNTNAP4*	p.W247*	Nonsense_Mutation	16	76482736	G	A	22	50.00%	Deleterious	.	.	passenger	.	.	1	0.80542	70%	Clonal	.	.	.	.	WES		
IDC53T	*LEFTY2*	p.Q189*	Nonsense_Mutation	1	226127233	G	A	274	3.28%	Deleterious	.	.	passenger	.	.	0.19	0	10%	Subclonal	.	.	.	.	WES		
IDC53T	*LRRC73*	p.E128*	Nonsense_Mutation	6	43476549	C	A	51	29.41%	Deleterious	.	.	likely_pathogenic	.	loh	1	0.83006	61%	Clonal	.	.	.	.	WES		
IDC53T	*PALB2*	p.Q61*	Nonsense_Mutation	16	23649201	G	A	27	48.15%	Deleterious	.	.	likely_pathogenic	.	.	1	0.92564	72%	Clonal	TRUE	.	.	YES	WES	TCACACTGTGGGAAAAAGAACA	TTTGTCTCCTCTAGCGTGCC
IDC53T	*PPP2R2C*	p.C330*	Nonsense_Mutation	4	6331051	A	T	65	13.85%	Deleterious	.	.	likely_pathogenic	.	loh	1	0.80138	52%	Clonal	.	.	.	.	WES		
IDC53T	*PSD2*	p.E27*	Nonsense_Mutation	5	139189104	G	T	91	67.03%	Deleterious	.	.	passenger	.	loh	1	0.19545	94%	Clonal	.	.	.	.	WES		
IDC53T	*RYR1*	p.Q1279*	Nonsense_Mutation	19	38964086	C	T	116	5.17%	Deleterious	.	.	likely_pathogenic	.	.	0.25	0.00003	11%	Subclonal	.	.	.	.	WES		
IDC53T	*SCNN1D*	p.G784*	Nonsense_Mutation	1	1226931	G	T	69	27.54%	Deleterious	.	.	likely_pathogenic	.	loh	0.76	0.56271	50%	Clonal	.	.	.	.	WES		
IDC53T	*ZNF446*	p.E221*	Nonsense_Mutation	19	58991043	G	T	190	22.11%	Neutral	.	.	passenger	.	.	1	0.88169	74%	Clonal	.	.	.	.	WES		
IDC53T	*ANK1*	p.X1151_splice	Splice_Site	8	41551621	C	A	43	51.16%	Deleterious	.	.	passenger	.	.	1	0.90252	82%	Clonal	.	.	.	.	WES		
IDC53T	*CLEC4G*	p.X74_splice	Splice_Site	19	7795976	T	C	111	6.31%	Deleterious	.	.	passenger	.	.	0.3	0.00033	14%	Subclonal	.	.	.	.	WES		
IDC53T	*SLC4A8*	p.X670_splice	Splice_Site	12	51868232	G	T	18	22.22%	Deleterious	PASSENGER/OTHER	Passenger	passenger	.	.	1	0.71107	34%	Clonal	.	.	.	.	WES		
IDC53T	*SYT15*	p.X119_splice	Splice_Site	10	46968580	C	A	35	20.00%	Deleterious	.	.	passenger	.	.	0.95	0.71102	42%	Clonal	.	.	.	.	WES		
IDC53T	*TNPO3*	p.X386_splice	Splice_Site	7	128637442	C	G	36	13.89%	Deleterious	.	.	passenger	.	.	0.66	0.49159	28%	Subclonal	.	.	.	.	WES		
IDC53T	*USP34*	p.X2369_splice	Splice_Site	2	61459570	AACATATTTTTCCTTACCTGTCTC	A	29	20.69%	.	.	.	passenger	.	.	0.99	0.71373	40%	Clonal	.	.	.	.	WES		
IDC53T	*VPS13D*	p.X2876_splice	Splice_Site	1	12398363	G	T	25	16.00%	Deleterious	.	.	likely_pathogenic	.	loh	0.44	0.19041	18%	Subclonal	.	.	.	.	WES		
IDC55T	*EXOC6B*	p.F599Lfs*16	Frame_Shift_Del	2	72707747	TA	T	89	40.45%	Deleterious	.	.	passenger	.	.	1	0.89139	76%	Clonal	.	.	.	.	WES		
IDC55T	*KMT2C*	p.K4215*	Frame_Shift_Del	7	151848543	AGATCTTT	A	115	29.57%	Deleterious	.	.	likely_pathogenic	.	loh	0.79	0.42462	58%	Clonal	TRUE	TRUE	TRUE	.	WES		
IDC55T	*NIPBL*	p.E1176Kfs*13	Frame_Shift_Del	5	37000935	GA	G	64	39.06%	Deleterious	.	.	passenger	.	.	1	0.85373	69%	Clonal	.	.	.	.	WES		
IDC55T	*TMC1*	p.E12Kfs*27	Frame_Shift_Del	9	75303640	AG	A	73	34.25%	Deleterious	.	.	passenger	.	.	0.92	0.75915	63%	Clonal	.	.	.	.	WES		
IDC55T	*KMT2D*	p.P1855Sfs*7	Frame_Shift_Ins	12	49436940	G	GGCACTGA	135	34.81%	Deleterious	.	.	likely_pathogenic	.	loh	0.58	0	45%	Clonal	TRUE	TRUE	TRUE	.	WES		
IDC55T	*PRTN3*	p.D210Vfs*8	Frame_Shift_Ins	19	847822	C	CTG	193	33.16%	Deleterious	.	.	passenger	.	.	1	0.49226	89%		.	.	.	.	WES		
IDC55T	*C17orf70*	p.Q169_G171del	In_Frame_Del	17	79518007	ACCGATCTGG	A	346	27.75%	Neutral	.	.	passenger	.	.	1	0.91049	82%	Clonal	.	.	.	.	WES		
IDC55T	*HELZ*	p.R459del	In_Frame_Del	17	65174828	CCGT	C	185	27.03%	Deleterious	.	.	passenger	.	.	0.72	0.04808	56%	Clonal	.	.	.	.	WES		
IDC55T	*HIST1H3J*	p.K65_R70del	In_Frame_Del	6	27858360	GGCGCTGAAATGGCAGTTT	G	479	14.61%	.	.	.	passenger	.	.	0.39	0	31%	subclonal	.	.	.	YES	WES		
IDC55T	*ABCC9*	p.L850V	Missense_Mutation	12	22005397	A	C	387	2.84%	Deleterious	CANCER	Passenger	likely_pathogenic	.	.	0.1	0	5%	subclonal	.	.	.	.	WES		
IDC55T	*ADCY2*	p.E35D	Missense_Mutation	5	7396514	G	C	253	37.94%	Deleterious	PASSENGER/OTHER	Passenger	passenger	.	.	1	0.91403	83%	Clonal	.	.	.	.	WES		
IDC55T	*ARFGEF2*	p.P313H	Missense_Mutation	20	47582439	C	A	140	35.00%	Neutral	PASSENGER/OTHER	Passenger	passenger	.	.	0.94	0.80624	72%	Clonal	.	.	.	.	WES		
IDC55T	*ATP11B*	p.M80I	Missense_Mutation	3	182547465	G	C	59	22.03%	Deleterious	PASSENGER/OTHER	Passenger	passenger	.	.	0.81	0.61525	47%	subclonal	.	.	.	.	WES		
IDC55T	*BID*	p.D172E	Missense_Mutation	22	18220981	G	T	52	55.77%	Neutral	PASSENGER/OTHER	Passenger	passenger	.	loh	0.93	0.8401	69%	Clonal	.	.	.	.	WES		
IDC55T	*BROX*	p.I200T	Missense_Mutation	1	222900528	T	C	154	55.19%	Deleterious	PASSENGER/OTHER	Passenger	passenger	.	.	1	0.97595	94%	Clonal	.	.	.	.	WES		
IDC55T	*C10orf35*	p.L98P	Missense_Mutation	10	71392742	T	C	301	27.24%	Deleterious	.	Passenger	passenger	.	.	0.73	0.00526	60%	subclonal	.	.	.	.	WES		
IDC55T	*C1GALT1C1*	p.M289T	Missense_Mutation	23	119760156	A	G	254	4.33%	Deleterious	PASSENGER/OTHER	Driver	likely_pathogenic	.	.	0.12	0	6%	subclonal	.	.	.	.	WES		
IDC55T	*CASKIN1*	p.P192S	Missense_Mutation	16	2239071	G	A	296	53.38%	Deleterious	PASSENGER/OTHER	Passenger	passenger	.	loh	1	0.00004	95%	Clonal	.	.	.	.	WES		
IDC55T	*CCNDBP1*	p.A309V	Missense_Mutation	15	43486617	C	T	40	65.00%	Deleterious	PASSENGER/OTHER	Passenger	passenger	.	loh	1	0.92227	76%	Clonal	.	.	.	.	WES		
IDC55T	*CDH17*	p.T35I	Missense_Mutation	8	95201461	G	A	256	26.17%	Neutral	PASSENGER/OTHER	Passenger	passenger	.	.	0.96	0.84594	76%	subclonal	.	.	.	.	WES		
IDC55T	*CDH9*	p.P266L	Missense_Mutation	5	26906082	G	A	126	43.65%	Deleterious	CANCER	Passenger	likely_pathogenic	.	.	1	0.92544	84%	Clonal	.	.	.	.	WES		
IDC55T	*CDK19*	p.E101K	Missense_Mutation	6	110991648	C	T	133	5.26%	Deleterious	PASSENGER/OTHER	Passenger	passenger	.	.	0.14	0	6%	subclonal	.	.	.	.	WES		
IDC55T	*CIB4*	p.R107H	Missense_Mutation	2	26818052	C	T	156	38.46%	Deleterious	PASSENGER/OTHER	Passenger	passenger	.	.	1	0.89949	79%	Clonal	.	.	.	.	WES		
IDC55T	*CITED2*	p.A90E	Missense_Mutation	6	139694813	G	T	434	4.15%	Deleterious	PASSENGER/OTHER	Passenger	passenger	.	.	0.11	0	7%	subclonal	.	.	.	.	WES		
IDC55T	*COL11A1*	p.C1639S	Missense_Mutation	1	103348810	C	G	136	34.56%	Deleterious	CANCER	Passenger	likely_pathogenic	.	.	0.92	0.78714	71%	Clonal	.	.	.	.	WES		
IDC55T	*COL3A1*	p.G101A	Missense_Mutation	2	189849942	G	C	134	5.97%	Deleterious	CANCER	Passenger	likely_pathogenic	.	.	0.16	0	8%	subclonal	.	.	.	.	WES		
IDC55T	*CXorf23*	p.P656R	Missense_Mutation	23	19947955	G	C	99	35.35%	Deleterious	PASSENGER/OTHER	Passenger	passenger	.	.	0.94	0.80614	69%	Clonal	.	.	.	.	WES		
IDC55T	*CYP4F3*	p.E352G	Missense_Mutation	19	15763702	A	G	114	41.23%	Deleterious	PASSENGER/OTHER	Passenger	passenger	.	.	1	0.54067	87%	Clonal	.	.	.	.	WES		
IDC55T	*DCN*	p.F330L	Missense_Mutation	12	91539925	G	T	63	52.38%	Deleterious	PASSENGER/OTHER	Passenger	passenger	.	loh	0.88	0.73615	66%	subclonal	.	.	.	.	WES		
IDC55T	*DCSTAMP*	p.G2A	Missense_Mutation	8	105360785	G	C	304	51.32%	Neutral	PASSENGER/OTHER	Passenger	passenger	.	.	1	0.96927	96%	Clonal	.	.	.	.	WES		
IDC55T	*EDA2R*	p.T94P	Missense_Mutation	23	65824335	T	G	170	44.71%	Deleterious	PASSENGER/OTHER	Passenger	passenger	.	.	1	0.92332	88%	Clonal	.	.	.	.	WES		
IDC55T	*ERC2*	p.V569L	Missense_Mutation	3	56052996	C	A	98	6.12%	Deleterious	PASSENGER/OTHER	Passenger	passenger	.	loh	0.1	0	4%	subclonal	.	.	.	.	WES		
IDC55T	*F8*	p.L417F	Missense_Mutation	23	154194721	T	G	49	26.53%	Neutral	CANCER	Passenger	passenger	.	.	0.71	0.39922	43%		.	.	.	.	WES		
IDC55T	*FAM49B*	p.N214Y	Missense_Mutation	8	130863154	T	A	117	31.62%	Deleterious	PASSENGER/OTHER	Passenger	passenger	.	.	1	0.90058	77%	Clonal	.	.	.	.	WES		
IDC55T	*FASN*	p.I175V	Missense_Mutation	17	80051227	T	C	150	12.67%	Deleterious	PASSENGER/OTHER	Passenger	passenger	.	.	0.47	0.00033	30%	Clonal	.	.	.	.	WES		
IDC55T	*FREM1*	p.P574Q	Missense_Mutation	9	14842331	G	T	280	26.07%	Deleterious	PASSENGER/OTHER	Passenger	passenger	.	.	0.96	0.84476	76%	subclonal	.	.	.	.	WES		
IDC55T	*FXR1*	p.V272L	Missense_Mutation	3	180671562	G	T	67	16.42%	Deleterious	PASSENGER/OTHER	Passenger	passenger	.	.	0.6	0.20882	34%	Clonal	.	.	.	.	WES		
IDC55T	*GALNT11*	p.S416P	Missense_Mutation	7	151814254	T	C	119	31.93%	Deleterious	PASSENGER/OTHER	Passenger	passenger	.	loh	0.85	0.63428	64%	Clonal	.	.	.	.	WES		
IDC55T	*GPC5*	p.Y175F	Missense_Mutation	13	92345639	A	T	401	26.68%	Deleterious	PASSENGER/OTHER	Passenger	passenger	.	loh	0.98	0.88491	81%	subclonal	.	.	.	.	WES		

IDC55T	GUCY1A3	p.P636R	Missense_Mutation	4	156651217	C	G	155	54.19%	Deleterious	PASSENGER/OTHER	Passenger	passenger	.	loh	0.91	0.7587	77%	subclonal	.	.	.	.	WES		
IDC55T	HELB	p.V603L	Missense_Mutation	12	66707892	G	C	88	4.55%	Deleterious	PASSENGER/OTHER	Passenger	passenger	.	loh	0.08	0	3%	subclonal	.	.	.	.	WES		
IDC55T	HERC4	p.S792R	Missense_Mutation	10	69700850	T	G	25	60.00%	Deleterious	PASSENGER/OTHER	Passenger	passenger	.	.	1	0.88487	73%	Clonal	.	.	.	.	WES		
IDC55T	HSD11B1	p.S161R	Missense_Mutation	1	209880439	C	G	242	26.03%	Deleterious	PASSENGER/OTHER	Passenger	passenger	.	.	0.96	0.83761	75%	subclonal	.	.	.	.	WES		
IDC55T	ITIH4	p.T192S	Missense_Mutation	3	52860612	G	C	141	56.74%	Deleterious	PASSENGER/OTHER	Passenger	passenger	.	loh	0.95	0.88067	80%	Clonal	.	.	.	.	WES		
IDC55T	KIF19	p.R229H	Missense_Mutation	17	72341003	G	A	326	34.97%	Deleterious	PASSENGER/OTHER	Passenger	passenger	.	.	0.93	0.81333	79%	Clonal	.	.	.	.	WES		
IDC55T	KLHL12	p.I537S	Missense_Mutation	1	202861758	A	C	308	26.62%	Deleterious	PASSENGER/OTHER	Driver	likely_pathogenic	.	.	0.98	0.87193	79%	Clonal	.	.	.	.	WES		
IDC55T	MESP2	p.H134P	Missense_Mutation	15	90319989	A	C	231	36.80%	Deleterious	CANCER	Passenger	likely_pathogenic	.	.	0.98	0.88484	80%	Clonal	.	.	.	.	WES		
IDC55T	MROH2B	p.I209M	Missense_Mutation	5	41058294	G	C	136	39.71%	Neutral	PASSENGER/OTHER	Passenger	passenger	.	.	1	0.90805	80%	Clonal	.	.	.	.	WES		
IDC55T	MYOZ1	p.G146S	Missense_Mutation	10	75394308	C	T	295	4.07%	Deleterious	PASSENGER/OTHER	Passenger	passenger	.	.	0.11	0	6%	subclonal	.	.	.	.	WES		
IDC55T	NAPG	p.D243N	Missense_Mutation	18	10549025	G	A	260	3.85%	Deleterious	PASSENGER/OTHER	Passenger	passenger	.	.	0.14	0	7%	subclonal	.	.	.	.	WES		
IDC55T	NPBWR2	p.E104D	Missense_Mutation	20	62737873	C	G	321	42.06%	Deleterious	PASSENGER/OTHER	Passenger	passenger	.	.	1	0.003	95%	Clonal	.	.	.	.	WES		
IDC55T	NT5C1B	p.S150I	Missense_Mutation	2	18766234	C	A	299	37.46%	Neutral	CANCER	Passenger	passenger	.	.	1	0.91149	84%	Clonal	.	.	.	.	WES		
IDC55T	OR51E1	p.F97C	Missense_Mutation	11	4674046	T	G	469	3.62%	Deleterious	PASSENGER/OTHER	Passenger	passenger	.	.	0.1	0	6%	subclonal	.	.	.	.	WES		
IDC55T	OR6T1	p.Q53R	Missense_Mutation	11	123814388	T	C	281	35.23%	Neutral	PASSENGER/OTHER	Passenger	passenger	.	.	0.94	0.82969	79%	Clonal	.	.	.	.	WES		
IDC55T	OTOG	p.C2330R	Missense_Mutation	11	17653653	T	C	297	37.04%	Deleterious	CANCER	Driver	likely_pathogenic	.	.	0.99	0.90126	83%	Clonal	.	.	.	.	WES		
IDC55T	PHLDB1	p.A102D	Missense_Mutation	11	118486876	C	A	314	39.49%	Deleterious	PASSENGER/OTHER	Passenger	passenger	.	.	1	0.94274	87%	Clonal	.	.	.	.	WES		
IDC55T	PKHD1L1	p.A254S	Missense_Mutation	8	110406663	G	T	101	16.83%	Neutral	PASSENGER/OTHER	Passenger	passenger	.	.	0.62	0.11892	39%	Clonal	.	.	.	.	WES		
IDC55T	PPIL2	p.P500S	Missense_Mutation	22	22049718	C	T	142	5.63%	Neutral	PASSENGER/OTHER	Passenger	passenger	.	loh	0.09	0	4%	subclonal	.	.	.	.	WES		
IDC55T	PTCHD3	p.I475L	Missense_Mutation	10	27688104	T	G	73	38.36%	Neutral	CANCER	Passenger	passenger	.	.	1	0.85283	70%	Clonal	.	.	.	.	WES		
IDC55T	RAB44	p.A617T	Missense_Mutation	6	36690078	G	A	463	40.39%		.	Passenger	passenger	.	.	1	0.95912	91%	Clonal	.	.	.	.	WES		
IDC55T	RADIL	p.T214K	Missense_Mutation	7	4876131	G	T	479	38.41%	Neutral	PASSENGER/OTHER	Passenger	passenger	.	.	1	0.00015	95%	Clonal	.	.	.	.	WES		
IDC55T	RALBP1	p.G616R	Missense_Mutation	18	9535813	G	C	204	21.57%	Neutral	PASSENGER/OTHER	Passenger	passenger	.	.	0.79	0.3986	60%	Clonal	.	.	.	.	WES		
IDC55T	RAP2B	p.K94N	Missense_Mutation	3	152880764	G	C	478	4.39%	Deleterious	PASSENGER/OTHER	Passenger	passenger	.	.	0.12	0	7%	subclonal	.	.	.	.	WES		
IDC55T	RBL1	p.P225T	Missense_Mutation	20	35695408	G	T	144	43.06%	Deleterious	CANCER	Passenger	likely_pathogenic	.	.	1	0.92981	85%	Clonal	.	.	.	.	WES		
IDC55T	RBM14	p.Y285H	Missense_Mutation	11	66392200	T	C	551	34.30%	Deleterious	PASSENGER/OTHER	Passenger	passenger	.	.	0.92	0.70507	81%	Clonal	.	.	.	.	WES		
IDC55T	RBP3	p.V1183M	Missense_Mutation	10	48382102	C	T	217	41.94%	Deleterious	PASSENGER/OTHER	Passenger	passenger	.	.	1	0.94184	87%	Clonal	.	.	.	.	WES		
IDC55T	RECQL4	p.P893S	Missense_Mutation	8	145738308	G	A	274	52.55%	.	.	Passenger	passenger	.	loh	1	0.0005	95%	Clonal	TRUE	.	.	YES	WES		
IDC55T	RHOT2	p.E177Q	Missense_Mutation	16	720546	G	C	150	68.00%	Deleterious	PASSENGER/OTHER	Passenger	passenger	.	loh	1	0.91163	95%	Clonal	.	.	.	.	WES		
IDC55T	RMND5B	p.Y129C	Missense_Mutation	5	177569953	A	G	185	4.86%	Deleterious	.	Passenger	passenger	.	loh	0.08	0	4%	subclonal	.	.	.	.	WES		
IDC55T	RYR2	p.R1009W	Missense_Mutation	1	237711849	C	T	86	34.88%	Deleterious	CANCER	Passenger	likely_pathogenic	.	.	1	0.89013	77%	Clonal	.	.	.	.	WES		
IDC55T	RYR2	p.K1348E	Missense_Mutation	1	237754174	A	G	410	26.83%	Deleterious	CANCER	Passenger	likely_pathogenic	.	.	0.99	0.89135	81%	subclonal	.	.	.	.	WES		
IDC55T	S1PR2	p.N10Y	Missense_Mutation	19	10335554	T	A	358	40.50%	Deleterious	.	Passenger	passenger	.	.	1	0.00013	95%	Clonal	.	.	.	.	WES		
IDC55T	SALL3	p.E641K	Missense_Mutation	18	76753912	G	A	481	27.86%	Deleterious	PASSENGER/OTHER	Passenger	passenger	.	.	0.74	0.00039	64%	subclonal	.	.	.	.	WES		
IDC55T	SAMD3	p.K48M	Missense_Mutation	6	130535608	T	A	153	38.56%	Deleterious	PASSENGER/OTHER	Passenger	passenger	.	.	1	0.89997	79%	Clonal	.	.	.	.	WES		
IDC55T	SEMA6C	p.T674S	Missense_Mutation	1	151105829	T	A	284	57.04%	Neutral	PASSENGER/OTHER	Passenger	passenger	.	.	1	0.97452	97%	Clonal	.	.	.	.	WES		
IDC55T	SERPINB2	p.T367N	Missense_Mutation	18	61570391	C	A	245	30.61%	Deleterious	CANCER	Passenger	likely_pathogenic	.	.	0.82	0.29053	67%	subclonal	.	.	.	.	WES		
IDC55T	SIGLEC12	p.D318A	Missense_Mutation	19	52002826	T	G	56	51.79%	Neutral	PASSENGER/OTHER	Passenger	passenger	.	loh	0.87	0.71863	64%	subclonal	.	.	.	.	WES		
IDC55T	SLC25A23	p.D94N	Missense_Mutation	19	6458212	C	T	98	42.86%	Deleterious	PASSENGER/OTHER	Passenger	passenger	.	.	1	0.7282	86%	Clonal	.	.	.	.	WES		
IDC55T	SPAG7	p.P113S	Missense_Mutation	17	4863397	G	A	195	34.87%	Deleterious	PASSENGER/OTHER	Passenger	passenger	.	loh	0.93	0.80708	75%	Clonal	.	.	.	.	WES		
IDC55T	STXBP5L	p.R66Q	Missense_Mutation	3	120673794	G	A	171	37.43%	Deleterious	PASSENGER/OTHER	Passenger	passenger	.	.	1	0.88595	79%	Clonal	.	.	.	.	WES		
IDC55T	SYNE2	p.Q796E	Missense_Mutation	14	64457201	C	G	43	6.98%	Deleterious	PASSENGER/OTHER	Passenger	passenger	.	loh	0.12	0	4%	subclonal	.	.	.	.	WES		
IDC55T	TAF1	p.S30C	Missense_Mutation	23	70586253	C	G	467	8.14%	Neutral	PASSENGER/OTHER	Passenger	passenger	.	.	0.22	0	15%	subclonal	.	TRUE	.	.	WES		
IDC55T	TBC1D19	p.A503E	Missense_Mutation	4	26756496	C	A	78	7.69%	Deleterious	PASSENGER/OTHER	Passenger	passenger	.	loh	0.13	0	6%	subclonal	.	.	.	.	WES		
IDC55T	TBX4	p.I64L	Missense_Mutation	17	59534901	A	C	423	24.82%	Deleterious	CANCER	Passenger	likely_pathogenic	.	.	0.66	0	55%	Clonal	.	.	.	.	WES		
IDC55T	TEX15	p.A600S	Missense_Mutation	8	30704736	C	A	193	6.22%	Neutral	PASSENGER/OTHER	Passenger	passenger	.	loh	0.1	0	5%	subclonal	.	.	.	.	WES		
IDC55T	TP53	p.R282W	Missense_Mutation	17	7577094	G	A	268	73.51%	Deleterious	CANCER	Passenger	likely_pathogenic	TRUE	loh	1	0.98681	97%	Clonal	TRUE	TRUE	TRUE	YES	WES	GCTTCTTGTCCTGCTTGCTT	CTACTGGGACGGAACAGCTT
IDC55T	TRERF1	p.Q505K	Missense_Mutation	6	42232564	G	T	201	41.29%	Neutral	PASSENGER/OTHER	Passenger	passenger	.	.	1	0.93769	86%	Clonal	.	.	.	.	WES		
IDC55T	TRIM42	p.E178K	Missense_Mutation	3	140401494	G	A	598	33.28%	Deleterious	PASSENGER/OTHER	Passenger	passenger	.	.	0.89	0.45108	79%	subclonal	.	.	.	.	WES		
IDC55T	TRIM58	p.E317Q	Missense_Mutation	1	248039279	G	C	740	24.05%	Deleterious	PASSENGER/OTHER	Passenger	passenger	.	.	0.88	0.49599	77%	subclonal	.	.	.	.	WES		
IDC55T	TRMT10A	p.L150R	Missense_Mutation	4	100477349	A	C	133	5.26%	Deleterious	PASSENGER/OTHER	Passenger	passenger	.	.	0.14	0	6%	subclonal	.	.	.	.	WES		
IDC55T	TSKU	p.R108C	Missense_Mutation	11	76506982	C	T	578	39.27%	Deleterious	PASSENGER/OTHER	Passenger	passenger	.	.	1	0.95964	91%	Clonal	.	.	.	.	WES		
IDC55T	TTN	p.T27265A	Missense_Mutation	2	179429066	T	C	167	43.11%	Deleterious	PASSENGER/OTHER	Passenger	passenger	.	.	1	0.93418	86%	Clonal	.	.	.	.	WES		
IDC55T	UPF2	p.R854P	Missense_Mutation	10	11998332	C	G	117	14.53%	Deleterious	PASSENGER/OTHER	Passenger	passenger	.	.	0.68	0.30019	43%	Clonal	.	.	.	.	WES		
IDC55T	WDR66	p.D318E	Missense_Mutation	12	122372218	C	G	103	56.31%	Deleterious	PASSENGER/OTHER	Passenger	passenger	.	loh	0.94	0.86559	77%	Clonal	.	.	.	.	WES		
IDC55T	ZBTB5	p.R132L	Missense_Mutation	9	37442154	C	A	380	28.16%	Deleterious	PASSENGER/OTHER	Passenger	passenger	.	.	1	0.92306	84%	Clonal	.	.	.	.	WES		
IDC55T	ZNF197	p.R49P	Missense_Mutation	3	44670792	G	C	303	13.20%	Neutral	PASSENGER/OTHER	Passenger	passenger	.	loh	0.35	0	26%	.	.	.	.	.	WES		
IDC55T	ZNF280C	p.A589T	Missense_Mutation	23	129349838	C	T	217	4.15%	Neutral	PASSENGER/OTHER	Passenger	passenger	.	.	0.11	0	5%	subclonal	.	.	.	.	WES		
IDC55T	ZNF507	p.Q17H	Missense_Mutation	19	32843787	G	T	289	3.46%	Neutral	PASSENGER/OTHER	Passenger	passenger	.	.	0.13	0	6%	subclonal	.	.	.	.	WES		
IDC55T	SPEN	p.S247*	Nonsense_Mutation	1	16203032	C	A	243	4.12%	Deleterious	.	.	likely_pathogenic	.	loh	0.11	0	6%	subclonal	TRUE	.	TRUE	YES	WES		
IDC55T	OR8B4	p.*310Yext*?	Nonstop_Mutation	11	124293838	T	A	46	47.83%	Neutral	.	.	passenger	.	.	1	0.8839	74%	Clonal	.	.	.	.	WES		
IDC55T	LZTFL1	p.X200_splice	Splice_Site	3	45872404	C	T	77	10.39%	Deleterious	.	.	likely_pathogenic	.	loh	0.17	0	9%	subclonal	.	.	.	.	WES		
IDC55T	NCAPH2	p.X141_splice	Splice_Site	22	50956401	G	T	311	42.44%	Deleterious	.	.	passenger	.	.	1	0.00721	95%	Clonal	.	.	.	.	WES		
IDC55T	VWF	p.X178_splice	Splice_Site	12	6219538	A	C	296	21.28%	Deleterious	.	.	passenger	.	.	1	0.91681	84%	Clonal	.	.	.	.	WES		
IDC55T	EPN3	p.M1?	Translation_Start_Site	17	48613919	T	C	400	38.25%	Deleterious	PASSENGER/OTHER	Passenger	passenger	.	.	1	0.93703	87%	Clonal	.	.	.	.	WES		
IDC55T	UNC45A	p.M1?	Translation_Start_Site	15	91478557	G	A	387	42.12%	Deleterious	PASSENGER/OTHER	Passenger	passenger	.	.	1	0.94929	92%	Clonal	.	.	.	.	WES		
IDC59T	C11orf70	p.F111Ifs*2	Frame_Shift_Del	11	101937275	TTTTTTCATCGG	T	47	53.19%	Deleterious	.	.	likely_pathogenic	.	loh	1	0.96956	83%	subclonal	.	.	.	.	WES		
IDC59T	CNBD1	p.D255Afs*2	Frame_Shift_Del	8	88249329	TATGACTCA	T	239	15.48%	Deleterious	.	.	likely_pathogenic	.	.	1	0.88217	80%	Clonal	.	.	TRUE	.	WES		
IDC59T	IQSEC2	p.L1370Pfs*22	Frame_Shift_Del	23	53263743	GGGGGCAGGACTGTACA	G	62	9.68%	.	.	.	passenger	.	.	0.57	0.36295	26%	subclonal	.	.	.	.	WES		
IDC59T	C14orf93	p.E491_P495del	In_Frame_Del	14	23456556	AGGATTGTAAAGTTCT	A	220	5.91%	Neutral	.	.	passenger	.	.	0.35	0.00002	20%	subclonal	.	.	.	.	WES		
IDC59T	LPIN2	p.A315_D319del	In_Frame_Del	18	2937901	GTCTTCCATGGAAGCA	G	86	11.63%	.	.	.	passenger	.	.	0.68	0.48518	37%	subclonal	.	.	.	.	WES		
IDC59T	SEPT11	p.V5M	Missense_Mutation	4	77871069	G	A	27	11.11%	Deleterious	PASSENGER/OTHER	Passenger	passenger	.	.	0.65	0.5567	21%	subclonal	.	.	.	.	WES		
IDC59T	AOC3	p.S594R	Missense_Mutation	17	41006646	C	G	196	16.84%	Deleterious	PASSENGER/OTHER	Passenger	passenger	.	.	0.98	0.85127	68%	Clonal	.	.	.	.	WES		
IDC59T	BNC2	p.V774A	Missense_Mutation	9	16435871	A	G	166	11.45%	Deleterious	PASSENGER/OTHER	Passenger	passenger	.	loh	0.44	0.0001	28%	subclonal	.	.	.	.	WES		
IDC59T	BUB1B	p.R677H	Missense_Mutation	15	40500858	G	A	98	26.53%	Neutral	PASSENGER/OTHER	Passenger	passenger	.	.	1	0.89814	74%	Clonal	TRUE	.	.	.	WES		
IDC59T	C12orf61	p.R17P	Missense_Mutation	12	62997069	C	G	46	30.43%	Neutral	.	Passenger	passenger	.	.	1	0.94458	69%	subclonal	.	.	.	.	WES		
IDC59T	CCDC122	p.I97M	Missense_Mutation	13	44434072	G	C	133	3.76%	Neutral	PASSENGER/OTHER	Passenger	passenger	.	loh	0.14	0	6%	subclonal	.	.	.	.	WES		
IDC59T	CCNJL	p.L92F	Missense_Mutation	5	159707538	G	A	27	11.11%	Deleterious	PASSENGER/OTHER	Passenger	passenger	.	.	0.65	0.5567	21%	subclonal	.	.	.	.	WES		
IDC59T	CDH26	p.R716H	Missense_Mutation	20	58576447	G	A	128	33.59%	Neutral	PASSENGER/OTHER	Passenger	passenger	.	.	1	0.79871	89%	Clonal	.	.	.	.	WES		
IDC59T	CLCN7	p.V420L	Missense_Mutation	16	1502851	C	A	119	31.09%	Deleterious	CANCER	Passenger	likely_pathogenic	.	loh	1	0.96411	85%	Clonal	.	.	.	.	WES		
IDC59T	CLEC4F	p.T208N	Missense_Mutation	2	71043890	G	T	119	17.65%	Neutral	PASSENGER/OTHER	Passenger	passenger	.	.	0.85	0.72066	55%	subclonal	.	.	.	.	WES		
IDC59T	CNTLN	p.T1109S	Missense_Mutation	9	17462933	C	G	58	25.86%	Neutral	PASSENGER/OTHER	Passenger	passenger	.	loh	0.99	0.81835	57%	subclonal	.	.	.	.	WES		
IDC59T	COL27A1	p.A17G	Missense_Mutation	9	116918280	C	G	9	77.78%	Neutral	CANCER	Passenger	passenger	.	loh	1	0.43341	62%	Clonal	.	.	.	.	WES		
IDC59T	CPAMD8	p.S523Y	Missense_Mutation	19	17091465	G	T	95	8.42%	Deleterious	.	Passenger	passenger	.	loh	0.32	0.00031	16%	subclonal	.	.	.	.	WES		
IDC59T	CXCR3	p.R214H	Missense_Mutation	23	70836822	C	T	88	28.41%	Neutral	PASSENGER/OTHER	Passenger	passenger	.	.	1	0.94028	79%	Clonal	.	.	.	.	WES		
IDC59T	DCAF6	p.G528A	Missense_Mutation	1	167992259	G	C	118	23.73%	Deleterious	PASSENGER/OTHER	Passenger	passenger	.	.	1	0.96182	81%	subclonal	.	.	.	.	WES		
IDC59T	DIP2A	p.V889M	Missense_Mutation	21	47970483	G	A	32	9.38%	Deleterious	PASSENGER/OTHER	Passenger	passenger	.	.	0.55	0.45886	18%	subclonal	.	.	.	.	WES		
IDC59T	FAM196B	p.S348P	Missense_Mutation	5	169309861	A	G	170	7.06%	Neutral	PASSENGER/OTHER	Passenger	passenger	.	.	0.41	0.00232	23%	subclonal	.	.	.	.	WES		
IDC59T	FERMT1	p.K66N	Missense_Mutation	20	6096645	C	A	107	31.78%	Deleterious	PASSENGER/OTHER	Passenger	passenger	.	.	1	0.89903	86%	Clonal	.	.	.	.	WES		
IDC59T	FOXR2	p.L213I	Missense_Mutation	23	55650781	C	A	299	10.37%	Deleterious	CANCER	Passenger	likely_pathogenic	.	.	0.61	0.01932	43%	subclonal	.	.	.	.	WES		
IDC59T	FURIN	p.P764T	Missense_Mutation	15	91425013	C	A	110	37.27%	Deleterious	PASSENGER/OTHER	Passenger	passenger	.	.	1	0.96438	87%	subclonal	.	.	.	.	WES		
IDC59T	GOLGA2	p.V719I	Missense_Mutation	9	131020787	C	T	94	5.32%	Neutral	PASSENGER/OTHER	Passenger	passenger	.	loh	0.31	0.00844	13%	subclonal	.	.	.	YES	WES		
IDC59T	GPR98	p.G4498R	Missense_Mutation	5	90079713	G	A	47	23.40%	Deleterious	PASSENGER/OTHER	Passenger	passenger	.	.	1	0.87241	59%	Clonal	.	.	.	.	WES		
IDC59T	GPRASP1	p.P14L	Missense_Mutation	23	101908882	C	T	105	40.00%	Neutral	PASSENGER/OTHER	Passenger	passenger	.	.	1	0.93646	89%	subclonal	.	.	.	.	WES		
IDC59T	GPRC6A	p.I606L	Missense_Mutation	6	117114270	T	A	110	70.91%	Neutral	CANCER	Passenger	passenger	.	loh	1	0.01593	95%	Clonal	.	.	.	.	WES		
IDC59T	HCAR2	p.I139M	Missense_Mutation	12	123187414	G	C	82	65.85%	Neutral	PASSENGER/OTHER	Passenger	passenger	.	loh	1	0.64781	92%	Clonal	.	.	.	.	WES		
IDC59T	HCN3	p.L108V	Missense_Mutation	1	155252245	C	G	263	46.77%	Deleterious	CANCER	Passenger	likely_pathogenic	.	.	1	0.28806	97%	.	.	.	.	.	WES		
IDC59T	ITGA2	p.I1143F	Missense_Mutation	5	52385854	A	T	114	48.25%	Deleterious	PASSENGER/OTHER	Passenger	passenger	.	.	1	0.92728	91%	Clonal	.	.	.	.	WES		
IDC59T	ITPR3	p.G1217D	Missense_Mutation	6	33646199	G	A	29	65.52%	Deleterious	CANCER	Passenger	likely_pathogenic	.	loh	1	0.9018	81%	Clonal	.	.	.	.	WES		
IDC59T	KCNJ6	p.G119E	Missense_Mutation	21	39087104	C	T	183	38.80%	Deleterious	CANCER	Passenger	likely_pathogenic	.	.	1	0.94745	93%	Clonal	.	.	.	.	WES		
IDC59T	LPIN2	p.N682S	Missense_Mutation	18	2924438	T	C	111	15.32%	Deleterious	PASSENGER/OTHER	Passenger	passenger	.	.	0.89	0.7568	54%	subclonal	.	.	.	.	WES		
IDC59T	MMP19	p.P286A	Missense_Mutation	12	56232429	G	C	272	7.35%	Deleterious	PASSENGER/OTHER	Passenger	passenger	.	.	0.43	0.00007	28%	subclonal	.	.	.	.	WES		
IDC59T	MR1	p.V206L	Missense_Mutation	1	181021382	G	C	192	23.44%	Neutral	PASSENGER/OTHER	Passenger	passenger	.	.	1	0.96624	87%	subclonal	.	.	.	.	WES		
IDC59T	MS4A8	p.S138N	Missense_Mutation	11	60476133	G	A	62	6.45%	Neutral	PASSENGER/OTHER	Passenger	passenger	.	.	0.38	0.10256	15%	subclonal	.	.	.	.	WES		
IDC59T	MYH9	p.E1601K	Missense_Mutation	22	36684429	C	T	84	4.76%	Deleterious	CANCER	Passenger	likely_pathogenic	.	loh	0.18	0.00001	7%	subclonal	TRUE	.	.	.	WES		
IDC59T	NCCRP1	p.Q229P	Missense_Mutation	19	39691123	A	C	130	47.69%	Deleterious	PASSENGER/OTHER	Passenger	passenger	.	.	1	0.57553	93%	Clonal	.	.	.	.	WES		
IDC59T	NRAS	p.R167Q	Missense_Mutation	1	115251226	C	T	123	30.89%	Deleterious	PASSENGER/OTHER	Passenger	passenger	.	loh	1	0.91915	78%	Clonal	TRUE	TRUE	TRUE	YES	WES		
IDC59T	PCDHGA12	p.D912E	Missense_Mutation	5	140890677	T	G	86	16.28%	Neutral	PASSENGER/OTHER	Passenger	passenger	.	.	0.79	0.63741	47%	subclonal	.	.	.	.	WES		
IDC59T	PKHD1L1	p.T1349N	Missense_Mutation	8	110453028	C	A	248	41.53%	Deleterious	CANCER	Passenger	likely_pathogenic	.	.	1	0.48746	96%	Clonal	.	.	.	.	WES		
IDC59T	PRR12	p.G437A	Missense_Mutation	19	50098902	G	C	118	5.93%	Neutral	.	Passenger	passenger	.	.	0.35	0.0048	17%	subclonal	.	.	.	.	WES		
IDC59T	PSME4	p.F1349L	Missense_Mutation	2	54120089	G	C	184	27.17%	Deleterious	PASSENGER/OTHER	Passenger	passenger	.	.	1	0.88303	84%	Clonal	.	.	.	.	WES		
IDC59T	PYHIN1	p.L220V	Missense_Mutation	1	158911845	C	G	240	30.00%	Neutral	PASSENGER/OTHER	Passenger	passenger	.	.	1	0.91543	93%	Clonal	.	.	.	.	WES		
IDC59T	RABGGTA	p.T454A	Missense_Mutation	14	24736089	T	C	51	41.18%	Deleterious	PASSENGER/OTHER	Passenger	passenger	.	.	1	0.92946	80%	Clonal	.	.	.	.	WES		
IDC59T	RBP3	p.Q896E	Missense_Mutation	10	48388192	G	C	87	45.98%	Deleterious	PASSENGER/OTHER	Passenger	passenger	.	.	1	0.74832	89%	Clonal	.	.	.	.	WES		
IDC59T	RGS18	p.R215Q	Missense_Mutation	1	192153620	G	A	400	27.50%	Deleterious	PASSENGER/OTHER	Passenger	passenger	.	.	1	0.97001	95%	.	.	.	.	.	WES		
IDC59T	RNF180	p.A92D	Missense_Mutation	5	63509428	C	A	151	21.19%	Deleterious	PASSENGER/OTHER	Passenger	passenger	.	.	1	0.87344	70%	subclonal	.	.	.	.	WES		
IDC59T	SDC3	p.Q266R	Missense_Mutation	1	31349472	T	C	73	26.03%	Neutral	PASSENGER/OTHER	Passenger	passenger	.	.	1	0.8844	69%	Clonal	.	.	.	.	WES		
IDC59T	SEMA6A	p.G736S	Missense_Mutation	5	115783196	C	T	282	35.46%	Neutral	PASSENGER/OTHER	Passenger	passenger	.	.	1	0.97936	94%	Clonal	.	.	.	.	WES		
IDC59T	SESTD1	p.S568P	Missense_Mutation	2	179979929	A	G	153	29.41%	Deleterious	PASSENGER/OTHER	Passenger	passenger	.	.	1	0.77817	84%	Clonal	.	.	.	.	WES		
IDC59T	SIX3	p.A314S	Missense_Mutation	2	45171840	G	T	99	39.39%	Deleterious	CANCER	Passenger	likely_pathogenic	.	.	1	0.97167	87%	subclonal	.	.	.	.	WES		
IDC59T	SNTG2	p.P165L	Missense_Mutation	2	1161316	C	T	55	7.27%	Deleterious	PASSENGER/OTHER	Passenger	passenger	.	loh	0.28	0.00573	11%	subclonal	.	.	.	.	WES		
IDC59T	SYDE2	p.E690D	Missense_Mutation	1	85648255	C	G	133	54.89%	Deleterious	PASSENGER/OTHER	Passenger	passenger	.	loh	1	0.97756	93%	subclonal	.	.	.	.	WES		
IDC59T	TBX20	p.N211S	Missense_Mutation	7	35284583	T	C	165	22.42%	Deleterious	CANCER	Passenger	likely_pathogenic	.	.	1	0.96734	85%	subclonal	.	.	.	.	WES		
IDC59T	TH	p.R507W	Missense_Mutation	11	2185531	G	A	39	7.69%	Neutral	CANCER	Passenger	passenger	.	loh	0.3	0.03768	10%	subclonal	.	.	.	.	WES		
IDC59T	TRAV18	p.A36V	Missense_Mutation	14	22471684	C	T	222	19.37%	.	.	Passenger	passenger	.	.	1	0.91385	77%	Clonal	.	.	.	.	WES		
IDC59T	TSPYL5	p.L274V	Missense_Mutation	8	98289253	G	C	458	8.52%	Neutral	PASSENGER/OTHER	Passenger	passenger	.	.	0.75	0.37339	55%	subclonal	.	.	.	.	WES		
IDC59T	UBL7	p.G223S	Missense_Mutation	15	74742014	C	T	110	19.09%	Deleterious	PASSENGER/OTHER	Passenger	passenger	.	.	0.92	0.7919	59%	subclonal	.	.	.	.	WES		
IDC59T	WAC	p.S523R	Missense_Mutation	10	28905112	A	C	226	24.78%	Deleterious	PASSENGER/OTHER	Passenger	passenger	.	.	1	0.93451	89%	Clonal	.	.	.	.	WES		
IDC59T	XPNPEP2	p.R598Q	Missense_Mutation	23	128901631	G	A	188	3.72%	Deleterious	PASSENGER/OTHER	Passenger	passenger	.	loh	0.14	0	7%	subclonal	.	.	.	.	WES		
IDC59T	ZNF48	p.P443T	Missense_Mutation	16	30409898	C	A	127	34.65%	Deleterious	PASSENGER/OTHER	Passenger	passenger	.	.	1	0.97227	88%	Clonal	.	.	.	.	WES		
IDC59T	GPRASP1	p.E15*	Nonsense_Mutation	23	101908884	G	T	101	40.59%	Deleterious	.	.	passenger	.	.	1	0.92732	89%	subclonal	.	.	.	.	WES		
IDC59T	KIAA1522	p.Q138*	Nonsense_Mutation	1	33233668	C	T	145	21.38%	Deleterious	.	.	passenger	.	.	1	0.87512	70%	Clonal	.	.	.	.	WES		
IDC59T	KIF26B	p.S1668*	Nonsense_Mutation	1	245851288	C	A	103	11.65%	Deleterious	.	.	passenger	.	.	0.91	0.75077	49%	subclonal	.	.	.	.	WES		
IDC59T	NLGN1	p.K47*	Nonsense_Mutation	3	173322527	A	T	157	56.69%	Deleterious	.	.	passenger	.	.	1	0.46975	95%	Clonal	.	.	.	.	WES		
IDC59T	C10orf76	p.*690Cext*9	Nonstop_Mutation	10	103607285	T	A	88	55.68%	Deleterious	.	.	likely_pathogenic	.	loh	1	0.97218	91%	subclonal	.	.	.	.	WES		
IDC59T	CFH	p.X82_splice	Splice_Site	1	196642294	GTAAGTACT	G	165	37.58%	Deleterious	.	.	passenger	.	.	1	0.58788	93%	.	.	.	.	.	WES		
IDC59T	CHD4	p.X983_splice	Splice_Site	12	6701225	T	C	210	40.95%	Deleterious	.	.	likely_pathogenic	.	.	1	0.85051	94%	Clonal	TRUE	.	TRUE	.	WES		
IDC59T	OPA1	p.X543_splice	Splice_Site	3	193363415	G	C	167	9.58%	Deleterious	.	.	likely_pathogenic	.	.	0.66	0.31685	40%	subclonal	.	.	.	.	WES		
IDC60T	DOCK3	p.E1178Ffs*4	Frame_Shift_Del	3	51370603	TTGAACAAGAAACATGGCGCGAGACCGGC	T	48	14.58%	.	.	.	likely_pathogenic	.	loh	0.53	0.22253	26%	subclonal	.	.	.	.	WES		
IDC60T	MYO1H	p.G212Rfs*17	Frame_Shift_Del	12	109839008	TGGCGA	T	62	11.29%	.	.	.	passenger	.	.	0.52	0.18616	25%	subclonal	.	.	.	.	WES		
IDC60T	MYO1H	p.A210Vfs*93	Frame_Shift_Del	12	109839001	TGGCA	T	68	13.24%	.	.	.	passenger	.	.	0.61	0.31325	32%	subclonal	.	.	.	.	WES		
IDC60T	MAR3	p.G184A	Missense_Mutation	5	126213929	C	G	27	37.04%	Deleterious	PASSENGER/OTHER	Passenger	passenger	.	loh	1	0.86671	59%	Clonal	.	.	.	.	WES		
IDC60T	ABCC12	p.H926N	Missense_Mutation	16	48138177	G	T	56	17.86%	Deleterious	CANCER	Passenger	likely_pathogenic	.	loh	0.82	0.65119	44%	Clonal	.	.	.	.	WES		
IDC60T	ADAM20	p.N336K	Missense_Mutation	14	70990617	G	T	17	29.41%	Neutral	PASSENGER/OTHER	Passenger	passenger	.	.	1	0.79446	42%	Clonal	.	.	.	.	WES		
IDC60T	AHNAK	p.P4397S	Missense_Mutation	11	62288700	G	A	122	21.31%	Deleterious	PASSENGER/OTHER	Passenger	passenger	.	.	0.98	0.82127	65%	Clonal	.	.	.	.	WES		
IDC60T	AKAP13	p.E710K	Missense_Mutation	15	86123427	G	A	39	28.21%	Neutral	PASSENGER/OTHER	Passenger	passenger	.	.	1	0.88313	62%	subclonal	.	.	.	.	WES		
IDC60T	ARHGAP44	p.V190G	Missense_Mutation	17	12832350	T	G	22	36.36%	Deleterious	PASSENGER/OTHER	Passenger	passenger	.	loh	1	0.84945	53%	Clonal	.	.	.	.	WES		
IDC60T	ARHGAP9	p.E130Q	Missense_Mutation	12	57872469	C	G	33	30.30%	Neutral	PASSENGER/OTHER	Passenger	passenger	.	.	1	0.85046	59%	Clonal	.	.	.	.	WES		
IDC60T	ARHGEF39	p.E189K	Missense_Mutation	9	35663051	C	T	47	29.79%	Deleterious	PASSENGER/OTHER	Passenger	passenger	.	.	1	0.86474	65%	Clonal	.	.	.	.	WES		
IDC60T	B3GAT1	p.D42E	Missense_Mutation	11	134254069	G	T	9	22.22%	Neutral	PASSENGER/OTHER	Passenger	passenger	.	loh	0.8	0.65345	19%	subclonal	.	.	.	.	WES		
IDC60T	BAI3	p.R1302T	Missense_Mutation	6	70071070	G	C	33	39.39%	Deleterious	PASSENGER/OTHER	Passenger	passenger	.	.	1	0.91409	71%	Clonal	.	.	.	.	WES		
IDC60T	BCL11B	p.P112L	Missense_Mutation	14	99723900	G	A	66	19.70%	Deleterious	PASSENGER/OTHER	Passenger	passenger	.	.	0.91	0.73116	51%	Clonal	TRUE	.	.	.	WES		
IDC60T	BORA	p.L160V	Missense_Mutation	13	73318666	C	G	59	32.20%	Deleterious	PASSENGER/OTHER	Passenger	passenger	.	loh	1	0.89383	67%	Clonal	.	.	.	.	WES		
IDC60T	BRIP1	p.E296D	Missense_Mutation	17	59885858	C	G	35	20.00%	Deleterious	PASSENGER/OTHER	Passenger	passenger	.	.	0.92	0.70413	41%	Clonal	TRUE	.	.	YES	WES		
IDC60T	C19orf60	p.S115W	Missense_Mutation	19	18700384	C	G	12	25.00%	Deleterious	.	Passenger	passenger	.	.	1	0.71678	29%	Clonal	.	.	.	.	WES		
IDC60T	C1orf222	p.T354K	Missense_Mutation	1	1861575	G	T	26	11.54%	Deleterious	PASSENGER/OTHER	Passenger	passenger	.	loh	0.42	0.24836	14%	subclonal	.	.	.	.	WES		
IDC60T	CACNA1E	p.M1497I	Missense_Mutation	1	181727244	G	A	45	26.67%	Deleterious	CANCER	Passenger	likely_pathogenic	.	.	1	0.87407	63%	subclonal	.	.	.	.	WES		
IDC60T	CCDC136	p.Q731E	Missense_Mutation	7	128452016	C	G	30	20.00%	Neutral	PASSENGER/OTHER	Passenger	passenger	.	.	0.92	0.6948	38%	Clonal	.	.	.	.	WES		
IDC60T	CCDC154	p.D218Y	Missense_Mutation	16	1492430	C	A	87	26.44%	Neutral	.	Passenger	passenger	.	loh	1	0.88041	71%	Clonal	.	.	.	.	WES		
IDC60T	CCDC159	p.E172Q	Missense_Mutation	19	11462756	G	C	47	23.40%	Neutral	PASSENGER/OTHER	Passenger	passenger	.	.	1	0.79744	54%	Clonal	.	.	.	.	WES		
IDC60T	CCR6	p.F323L	Missense_Mutation	6	167550687	C	G	41	26.83%	Neutral	PASSENGER/OTHER	Passenger	passenger	.	loh	0.97	0.80635	51%	Clonal	.	.	.	.	WES		
IDC60T	CDC27	p.S231P	Missense_Mutation	17	45234430	A	G	26	15.38%	Deleterious	PASSENGER/OTHER	Driver	likely_pathogenic	.	.	0.71	0.55599	26%	subclonal	.	.	TRUE	.	WES		
IDC60T	CELSR1	p.S2877N	Missense_Mutation	22	46760558	C	T	39	20.51%	Neutral	PASSENGER/OTHER	Passenger	passenger	.	loh	0.74	0.6237	37%	subclonal	.	.	.	.	WES		
IDC60T	CENPE	p.M2136I	Missense_Mutation	4	104057412	C	G	65	47.69%	Neutral	PASSENGER/OTHER	Passenger	passenger	.	loh	1	0.9385	86%	Clonal	.	.	.	.	WES		
IDC60T	CHAT	p.L243F	Missense_Mutation	10	50830171	C	T	43	6.98%	Deleterious	CANCER	Passenger	likely_pathogenic	.	.	0.32	0.06527	11%	subclonal	.	.	.	.	WES		
IDC60T	CHST1	p.V229I	Missense_Mutation	11	45671789	C	T	37	16.22%	Deleterious	PASSENGER/OTHER	Passenger	passenger	.	.	0.75	0.58095	33%	subclonal	.	.	.	.	WES		
IDC60T	CPT1B	p.R645S	Missense_Mutation	22	51009415	G	T	50	6.00%	Deleterious	CANCER	Passenger	likely_pathogenic	.	loh	0.22	0.00187	7%	subclonal	.	.	.	.	WES		
IDC60T	CRTC3	p.P417L	Missense_Mutation	15	91172748	C	T	21	19.05%	Deleterious	PASSENGER/OTHER	Passenger	passenger	.	.	1	0.71527	33%	Clonal	TRUE	.	.	.	WES		
IDC60T	CXorf22	p.G862D	Missense_Mutation	23	35993902	G	A	52	23.08%	Neutral	PASSENGER/OTHER	Passenger	passenger	.	.	1	0.80002	55%	Clonal	.	.	.	.	WES		
IDC60T	CYP3A5	p.P344L	Missense_Mutation	7	99250398	G	A	48	20.83%	Neutral	PASSENGER/OTHER	Passenger	passenger	.	.	0.96	0.74591	49%	Clonal	.	.	.	.	WES		
IDC60T	DNMT3L	p.R161H	Missense_Mutation	21	45678440	C	T	75	24.00%	Deleterious	PASSENGER/OTHER	Passenger	passenger	.	.	1	0.84418	64%	Clonal	.	.	.	.	WES		
IDC60T	DOCK3	p.E1286Q	Missense_Mutation	3	51378757	G	C	32	31.25%	Deleterious	PASSENGER/OTHER	Passenger	passenger	.	loh	1	0.84501	54%	Clonal	.	.	.	.	WES		
IDC60T	DONSON	p.Q169E	Missense_Mutation	21	34958385	G	C	40	25.00%	Deleterious	.	Passenger	passenger	.	.	1	0.80793	54%	Clonal	.	.	.	.	WES		
IDC60T	DOPEY1	p.Q455H	Missense_Mutation	6	83834448	G	C	50	14.00%	Deleterious	PASSENGER/OTHER	Passenger	passenger	.	.	0.78	0.61433	36%	subclonal	.	.	.	.	WES		
IDC60T	EIF3A	p.R803K	Missense_Mutation	10	120810098	C	T	121	4.96%	Deleterious	PASSENGER/OTHER	Passenger	passenger	.	loh	0.18	0	8%	subclonal	.	.	.	.	WES		
IDC60T	ELP5	p.G101R	Missense_Mutation	17	7157966	G	A	34	8.82%	Neutral	PASSENGER/OTHER	Passenger	passenger	.	loh	0.32	0.06646	11%	subclonal	.	.	.	.	WES		
IDC60T	EPG5	p.V2495A	Missense_Mutation	18	43435611	A	G	28	28.57%	Deleterious	PASSENGER/OTHER	Passenger	passenger	.	.	1	0.82251	52%	Clonal	.	.	.	.	WES		
IDC60T	F5	p.S1495F	Missense_Mutation	1	169509844	G	A	49	8.16%	Neutral	CANCER	Passenger	passenger	.	.	0.46	0.22767	18%	subclonal	.	.	.	.	WES		
IDC60T	F5	p.D1414V	Missense_Mutation	1	169510087	T	A	110	14.55%	Neutral	PASSENGER/OTHER	Passenger	passenger	.	.	0.82	0.63984	50%	Clonal	.	.	.	.	WES		
IDC60T	FAM179B	p.D1162H	Missense_Mutation	14	45496657	G	C	28	17.86%	Deleterious	PASSENGER/OTHER	Passenger	passenger	.	.	0.82	0.63557	33%	Clonal	.	.	.	.	WES		
IDC60T	FAM71B	p.E508V	Missense_Mutation	5	156589753	T	A	135	33.33%	Neutral	PASSENGER/OTHER	Passenger	passenger	.	loh	1	0.93565	81%	Clonal	.	.	.	.	WES		
IDC60T	FATE1	p.G26V	Missense_Mutation	23	150884668	G	T	22	13.64%	Neutral	PASSENGER/OTHER	Passenger	passenger	.	.	0.63	0.50067	21%	subclonal	.	.	.	.	WES		
IDC60T	FRMPD4	p.A973T	Missense_Mutation	23	12735862	G	A	29	31.03%	Neutral	PASSENGER/OTHER	Passenger	passenger	.	.	1	0.84964	57%	Clonal	.	.	.	.	WES		
IDC60T	GABRA3	p.V332G	Missense_Mutation	23	151358350	A	C	40	12.50%	Deleterious	CANCER	Passenger	likely_pathogenic	.	.	0.58	0.37779	25%	subclonal	.	.	.	.	WES		
IDC60T	GJA9	p.P493T	Missense_Mutation	1	39340294	G	T	48	6.25%	Neutral	CANCER	Passenger	passenger	.	.	0.35	0.11415	12%	subclonal	.	.	.	.	WES		
IDC60T	GPNMB	p.A22T	Missense_Mutation	7	23286540	G	A	67	23.88%	Neutral	PASSENGER/OTHER	Passenger	passenger	.	.	1	0.86616	67%	Clonal	.	.	.	.	WES		
IDC60T	HERC6	p.G211R	Missense_Mutation	4	89311998	G	A	94	27.66%	Deleterious	CANCER	Passenger	likely_pathogenic	.	loh	1	0.88907	74%	Clonal	.	.	.	.	WES		
IDC60T	HIVEP1	p.Q2166E	Missense_Mutation	6	12161680	C	G	28	28.57%	Deleterious	PASSENGER/OTHER	Passenger	passenger	.	.	1	0.86575	56%	subclonal	.	.	.	.	WES		
IDC60T	HSD3B2	p.P313L	Missense_Mutation	1	119965062	C	T	57	19.30%	Deleterious	PASSENGER/OTHER	Passenger	passenger	.	.	0.89	0.70988	48%	Clonal	.	.	.	.	WES		
IDC60T	IFITM2	p.I4F	Missense_Mutation	11	308202	A	T	41	21.95%	Neutral	PASSENGER/OTHER	Passenger	passenger	.	loh	0.79	0.67926	41%	subclonal	.	.	.	.	WES		
IDC60T	ING5	p.E48Q	Missense_Mutation	2	242648663	G	C	57	17.54%	Deleterious	.	Passenger	passenger	.	.	0.81	0.6366	43%	subclonal	.	.	.	.	WES		
IDC60T	INVS	p.L867M	Missense_Mutation	9	103055138	C	A	76	23.68%	Neutral	PASSENGER/OTHER	Passenger	passenger	.	.	1	0.84035	63%	Clonal	.	.	.	.	WES		
IDC60T	ITPR3	p.E1310K	Missense_Mutation	6	33646572	G	A	22	40.91%	Deleterious	CANCER	Passenger	likely_pathogenic	.	.	1	0.90689	61%	Clonal	.	.	.	.	WES		
IDC60T	KIF7	p.E539K	Missense_Mutation	15	90190234	C	T	79	18.99%	Deleterious	PASSENGER/OTHER	Passenger	passenger	.	.	1	0.81461	59%	Clonal	.	.	.	.	WES		
IDC60T	LAMA5	p.L169V	Missense_Mutation	20	60928253	G	C	86	27.91%	Deleterious	PASSENGER/OTHER	Passenger	passenger	.	.	1	0.88523	73%	Clonal	.	.	.	.	WES		
IDC60T	LGR5	p.Q860R	Missense_Mutation	12	71978369	A	G	37	16.22%	Neutral	PASSENGER/OTHER	Passenger	passenger	.	.	0.75	0.58095	33%	subclonal	.	.	.	.	WES		
IDC60T	LRRK1	p.S1326C	Missense_Mutation	15	101593547	A	T	50	26.00%	Deleterious	CANCER	Passenger	likely_pathogenic	.	.	1	0.90305	70%	Clonal	.	.	.	.	WES		
IDC60T	MAP3K15	p.R1184T	Missense_Mutation	23	19387187	C	G	59	13.56%	Neutral	PASSENGER/OTHER	Passenger	passenger	.	.	0.62	0.37384	32%	subclonal	.	.	.	.	WES		
IDC60T	MMD	p.A47D	Missense_Mutation	17	53488747	G	T	45	6.67%	Deleterious	PASSENGER/OTHER	Passenger	passenger	.	.	0.31	0.04882	11%	subclonal	.	.	.	.	WES		
IDC60T	MYO3A	p.P1551S	Missense_Mutation	10	26491957	C	T	44	29.55%	Deleterious	PASSENGER/OTHER	Passenger	passenger	.	.	1	0.8598	64%	Clonal	.	.	.	.	WES		
IDC60T	NLRP12	p.C752Y	Missense_Mutation	19	54308693	C	T	29	24.14%	Deleterious	PASSENGER/OTHER	Passenger	passenger	.	.	1	0.76998	46%	Clonal	.	.	.	.	WES		
IDC60T	NRIP1	p.S757C	Missense_Mutation	21	16338244	G	C	58	20.69%	Deleterious	PASSENGER/OTHER	Passenger	passenger	.	.	0.95	0.75492	52%	Clonal	.	.	.	.	WES		
IDC60T	PABPC4	p.V37E	Missense_Mutation	1	40041514	A	T	118	33.05%	Deleterious	PASSENGER/OTHER	Passenger	passenger	.	.	1	0.9497	86%	Clonal	.	.	.	.	WES		
IDC60T	PDHA2	p.S109L	Missense_Mutation	4	96761627	C	T	73	23.29%	Neutral	CANCER	Passenger	passenger	.	loh	1	0.83063	62%	Clonal	.	.	.	.	WES		
IDC60T	PEX13	p.S125T	Missense_Mutation	2	61258835	G	C	65	15.38%	Deleterious	CANCER	Passenger	likely_pathogenic	.	.	0.71	0.4981	38%	subclonal	.	.	.	.	WES		
IDC60T	PHF3	p.H1324Y	Missense_Mutation	6	64421231	C	T	43	13.95%	Deleterious	PASSENGER/OTHER	Passenger	passenger	.	.	0.64	0.4553	29%	subclonal	.	.	.	.	WES		
IDC60T	PPM1H	p.R38S	Missense_Mutation	12	63328405	G	T	7	28.57%	Deleterious	PASSENGER/OTHER	Passenger	passenger	.	.	1	0.71361	23%	Clonal	.	.	.	.	WES		
IDC60T	PRDM10	p.K353N	Missense_Mutation	11	129805094	C	G	13	15.38%	Deleterious	PASSENGER/OTHER	Passenger	passenger	.	loh	0.55	0.52569	15%	subclonal	.	.	.	.	WES		
IDC60T	PSMC3	p.E143Q	Missense_Mutation	11	47446006	C	G	62	29.03%	Deleterious	CANCER	Passenger	likely_pathogenic	.	.	1	0.87307	69%	Clonal	.	.	.	.	WES		
IDC60T	RAB44	p.S672Y	Missense_Mutation	6	36690244	C	A	30	16.67%	.	.	Passenger	passenger	.	.	0.77	0.59937	31%	subclonal	.	.	.	.	WES		
IDC60T	RASGRF1	p.S1152F	Missense_Mutation	15	79277356	G	A	92	14.13%	Deleterious	PASSENGER/OTHER	Driver	likely_pathogenic	.	.	0.79	0.61353	46%	subclonal	.	.	.	.	WES		
IDC60T	RIOK3	p.W420R	Missense_Mutation	18	21057146	T	C	48	29.17%	Deleterious	PASSENGER/OTHER	Driver	likely_pathogenic	.	.	1	0.8616	65%	Clonal	.	.	.	.	WES		
IDC60T	SAMD14	p.S64W	Missense_Mutation	17	48195544	G	C	31	16.13%	Deleterious	.	Passenger	passenger	.	.	0.74	0.58007	30%	subclonal	.	.	.	.	WES		
IDC60T	SGCA	p.R221H	Missense_Mutation	17	48246530	G	A	40	22.50%	Deleterious	CANCER	Passenger	likely_pathogenic	.	.	1	0.76842	49%	Clonal	.	.	.	.	WES		
IDC60T	SH3BP5	p.E391Q	Missense_Mutation	3	15297790	C	G	132	20.45%	Deleterious	.	Passenger	passenger	.	.	0.94	0.795	64%	Clonal	.	.	.	.	WES		
IDC60T	SH3D19	p.I583V	Missense_Mutation	4	152054357	T	C	18	16.67%	Deleterious	PASSENGER/OTHER	Passenger	passenger	.	loh	0.6	0.53587	20%	subclonal	.	.	.	.	WES		
IDC60T	SORCS2	p.M962I	Missense_Mutation	4	7730093	G	A	23	21.74%	Neutral	PASSENGER/OTHER	Passenger	passenger	.	loh	0.78	0.672	32%	subclonal	.	.	.	.	WES		
IDC60T	STOX1	p.S654C	Missense_Mutation	10	70645513	C	G	15	26.67%	Neutral	PASSENGER/OTHER	Passenger	passenger	.	.	1	0.75353	36%	Clonal	.	.	.	.	WES		
IDC60T	TEX14	p.S1296F	Missense_Mutation	17	56647814	G	A	40	22.50%	Neutral	CANCER	Passenger	passenger	.	.	1	0.76842	49%	Clonal	.	.	.	.	WES		

IDC60T	TIAM1	p.S20G	Missense_Mutation	21	32639231	T	C	36	22.22%	Neutral	PASSENGER/OTHER	Passenger	passenger	.	.	1	0.75492	46%	Clonal	.	.	.	.	WES		
IDC60T	TMEM216	p.R115C	Missense_Mutation	11	61165359	C	T	75	24.00%	Deleterious	CANCER	Passenger	likely_pathogenic	.	.	1	0.84418	64%	Clonal	.	.	.	.	WES		
IDC60T	TPST1	p.D288H	Missense_Mutation	7	65751514	G	C	38	18.42%	Deleterious	PASSENGER/OTHER	Passenger	passenger	.	.	0.85	0.66315	39%	Clonal	.	.	.	.	WES		
IDC60T	UBR4	p.I4314F	Missense_Mutation	1	19428097	T	A	26	26.92%	Deleterious	PASSENGER/OTHER	Passenger	passenger	.	loh	0.97	0.77878	43%	Clonal	.	.	.	.	WES		
IDC60T	UBXN11	p.H172Q	Missense_Mutation	1	26620739	A	T	34	29.41%	Neutral	PASSENGER/OTHER	Passenger	passenger	.	loh	1	0.83069	53%	Clonal	.	.	.	.	WES		
IDC60T	VPRBP	p.H136Y	Missense_Mutation	3	51464977	G	A	19	31.58%	Deleterious	PASSENGER/OTHER	Passenger	passenger	.	loh	1	0.80709	44%	Clonal	.	.	.	.	WES		
IDC60T	YEATS2	p.V238I	Missense_Mutation	3	183446539	G	A	32	21.88%	Deleterious	PASSENGER/OTHER	Passenger	passenger	.	.	1	0.7393	43%	Clonal	.	.	.	.	WES		
IDC60T	ZFAND4	p.S311C	Missense_Mutation	10	46122339	G	C	21	14.29%	Deleterious	PASSENGER/OTHER	Passenger	passenger	.	.	0.66	0.52511	21%	subclonal	.	.	.	.	WES		
IDC60T	ZNF318	p.E265Q	Missense_Mutation	6	43325259	C	G	88	19.32%	Deleterious	PASSENGER/OTHER	Passenger	passenger	.	.	0.89	0.72817	54%	Clonal	.	.	.	.	WES		
IDC60T	ARID1A	p.Q944*	Nonsense_Mutation	1	27092809	C	T	39	33.33%	Deleterious	.	.	likely_pathogenic	.	loh	1	0.87482	61%	Clonal	TRUE	TRUE	TRUE	YES	WES		
IDC60T	CALCRL	p.Q93*	Nonsense_Mutation	2	188245422	G	A	29	13.79%	Deleterious	.	.	passenger	.	.	0.64	0.48783	24%	subclonal	.	.	.	.	WES		
IDC60T	FERD3L	p.E77*	Nonsense_Mutation	7	19184757	C	A	34	17.65%	Deleterious	.	.	passenger	.	.	0.99	0.71923	40%	Clonal	.	.	.	.	WES		
IDC60T	KIAA0754	p.S766*	Nonsense_Mutation	1	39878642	C	G	37	8.11%	Deleterious	.	.	passenger	.	.	0.45	0.29242	16%	subclonal	.	.	.	.	WES		
IDC60T	MUC16	p.E1826*	Nonsense_Mutation	19	9086339	C	A	34	14.71%	.	.	.	passenger	.	.	0.68	0.51753	28%	subclonal	.	.	.	.	WES		
IDC60T	NRIP1	p.S742*	Nonsense_Mutation	21	16338289	G	C	45	22.22%	Deleterious	.	.	passenger	.	.	1	0.77235	51%	Clonal	.	.	.	.	WES		
IDC60T	PPFIBP1	p.E104*	Nonsense_Mutation	12	27799034	G	T	42	19.05%	Deleterious	.	.	likely_pathogenic	.	.	0.88	0.68733	42%	Clonal	TRUE	.	.	.	WES		
IDC60T	SEL1L2	p.E43*	Nonsense_Mutation	20	13912405	C	A	32	21.88%	Deleterious	.	.	passenger	.	.	1	0.7393	43%	Clonal	.	.	.	.	WES		
IDC60T	ZFX	p.S42*	Nonsense_Mutation	23	24197366	C	G	75	25.33%	Deleterious	.	.	passenger	.	.	1	0.86097	66%	Clonal	.	.	.	.	WES		
IDC60T	KRT34	p.*437Sext*14	Nonstop_Mutation	17	39534312	C	G	30	23.33%	Neutral	.	.	passenger	.	.	1	0.76014	45%	Clonal	.	.	.	.	WES		
IDC60T	C16orf59	p.X66_splice	Splice_Site	16	2510695	G	C	58	17.24%	Deleterious	.	.	likely_pathogenic	.	loh	0.79	0.62137	42%	subclonal	.	.	.	.	WES		
IDC60T	COL22A1	p.X1186_splice	Splice_Site	8	139647307	C	A	48	22.92%	Deleterious	.	.	passenger	.	.	1	0.83672	58%	Clonal	.	.	.	.	WES		
IDC60T	SYNRG	p.X80_splice	Splice_Site	17	35956266	GTA	G	44	13.64%	.	.	.	passenger	.	.	0.63	0.4326	29%	subclonal	.	.	.	.	WES		
IDC61T	DNAH3	p.H2346Tfs*8	Frame_Shift_Del	16	20997027	TG	T	42	28.57%	Deleterious	.	.	likely_pathogenic	.	loh	1	0.86955	64%	Clonal	.	.	.	.	WES		
IDC61T	HNRNPD	p.K242Efs*8	Frame_Shift_Del	4	83278493	CTT	C	171	47.95%	Deleterious	.	.	likely_pathogenic	.	loh	1	0.11403	92%	subclonal	.	.	.	.	WES		
IDC61T	PRDM14	p.P171Tfs*63	Frame_Shift_Del	8	70981581	CAAGGTAAT	C	114	12.28%	Deleterious	.	.	passenger	.	.	0.65	0.2552	39%	Clonal	.	.	.	.	WES		
IDC61T	RGS22	p.D206Ifs*5	Frame_Shift_Del	8	101078503	CT	C	205	16.59%	Deleterious	.	.	passenger	.	.	1	0.83939	70%	Clonal	.	.	.	.	WES		
IDC61T	ARFGEF1	p.M1255I	Missense_Mutation	8	68139523	C	T	94	14.89%	Deleterious	PASSENGER/OTHER	Passenger	passenger	.	.	0.49	0.0057	29%	subclonal	.	.	.	.	WES		
IDC61T	ASL	p.A218V	Missense_Mutation	7	65552371	C	T	27	33.33%	Neutral	CANCER	Passenger	passenger	.	.	1	0.89721	60%	Clonal	.	.	.	.	WES		
IDC61T	ATP2B2	p.S860T	Missense_Mutation	3	10387192	C	G	116	32.76%	Deleterious	CANCER	Passenger	likely_pathogenic	.	.	1	0.87429	75%	Clonal	.	.	.	.	WES		
IDC61T	ATP2B2	p.S860R	Missense_Mutation	3	10387191	G	T	118	33.05%	Deleterious	CANCER	Passenger	likely_pathogenic	.	.	1	0.87932	76%	Clonal	.	.	.	.	WES		
IDC61T	BBS10	p.R249S	Missense_Mutation	12	76741020	G	T	62	33.87%	Neutral	PASSENGER/OTHER	Passenger	passenger	.	.	1	0.8415	68%	Clonal	.	.	.	.	WES		
IDC61T	BPIFC	p.V178F	Missense_Mutation	22	32841684	C	A	90	10.00%	Deleterious	PASSENGER/OTHER	Passenger	passenger	.	.	0.63	0.3237	33%	subclonal	.	.	.	.	WES		
IDC61T	C10orf12	p.D415Y	Missense_Mutation	10	98742390	G	T	24	20.83%	Neutral	PASSENGER/OTHER	Passenger	passenger	.	.	0.68	0.46708	29%	subclonal	.	.	.	.	WES		
IDC61T	C10orf12	p.K611E	Missense_Mutation	10	98742978	A	G	39	12.82%	Neutral	PASSENGER/OTHER	Passenger	passenger	.	.	0.42	0.06244	18%	subclonal	.	.	.	.	WES		
IDC61T	C1orf173	p.K495I	Missense_Mutation	1	75072290	T	A	282	2.84%	Deleterious	PASSENGER/OTHER	Passenger	passenger	.	.	0.15	0	7%	subclonal	.	.	.	.	WES		
IDC61T	CASP8	p.Y285F	Missense_Mutation	2	202141566	A	T	91	57.14%	Deleterious	CANCER	Passenger	likely_pathogenic	.	loh	1	0.96738	91%	Clonal	TRUE	.	TRUE	YES	WES		
IDC61T	CCDC147	p.D622E	Missense_Mutation	10	106160488	T	A	74	10.81%	Deleterious	PASSENGER/OTHER	Passenger	passenger	.	.	0.35	0.00079	18%	subclonal	.	.	.	.	WES		
IDC61T	CDC42BPB	p.K1675N	Missense_Mutation	14	103400160	T	G	66	43.94%	Deleterious	PASSENGER/OTHER	Passenger	passenger	.	loh	1	0.87577	70%	Clonal	.	.	.	.	WES		
IDC61T	CDCP2	p.P271T	Missense_Mutation	1	54605732	G	T	10	30.00%	Deleterious	PASSENGER/OTHER	Passenger	passenger	.	loh	0.98	0.63892	28%	Clonal	.	.	.	.	WES		
IDC61T	CECR2	p.H1281N	Missense_Mutation	22	18028881	C	A	45	42.22%	Deleterious	PASSENGER/OTHER	Passenger	passenger	.	.	1	0.94529	76%	Clonal	.	.	.	.	WES		
IDC61T	DIAPH1	p.P740A	Missense_Mutation	5	140953199	G	C	40	20.00%	Deleterious	CANCER	Passenger	likely_pathogenic	.	loh	0.65	0.36804	34%	subclonal	.	.	TRUE	.	WES		
IDC61T	DNASE1L1	p.F252L	Missense_Mutation	23	153631301	G	C	15	86.67%	Neutral	PASSENGER/OTHER	Passenger	passenger	.	loh	1	0.68449	76%	Clonal	.	.	.	.	WES		
IDC61T	EGFLAM	p.R642W	Missense_Mutation	5	38427224	C	T	120	7.50%	Deleterious	PASSENGER/OTHER	Passenger	passenger	.	loh	0.25	0	13%	subclonal	.	.	.	.	WES		
IDC61T	FAM186A	p.M794I	Missense_Mutation	12	50748233	C	A	101	25.74%	Neutral	PASSENGER/OTHER	Passenger	passenger	.	.	0.84	0.61729	58%	subclonal	.	.	.	.	WES		
IDC61T	FAM83F	p.S316F	Missense_Mutation	22	40417461	C	T	38	39.47%	Deleterious	PASSENGER/OTHER	Passenger	passenger	.	.	1	0.89485	74%	Clonal	.	.	.	.	WES		
IDC61T	FHOD3	p.F1259L	Missense_Mutation	18	34335149	T	C	197	5.08%	Deleterious	PASSENGER/OTHER	Passenger	passenger	.	.	0.22	0	11%	subclonal	.	.	.	.	WES		
IDC61T	FMNL1	p.G284R	Missense_Mutation	17	43314962	G	A	56	66.07%	Deleterious	CANCER	Passenger	likely_pathogenic	.	loh	1	0.96074	89%	Clonal	.	.	.	.	WES		
IDC61T	FSTL5	p.K783R	Missense_Mutation	4	162307095	T	C	125	22.40%	Deleterious	PASSENGER/OTHER	Passenger	passenger	.	loh	0.73	0.26491	52%	subclonal	.	.	.	.	WES		
IDC61T	GP6	p.R352T	Missense_Mutation	19	55526258	C	G	44	27.27%	Neutral	PASSENGER/OTHER	Passenger	passenger	.	.	1	0.79925	58%	subclonal	.	.	.	.	WES		
IDC61T	GPR126	p.T752A	Missense_Mutation	6	142726951	A	G	80	63.75%	Deleterious	PASSENGER/OTHER	Passenger	passenger	.	loh	1	0.97164	91%	Clonal	.	.	.	.	WES		
IDC61T	GPR158	p.Y157H	Missense_Mutation	10	25464818	T	C	28	14.29%	Deleterious	PASSENGER/OTHER	Passenger	passenger	.	.	0.61	0.41076	23%	subclonal	.	.	.	.	WES		
IDC61T	GRID1	p.F73I	Missense_Mutation	10	88123716	A	T	60	31.67%	Deleterious	CANCER	Driver	likely_pathogenic	.	.	1	0.80704	64%	Clonal	.	.	.	.	WES		
IDC61T	HIST1H3I	p.A26P	Missense_Mutation	6	27840018	C	G	24	20.83%	Deleterious	PASSENGER/OTHER	Passenger	passenger	.	.	0.89	0.62923	35%	Clonal	.	.	.	YES	WES		
IDC61T	HMCN1	p.V2661L	Missense_Mutation	1	186038896	G	C	133	4.51%	Deleterious	PASSENGER/OTHER	Passenger	passenger	.	.	0.24	0.00002	11%	subclonal	.	.	.	.	WES		
IDC61T	IGSF1	p.Y394C	Missense_Mutation	23	130416483	T	C	61	68.85%	Neutral	PASSENGER/OTHER	Passenger	passenger	.	loh	1	0.94069	90%	Clonal	.	.	.	.	WES		
IDC61T	IL1R1	p.A379P	Missense_Mutation	2	102791190	G	C	168	8.33%	Deleterious	PASSENGER/OTHER	Passenger	passenger	.	loh	0.27	0	16%	subclonal	.	.	.	.	WES		
IDC61T	IMMT	p.Q131E	Missense_Mutation	2	86400803	G	C	300	24.67%	Neutral	PASSENGER/OTHER	Passenger	passenger	.	loh	0.81	0.24894	65%	subclonal	.	.	.	.	WES		
IDC61T	KCNT2	p.S1005L	Missense_Mutation	1	196227521	G	A	434	20.97%	Deleterious	PASSENGER/OTHER	Passenger	passenger	.	.	1	0.924	85%	subclonal	.	.	.	.	WES		
IDC61T	LILRB1	p.T40S	Missense_Mutation	19	55142998	A	T	133	8.27%	Neutral	PASSENGER/OTHER	Passenger	passenger	.	.	0.35	0.00009	20%	subclonal	.	.	.	.	WES		
IDC61T	LNX2	p.E235Q	Missense_Mutation	13	28141929	C	G	121	4.96%	Deleterious	PASSENGER/OTHER	Passenger	passenger	.	.	0.16	0	7%	subclonal	.	.	.	.	WES		
IDC61T	LPHN3	p.T467S	Missense_Mutation	4	62758497	C	G	113	7.08%	Deleterious	PASSENGER/OTHER	Passenger	passenger	.	loh	0.23	0	11%	subclonal	.	.	.	.	WES		
IDC61T	LTN1	p.H908Q	Missense_Mutation	21	30331787	A	C	74	22.97%	Neutral	PASSENGER/OTHER	Passenger	passenger	.	.	0.98	0.76257	59%	Clonal	.	.	.	.	WES		
IDC61T	MACROD2	p.F182S	Missense_Mutation	20	15412054	T	C	477	3.14%	Deleterious	PASSENGER/OTHER	Passenger	passenger	.	.	0.13	0	8%	subclonal	.	.	.	.	WES		
IDC61T	MUC1	p.T112P	Missense_Mutation	1	155161799	T	G	17	35.29%	Neutral	PASSENGER/OTHER	Passenger	passenger	.	.	1	0.8709	51%	Clonal	.	.	.	.	WES		
IDC61T	MYO1A	p.E126G	Missense_Mutation	12	57441140	T	C	43	41.86%	Deleterious	CANCER	Passenger	likely_pathogenic	.	.	1	0.85697	71%	Clonal	.	.	.	.	WES		
IDC61T	NBEAL1	p.I1098T	Missense_Mutation	2	203992635	T	C	83	37.35%	Deleterious	PASSENGER/OTHER	Passenger	passenger	.	loh	1	0.88685	77%	Clonal	.	.	.	.	WES		
IDC61T	NUAK1	p.T461M	Missense_Mutation	12	106461184	G	A	57	21.05%	Deleterious	PASSENGER/OTHER	Passenger	passenger	.	.	0.69	0.3616	40%	subclonal	.	.	.	.	WES		
IDC61T	OR1A2	p.M104L	Missense_Mutation	17	3101122	A	T	143	34.97%	Neutral	PASSENGER/OTHER	Passenger	passenger	.	loh	1	0.9081	81%	Clonal	.	.	.	.	WES		
IDC61T	OR2T1	p.C252S	Missense_Mutation	1	248570049	T	A	362	35.36%	Deleterious	PASSENGER/OTHER	Passenger	passenger	.	.	1	0.96787	95%	Clonal	.	.	.	.	WES		
IDC61T	OR2T4	p.A70V	Missense_Mutation	1	248525091	C	T	226	5.75%	Neutral	PASSENGER/OTHER	Passenger	passenger	.	.	0.3	0	17%	subclonal	.	.	.	.	WES		
IDC61T	OR8D1	p.M59I	Missense_Mutation	11	124180486	C	G	89	12.36%	Deleterious	PASSENGER/OTHER	Passenger	passenger	.	.	0.53	0.06586	30%	subclonal	.	.	.	.	WES		
IDC61T	PACSIN2	p.W83C	Missense_Mutation	22	43287157	C	G	68	30.88%	Deleterious	PASSENGER/OTHER	Passenger	passenger	.	.	1	0.94875	78%	Clonal	.	.	.	.	WES		
IDC61T	PADI1	p.M76K	Missense_Mutation	1	17548919	T	A	46	28.26%	Deleterious	PASSENGER/OTHER	Passenger	passenger	.	loh	0.92	0.71165	53%	Clonal	.	.	.	.	WES		
IDC61T	PAH	p.P69S	Missense_Mutation	12	103288660	G	A	76	21.05%	Deleterious	CANCER	Passenger	likely_pathogenic	.	.	0.69	0.29142	43%	subclonal	.	.	.	.	WES		
IDC61T	PCDHB16	p.S746F	Missense_Mutation	5	140564371	C	T	55	52.73%	Neutral	PASSENGER/OTHER	Passenger	passenger	.	loh	1	0.9333	83%	subclonal	.	.	.	.	WES		
IDC61T	PRPS1L1	p.Q133E	Missense_Mutation	7	18067009	G	C	148	3.38%	.	CANCER	Passenger	passenger	.	loh	0.11	0	4%	subclonal	.	.	.	.	WES		
IDC61T	PTPRJ	p.H402Y	Missense_Mutation	11	48149442	C	T	29	13.79%	Neutral	PASSENGER/OTHER	Passenger	passenger	.	loh	0.31	0.00728	12%	subclonal	.	.	.	.	WES		
IDC61T	RAB11FIP1	p.A1219G	Missense_Mutation	8	37720609	G	C	107	16.82%	Deleterious	PASSENGER/OTHER	Passenger	passenger	.	.	0.55	0.01403	35%	subclonal	.	.	.	.	WES		
IDC61T	RSPH6A	p.I665S	Missense_Mutation	19	46299287	A	C	82	14.63%	Neutral	PASSENGER/OTHER	Passenger	passenger	.	.	0.63	0.23008	36%	subclonal	.	.	.	.	WES		
IDC61T	SDK1	p.D2100G	Missense_Mutation	7	4285355	A	G	22	50.00%	Deleterious	PASSENGER/OTHER	Driver	likely_pathogenic	.	loh	1	0.90045	65%	subclonal	.	.	.	.	WES		
IDC61T	SH3BP4	p.P481R	Missense_Mutation	2	235950855	C	G	41	34.15%	Deleterious	PASSENGER/OTHER	Passenger	passenger	.	loh	1	0.80895	61%	Clonal	.	.	.	.	WES		
IDC61T	SIK2	p.D870N	Missense_Mutation	11	111594680	G	A	78	24.36%	Deleterious	PASSENGER/OTHER	Passenger	passenger	.	.	1	0.85095	69%	Clonal	.	.	.	.	WES		
IDC61T	SLC6A3	p.S517T	Missense_Mutation	5	1406352	C	G	12	83.33%	Neutral	PASSENGER/OTHER	Passenger	passenger	.	loh	1	0.2084	71%	Clonal	.	.	.	.	WES		
IDC61T	SLC7A4	p.A132T	Missense_Mutation	22	21385708	C	T	7	57.14%	Deleterious	CANCER	Passenger	likely_pathogenic	.	.	1	0.85726	44%	Clonal	.	.	.	.	WES		
IDC61T	SPATA20	p.R242Q	Missense_Mutation	17	48626711	G	A	12	16.67%	Neutral	PASSENGER/OTHER	Passenger	passenger	.	loh	0.55	0.40268	15%	subclonal	.	.	.	.	WES		
IDC61T	TOP1MT	p.T405P	Missense_Mutation	8	144400190	T	G	84	19.05%	Neutral	PASSENGER/OTHER	Passenger	passenger	.	.	1	0.82263	63%	Clonal	.	.	.	.	WES		
IDC61T	TP53	p.Y126N	Missense_Mutation	17	7578554	A	T	44	61.36%	Deleterious	CANCER	Passenger	likely_pathogenic	.	loh	1	0.96602	84%	Clonal	TRUE	TRUE	TRUE	YES	WES		
IDC61T	TPD52L2	p.T186P	Missense_Mutation	20	62520553	A	C	171	46.78%	Deleterious	PASSENGER/OTHER	Passenger	passenger	.	.	1	0.97629	93%	Clonal	.	.	.	.	WES		
IDC61T	TRHDE	p.T570I	Missense_Mutation	12	72956000	C	T	61	27.87%	Deleterious	PASSENGER/OTHER	Passenger	passenger	.	.	0.91	0.71538	56%	Clonal	.	.	.	.	WES		
IDC61T	TRIM52	p.F228V	Missense_Mutation	5	180687133	A	C	30	43.33%	Neutral	PASSENGER/OTHER	Passenger	passenger	.	loh	0.98	0.8221	57%	Clonal	.	.	.	.	WES		
IDC61T	TRPS1	p.P880T	Missense_Mutation	8	116599290	G	T	34	8.82%	Neutral	PASSENGER/OTHER	Passenger	passenger	.	.	0.55	0.38618	18%	subclonal	.	.	.	.	WES		
IDC61T	TSPYL2	p.E567K	Missense_Mutation	23	53115273	G	A	211	9.95%	Neutral	PASSENGER/OTHER	Passenger	passenger	.	loh	0.33	0	21%	subclonal	.	.	.	.	WES		
IDC61T	USH2A	p.A4511S	Missense_Mutation	1	215847722	C	A	109	28.44%	Deleterious	PASSENGER/OTHER	Passenger	passenger	.	.	1	0.84709	80%	Clonal	.	.	.	.	WES		
IDC61T	VPS26B	p.V299I	Missense_Mutation	11	134115368	G	A	55	52.73%	Deleterious	.	Passenger	passenger	.	.	1	0.94125	85%	Clonal	.	.	.	.	WES		
IDC61T	YLPM1	p.P543T	Missense_Mutation	14	75248373	C	A	108	50.00%	Deleterious	.	Passenger	passenger	.	loh	1	0.94346	84%	Clonal	.	.	.	.	WES		
IDC61T	ZFAT	p.D154G	Missense_Mutation	8	135622886	T	C	316	11.71%	Deleterious	PASSENGER/OTHER	Passenger	passenger	.	.	0.73	0.2343	54%	subclonal	.	.	.	.	WES		
IDC61T	ZFHX3	p.D1939H	Missense_Mutation	16	72830766	C	G	102	28.43%	Deleterious	PASSENGER/OTHER	Passenger	passenger	.	loh	1	0.87679	75%	subclonal	TRUE	.	TRUE	YES	WES		
IDC61T	ZNF263	p.H338Y	Missense_Mutation	16	3339518	C	T	39	23.08%	Neutral	PASSENGER/OTHER	Passenger	passenger	.	loh	1	0.78413	53%	subclonal	.	.	.	.	WES		
IDC61T	ZNF568	p.L57H	Missense_Mutation	19	37427682	T	A	100	18.00%	Deleterious	PASSENGER/OTHER	Passenger	passenger	.	.	0.77	0.49524	49%	Clonal	.	.	.	.	WES		
IDC61T	BLMH	p.Y169*	Nonsense_Mutation	17	28613877	A	C	110	33.64%	Deleterious	.	.	likely_pathogenic	.	loh	1	0.88155	76%	Clonal	.	.	.	.	WES		
IDC61T	CPT1A	p.Y231*	Nonsense_Mutation	11	68566686	G	T	44	20.45%	Deleterious	.	.	passenger	.	.	0.87	0.64938	44%	Clonal	.	.	.	.	WES		
IDC61T	IL1RAP	p.K46*	Nonsense_Mutation	3	190321988	A	T	114	30.70%	Deleterious	.	.	passenger	.	.	1	0.87546	80%	Clonal	.	.	.	.	WES		
IDC61T	TRIM67	p.Q436*	Nonsense_Mutation	1	231335936	C	T	95	18.95%	Deleterious	.	.	passenger	.	.	1	0.77258	60%	Clonal	.	.	.	.	WES		
IDC61T	AP2M1	p.X142_splice	Splice_Site	3	183898425	GCCTCCAGCATCAGGTAAGCT	G	155	13.55%	.	.	.	passenger	.	.	0.58	0.0247	38%	subclonal	.	.	.	.	WES		
IDC61T	DYX1C1	p.X298_splice	Splice_Site	15	55727258	T	C	74	40.54%	Deleterious	.	.	likely_pathogenic	.	loh	1	0.87079	79%	Clonal	.	.	.	.	WES		
IDC61T	ITPKC	p.X419_splice	Splice_Site	19	41231346	T	G	74	18.92%	Deleterious	.	.	passenger	.	.	0.81	0.58658	48%	Clonal	.	.	.	.	WES		
IDC61T	MLLT4	p.X1027_splice	Splice_Site	6	168343808	G	C	154	66.88%	Deleterious	.	.	likely_pathogenic	.	loh	1	0.9475	96%	Clonal	TRUE	.	.	.	WES		
IDC61T	MORC4	p.X887_splice	Splice_Site	23	106184858	AAGCCCTGTATGAAGGAGTG	A	50	44.00%	.	.	.	likely_pathogenic	.	loh	1	0.85006	76%	Clonal	.	.	.	.	WES		
IDC62T	CD79A	p.Q9Cfs*32	Frame_Shift_Del	19	42381395	CCTCCAAG	C	598	24.92%	.	.	.	likely_pathogenic	.	.	0.79	0.01247	68%	.	TRUE	.	.	YES	MSK-IMPACT		
IDC62T	NOTCH3	p.A2213Tfs*25	Frame_Shift_Del	19	15271791	TCCTGGGACTGC	T	379	29.02%	.	.	.	likely_pathogenic	.	loh	0.64	0	53%	subclonal	.	.	.	YES	MSK-IMPACT		
IDC62T	FGFR4	p.S440P	Missense_Mutation	5	176520473	T	C	717	33.05%	Deleterious	CANCER	Passenger	likely_pathogenic	.	.	1	0.94845	91%	.	TRUE	.	.	YES	MSK-IMPACT		
IDC62T	HIST1H3B	p.K38N	Missense_Mutation	6	26032175	C	G	647	19.47%	Neutral	PASSENGER/OTHER	Passenger	passenger	.	.	1	0.88597	90%	.	TRUE	.	TRUE	YES	MSK-IMPACT		
IDC62T	KMT2A	p.K1133T	Missense_Mutation	11	118348745	A	C	406	53.45%	Deleterious	PASSENGER/OTHER	Driver	likely_pathogenic	.	loh	1	0.73512	95%	Clonal	TRUE	.	TRUE	YES	MSK-IMPACT		
IDC62T	NF1	p.D2736H	Missense_Mutation	17	29687550	G	C	792	23.11%	Deleterious	PASSENGER/OTHER	Passenger	passenger	.	.	0.97	0.85569	84%	Clonal	TRUE	TRUE	TRUE	YES	MSK-IMPACT		
IDC62T	PGR	p.V912L	Missense_Mutation	11	100909915	C	G	410	3.41%	Deleterious	PASSENGER/OTHER	Passenger	passenger	.	loh	0.07	0	4%	subclonal	.	.	.	YES	MSK-IMPACT		
IDC62T	TAP1	p.A609S	Missense_Mutation	6	32815791	C	A	735	30.07%	Deleterious	CANCER	Passenger	likely_pathogenic	.	.	0.96	0.84072	85%	Clonal	.	.	.	.	MSK-IMPACT		
IDC62T	PALB2	p.Q822*	Nonsense_Mutation	16	23641011	G	A	517	30.37%	Deleterious	.	.	likely_pathogenic	.	.	0.97	0.8553	83%	Clonal	TRUE	.	.	YES	MSK-IMPACT		
IDC62T	EPHA7	p.X278_splice	Splice_Site	6	94120218	C	G	628	25.00%	Deleterious	PASSENGER/OTHER	Passenger	passenger	.	.	1	0.92587	87%	Clonal	.	.	.	YES	MSK-IMPACT		
IDC63T	KMT2A	p.R736Sfs*3	Frame_Shift_Del	11	118344076	AAAAAGGAAAAG	A	186	6.99%	.	.	.	likely_pathogenic	.	.	0.34	0	20%	subclonal	TRUE	.	TRUE	YES	MSK-IMPACT		
IDC8T	ACVR1C	p.L16F	Missense_Mutation	2	158485111	G	A	36	11.11%	Neutral	CANCER	Passenger	passenger	.	loh	0.66	0.38868	25%	Subclonal	.	.	.	.	WES		
IDC8T	AGMO	p.L366F	Missense_Mutation	7	15405826	G	A	143	9.79%	Deleterious	PASSENGER/OTHER	Passenger	passenger	.	.	0.58	0.08718	35%	Subclonal	.	.	.	.	WES		
IDC8T	ALPI	p.V477I	Missense_Mutation	2	233323698	G	A	211	12.32%	Neutral	PASSENGER/OTHER	Passenger	passenger	.	loh	0.74	0.27149	50%	Subclonal	.	.	.	.	WES		
IDC8T	C10orf12	p.T1182A	Missense_Mutation	10	98744691	A	G	175	5.14%	Neutral	PASSENGER/OTHER	Passenger	passenger	.	loh	0.26	0	13%	Subclonal	.	.	.	.	WES		
IDC8T	C10orf95	p.G162A	Missense_Mutation	10	104210503	C	G	10	40.00%	Neutral	.	Passenger	passenger	.	.	1	0.78898	43%	Clonal	.	.	.	.	WES		
IDC8T	C21orf2	p.G323E	Missense_Mutation	21	45750377	C	T	230	13.04%	Neutral	PASSENGER/OTHER	Passenger	passenger	.	.	0.78	0.36708	55%	Subclonal	.	.	.	.	WES		
IDC8T	CACNA1G	p.P297H	Missense_Mutation	17	48650058	C	A	208	6.73%	Neutral	PASSENGER/OTHER	Passenger	passenger	.	.	0.47	0.0052	28%	Subclonal	.	.	.	.	WES		
IDC8T	CAT	p.Q395L	Missense_Mutation	11	34482925	A	T	86	18.60%	Neutral	.	Passenger	passenger	.	.	1	0.74586	61%	Clonal	.	.	.	.	WES		
IDC8T	CCDC160	p.N170S	Missense_Mutation	X	133379339	A	G	95	15.79%	Neutral	.	Passenger	passenger	.	loh	0.79	0.54461	47%	Clonal	.	.	.	.	WES		
IDC8T	CNTNAP4	p.I1180T	Missense_Mutation	16	76592411	T	C	125	8.80%	Deleterious	PASSENGER/OTHER	Passenger	passenger	.	.	0.53	0.05461	29%	Subclonal	.	.	.	.	WES		
IDC8T	CPAMD8	p.R1135Q	Missense_Mutation	19	17038926	C	T	139	13.67%	Deleterious	PASSENGER/OTHER	Passenger	passenger	.	.	0.82	0.50598	52%	Clonal	.	.	.	.	WES		
IDC8T	DERL2	p.L158F	Missense_Mutation	17	5383758	G	A	47	8.51%	Deleterious	PASSENGER/OTHER	Passenger	passenger	.	loh	0.42	0.13361	17%	Subclonal	.	.	.	.	WES		
IDC8T	FOXRED1	p.G71S	Missense_Mutation	11	126141457	G	A	89	5.62%	Deleterious	PASSENGER/OTHER	Passenger	passenger	.	loh	0.28	0.00132	12%	Subclonal	.	.	.	.	WES		
IDC8T	GGT5	p.R362C	Missense_Mutation	22	24622189	G	A	24	12.50%	Deleterious	PASSENGER/OTHER	Passenger	passenger	.	.	0.75	0.45304	23%	Subclonal	.	.	.	.	WES		
IDC8T	HTT	p.A3071G	Missense_Mutation	4	3240702	C	G	96	17.71%	Deleterious	PASSENGER/OTHER	Passenger	passenger	.	loh	0.88	0.6758	54%	Clonal	.	.	.	.	WES		
IDC8T	IFT172	p.E1267K	Missense_Mutation	2	27676519	C	T	122	13.11%	Deleterious	PASSENGER/OTHER	Passenger	passenger	.	.	0.78	0.45719	48%	Subclonal	.	.	.	.	WES		
IDC8T	INADL	p.R439Q	Missense_Mutation	1	62263014	G	A	60	8.33%	Deleterious	PASSENGER/OTHER	Passenger	passenger	.	loh	0.41	0.07443	18%	Subclonal	.	.	.	.	WES		
IDC8T	KIF2A	p.G554R	Missense_Mutation	5	61668278	G	C	32	12.50%	Deleterious	PASSENGER/OTHER	Passenger	passenger	.	.	0.75	0.45296	27%	Subclonal	.	.	.	.	WES		
IDC8T	LY75	p.F536L	Missense_Mutation	2	160735001	A	C	195	13.85%	Deleterious	PASSENGER/OTHER	Driver	likely_pathogenic	.	loh	0.83	0.50686	57%	Clonal	.	.	.	.	WES		
IDC8T	MAGEB3	p.R107G	Missense_Mutation	X	30254360	C	G	60	18.33%	Neutral	PASSENGER/OTHER	Passenger	passenger	.	loh	0.91	0.68231	48%	Clonal	.	.	.	.	WES		
IDC8T	MAPKBP1	p.S430R	Missense_Mutation	15	42107558	C	A	114	11.40%	Deleterious	PASSENGER/OTHER	Passenger	passenger	.	.	0.8	0.48691	46%	Subclonal	.	.	.	.	WES		
IDC8T	MIPOL1	p.Q404E	Missense_Mutation	14	37969291	C	G	81	13.58%	Neutral	PASSENGER/OTHER	Passenger	passenger	.	.	0.81	0.51033	44%	Clonal	.	.	.	.	WES		
IDC8T	OSBPL8	p.L632Q	Missense_Mutation	12	76767146	A	T	73	10.96%	Deleterious	PASSENGER/OTHER	Passenger	passenger	.	.	0.65	0.31619	33%	Subclonal	.	.	.	.	WES		
IDC8T	PCLO	p.S3119F	Missense_Mutation	7	82580548	G	A	130	5.38%	Deleterious	PASSENGER/OTHER	Passenger	passenger	.	.	0.32	0.00083	15%	Subclonal	.	.	.	.	WES		
IDC8T	PGBD5	p.N348K	Missense_Mutation	1	230468612	G	C	133	16.54%	Deleterious	PASSENGER/OTHER	Passenger	passenger	.	.	1	0.81557	71%	Clonal	.	.	.	.	WES		
IDC8T	PHYH	p.P41T	Missense_Mutation	10	13340200	G	T	117	12.82%	Neutral	PASSENGER/OTHER	Passenger	passenger	.	.	0.77	0.43076	46%	Subclonal	.	.	.	.	WES		
IDC8T	PXDN	p.E1191K	Missense_Mutation	2	1651981	C	T	353	8.78%	Deleterious	PASSENGER/OTHER	Passenger	passenger	.	.	0.52	0.00022	37%	Subclonal	.	.	.	.	WES		
IDC8T	PXDNL	p.A969T	Missense_Mutation	8	52321279	C	T	223	16.59%	Neutral	PASSENGER/OTHER	Passenger	passenger	.	.	0.99	0.76159	69%	Clonal	.	.	.	.	WES		
IDC8T	SLC30A8	p.A175V	Missense_Mutation	8	118170035	C	T	91	7.69%	Deleterious	PASSENGER/OTHER	Passenger	passenger	.	.	0.54	0.15621	26%	Subclonal	.	.	.	.	WES		
IDC8T	SLK	p.E389K	Missense_Mutation	10	105762101	G	A	179	16.76%	Deleterious	PASSENGER/OTHER	Passenger	passenger	.	.	1	0.75221	67%	Clonal	.	.	.	.	WES		
IDC8T	SPHKAP	p.E551Q	Missense_Mutation	2	228883919	C	G	201	10.45%	Deleterious	PASSENGER/OTHER	Passenger	passenger	.	loh	0.62	0.06948	41%	Subclonal	.	.	.	.	WES		
IDC8T	TLE6	p.D390N	Missense_Mutation	19	2989707	G	A	115	15.65%	Neutral	PASSENGER/OTHER	Passenger	passenger	.	.	0.93	0.65676	57%	Clonal	.	.	.	.	WES		
IDC8T	TMEM57	p.R591Q	Missense_Mutation	1	25818055	G	A	38	7.89%	Deleterious	PASSENGER/OTHER	Passenger	passenger	.	loh	0.39	0.15189	14%	Subclonal	.	.	.	.	WES		
IDC8T	TMPRSS13	p.A77G	Missense_Mutation	11	117789345	G	C	13	23.08%	Neutral	PASSENGER/OTHER	Passenger	passenger	.	loh	0.92	0.33809	26%	Subclonal	.	.	.	.	WES		
IDC8T	TOR1AIP1	p.E89Q	Missense_Mutation	1	179851902	G	C	368	8.42%	Neutral	PASSENGER/OTHER	Passenger	passenger	.	.	0.59	0.00567	41%	Subclonal	.	.	.	.	WES		
IDC8T	TRPM3	p.W175C	Missense_Mutation	9	73461445	C	G	124	18.55%	Deleterious	PASSENGER/OTHER	Passenger	passenger	.	.	1	0.78269	67%	Clonal	.	.	.	.	WES		
IDC8T	UNC13B	p.Q164L	Missense_Mutation	9	35259012	A	T	48	10.42%	Neutral	PASSENGER/OTHER	Passenger	passenger	.	.	0.62	0.32892	26%	Subclonal	.	.	.	.	WES		
IDC8T	ZNF331	p.E212K	Missense_Mutation	19	54080448	G	A	74	13.51%	Neutral	PASSENGER/OTHER	Passenger	passenger	.	.	0.81	0.50597	43%	Clonal	TRUE	.	.	.	WES		
IDC8T	ZNF345	p.E227D	Missense_Mutation	19	37368413	G	C	84	8.33%	Neutral	PASSENGER/OTHER	Passenger	passenger	.	.	0.5	0.09735	24%	Subclonal	.	.	.	.	WES		
IDC8T	ZNF516	p.A939T	Missense_Mutation	18	74091255	C	T	160	8.13%	Neutral	PASSENGER/OTHER	Passenger	passenger	.	.	0.49	0.01136	28%	Subclonal	.	.	.	.	WES		
IDC8T	ZNF536	p.P227L	Missense_Mutation	19	30935149	C	T	38	28.95%	Neutral	PASSENGER/OTHER	Passenger	passenger	.	.	1	0.87433	64%	Clonal	.	.	.	.	WES		
IDC9T	CCDC82	p.E229Kfs*4	Frame_Shift_Del	11	96117226	TC	T	122	22.95%	.	.	.	likely_pathogenic	.	loh	0.97	0.60234	66%	Clonal	.	.	.	.	WES		
IDC9T	ABCF2	p.R210Q	Missense_Mutation	7	150920857	C	T	88	5.68%	Deleterious	PASSENGER/OTHER	Passenger	passenger	.	.	0.41	0.03423	18%	Subclonal	.	.	.	.	WES		
IDC9T	AKR1E2	p.M105L	Missense_Mutation	10	4875647	A	T	79	6.33%	Deleterious	PASSENGER/OTHER	Passenger	passenger	.	.	0.33	0.00452	14%	Subclonal	.	.	.	.	WES		
IDC9T	AP3D1	p.R393C	Missense_Mutation	19	2121235	G	A	98	5.10%	Deleterious	PASSENGER/OTHER	Passenger	passenger	.	loh	0.22	0.00001	9%	Subclonal	.	.	.	.	WES		
IDC9T	ARFGAP3	p.R77Q	Missense_Mutation	22	43236981	C	T	96	5.21%	Deleterious	PASSENGER/OTHER	Passenger	passenger	.	loh	0.22	0.00001	9%	Subclonal	.	.	.	.	WES		
IDC9T	BAG4	p.D241Y	Missense_Mutation	8	38066618	G	T	181	7.18%	Deleterious	PASSENGER/OTHER	Passenger	passenger	.	.	0.59	0.07152	35%	Subclonal	.	.	.	.	WES		
IDC9T	BCAR3	p.R670Q	Missense_Mutation	1	94033374	C	T	38	60.53%	Deleterious	PASSENGER/OTHER	Passenger	passenger	.	loh	1	0.23421	84%	Subclonal	.	.	.	.	WES		
IDC9T	BTBD11	p.G214A	Missense_Mutation	12	107713358	G	C	53	18.87%	Deleterious	PASSENGER/OTHER	Passenger	passenger	.	.	1	0.5855	54%	Clonal	.	.	.	.	WES		
IDC9T	C20orf196	p.A74T	Missense_Mutation	20	5843711	G	A	93	5.38%	Neutral	PASSENGER/OTHER	Passenger	passenger	.	.	0.34	0.00576	14%	Subclonal	.	.	.	.	WES		
IDC9T	C6orf57	p.L43R	Missense_Mutation	6	71289180	T	G	65	9.23%	Neutral	PASSENGER/OTHER	Passenger	passenger	.	loh	0.39	0.00941	18%	Subclonal	.	.	.	.	WES		
IDC9T	CAMK2G	p.G328S	Missense_Mutation	10	75597234	C	T	58	8.62%	Neutral	PASSENGER/OTHER	Passenger	passenger	.	loh	0.37	0.00994	16%	Subclonal	.	.	.	.	WES		
IDC9T	CD28	p.A35V	Missense_Mutation	2	204591407	C	T	28	14.29%	Neutral	CANCER	Passenger	passenger	.	.	0.75	0.31631	27%	Subclonal	.	.	.	.	WES		
IDC9T	CD6	p.D663N	Missense_Mutation	11	60786770	G	A	138	4.35%	Neutral	PASSENGER/OTHER	Passenger	passenger	.	.	0.23	0	10%	Subclonal	.	.	.	.	WES		
IDC9T	CHD8	p.R286C	Missense_Mutation	14	21897482	G	A	98	6.12%	Deleterious	PASSENGER/OTHER	Driver	likely_pathogenic	.	.	0.32	0.0011	15%	Subclonal	.	.	TRUE	.	WES		
IDC9T	CHST3	p.A110T	Missense_Mutation	10	73767117	G	A	39	10.26%	Neutral	PASSENGER/OTHER	Passenger	passenger	.	loh	0.43	0.06305	17%	Subclonal	.	.	.	.	WES		
IDC9T	CKMT2	p.V126I	Missense_Mutation	5	80550239	G	A	135	4.44%	Deleterious	PASSENGER/OTHER	Driver	likely_pathogenic	.	loh	0.19	0	8%	Subclonal	.	.	.	.	WES		
IDC9T	CLK3	p.R343Q	Missense_Mutation	15	74917293	G	A	89	5.62%	Deleterious	PASSENGER/OTHER	Passenger	passenger	.	.	0.35	0.00884	15%	Subclonal	.	.	.	.	WES		
IDC9T	CLPB	p.F254L	Missense_Mutation	11	72070027	G	C	125	14.40%	Deleterious	CANCER	Passenger	likely_pathogenic	.	.	1	0.59652	61%	Clonal	.	.	.	.	WES		
IDC9T	CMYA5	p.G3363R	Missense_Mutation	5	79034675	G	A	92	5.43%	Neutral	PASSENGER/OTHER	Passenger	passenger	.	loh	0.23	0.00002	10%	Subclonal	.	.	.	.	WES		
IDC9T	CMYA5	p.R717H	Missense_Mutation	5	79026738	G	A	95	5.26%	Neutral	PASSENGER/OTHER	Passenger	passenger	.	loh	0.22	0.00001	9%	Subclonal	.	.	.	.	WES		
IDC9T	COL22A1	p.P1305Q	Missense_Mutation	8	139628254	G	T	81	11.11%	Deleterious	PASSENGER/OTHER	Passenger	passenger	.	.	0.92	0.44468	44%	Subclonal	.	.	.	.	WES		
IDC9T	COL5A1	p.D422N	Missense_Mutation	9	137623441	G	A	82	6.10%	Deleterious	PASSENGER/OTHER	Passenger	passenger	.	.	0.32	0.00301	14%	Subclonal	.	.	TRUE	.	WES		
IDC9T	CUL5	p.N153I	Missense_Mutation	11	107923433	A	T	41	58.54%	Neutral	PASSENGER/OTHER	Passenger	passenger	.	loh	1	0.75108	83%	Clonal	.	.	.	.	WES		
IDC9T	CYP4B1	p.R173Q	Missense_Mutation	1	47279176	G	A	64	7.81%	Neutral	PASSENGER/OTHER	Passenger	passenger	.	loh	0.41	0.02929	18%	Subclonal	.	.	.	.	WES		
IDC9T	DACH1	p.G82S	Missense_Mutation	13	72440664	C	T	6	83.33%	Neutral	PASSENGER/OTHER	Passenger	passenger	.	.	1	0.25883	53%	Subclonal	.	.	.	.	WES		
IDC9T	DDX20	p.V14L	Missense_Mutation	1	112298586	G	C	128	9.38%	Neutral	PASSENGER/OTHER	Passenger	passenger	.	.	0.77	0.32032	43%	Subclonal	.	.	.	.	WES		
IDC9T	DENND3	p.A852T	Missense_Mutation	8	142188253	G	A	139	4.32%	Deleterious	PASSENGER/OTHER	Driver	likely_pathogenic	.	.	0.36	0.00536	16%	Subclonal	.	.	.	.	WES		
IDC9T	DOT1L	p.D770N	Missense_Mutation	19	2216664	G	A	90	6.67%	Deleterious	PASSENGER/OTHER	Passenger	passenger	.	loh	0.28	0.00012	13%	Subclonal	.	.	.	YES	WES		
IDC9T	DPP9	p.R872C	Missense_Mutation	19	4676641	G	A	96	5.21%	Deleterious	PASSENGER/OTHER	Passenger	passenger	.	.	0.27	0.00041	12%	Subclonal	.	.	.	.	WES		
IDC9T	DPYS	p.I273L	Missense_Mutation	8	105441906	T	A	118	16.10%	Deleterious	PASSENGER/OTHER	Passenger	passenger	.	.	1	0.70336	69%	Clonal	.	.	.	.	WES		
IDC9T	EFNB2	p.T180R	Missense_Mutation	13	107147303	G	C	89	17.98%	Deleterious	PASSENGER/OTHER	Passenger	passenger	.	.	0.94	0.51617	56%	Clonal	.	.	.	.	WES		
IDC9T	EGFLAM	p.R856T	Missense_Mutation	5	38448481	G	C	71	11.27%	Deleterious	PASSENGER/OTHER	Passenger	passenger	.	.	0.7	0.26024	35%	Subclonal	.	.	.	.	WES		
IDC9T	EPX	p.P19A	Missense_Mutation	17	56270262	C	G	45	20.00%	Neutral	PASSENGER/OTHER	Passenger	passenger	.	.	1	0.70928	63%	Clonal	.	.	.	.	WES		
IDC9T	ERC2	p.A724V	Missense_Mutation	3	56026169	G	A	102	5.88%	Neutral	PASSENGER/OTHER	Passenger	passenger	.	loh	0.25	0.00001	11%	Subclonal	.	.	.	.	WES		
IDC9T	FAM155B	p.L438F	Missense_Mutation	X	68749692	C	T	133	6.02%	Deleterious	PASSENGER/OTHER	Passenger	passenger	.	.	0.32	0.00009	16%	Subclonal	.	.	.	.	WES		
IDC9T	GALNT16	p.L79M	Missense_Mutation	14	69787485	C	A	42	16.67%	Deleterious	PASSENGER/OTHER	Passenger	passenger	.	loh	0.71	0.26775	34%	Subclonal	.	.	.	.	WES		
IDC9T	GALNT18	p.L445P	Missense_Mutation	11	11354323	A	G	47	46.81%	Deleterious	PASSENGER/OTHER	Driver	likely_pathogenic	.	loh	1	0.90118	80%	Clonal	.	.	.	.	WES		
IDC9T	GPR173	p.G86S	Missense_Mutation	X	53106059	G	A	94	5.32%	Deleterious	PASSENGER/OTHER	Passenger	passenger	.	.	0.28	0.00055	12%	Subclonal	.	.	.	.	WES		
IDC9T	GTF3C1	p.R230Q	Missense_Mutation	16	27549168	C	T	91	5.49%	Deleterious	PASSENGER/OTHER	Passenger	passenger	.	loh	0.34	0.00715	15%	Subclonal	.	.	.	.	WES		
IDC9T	H2AFY	p.S2L	Missense_Mutation	5	134724779	G	A	39	10.26%	Deleterious	PASSENGER/OTHER	Passenger	passenger	.	.	0.64	0.24405	24%	Subclonal	.	.	.	.	WES		
IDC9T	HAPLN2	p.C170F	Missense_Mutation	1	156594212	G	T	128	8.59%	Deleterious	PASSENGER/OTHER	Passenger	passenger	.	.	0.62	0.1279	35%	Subclonal	.	.	.	.	WES		
IDC9T	HAPLN2	p.C170W	Missense_Mutation	1	156594213	C	G	130	9.23%	Deleterious	PASSENGER/OTHER	Driver	likely_pathogenic	.	.	0.67	0.17746	38%	Subclonal	.	.	.	.	WES		
IDC9T	HEBP1	p.R103W	Missense_Mutation	12	13140177	G	A	122	4.92%	Deleterious	PASSENGER/OTHER	Passenger	passenger	.	.	0.31	0.00071	14%	Subclonal	.	.	.	.	WES		
IDC9T	HELZ	p.I469L	Missense_Mutation	17	65174800	T	G	191	3.66%	Deleterious	PASSENGER/OTHER	Passenger	passenger	.	.	0.49	0.05514	23%	Subclonal	.	.	.	.	WES		
IDC9T	HERC2	p.A4800T	Missense_Mutation	15	28357016	C	T	161	4.35%	Deleterious	PASSENGER/OTHER	Passenger	passenger	.	.	0.27	0.00002	13%	Subclonal	.	.	.	.	WES		
IDC9T	HSP90AB1	p.R197Q	Missense_Mutation	6	44217833	G	A	48	8.33%	Deleterious	PASSENGER/OTHER	Passenger	passenger	.	.	0.52	0.14413	20%	Subclonal	TRUE	.	.	.	WES		
IDC9T	ITPR1	p.E2035K	Missense_Mutation	3	4817094	G	A	95	5.26%	Deleterious	CANCER	Passenger	likely_pathogenic	.	loh	0.22	0.00001	9%	Subclonal	.	.	.	.	WES		
IDC9T	KCNK5	p.T128M	Missense_Mutation	6	39162452	G	A	76	6.58%	Deleterious	PASSENGER/OTHER	Passenger	passenger	.	.	0.41	0.03213	18%	Subclonal	.	.	.	.	WES		
IDC9T	KIAA0947	p.R1849H	Missense_Mutation	5	5464993	G	A	105	5.71%	Deleterious	PASSENGER/OTHER	Passenger	passenger	.	.	0.36	0.00472	16%	Subclonal	.	.	.	.	WES		
IDC9T	KIF5A	p.R191H	Missense_Mutation	12	57960979	G	A	117	5.13%	Deleterious	PASSENGER/OTHER	Driver	likely_pathogenic	.	.	0.32	0.00126	15%	Subclonal	.	.	.	.	WES		
IDC9T	KIFC1	p.R352Q	Missense_Mutation	6	33372927	G	A	198	4.55%	Neutral	PASSENGER/OTHER	Passenger	passenger	.	.	0.28	0	15%	Subclonal	.	.	.	.	WES		
IDC9T	KLHDC9	p.A2V	Missense_Mutation	1	161068330	C	T	43	9.30%	Neutral	PASSENGER/OTHER	Passenger	passenger	.	.	0.67	0.26878	25%	Subclonal	.	.	.	.	WES		
IDC9T	KLHL32	p.M396L	Missense_Mutation	6	97562217	A	T	36	25.00%	Deleterious	PASSENGER/OTHER	Passenger	passenger	.	loh	1	0.53665	50%	Clonal	.	.	.	.	WES		
IDC9T	KNDC1	p.G740R	Missense_Mutation	10	135012230	G	A	30	26.67%	Neutral	PASSENGER/OTHER	Passenger	passenger	.	loh	1	0.7113	56%	Clonal	.	.	.	.	WES		
IDC9T	KRT24	p.F64Y	Missense_Mutation	17	38859755	A	T	49	38.78%	Neutral	PASSENGER/OTHER	Passenger	passenger	.	.	1	0.80061	78%	Clonal	.	.	.	.	WES		
IDC9T	KRT36	p.D177N	Missense_Mutation	17	39644907	C	T	68	10.29%	Deleterious	PASSENGER/OTHER	Passenger	passenger	.	.	0.64	0.20486	31%	Subclonal	.	.	.	.	WES		
IDC9T	LAMA2	p.S2452L	Missense_Mutation	6	129794413	C	T	50	8.00%	Neutral	PASSENGER/OTHER	Passenger	passenger	.	loh	0.34	0.01203	13%	Subclonal	.	.	.	.	WES		
IDC9T	LAMB3	p.G815C	Missense_Mutation	1	209796440	C	A	82	7.32%	Deleterious	PASSENGER/OTHER	Passenger	passenger	.	.	0.75	0.31344	33%	Subclonal	.	.	.	.	WES		
IDC9T	LOXL1	p.S277I	Missense_Mutation	15	74219954	G	T	56	25.00%	Deleterious	PASSENGER/OTHER	Passenger	passenger	.	.	1	0.75447	67%	Clonal	.	.	.	.	WES		

IDC9T	*LRRK2*	p.R1728C	Missense_Mutation	12	40715848	C	T	99	5.05%	Deleterious	CANCER	Passenger	likely_pathogenic	.	.	0.32	0.00296	13%	Subclonal	.	TRUE	.	.	WES		
IDC9T	*MAPK8IP3*	p.A1095V	Missense_Mutation	16	1817614	C	T	85	5.88%	Deleterious	PASSENGER/OTHER	Passenger	passenger	.	loh	0.37	0.01339	16%	Subclonal	.	.	.	.	WES		
IDC9T	*MC5R*	p.V240I	Missense_Mutation	18	13826482	G	A	82	13.41%	Neutral	PASSENGER/OTHER	Passenger	passenger	.	.	0.57	0.05601	32%	Subclonal	.	.	.	.	WES		
IDC9T	*MCM2*	p.R115Q	Missense_Mutation	3	127323558	G	A	84	5.95%	Deleterious	PASSENGER/OTHER	Passenger	passenger	.	.	0.37	0.01482	16%	Subclonal	.	.	.	.	WES		
IDC9T	*MCM3*	p.A485G	Missense_Mutation	6	52141121	G	C	30	20.00%	Deleterious	PASSENGER/OTHER	Passenger	passenger	.	.	1	0.54904	44%	Clonal	.	.	.	.	WES		
IDC9T	*MED22*	p.P180L	Missense_Mutation	9	136208419	G	A	57	15.79%	Neutral	.	Passenger	passenger	.	.	0.83	0.382	42%	Subclonal	.	.	.	.	WES		
IDC9T	*MLNR*	p.R246C	Missense_Mutation	13	49795209	C	T	132	4.55%	Neutral	PASSENGER/OTHER	Passenger	passenger	.	.	0.24	0.00001	11%	Subclonal	.	.	.	.	WES		
IDC9T	*MMAA*	p.R326C	Missense_Mutation	4	146576305	C	T	108	5.56%	Deleterious	PASSENGER/OTHER	Passenger	passenger	.	.	0.35	0.00342	16%	Subclonal	.	.	.	.	WES		
IDC9T	*MN1*	p.L1199V	Missense_Mutation	22	28192937	G	C	115	5.22%	Deleterious	CANCER	Passenger	likely_pathogenic	.	loh	0.22	0	10%	Subclonal	TRUE	.	.	.	WES		
IDC9T	*MTX1*	p.G13R	Missense_Mutation	1	155178632	G	A	143	9.79%	Neutral	PASSENGER/OTHER	Passenger	passenger	.	.	0.71	0.21737	42%	Subclonal	.	.	.	.	WES		
IDC9T	*MUC17*	p.T1808N	Missense_Mutation	7	100680120	C	A	53	13.21%	Neutral	PASSENGER/OTHER	Passenger	passenger	.	.	0.69	0.25566	33%	Subclonal	.	.	TRUE	.	WES		
IDC9T	*MYH14*	p.R1593Q	Missense_Mutation	19	50792718	G	A	83	7.23%	Deleterious	PASSENGER/OTHER	Passenger	passenger	.	.	0.45	0.04001	21%	Subclonal	.	.	.	.	WES		
IDC9T	*NFAT5*	p.R217C	Missense_Mutation	16	69681326	C	T	65	7.69%	Deleterious	PASSENGER/OTHER	Passenger	passenger	.	.	0.48	0.08186	21%	Subclonal	.	.	.	.	WES		
IDC9T	*NGFR*	p.V161M	Missense_Mutation	17	47583933	G	A	229	3.93%	Neutral	PASSENGER/OTHER	Passenger	passenger	.	.	0.44	0.01171	23%	Subclonal	.	.	.	.	WES		
IDC9T	*NID2*	p.A1213T	Missense_Mutation	14	52477679	C	T	113	5.31%	Deleterious	PASSENGER/OTHER	Passenger	passenger	.	.	0.33	0.00198	15%	Subclonal	.	.	.	.	WES		
IDC9T	*NLN*	p.V315I	Missense_Mutation	5	65081722	G	A	57	8.77%	Deleterious	PASSENGER/OTHER	Passenger	passenger	.	loh	0.37	0.01167	16%	Subclonal	.	.	.	.	WES		
IDC9T	*NOTCH3*	p.A1127V	Missense_Mutation	19	15290255	G	A	87	5.75%	Deleterious	PASSENGER/OTHER	Passenger	passenger	.	.	0.3	0.0015	13%	Subclonal	.	.	.	YES	WES		
IDC9T	*NRXN1*	p.V236M	Missense_Mutation	2	51254706	C	T	96	5.21%	Neutral	PASSENGER/OTHER	Passenger	passenger	.	.	0.32	0.00415	14%	Subclonal	.	.	.	.	WES		
IDC9T	*NT5C2*	p.R526W	Missense_Mutation	10	104849539	G	A	75	6.67%	Deleterious	PASSENGER/OTHER	Passenger	passenger	.	loh	0.28	0.0005	12%	Subclonal	TRUE	.	.	.	WES		
IDC9T	*NUDT18*	p.R123C	Missense_Mutation	8	21965653	G	A	81	6.17%	Deleterious	PASSENGER/OTHER	Passenger	passenger	.	loh	0.26	0.00016	11%	Subclonal	.	.	.	.	WES		
IDC9T	*NYX*	p.D168N	Missense_Mutation	X	41333208	G	A	83	6.02%	Neutral	PASSENGER/OTHER	Passenger	passenger	.	.	0.32	0.00262	13%	Subclonal	.	.	.	.	WES		
IDC9T	*OBSCN*	p.G4216R	Missense_Mutation	1	228482080	G	A	94	5.32%	Deleterious	PASSENGER/OTHER	Passenger	passenger	.	.	0.54	0.14716	23%	Subclonal	.	.	.	.	WES		
IDC9T	*OLFM2*	p.V382M	Missense_Mutation	19	9965083	C	T	105	6.67%	Deleterious	PASSENGER/OTHER	Passenger	passenger	.	.	0.35	0.00137	17%	Subclonal	.	.	.	.	WES		
IDC9T	*OR5H15*	p.V225D	Missense_Mutation	3	97888217	T	A	37	21.62%	Neutral	PASSENGER/OTHER	Passenger	passenger	.	.	1	0.61529	52%	Clonal	.	.	.	.	WES		
IDC9T	*PABPN1L*	p.P277L	Missense_Mutation	16	88930171	G	A	48	10.42%	Deleterious	PASSENGER/OTHER	Passenger	passenger	.	.	0.55	0.13929	23%	Subclonal	.	.	.	.	WES		
IDC9T	*PCK2*	p.R189H	Missense_Mutation	14	24567789	G	A	129	4.65%	Deleterious	PASSENGER/OTHER	Passenger	passenger	.	.	0.24	0.00001	11%	Subclonal	.	.	.	.	WES		
IDC9T	*PCSK1N*	p.P10S	Missense_Mutation	X	48693907	G	A	23	17.39%	Neutral	PASSENGER/OTHER	Passenger	passenger	.	.	0.91	0.39706	31%	Subclonal	.	.	.	.	WES		
IDC9T	*PDE2A*	p.D142G	Missense_Mutation	11	72308562	T	C	23	30.43%	Neutral	PASSENGER/OTHER	Passenger	passenger	.	.	1	0.75235	56%	Clonal	.	.	.	.	WES		
IDC9T	*PDGFD*	p.E121K	Missense_Mutation	11	103866942	C	T	94	5.32%	Deleterious	PASSENGER/OTHER	Passenger	passenger	.	loh	0.23	0.00001	9%	Subclonal	.	.	.	.	WES		
IDC9T	*PDHA2*	p.R261C	Missense_Mutation	4	96762082	C	T	144	4.17%	Deleterious	PASSENGER/OTHER	Passenger	passenger	.	.	0.22	0	10%	Subclonal	.	.	.	.	WES		
IDC9T	*PDXDC1*	p.E540K	Missense_Mutation	16	15126764	G	A	99	6.06%	Deleterious	PASSENGER/OTHER	Passenger	passenger	.	loh	0.38	0.00882	17%	Subclonal	.	.	.	.	WES		
IDC9T	*PIGR*	p.R571C	Missense_Mutation	1	207106506	G	A	140	4.29%	Neutral	PASSENGER/OTHER	Passenger	passenger	.	.	0.44	0.03729	20%	Subclonal	.	.	.	.	WES		
IDC9T	*PLA2G4B*	p.C920F	Missense_Mutation	15	42139653	G	T	192	34.90%	Neutral	PASSENGER/OTHER	Passenger	passenger	.	.	1	0.90089	92%	Clonal	.	.	.	.	WES		
IDC9T	*PLIN4*	p.L613V	Missense_Mutation	19	4512093	G	C	140	13.57%	Neutral	PASSENGER/OTHER	Passenger	passenger	.	.	0.71	0.17142	46%	Subclonal	.	.	.	.	WES		
IDC9T	*PLXNB3*	p.A359G	Missense_Mutation	X	153033289	C	G	71	15.49%	Neutral	PASSENGER/OTHER	Passenger	passenger	.	.	0.66	0.15978	37%	Subclonal	.	.	.	.	WES		
IDC9T	*PMP22*	p.P122L	Missense_Mutation	17	15134352	G	A	15	20.00%	Neutral	PASSENGER/OTHER	Passenger	passenger	.	loh	0.85	0.35951	25%	Subclonal	.	.	.	.	WES		
IDC9T	*POLR3B*	p.V191M	Missense_Mutation	12	106772119	G	A	50	10.00%	Deleterious	PASSENGER/OTHER	Passenger	passenger	.	.	0.62	0.2163	26%	Subclonal	.	.	.	.	WES		
IDC9T	*PRR12*	p.G963S	Missense_Mutation	19	50100479	G	A	81	6.17%	Neutral	PASSENGER/OTHER	Passenger	passenger	.	.	0.39	0.01997	17%	Subclonal	.	.	.	.	WES		
IDC9T	*PSAPL1*	p.R369W	Missense_Mutation	4	7435502	G	A	90	5.56%	Neutral	PASSENGER/OTHER	Passenger	passenger	.	.	0.29	0.00098	12%	Subclonal	.	.	.	.	WES		
IDC9T	*PSTK*	p.G51V	Missense_Mutation	10	124740147	G	T	91	10.99%	Neutral	PASSENGER/OTHER	Passenger	passenger	.	loh	0.69	0.22029	37%	Subclonal	.	.	.	.	WES		
IDC9T	*RAB7A*	p.R197W	Missense_Mutation	3	128532230	C	T	41	12.20%	Neutral	CANCER	Passenger	passenger	.	.	0.76	0.32403	31%	Subclonal	.	.	.	.	WES		
IDC9T	*RBFOX1*	p.N301I	Missense_Mutation	16	7703901	A	T	66	28.79%	Deleterious	PASSENGER/OTHER	Passenger	passenger	.	loh	1	0.86231	75%	Clonal	.	.	.	.	WES		
IDC9T	*RFFL*	p.E121K	Missense_Mutation	17	33348620	C	T	85	7.06%	Deleterious	PASSENGER/OTHER	Passenger	passenger	.	.	0.44	0.03361	20%	Subclonal	.	.	.	.	WES		
IDC9T	*RILPL1*	p.R200W	Missense_Mutation	12	123983294	G	A	100	5.00%	Deleterious	PASSENGER/OTHER	Passenger	passenger	.	.	0.36	0.01271	15%	Subclonal	.	.	.	.	WES		
IDC9T	*RIN2*	p.R430Q	Missense_Mutation	20	19955811	G	A	72	6.94%	Neutral	PASSENGER/OTHER	Passenger	passenger	.	.	0.43	0.04602	19%	Subclonal	.	.	.	.	WES		
IDC9T	*RNF213*	p.E2590K	Missense_Mutation	17	78319903	G	A	197	3.55%	Deleterious	PASSENGER/OTHER	Passenger	passenger	.	.	0.36	0.00362	18%	Subclonal	.	.	.	.	WES		
IDC9T	*RP11-127H5.1*	p.A68V	Missense_Mutation	8	105845627	C	T	36	16.67%		PASSENGER/OTHER	Passenger	passenger	.	.	1	0.58126	45%	Clonal	.	.	.	.	WES		
IDC9T	*RPGRIP1L*	p.R1177Q	Missense_Mutation	16	53653023	C	T	76	6.58%	Deleterious	PASSENGER/OTHER	Passenger	passenger	.	.	0.41	0.03213	18%	Subclonal	.	.	.	.	WES		
IDC9T	*RYR2*	p.T3631A	Missense_Mutation	1	237893612	A	G	143	12.59%	Deleterious	PASSENGER/OTHER	Passenger	passenger	.	.	1	0.68427	67%	Clonal	.	.	.	.	WES		
IDC9T	*SASH1*	p.D1107N	Missense_Mutation	6	148865925	G	A	28	14.29%	Deleterious	PASSENGER/OTHER	Passenger	passenger	.	loh	0.61	0.21192	23%	Subclonal	.	.	.	.	WES		
IDC9T	*SH3BP5*	p.E259K	Missense_Mutation	3	15300452	C	T	77	6.49%	Deleterious	PASSENGER/OTHER	Passenger	passenger	.	loh	0.28	0.00034	12%	Subclonal	.	.	.	.	WES		
IDC9T	*SLC22A8*	p.V448I	Missense_Mutation	11	62761083	C	T	46	8.70%	Neutral	PASSENGER/OTHER	Passenger	passenger	.	.	0.46	0.08462	18%	Subclonal	.	.	.	.	WES		
IDC9T	*SLC39A1*	p.R202Q	Missense_Mutation	1	153932944	C	T	138	4.35%	Neutral	PASSENGER/OTHER	Passenger	passenger	.	.	0.31	0.00114	14%	Subclonal	.	.	.	.	WES		
IDC9T	*SLC39A14*	p.E138K	Missense_Mutation	8	22265964	G	A	26	15.38%	Neutral	PASSENGER/OTHER	Passenger	passenger	.	loh	0.65	0.24956	25%	Subclonal	.	.	.	.	WES		
IDC9T	*SLC3A1*	p.R86W	Missense_Mutation	2	44502930	C	T	80	6.25%	Deleterious	PASSENGER/OTHER	Passenger	passenger	.	.	0.39	0.02201	17%	Subclonal	.	.	.	.	WES		
IDC9T	*SLC9C1*	p.E472D	Missense_Mutation	3	111958717	C	A	77	20.78%	Neutral	PASSENGER/OTHER	Passenger	passenger	.	.	1	0.6818	65%	Clonal	.	.	.	.	WES		
IDC9T	*SORBS2*	p.P1145L	Missense_Mutation	4	186515040	G	A	209	3.83%	Deleterious	PASSENGER/OTHER	Passenger	passenger	.	.	0.2	0	10%	Subclonal	.	.	.	.	WES		
IDC9T	*SRRM3*	p.G8R	Missense_Mutation	7	75864406	G	A	93	5.38%	Deleterious	.	Passenger	passenger	.	.	0.34	0.00576	14%	Subclonal	.	.	.	.	WES		
IDC9T	*SSUH2*	p.R115C	Missense_Mutation	3	8673792	G	A	100	5.00%	Deleterious	PASSENGER/OTHER	Passenger	passenger	.	loh	0.21	0	9%	Subclonal	.	.	.	.	WES		
IDC9T	*STAG3*	p.I864T	Missense_Mutation	7	99800104	T	C	117	7.69%	Deleterious	PASSENGER/OTHER	Passenger	passenger	.	.	0.4	0.00242	21%	Subclonal	.	.	.	.	WES		
IDC9T	*TAF1L*	p.R1416T	Missense_Mutation	9	32631331	C	G	146	42.47%	Deleterious	PASSENGER/OTHER	Passenger	passenger	.	.	1	0.88124	92%	Clonal	.	.	.	.	WES		
IDC9T	*TAF5*	p.G361R	Missense_Mutation	10	105138275	G	A	48	8.33%	Deleterious	PASSENGER/OTHER	Passenger	passenger	.	loh	0.35	0.01667	14%	Subclonal	.	.	.	.	WES		
IDC9T	*TBC1D22B*	p.R386Q	Missense_Mutation	6	37281659	G	A	47	8.51%	Deleterious	PASSENGER/OTHER	Passenger	passenger	.	.	0.53	0.15394	21%	Subclonal	.	.	.	.	WES		
IDC9T	*TEX13A*	p.E271K	Missense_Mutation	X	104464065	C	T	94	5.32%	Neutral	.	Passenger	passenger	.	.	0.23	0.00001	9%	Subclonal	.	.	.	.	WES		
IDC9T	*THBD*	p.S492T	Missense_Mutation	20	23028668	A	T	103	33.01%	Deleterious	PASSENGER/OTHER	Passenger	passenger	.	.	1	0.90383	85%	Clonal	.	.	.	.	WES		
IDC9T	*TLE2*	p.S436L	Missense_Mutation	19	3006611	G	A	67	7.46%	Deleterious	PASSENGER/OTHER	Passenger	passenger	.	loh	0.32	0.00216	13%	Subclonal	.	.	.	.	WES		
IDC9T	*TLR1*	p.D523H	Missense_Mutation	4	38798886	C	G	17	23.53%	Neutral	PASSENGER/OTHER	Passenger	passenger	.	.	1	0.50344	36%	Clonal	.	.	.	.	WES		
IDC9T	*TMEM130*	p.R122K	Missense_Mutation	7	98460744	C	T	47	8.51%	Neutral	PASSENGER/OTHER	Passenger	passenger	.	.	0.45	0.07669	18%	Subclonal	.	.	.	.	WES		
IDC9T	*TMPRSS11B*	p.R287H	Missense_Mutation	4	69095061	C	T	232	3.88%	Neutral	PASSENGER/OTHER	Passenger	passenger	.	.	0.44	0.01012	23%	Subclonal	.	.	.	.	WES		
IDC9T	*TNNI1*	p.P184L	Missense_Mutation	1	201379469	G	A	92	6.52%	Deleterious	PASSENGER/OTHER	Passenger	passenger	.	.	0.47	0.05675	22%	Subclonal	.	.	.	.	WES		
IDC9T	*TNS1*	p.T1105M	Missense_Mutation	2	218683429	G	A	26	15.38%	Deleterious	CANCER	Passenger	likely_pathogenic	.	.	0.81	0.34829	29%	Subclonal	.	.	.	.	WES		
IDC9T	*TRA2A*	p.R248K	Missense_Mutation	7	23545784	C	T	30	13.33%	Deleterious	PASSENGER/OTHER	Passenger	passenger	.	.	0.83	0.3602	29%	Subclonal	.	.	.	.	WES		
IDC9T	*TSSK1B*	p.T299I	Missense_Mutation	5	112769641	G	A	96	6.25%	Neutral	PASSENGER/OTHER	Passenger	passenger	.	.	0.39	0.01193	18%	Subclonal	.	.	.	.	WES		
IDC9T	*TTN*	p.E10199K	Missense_Mutation	2	179566258	C	T	141	9.93%	Deleterious	.	Passenger	passenger	.	.	0.52	0.01146	31%	Subclonal	.	.	.	.	WES		
IDC9T	*UAP1L1*	p.R486Q	Missense_Mutation	9	139977114	G	A	95	5.26%	Neutral	PASSENGER/OTHER	Passenger	passenger	.	.	0.28	0.00047	12%	Subclonal	.	.	.	.	WES		
IDC9T	*UBR2*	p.W157R	Missense_Mutation	6	42561980	T	A	101	8.91%	Deleterious	PASSENGER/OTHER	Driver	likely_pathogenic	.	.	0.56	0.07835	29%	Subclonal	.	.	.	.	WES		
IDC9T	*UTS2R*	p.R81H	Missense_Mutation	17	80332442	G	A	85	5.88%	Deleterious	PASSENGER/OTHER	Passenger	passenger	.	.	0.37	0.01339	16%	Subclonal	.	.	.	.	WES		
IDC9T	*VLDLR*	p.R634C	Missense_Mutation	9	2648285	C	T	118	5.93%	Deleterious	PASSENGER/OTHER	Passenger	passenger	.	.	0.31	0.00023	15%	Subclonal	.	.	.	.	WES		
IDC9T	*VPS51*	p.R138Q	Missense_Mutation	11	64875107	G	A	86	5.81%	Deleterious	.	Passenger	passenger	.	.	0.3	0.00173	13%	Subclonal	.	.	.	.	WES		
IDC9T	*WDR87*	p.R2701C	Missense_Mutation	19	38376093	G	A	145	4.14%	Neutral	PASSENGER/OTHER	Passenger	passenger	.	.	0.26	0.00004	12%	Subclonal	.	.	.	.	WES		
IDC9T	*WNT11*	p.T68M	Missense_Mutation	11	75907643	G	A	89	5.62%	Deleterious	PASSENGER/OTHER	Passenger	passenger	.	.	0.29	0.00113	13%	Subclonal	.	.	.	.	WES		
IDC9T	*XPNPEP3*	p.R160Q	Missense_Mutation	22	41278071	G	A	83	6.02%	Deleterious	PASSENGER/OTHER	Passenger	passenger	.	loh	0.26	0.00011	11%	Subclonal	.	.	.	.	WES		
IDC9T	*YTHDF2*	p.S143C	Missense_Mutation	1	29069210	C	G	94	7.45%	Deleterious	PASSENGER/OTHER	Passenger	passenger	.	loh	0.39	0.00565	19%	Subclonal	.	.	.	.	WES		
IDC9T	*ZMYM1*	p.R1031Q	Missense_Mutation	1	35580523	G	A	184	3.80%	Neutral	PASSENGER/OTHER	Passenger	passenger	.	loh	0.2	0	9%	Subclonal	.	.	.	.	WES		
IDC9T	*ZNF354B*	p.G416E	Missense_Mutation	5	178310700	G	A	67	19.40%	Deleterious	PASSENGER/OTHER	Passenger	passenger	.	.	1	0.63038	60%	Clonal	.	.	.	.	WES		
IDC9T	*ZNF366*	p.V558A	Missense_Mutation	5	71743096	A	G	113	19.47%	Deleterious	PASSENGER/OTHER	Passenger	passenger	.	loh	0.83	0.3688	55%	Subclonal	.	.	.	.	WES		
IDC9T	*ZNF425*	p.P557H	Missense_Mutation	7	148801293	G	T	164	4.88%	Neutral	PASSENGER/OTHER	Passenger	passenger	.	.	0.35	0.00092	18%	Subclonal	.	.	.	.	WES		
IDC9T	*ZNF557*	p.P263L	Missense_Mutation	19	7083250	C	T	71	18.31%	Deleterious	PASSENGER/OTHER	Passenger	passenger	.	loh	0.78	0.31684	45%	Subclonal	.	.	.	.	WES		
IDC9T	*ZNF677*	p.D48N	Missense_Mutation	19	53747024	C	T	38	10.53%	Neutral	PASSENGER/OTHER	Passenger	passenger	.	.	0.66	0.25644	25%	Subclonal	.	.	.	.	WES		
IDC9T	*ZNF703*	p.S585W	Missense_Mutation	8	37556173	C	G	181	8.29%	Deleterious	PASSENGER/OTHER	Passenger	passenger	.	.	0.68	0.17015	41%	Subclonal	.	.	.	.	WES		
IDC9T	*ABCC4*	p.R998*	Nonsense_Mutation	13	95725484	G	A	172	4.07%	Deleterious	.	.	passenger	.	.	0.21	0	10%	Subclonal	.	.	.	.	WES		
IDC9T	*BCHE*	p.R70*	Nonsense_Mutation	3	165548614	G	A	136	4.41%	Deleterious	.	.	passenger	.	.	0.28	0.00013	12%	Subclonal	.	.	.	.	WES		
IDC9T	*CDC25C*	p.R448*	Nonsense_Mutation	5	137621461	G	A	119	5.88%	Deleterious	.	.	passenger	.	.	0.37	0.00308	18%	Subclonal	.	.	.	.	WES		
IDC9T	*PBRM1*	p.R58*	Nonsense_Mutation	3	52712580	G	A	53	9.43%	Deleterious	.	.	likely_pathogenic	.	loh	0.4	0.0217	17%	Subclonal	TRUE	TRUE	TRUE	YES	WES		
IDC9T	*PLB1*	p.R422*	Nonsense_Mutation	2	28788204	C	T	39	10.26%	Deleterious	.	.	passenger	.	.	0.64	0.24405	24%	Subclonal	.	.	.	.	WES		
IDC9T	*RBM5*	p.R702*	Nonsense_Mutation	3	50154516	C	T	101	6.93%	Deleterious	.	.	likely_pathogenic	.	loh	0.29	0.00006	14%	Subclonal	.	.	.	.	WES		
IDC9T	*SCUBE2*	p.R609*	Nonsense_Mutation	11	9069080	G	A	35	11.43%	Deleterious	.	.	likely_pathogenic	.	loh	0.48	0.10401	19%	Subclonal	.	.	.	.	WES		
IDC9T	*ZFHX4*	p.R2933*	Nonsense_Mutation	8	77767954	C	T	222	4.05%	Deleterious	.	.	passenger	.	.	0.33	0.00017	17%	Subclonal	.	.	.	.	WES		
IDC9T	*ZNF512B*	p.R813*	Nonsense_Mutation	20	62591483	G	A	138	5.80%	Deleterious	.	.	passenger	.	.	0.42	0.00896	21%	Subclonal	.	.	.	.	WES		
IDC9T	*ZNFX1*	p.W590*	Nonsense_Mutation	20	47886580	C	T	84	30.95%	Deleterious	.	.	passenger	.	.	1	0.85856	82%	Clonal	.	.	.	.	WES		
IDC9T	*NIN*	p.X271_splice	Splice_Site	14	51239666	C	T	46	10.87%	Deleterious	.	.	likely_pathogenic	.	.	0.68	0.26294	28%	Subclonal	TRUE	.	.	.	WES		
IDC9T	*SUSD3*	p.X93_splice	Splice_Site	9	95838255	G	A	37	10.81%	Deleterious	.	.	passenger	.	.	0.57	0.18232	22%	Subclonal	.	.	.	.	WES		

Supplementary Table 4. Clinicopathologic characteristics of the *PALB2*- associated breast cancers according to bi-allelic inactivation of the *PALB2* wild-type allele.

	All cases		Bi-allelic *PALB2* alterations		Mono-allelic *PALB2* alterations		*P* value
	No.	Percentage	No.	Percentage	No.	Percentage	
**Study**							
Li et al.	24	62%	16	62%	8	62%	
Lee et al.	15	38%	10	38%	5	38%	
**Age (years)**							
<50	21	54%	11	42%	10	77%	0.051
≥50	18	46%	15	58%	3	23%	
**Receptor status**							
ER+/HER2-	21	54%	16	62%	5	38%	0.383
ER+/HER2+	5	13%	3	12%	2	15%	
ER-/HER2-	13	33%	7	27%	6	46%	
**Histologic grade**							
I/II	13	33%	8	31%	5	38%	0.725
III	26	67%	18	69%	8	62%	
**Germline mutation type**							
Frameshift	22	56%	12	46%	10	77%	0.175
Truncating	16	41%	13	50%	3	23%	
Missense	1	3%	1	4%	0	0%	
***TP53* gene**							
Mutant	10	26%	5	19%	5	38%	0.254
Wild-type	29	74%	21	81%	8	62%	
**LSTs*, [1]**							
High	27	87%	22	100	5	62.5	0.004
Low	4	13%	0	0	4	37.5	

Abbreviations: Age, age at diagnosis; ER, estrogen receptor; LST, large-scale state transition; *, LST high, ≥15; LST low, <15; No., number of cases.

[1]LSTs were investigated in 16 *PALB2*- associated breast cancers from this study and 15 *PALB2* breast cancers from Lee et al, J Pathol 2018.

Supplementary Table 5: Comparisons of frequencies of somatic mutations affecting 410 cancer genes between *PALB2*- associated breast cancers and non-*BRCA1/2/PALB2*-, *BRCA1*- and *BRCA2*- associated breast cancers from TCGA.

	ER+/HER2-, ER+/HER2+ and ER-/HER2-						ER+/HER2-						*PALB2* with bi-allelic alterations (n=16)		TCGA *BRCA1* with bi-allelic alterations (n=17)		*PALB2* vs. TCGA *BRCA1*	*PALB2* with bi-allelic alterations (n=16)		TCGA *BRCA2* with bi-allelic alterations (n=16)		*PALB2* vs. TCGA *BRCA2*
	PALB2 (n=24)		TCGA non-*BRCA1/2/PALB2* (n=683)		*PALB2* vs. TCGA non-*BRCA1/2/PALB2*		*PALB2* (n=18)		TCGA non-*BRCA1/2/PALB2* (n=441)		*PALB2* vs. TCGA non-*BRCA1/2/PALB2*											
MSK-IMPACT 410 cancer genes	Number of cases with somatic mutations affecting 410 cancer genes	% of cases with somatic mutations affecting 410 cancer genes	Number of cases with somatic mutations affecting 410 cancer genes	% of cases with somatic mutations affecting 410 cancer genes	Fisher's exact test *P* value	Fisher's exact test *P* value of bootstrapping comparison	Number of cases with somatic mutations affecting 410 cancer genes	% of cases with somatic mutations affecting 410 cancer genes	Number of cases with somatic mutations affecting 410 cancer genes	% of cases with somatic mutations affecting 410 cancer genes	Fisher's exact test *P* value	Fisher's exact test *P* value of bootstrapping comparison	Number of cases with somatic mutations affecting 410 cancer genes	% of cases with somatic mutations affecting 410 cancer genes	Number of cases with somatic mutations affecting 410 cancer genes	% of cases with somatic mutations affecting 410 cancer genes	Fisher's exact test *P* value	Number of cases with somatic mutations affecting 410 cancer genes	% of cases with somatic mutations affecting 410 cancer genes	Number of cases with somatic mutations affecting 410 cancer genes	% of cases with somatic mutations affecting 410 cancer genes	Fisher's exact test *P* value
ABL1	0	0%	4	0.6%	1	1	0	0%	2	0.5%	1	1	0	0%	0	0%	1	0	0%	0	0%	1
ACVR1	0	0%	1	0%	1	1	0	0%	0	0%	1	1	0	0%	0	0%	1	0	0%	0	0%	1
AKT1	0	0%	20	2.9%	1	0.87	0	0%	18	4.1%	1	0.85	0	0%	0	0%	1	0	0%	0	0%	1
AKT2	0	0%	3	0%	1	1	0	0%	3	0.7%	1	1	0	0%	0	0%	1	0	0%	0	0%	1
AKT3	0	0%	4	0.6%	1	0.99	0	0%	3	0.7%	1	1	0	0%	0	0%	1	0	0%	0	0%	1
ALK	0	0%	7	1.0%	1	0.98	0	0%	5	1.1%	1	1	0	0%	0	0%	1	0	0%	1	6.2%	1
ALOX12B	0	0%	2	0%	1	1	0	0%	1	0.2%	1	1	0	0%	0	0%	1	0	0%	0	0%	1
AMER1	0	0%	12	1.8%	1	0.93	0	0%	7	1.6%	1	1	0	0%	0	0%	1	0	0%	0	0%	1
ANKRD11	0	0%	16	2.3%	1	0.94	0	0%	7	1.6%	1	0.99	0	0%	1	5.9%	1	0	0%	0	0%	1
APC	0	0%	9	1.3%	1	0.97	0	0%	4	0.9%	1	1	0	0%	2	11.8%	0.48	0	0%	0	0%	1
AR	0	0%	5	0.7%	1	0.99	0	0%	3	0.7%	1	1	0	0%	1	5.9%	1	0	0%	0	0%	1
ARAF	0	0%	6	0.9%	1	0.99	0	0%	4	0.9%	1	1	0	0%	0	0%	1	0	0%	0	0%	1
ARID1A	3	12.5%	26	3.8%	0.07	0.19	3	16.7%	15	3.4%	0.03	0.11	3	18.8%	0	0%	0.10	3	18.8%	1	6.2%	0.60
ARID1B	0	0%	16	2.3%	1	0.92	0	0%	6	1.4%	1	0.98	0	0%	1	5.9%	1	0	0%	1	6.2%	1
ARID2	1	4.2%	11	1.6%	0.34	0.58	0	0%	7	1.6%	1	0.43	0	0%	0	0%	1	0	0%	0	0%	1
ARID5B	0	0%	3	0%	1	1	0	0%	2	0.5%	1	1	0	0%	0	0%	1	0	0%	2	12.5%	0.48
ASXL1	1	4.2%	7	1.0%	0.24	0.43	0	0%	4	0.9%	1	0.34	0	0%	0	0%	1	0	0%	0	0%	1
ASXL2	1	4.2%	14	2.0%	0.41	0.66	1	5.6%	6	1.4%	0.25	0.40	0	0%	2	11.8%	0.48	0	0%	0	0%	1
ATM	0	0%	20	2.9%	1	0.89	0	0%	7	1.6%	1	1	0	0%	0	0%	1	0	0%	0	0%	1
ATR	0	0%	8	1.2%	1	0.98	0	0%	5	1.1%	1	1	0	0%	0	0%	1	0	0%	0	0%	1
ATRX	0	0%	21	3.1%	1	0.84	0	0%	13	2.9%	1	0.89	0	0%	1	5.9%	1	0	0%	0	0%	1
AURKA	0	0%	4	0.6%	1	1	0	0%	2	0.5%	1	1	0	0%	0	0%	1	0	0%	0	0%	1
AURKB	0	0%	2	0%	1	1	0	0%	1	0.2%	1	1	0	0%	0	0%	1	0	0%	0	0%	1
AXIN1	0	0%	4	0.6%	1	1	0	0%	3	0.7%	1	1	0	0%	0	0%	1	0	0%	1	6.2%	1
AXIN2	0	0%	5	0.7%	1	1	0	0%	2	0.5%	1	1	0	0%	1	5.9%	1	0	0%	0	0%	1
AXL	0	0%	2	0%	1	1	0	0%	1	0.2%	1	1	0	0%	2	11.8%	0.48	0	0%	0	0%	1
B2M	0	0%	2	0%	1	1	0	0%	1	0.2%	1	1	0	0%	0	0%	1	0	0%	0	0%	1
BAP1	0	0%	7	1.0%	1	0.99	0	0%	3	0.7%	1	1	0	0%	0	0%	1	0	0%	0	0%	1
BARD1	0	0%	0	0%	1	1	0	0%	0	0%	1	1	0	0%	0	0%	1	0	0%	0	0%	1
BBC3	0	0%	0	0%	1	1	0	0%	0	0%	1	1	0	0%	0	0%	1	0	0%	0	0%	1
BCL10	0	0%	1	0%	1	1	0	0%	1	0.2%	1	1	0	0%	1	5.9%	1	0	0%	0	0%	1
BCL2	0	0%	0	0%	1	1	0	0%	0	0%	1	1	0	0%	0	0%	1	0	0%	0	0%	1
BCL2L1	0	0%	3	0%	1	1	0	0%	1	0.2%	1	1	0	0%	0	0%	1	0	0%	0	0%	1
BCL2L11	0	0%	5	0.7%	1	1	0	0%	3	0.7%	1	1	0	0%	0	0%	1	0	0%	0	0%	1
BCL6	0	0%	4	0.6%	1	1	0	0%	2	0.5%	1	1	0	0%	0	0%	1	0	0%	0	0%	1
BCOR	1	4.2%	10	1.5%	0.32	0.49	1	5.6%	4	0.9%	0.18	0.37	1	6.2%	0	0%	0.48	1	6.2%	1	6.2%	1
BIRC3	0	0%	2	0%	1	1	0	0%	0	0%	1	1	0	0%	0	0%	1	0	0%	0	0%	1
BLM	0	0%	10	1.5%	1	0.96	0	0%	5	1.1%	1	0.99	0	0%	1	5.9%	1	0	0%	0	0%	1
BMPR1A	0	0%	0	0%	1	1	0	0%	0	0%	1	1	0	0%	0	0%	1	0	0%	0	0%	1
BRAF	1	4.2%	6	0.9%	0.22	0.41	1	5.6%	3	0.7%	0.15	0.32	1	6.2%	0	0%	0.48	1	6.2%	0	0%	1
BRCA1	0	0%	19	2.8%	1	0.86	0	0%	10	2.3%	1	0.93	0	0%	0	0%	1	0	0%	0	0%	1
BRCA2	0	0%	20	2.9%	1	0.88	0	0%	11	2.5%	1	0.92	0	0%	1	5.9%	1	0	0%	2	12.5%	0.48
BRD4	1	4.2%	4	0.6%	0.16	0.37	1	5.6%	1	0.2%	0.08	0.26	1	6.2%	1	5.9%	1	1	6.2%	0	0%	1
BRIP1	2	8.3%	8	1.2%	0.04	0.16	2	11.1%	4	0.9%	0.02	0.10	2	12.5%	0	0%	0.23	2	12.5%	0	0%	0.48
BTK	0	0%	7	1.0%	1	0.99	0	0%	5	1.1%	1	1	0	0%	0	0%	1	0	0%	0	0%	1
CALR	0	0%	1	0%	1	1	0	0%	0	0%	1	1	0	0%	0	0%	1	0	0%	0	0%	1
CARD11	1	4.2%	7	1.0%	0.24	0.40	1	5.6%	1	0.2%	0.08	0.28	1	6.2%	1	5.9%	1	1	6.2%	0	0%	1
CASP8	1	4.2%	12	1.8%	0.36	0.58	0	0%	7	1.6%	1	0.48	1	6.2%	2	11.8%	1	1	6.2%	1	6.2%	1
CBFB	0	0%	21	3.1%	1	0.80	0	0%	17	3.9%	1	0.90	0	0%	0	0%	1	0	0%	1	6.2%	1
CBL	0	0%	4	0.6%	1	0.98	0	0%	2	0.5%	1	1	0	0%	0	0%	1	0	0%	0	0%	1
CCND1	1	4.2%	1	0%	0.07	0.27	1	5.6%	1	0.2%	0.08	0.27	0	0%	0	0%	1	0	0%	0	0%	1
CCND2	0	0%	2	0%	1	1	0	0%	0	0%	1	1	0	0%	0	0%	1	0	0%	0	0%	1
CCND3	0	0%	2	0%	1	1	0	0%	2	0.5%	1	1	0	0%	0	0%	1	0	0%	0	0%	1
CCNE1	0	0%	0	0%	1	1	0	0%	0	0%	1	1	0	0%	0	0%	1	0	0%	0	0%	1
CD274	1	4.2%	2	0%	0.10	0.31	1	5.6%	1	0.2%	0.08	0.27	0	0%	0	0%	1	0	0%	0	0%	1
CD276	0	0%	2	0%	1	1	0	0%	1	0.2%	1	1	0	0%	0	0%	1	0	0%	0	0%	1
CD79A	2	8.3%	1	0%	0.003	0.08	2	11.1%	1	0.2%	0.004	0.07	2	12.5%	0	0%	0.23	2	12.5%	0	0%	0.48
CD79B	0	0%	0	0%	1	1	0	0%	0	0%	1	1	0	0%	0	0%	1	0	0%	0	0%	1
CDC73	0	0%	5	0.7%	1	1	0	0%	4	0.9%	1	1	0	0%	0	0%	1	0	0%	0	0%	1
CDH1	1	4.2%	93	13.6%	0.23	0.41	1	5.6%	80	18.1%	0.22	0.39	1	6.2%	0	0%	0.48	1	6.2%	1	6.2%	1
CDK12	1	4.2%	9	1.3%	0.29	0.49	1	5.6%	5	1.1%	0.21	0.40	1	6.2%	0	0%	0.48	1	6.2%	1	6.2%	1
CDK4	0	0%	1	0%	1	1	0	0%	0	0%	1	1	0	0%	0	0%	1	0	0%	0	0%	1
CDK6	0	0%	3	0%	1	1	0	0%	2	0.5%	1	1	0	0%	0	0%	1	0	0%	0	0%	1
CDK8	0	0%	1	0%	1	1	0	0%	1	0.2%	1	1	0	0%	0	0%	1	0	0%	0	0%	1
CDKN1A	0	0%	1	0%	1	1	0	0%	0	0%	1	1	0	0%	0	0%	1	0	0%	0	0%	1
CDKN1B	0	0%	8	1.2%	1	0.97	0	0%	6	1.4%	1	1	0	0%	0	0%	1	0	0%	0	0%	1
CDKN2A	0	0%	0	0%	1	1	0	0%	0	0%	1	1	0	0%	0	0%	1	0	0%	0	0%	1
CDKN2B	0	0%	0	0%	1	1	0	0%	0	0%	1	1	0	0%	0	0%	1	0	0%	0	0%	1
CDKN2C	0	0%	0	0%	1	1	0	0%	0	0%	1	1	0	0%	0	0%	1	0	0%	0	0%	1
CEBPA	0	0%	0	0%	1	1	0	0%	0	0%	1	1	0	0%	0	0%	1	0	0%	0	0%	1
CENPA	0	0%	0	0%	1	1	0	0%	0	0%	1	1	0	0%	0	0%	1	0	0%	0	0%	1
CHEK1	0	0%	2	0%	1	1	0	0%	1	0.2%	1	1	0	0%	0	0%	1	0	0%	0	0%	1
CHEK2	1	4.2%	6	0.9%	0.22	0.41	1	5.6%	4	0.9%	0.18	0.37	0	0%	0	0%	1	0	0%	0	0%	1
CIC	1	4.2%	3	0%	0.13	0.34	1	5.6%	1	0.2%	0.08	0.27	1	6.2%	1	5.9%	1	1	6.2%	0	0%	1
CREBBP	0	0%	17	2.5%	1	0.86	0	0%	9	2.0%	1	0.96	0	0%	2	11.8%	0.48	0	0%	0	0%	1
CRKL	0	0%	0	0%	1	1	0	0%	0	0%	1	1	0	0%	0	0%	1	0	0%	0	0%	1
CRLF2	0	0%	0	0%	1	1	0	0%	0	0%	1	1	0	0%	0	0%	1	0	0%	0	0%	1
CSF1R	0	0%	2	0%	1	1	0	0%	1	0.2%	1	1	0	0%	0	0%	1	0	0%	0	0%	1
CSF3R	0	0%	4	0.6%	1	1	0	0%	2	0.5%	1	1	0	0%	1	5.9%	1	0	0%	0	0%	1
CTCF	0	0%	11	1.6%	1	0.97	0	0%	8	1.8%	1	0.97	0	0%	0	0%	1	0	0%	0	0%	1
CTLA4	0	0%	1	0%	1	1	0	0%	1	0.2%	1	1	0	0%	0	0%	1	0	0%	0	0%	1
CTNNB1	0	0%	1	0%	1	1	0	0%	0	0%	1	1	0	0%	0	0%	1	0	0%	0	0%	1
CUL3	0	0%	4	0.6%	1	1	0	0%	2	0.5%	1	1	0	0%	0	0%	1	0	0%	0	0%	1
CXCR4	0	0%	3	0%	1	1	0	0%	3	0.7%	1	1	0	0%	1	5.9%	1	0	0%	0	0%	1
DAXX	1	4.2%	2	0%	0.10	0.30	0	0%	2	0.5%	1	0.30	0	0%	0	0%	1	0	0%	0	0%	1
DCUN1D1	0	0%	4	0.6%	1	1	0	0%	1	0.2%	1	1	0	0%	0	0%	1	0	0%	0	0%	1
DDR2	0	0%	7	1.0%	1	0.98	0	0%	5	1.1%	1	0.99	0	0%	1	5.9%	1	0	0%	0	0%	1
DICER1	1	4.2%	10	1.5%	0.32	0.51	1	5.6%	8	1.8%	0.30	0.49	1	6.2%	1	5.9%	1	1	6.2%	0	0%	1
DIS3	0	0%	4	0.6%	1	1	0	0%	3	0.7%	1	1	0	0%	0	0%	1	0	0%	1	6.2%	1
DNAJB1	0	0%	3	0%	1	1	0	0%	1	0.2%	1	1	0	0%	0	0%	1	0	0%	0	0%	1
DNMT1	0	0%	10	1.5%	1	0.97	0	0%	4	0.9%	1	1	0	0%	2	11.8%	0.48	0	0%	0	0%	1
DNMT3A	2	8.3%	6	0.9%	0.03	0.13	2	11.1%	4	0.9%	0.02	0.10	1	6.2%	1	5.9%	1	1	6.2%	0	0%	1
DNMT3B	0	0%	4	0.6%	1	1	0	0%	3	0.7%	1	1	0	0%	0	0%	1	0	0%	0	0%	1
DOT1L	1	4.2%	7	1.0%	0.24	0.45	1	5.6%	5	1.1%	0.21	0.40	1	6.2%	0	0%	0.48	1	6.2%	0	0%	1
E2F3	0	0%	4	0.6%	1	1	0	0%	2	0.5%	1	1	0	0%	0	0%	1	0	0%	0	0%	1
EED	0	0%	2	0%	1	1	0	0%	1	0.2%	1	1	0	0%	0	0%	1	0	0%	0	0%	1
EGFL7	0	0%	1	0%	1	1	0	0%	0	0%	1	1	0	0%	0	0%	1	0	0%	1	6.2%	1
EGFR	1	4.2%	12	1.8%	0.36	0.62	1	5.6%	6	1.4%	0.25	0.41	1	6.2%	0	0%	0.48	1	6.2%	0	0%	1
EIF1AX	0	0%	3	0%	1	1	0	0%	3	0.7%	1	1	0	0%	0	0%	1	0	0%	0	0%	1
EIF4A2	0	0%	11	1.6%	1	0.95	0	0%	5	1.1%	1	1	0	0%	0	0%	1	0	0%	0	0%	1
EIF4E	0	0%	1	0%	1	1	0	0%	1	0.2%	1	1	0	0%	0	0%	1	0	0%	0	0%	1
EP300	0	0%	9	1.3%	1	0.99	0	0%	5	1.1%	1	1	0	0%	1	5.9%	1	0	0%	0	0%	1
EPCAM	0	0%	0	0%	1	1	0	0%	0	0%	1	1	0	0%	0	0%	1	0	0%	0	0%	1
EPHA3	1	4.2%	7	1.0%	0.24	0.43	1	5.6%	4	0.9%	0.18	0.38	1	6.2%	1	5.9%	1	1	6.2%	0	0%	1
EPHA5	0	0%	8	1.2%	1	0.99	0	0%	6	1.4%	1	0.99	0	0%	1	5.9%	1	0	0%	0	0%	1
EPHA7	1	4.2%	6	0.9%	0.22	0.42	1	5.6%	3	0.7%	0.15	0.33	1	6.2%	1	5.9%	1	1	6.2%	0	0%	1
EPHB1	0	0%	7	1.0%	1	1	0	0%	5	1.1%	1	0.99	0	0%	1	5.9%	1	0	0%	0	0%	1
ERBB2	0	0%	20	2.9%	1	0.85	0	0%	11	2.5%	1	0.93	0	0%	0	0%	1	0	0%	1	6.2%	1
ERBB3	0	0%	17	2.5%	1	0.89	0	0%	7	1.6%	1	0.99	0	0%	1	5.9%	1	0	0%	0	0%	1
ERBB4	0	0%	10	1.5%	1	0.94	0	0%	5	1.1%	1	0.99	0	0%	1	5.9%	1	0	0%	0	0%	1
ERCC2	0	0%	2	0%	1	1	0	0%	1	0.2%	1	1	0	0%	0	0%	1	0	0%	0	0%	1
ERCC3	0	0%	3	0%	1	1	0	0%	2	0.5%	1	1	0	0%	0	0%	1	0	0%	0	0%	1
ERCC4	0	0%	5	0.7%	1	1	0	0%	3	0.7%	1	1	0	0%	0	0%	1	0	0%	0	0%	1
ERCC5	0	0%	0	0%	1	1	0	0%	0	0%	1	1	0	0%	0	0%	1	0	0%	0	0%	1
ERG	0	0%	7	1.0%	1	0.99	0	0%	3	0.7%	1	1	0	0%	0	0%	1	0	0%	0	0%	1
ERRFI1	0	0%	1	0%	1	1	0	0%	1	0.2%	1	1	0	0%	0	0%	1	0	0%	0	0%	1
ESR1	0	0%	6	0.9%	1	1	0	0%	3	0.7%	1	1	0	0%	0	0%	1	0	0%	0	0%	1
ETV1	1	4.2%	3	0%	0.13	0.33	1	5.6%	3	0.7%	0.15	0.34	1	6.2%	1	5.9%	1	1	6.2%	0	0%	1
ETV6	0	0%	5	0.7%	1	1	0	0%	1	0.2%	1	1	0	0%	0	0%	1	0	0%	1	6.2%	1
EZH2	0	0%	2	0%	1	1	0	0%	0	0%	1	1	0	0%	0	0%	1	0	0%	0	0%	1
FAM175A	0	0%	2	0%	1	1	0	0%	2	0.5%	1	1	0	0%	0	0%	1	0	0%	0	0%	1
FAM46C	0	0%	3	0%	1	1	0	0%	1	0.2%	1	1	0	0%	0	0%	1	0	0%	0	0%	1
FANCA	0	0%	10	1.5%	1	0.95	0	0%	5	1.1%	1	1	0	0%	0	0%	1	0	0%	0	0%	1
FANCC	0	0%	3	0%	1	1	0	0%	3	0.7%	1	1	0	0%	0	0%	1	0	0%	0	0%	1
FAT1	0	0%	23	3.4%	1	0.75	0	0%	10	2.3%	1	0.93	0	0%	2	11.8%	0.48	0	0%	0	0%	1

FBXW7	0	0%	14	2.0%	1	0.90	0	0%	6	1.4%	1	0.99	0	0%	2	11.8%	0.48	0	0%	0	0%	1
FGF19	0	0%	0	0%	1	1	0	0%	0	0%	1	1	0	0%	0	0%	1	0	0%	0	0%	1
FGF3	1	4.2%	1	0%	0.07	0.27	1	5.6%	1	0.2%	0.08	0.27	1	6.2%	0	0%	0.48	1	6.2%	0	0%	1
FGF4	0	0%	0	0%	1	1	0	0%	0	0%	1	1	0	0%	0	0%	1	0	0%	0	0%	1
FGFR1	1	4.2%	5	0.7%	0.19	0.38	1	5.6%	5	1.1%	0.21	0.39	1	6.2%	1	5.9%	1	1	6.2%	0	0%	1
FGFR2	0	0%	12	1.8%	1	0.96	0	0%	8	1.8%	1	0.97	0	0%	1	5.9%	1	0	0%	0	0%	1
FGFR3	0	0%	1	0%	1	1	0	0%	1	0.2%	1	1	0	0%	0	0%	1	0	0%	0	0%	1
FGFR4	2	8.3%	4	0.6%	0.02	0.11	2	11.1%	2	0.5%	0.008	0.08	2	12.5%	0	0%	0.23	2	12.5%	0	0%	0.48
FH	0	0%	3	0%	1	1	0	0%	1	0.2%	1	1	0	0%	0	0%	1	0	0%	0	0%	1
FLCN	0	0%	1	0%	1	1	0	0%	1	0.2%	1	1	0	0%	0	0%	1	0	0%	0	0%	1
FLT1	0	0%	6	0.9%	1	1	0	0%	0	0%	1	1	0	0%	1	5.9%	1	0	0%	0	0%	1
FLT3	0	0%	5	0.7%	1	1	0	0%	3	0.7%	1	1	0	0%	0	0%	1	0	0%	0	0%	1
FLT4	0	0%	1	0%	1	1	0	0%	0	0%	1	1	0	0%	0	0%	1	0	0%	0	0%	1
FOXA1	0	0%	22	3.2%	1	0.86	0	0%	18	4.1%	1	0.83	0	0%	0	0%	1	0	0%	0	0%	1
FOXL2	0	0%	0	0%	1	1	0	0%	0	0%	1	1	0	0%	0	0%	1	0	0%	0	0%	1
FOXO1	0	0%	1	0%	1	1	0	0%	1	0.2%	1	1	0	0%	0	0%	1	0	0%	0	0%	1
FOXP1	0	0%	4	0.6%	1	1	0	0%	2	0.5%	1	1	0	0%	0	0%	1	0	0%	0	0%	1
FUBP1	1	4.2%	4	0.6%	0.16	0.35	0	0%	2	0.5%	1	0.31	0	0%	0	0%	1	0	0%	0	0%	1
FYN	0	0%	2	0%	1	1	0	0%	2	0.5%	1	1	0	0%	0	0%	1	0	0%	0	0%	1
GATA1	0	0%	1	0%	1	1	0	0%	1	0.2%	1	1	0	0%	0	0%	1	0	0%	0	0%	1
GATA2	0	0%	2	0%	1	1	0	0%	1	0.2%	1	1	0	0%	0	0%	1	0	0%	0	0%	1
GATA3	0	0%	89	13.0%	0.06	0.15	0	0%	70	15.9%	0.09	0.22	0	0%	0	0%	1	0	0%	2	12.5%	0.48
GLI1	1	4.2%	4	0.6%	0.16	0.36	1	5.6%	3	0.7%	0.15	0.34	1	6.2%	0	0%	0.48	1	6.2%	0	0%	1
GNA11	0	0%	4	0.6%	1	1	0	0%	2	0.5%	1	1	0	0%	0	0%	1	0	0%	0	0%	1
GNAQ	2	8.3%	2	0%	0.006	0.08	2	11.1%	1	0.2%	0.004	0.07	0	0%	0	0%	1	0	0%	0	0%	1
GNAS	0	0%	11	1.6%	1	0.97	0	0%	10	2.3%	1	0.96	0	0%	0	0%	1	0	0%	0	0%	1
GPS2	0	0%	8	1.2%	1	0.99	0	0%	6	1.4%	1	0.99	0	0%	0	0%	1	0	0%	0	0%	1
GREM1	0	0%	0	0%	1	1	0	0%	0	0%	1	1	0	0%	0	0%	1	0	0%	0	0%	1
GRIN2A	0	0%	17	2.5%	1	0.91	0	0%	12	2.7%	1	0.95	0	0%	0	0%	1	0	0%	0	0%	1
GSK3B	0	0%	0	0%	1	1	0	0%	0	0%	1	1	0	0%	0	0%	1	0	0%	0	0%	1
H3F3A	0	0%	2	0%	1	1	0	0%	1	0.2%	1	1	0	0%	0	0%	1	0	0%	0	0%	1
H3F3B	0	0%	2	0%	1	1	0	0%	1	0.2%	1	1	0	0%	0	0%	1	0	0%	0	0%	1
H3F3C	0	0%	1	0%	1	1	0	0%	1	0.2%	1	1	0	0%	0	0%	1	0	0%	0	0%	1
HGF	1	4.2%	4	0.6%	0.16	0.35	0	0%	3	0.7%	1	0.33	0	0%	0	0%	1	0	0%	0	0%	1
HIST1H1C	0	0%	5	0.7%	1	1	0	0%	2	0.5%	1	1	0	0%	0	0%	1	0	0%	0	0%	1
HIST1H2BD	0	0%	2	0%	1	1	0	0%	0	0%	1	1	0	0%	0	0%	1	0	0%	0	0%	1
HIST1H3A	0	0%	1	0%	1	1	0	0%	0	0%	1	1	0	0%	0	0%	1	0	0%	0	0%	1
HIST1H3B	1	4.2%	11	1.6%	0.34	0.56	1	5.6%	5	1.1%	0.21	0.40	1	6.2%	0	0%	0.48	1	6.2%	0	0%	1
HIST1H3C	0	0%	2	0%	1	1	0	0%	0	0%	1	1	0	0%	0	0%	1	0	0%	0	0%	1
HIST1H3D	0	0%	2	0%	1	1	0	0%	0	0%	1	1	0	0%	0	0%	1	0	0%	0	0%	1
HIST1H3E	0	0%	1	0%	1	1	0	0%	0	0%	1	1	0	0%	0	0%	1	0	0%	0	0%	1
HIST1H3F	0	0%	4	0.6%	1	1	0	0%	1	0.2%	1	1	0	0%	0	0%	1	0	0%	0	0%	1
HIST1H3G	0	0%	4	0.6%	1	1	0	0%	3	0.7%	1	1	0	0%	0	0%	1	0	0%	0	0%	1
HIST1H3H	0	0%	0	0%	1	1	0	0%	0	0%	1	1	0	0%	0	0%	1	0	0%	0	0%	1
HIST1H3I	1	4.2%	1	0%	0.07	0.27	0	0%	0	0%	1	0.25	1	6.2%	0	0%	0.48	1	6.2%	0	0%	1
HIST1H3J	1	4.2%	1	0%	0.07	0.27	0	0%	1	0.2%	1	0.27	1	6.2%	0	0%	0.48	1	6.2%	0	0%	1
HIST2H3C	0	0%	0	0%	1	1	0	0%	0	0%	1	1	0	0%	0	0%	1	0	0%	0	0%	1
HIST2H3D	1	4.2%	3	0%	0.13	0.33	1	5.6%	2	0.5%	0.11	0.30	1	6.2%	1	5.9%	1	1	6.2%	0	0%	1
HIST3H3	0	0%	3	0%	1	1	0	0%	3	0.7%	1	1	0	0%	0	0%	1	0	0%	0	0%	1
HNF1A	0	0%	3	0%	1	1	0	0%	1	0.2%	1	1	0	0%	0	0%	1	0	0%	0	0%	1
HOXB13	0	0%	2	0%	1	1	0	0%	0	0%	1	1	0	0%	0	0%	1	0	0%	0	0%	1
HRAS	0	0%	2	0%	1	1	0	0%	1	0.2%	1	1	0	0%	0	0%	1	0	0%	0	0%	1
ICOSLG	0	0%	2	0%	1	1	0	0%	2	0.5%	1	1	0	0%	0	0%	1	0	0%	0	0%	1
ID3	0	0%	0	0%	1	1	0	0%	0	0%	1	1	0	0%	0	0%	1	0	0%	0	0%	1
IDH1	0	0%	2	0%	1	1	0	0%	0	0%	1	1	0	0%	0	0%	1	0	0%	1	6.2%	1
IDH2	0	0%	0	0%	1	1	0	0%	0	0%	1	1	0	0%	0	0%	1	0	0%	0	0%	1
IFNGR1	0	0%	2	0%	1	1	0	0%	1	0.2%	1	1	0	0%	0	0%	1	0	0%	0	0%	1
IGF1	0	0%	1	0%	1	1	0	0%	1	0.2%	1	1	0	0%	0	0%	1	0	0%	0	0%	1
IGF1R	0	0%	8	1.2%	1	0.99	0	0%	5	1.1%	1	1	0	0%	0	0%	1	0	0%	0	0%	1
IGF2	0	0%	1	0%	1	1	0	0%	0	0%	1	1	0	0%	0	0%	1	0	0%	0	0%	1
IKBKE	1	4.2%	3	0%	0.13	0.30	1	5.6%	1	0.2%	0.08	0.28	1	6.2%	0	0%	0.48	1	6.2%	1	6.2%	1
IKZF1	1	4.2%	3	0%	0.13	0.34	1	5.6%	1	0.2%	0.08	0.27	1	6.2%	1	5.9%	1	1	6.2%	0	0%	1
IL10	0	0%	0	0%	1	1	0	0%	0	0%	1	1	0	0%	0	0%	1	0	0%	0	0%	1
IL7R	0	0%	2	0%	1	1	0	0%	1	0.2%	1	1	0	0%	0	0%	1	0	0%	0	0%	1
INHA	0	0%	3	0%	1	1	0	0%	2	0.5%	1	1	0	0%	0	0%	1	0	0%	0	0%	1
INHBA	0	0%	2	0%	1	1	0	0%	1	0.2%	1	1	0	0%	0	0%	1	0	0%	0	0%	1
INPP4A	0	0%	6	0.9%	1	1	0	0%	3	0.7%	1	1	0	0%	0	0%	1	0	0%	0	0%	1
INPP4B	1	4.2%	6	0.9%	0.22	0.43	1	5.6%	6	1.4%	0.25	0.41	1	6.2%	1	5.9%	1	1	6.2%	0	0%	1
INSR	1	4.2%	10	1.5%	0.32	0.57	1	5.6%	5	1.1%	0.21	0.40	0	0%	0	0%	1	0	0%	0	0%	1
IRF4	0	0%	1	0%	1	1	0	0%	1	0.2%	1	1	0	0%	0	0%	1	0	0%	0	0%	1
IRS1	0	0%	4	0.6%	1	1	0	0%	2	0.5%	1	1	0	0%	0	0%	1	0	0%	0	0%	1
IRS2	1	4.2%	0	0%	0.03	0.25	1	5.6%	0	0%	0.04	0.25	0	0%	0	0%	1	0	0%	0	0%	1
JAK1	0	0%	10	1.5%	1	0.96	0	0%	8	1.8%	1	0.98	0	0%	0	0%	1	0	0%	0	0%	1
JAK2	0	0%	2	0%	1	1	0	0%	1	0.2%	1	1	0	0%	1	5.9%	1	0	0%	0	0%	1
JAK3	0	0%	10	1.5%	1	0.96	0	0%	6	1.4%	1	0.99	0	0%	1	5.9%	1	0	0%	0	0%	1
JUN	0	0%	3	0%	1	1	0	0%	1	0.2%	1	1	0	0%	0	0%	1	0	0%	0	0%	1
KDM5A	0	0%	8	1.2%	1	0.97	0	0%	4	0.9%	1	1	0	0%	1	5.9%	1	0	0%	0	0%	1
KDM5C	0	0%	7	1.0%	1	1	0	0%	3	0.7%	1	1	0	0%	0	0%	1	0	0%	0	0%	1
KDM6A	0	0%	15	2.2%	1	0.89	0	0%	10	2.3%	1	0.95	0	0%	1	5.9%	1	0	0%	0	0%	1
KDR	0	0%	7	1.0%	1	0.98	0	0%	5	1.1%	1	1	0	0%	0	0%	1	0	0%	0	0%	1
KEAP1	1	4.2%	2	0%	0.10	0.30	0	0%	2	0.5%	1	0.30	0	0%	0	0%	1	0	0%	0	0%	1
KIT	0	0%	7	1.0%	1	0.99	0	0%	5	1.1%	1	1	0	0%	0	0%	1	0	0%	0	0%	1
KLF4	0	0%	4	0.6%	1	1	0	0%	4	0.9%	1	1	0	0%	0	0%	1	0	0%	0	0%	1
KMT2A	3	12.5%	15	2.2%	0.02	0.09	2	11.1%	8	1.8%	0.05	0.05	2	12.5%	1	5.9%	0.60	2	12.5%	1	6.2%	1
KMT2C	1	4.2%	75	11.0%	0.50	0.57	0	0%	57	12.9%	0.15	0.71	1	6.2%	0	0%	0.48	1	6.2%	0	0%	1
KMT2D	2	8.3%	21	3.1%	0.18	0.31	1	5.6%	10	2.3%	0.36	0.20	1	6.2%	0	0%	0.48	1	6.2%	1	6.2%	1
KRAS	0	0%	6	0.9%	1	1	0	0%	5	1.1%	1	0.98	0	0%	0	0%	1	0	0%	0	0%	1
LATS1	1	4.2%	8	1.2%	0.27	0.46	1	5.6%	7	1.6%	0.28	0.48	0	0%	1	5.9%	1	0	0%	0	0%	1
LATS2	0	0%	4	0.6%	1	1	0	0%	3	0.7%	1	1	0	0%	0	0%	1	0	0%	0	0%	1
LMO1	0	0%	0	0%	1	1	0	0%	0	0%	1	1	0	0%	0	0%	1	0	0%	0	0%	1
MALT1	0	0%	2	0%	1	1	0	0%	0	0%	1	1	0	0%	0	0%	1	0	0%	0	0%	1
MAP2K1	0	0%	3	0%	1	1	0	0%	2	0.5%	1	1	0	0%	1	5.9%	1	0	0%	0	0%	1
MAP2K2	0	0%	2	0%	1	1	0	0%	1	0.2%	1	1	0	0%	1	5.9%	1	0	0%	0	0%	1
MAP2K4	0	0%	26	3.8%	1	0.77	0	0%	22	5.0%	1	0.72	0	0%	0	0%	1	0	0%	0	0%	1
MAP3K1	1	4.2%	68	10.0%	0.50	0.64	1	5.6%	54	12.2%	0.71	0.71	1	6.2%	0	0%	0.48	1	6.2%	0	0%	1
MAP3K13	0	0%	10	1.5%	1	0.97	0	0%	8	1.8%	1	0.98	0	0%	0	0%	1	0	0%	1	6.2%	1
MAP3K14	0	0%	0	0%	1	1	0	0%	0	0%	1	1	0	0%	0	0%	1	0	0%	0	0%	1
MAPK1	0	0%	1	0%	1	1	0	0%	1	0.2%	1	1	0	0%	1	5.9%	1	0	0%	0	0%	1
MAPK3	0	0%	0	0%	1	1	0	0%	0	0%	1	1	0	0%	0	0%	1	0	0%	0	0%	1
MAX	0	0%	1	0%	1	1	0	0%	1	0.2%	1	1	0	0%	0	0%	1	0	0%	0	0%	1
MCL1	0	0%	1	0%	1	1	0	0%	1	0.2%	1	1	0	0%	0	0%	1	0	0%	0	0%	1
MDC1	0	0%	4	0.6%	1	1	0	0%	1	0.2%	1	1	0	0%	0	0%	1	0	0%	0	0%	1
MDM2	0	0%	2	0%	1	1	0	0%	1	0.2%	1	1	0	0%	0	0%	1	0	0%	0	0%	1
MDM4	0	0%	4	0.6%	1	1	0	0%	1	0.2%	1	1	0	0%	0	0%	1	0	0%	0	0%	1
MED12	0	0%	22	3.2%	1	0.79	0	0%	11	2.5%	1	0.95	0	0%	1	5.9%	1	0	0%	0	0%	1
MEF2B	0	0%	2	0%	1	1	0	0%	2	0.5%	1	1	0	0%	0	0%	1	0	0%	0	0%	1
MEN1	0	0%	6	0.9%	1	0.99	0	0%	4	0.9%	1	1	0	0%	0	0%	1	0	0%	0	0%	1
MET	0	0%	8	1.2%	1	0.99	0	0%	4	0.9%	1	1	0	0%	0	0%	1	0	0%	0	0%	1
MGA	0	0%	17	2.5%	1	0.90	0	0%	8	1.8%	1	0.98	0	0%	0	0%	1	0	0%	0	0%	1
MITF	0	0%	4	0.6%	1	1	0	0%	3	0.7%	1	1	0	0%	0	0%	1	0	0%	0	0%	1
MLH1	0	0%	6	0.9%	1	1	0	0%	4	0.9%	1	1	0	0%	1	5.9%	1	0	0%	0	0%	1
MPL	0	0%	2	0%	1	1	0	0%	1	0.2%	1	1	0	0%	0	0%	1	0	0%	0	0%	1
MRE11A	0	0%	5	0.7%	1	0.99	0	0%	4	0.9%	1	1	0	0%	0	0%	1	0	0%	0	0%	1
MSH2	0	0%	6	0.9%	1	1	0	0%	4	0.9%	1	1	0	0%	0	0%	1	0	0%	0	0%	1
MSH6	0	0%	8	1.2%	1	0.98	0	0%	3	0.7%	1	1	0	0%	0	0%	1	0	0%	0	0%	1
MST1	0	0%	1	0%	1	1	0	0%	1	0.2%	1	1	0	0%	0	0%	1	0	0%	0	0%	1
MST1R	0	0%	5	0.7%	1	1	0	0%	3	0.7%	1	1	0	0%	0	0%	1	0	0%	0	0%	1
MTOR	1	4.2%	15	2.2%	0.43	0.71	1	5.6%	10	2.3%	0.36	0.58	1	6.2%	0	0%	0.48	1	6.2%	2	12.5%	1
MUTYH	0	0%	2	0%	1	1	0	0%	2	0.5%	1	1	0	0%	0	0%	1	0	0%	0	0%	1
MYC	0	0%	1	0%	1	1	0	0%	0	0%	1	1	0	0%	1	5.9%	1	0	0%	0	0%	1
MYCL	1	4.2%	0	0%	0.03	0.25	1	5.6%	0	0%	0.04	0.25	1	6.2%	0	0%	0.48	1	6.2%	0	0%	1
MYCN	0	0%	0	0%	1	1	0	0%	0	0%	1	1	0	0%	0	0%	1	0	0%	0	0%	1
MYD88	1	4.2%	0	0%	0.03	0.25	1	5.6%	0	0%	0.04	0.25	0	0%	0	0%	1	0	0%	0	0%	1
MYOD1	0	0%	0	0%	1	1	0	0%	0	0%	1	1	0	0%	0	0%	1	0	0%	0	0%	1
NBN	0	0%	5	0.7%	1	0.99	0	0%	3	0.7%	1	1	0	0%	0	0%	1	0	0%	0	0%	1
NCOA3	0	0%	13	1.9%	1	0.94	0	0%	6	1.4%	1	1	0	0%	0	0%	1	0	0%	0	0%	1
NCOR1	1	4.2%	37	5.4%	1	0.92	1	5.6%	24	5.4%	1	0.84	1	6.2%	1	5.9%	1	1	6.2%	1	6.2%	1
NEGR1	0	0%	2	0%	1	1	0	0%	2	0.5%	1	1	0	0%	0	0%	1	0	0%	0	0%	1
NF1	1	4.2%	25	3.7%	0.60	0.85	1	5.6%	15	3.4%	0.48	0.71	1	6.2%	2	11.8%	1	1	6.2%	1	6.2%	1
NF2	0	0%	3	0%	1	1	0	0%	0	0%	1	1	0	0%	0	0%	1	0	0%	0	0%	1
NFE2L2	0	0%	4	0.6%	1	1	0	0%	3	0.7%	1	1	0	0%	0	0%	1	0	0%	0	0%	1
NFKBIA	1	4.2%	1	0%	0.07	0.27	1	5.6%	0	0%	0.04	0.25	1	6.2%	0	0%	0.48	1	6.2%	0	0%	1
NKX2-1	0	0%	0	0%	1	1	0	0%	0	0%	1	1	0	0%	0	0%	1	0	0%	0	0%	1
NKX3-1	0	0%	1	0%	1	1	0	0%	1	0.2%	1	1	0	0%	0	0%	1	0	0%	0	0%	1
NOTCH1	0	0%	7	1.0%	1	0.99	0	0%	3	0.7%	1	1	0	0%	0	0%	1	0	0%	0	0%	1
NOTCH2	0	0%	14	2.0%	1	0.96	0	0%	10	2.3%	1	0.95	0	0%	2	11.8%	0.48	0	0%	0	0%	1
NOTCH3	4	16.7%	4	0.6%	0.00007	0.007	3	16.7%	1	0.2%	0.0002	0.004	3	18.8%	0	0%	0.10	3	18.8%	0	0%	0.23
NOTCH4	0	0%	7	1.0%	1	1	0	0%	3	0.7%	1	1	0	0%	2	11.8%	0.48	0	0%	0	0%	1
NPM1	0	0%	0	0%	1	1	0	0%	0	0%	1	1	0	0%	0	0%	1	0	0%	0	0%	1
NRAS	1	4.2%	4	0.6%	0.16	0.35	1	5.6%	3	0.7%	0.15	0.32	1	6.2%	0	0%	0.48	1	6.2%	0	0%	1
NSD1	0	0%	9	1.3%	1	0.98	0	0%	5	1.1%	1	0.99	0	0%	1	5.9%	1	0	0%	0	0%	1
NTRK1	0	0%	7	1.0%	1	0.98	0	0%	4	0.9%	1	1	0	0%	0	0%	1	0	0%	0	0%	1

NTRK2	0	0%	5	0.7%	1	0.99	0	0%	3	0.7%	1	1	0	0%	0	0%	1	0	0%	1	6.2%	1
NTRK3	0	0%	8	1.2%	1	0.98	0	0%	3	0.7%	1	1	0	0%	0	0%	1	0	0%	0	0%	1
NUP93	0	0%	3	0%	1	1	0	0%	2	0.5%	1	1	0	0%	0	0%	1	0	0%	0	0%	1
PAK1	0	0%	4	0.6%	1	1	0	0%	2	0.5%	1	1	0	0%	0	0%	1	0	0%	0	0%	1
PAK7	0	0%	5	0.7%	1	1	0	0%	3	0.7%	1	1	0	0%	0	0%	1	0	0%	0	0%	1
PALB2	5	20.8%	4	0.6%	0.000003	0.002	5	27.8%	1	0.2%	0.0000003	0.001	5	31.2%	0	0%	0.02	5	31.2%	0	0%	0.04
PARK2	0	0%	5	0.7%	1	1	0	0%	3	0.7%	1	1	0	0%	0	0%	1	0	0%	0	0%	1
PARP1	0	0%	5	0.7%	1	1	0	0%	2	0.5%	1	1	0	0%	1	5.9%	1	0	0%	0	0%	1
PAX5	0	0%	4	0.6%	1	1	0	0%	1	0.2%	1	1	0	0%	0	0%	1	0	0%	0	0%	1
PBRM1	1	4.2%	8	1.2%	0.27	0.48	1	5.6%	4	0.9%	0.18	0.36	1	6.2%	1	5.9%	1	1	6.2%	0	0%	1
PDCD1	0	0%	3	0%	1	1	0	0%	3	0.7%	1	1	0	0%	0	0%	1	0	0%	0	0%	1
PDGFRA	0	0%	5	0.7%	1	1	0	0%	3	0.7%	1	1	0	0%	0	0%	1	0	0%	1	6.2%	1
PDGFRB	0	0%	7	1.0%	1	0.99	0	0%	7	1.6%	1	0.98	0	0%	0	0%	1	0	0%	0	0%	1
PDPK1	0	0%	2	0%	1	1	0	0%	2	0.5%	1	1	0	0%	0	0%	1	0	0%	0	0%	1
PGR	1	4.2%	4	0.6%	0.16	0.35	1	5.6%	3	0.7%	0.15	0.32	1	6.2%	0	0%	0.48	1	6.2%	0	0%	1
PHOX2B	0	0%	2	0%	1	1	0	0%	1	0.2%	1	1	0	0%	0	0%	1	0	0%	0	0%	1
PIK3C2G	0	0%	8	1.2%	1	0.97	0	0%	4	0.9%	1	1	0	0%	0	0%	1	0	0%	0	0%	1
PIK3C3	0	0%	5	0.7%	1	1	0	0%	3	0.7%	1	1	0	0%	0	0%	1	0	0%	0	0%	1
PIK3CA	7	29.2%	246	36.0%	0.67	0.77	7	38.9%	187	42.4%	0.81	0.81	3	18.8%	0	0%	0.10	3	18.8%	1	6.2%	0.60
PIK3CB	1	4.2%	7	1.0%	0.24	0.43	1	5.6%	5	1.1%	0.21	0.44	0	0%	1	5.9%	1	0	0%	0	0%	1
PIK3CD	0	0%	3	0%	1	1	0	0%	1	0.2%	1	1	0	0%	0	0%	1	0	0%	0	0%	1
PIK3CG	0	0%	5	0.7%	1	0.99	0	0%	1	0.2%	1	1	0	0%	0	0%	1	0	0%	0	0%	1
PIK3R1	0	0%	21	3.1%	1	0.82	0	0%	14	3.2%	1	0.89	0	0%	2	11.8%	0.48	0	0%	0	0%	1
PIK3R2	0	0%	3	0%	1	1	0	0%	2	0.5%	1	1	0	0%	0	0%	1	0	0%	0	0%	1
PIK3R3	0	0%	4	0.6%	1	1	0	0%	4	0.9%	1	1	0	0%	1	5.9%	1	0	0%	0	0%	1
PIM1	0	0%	1	0%	1	1	0	0%	0	0%	1	1	0	0%	0	0%	1	0	0%	0	0%	1
PLCG2	0	0%	5	0.7%	1	1	0	0%	2	0.5%	1	1	0	0%	0	0%	1	0	0%	1	6.2%	1
PLK2	0	0%	8	1.2%	1	0.98	0	0%	5	1.1%	1	1	0	0%	0	0%	1	0	0%	1	6.2%	1
PMAIP1	0	0%	0	0%	1	1	0	0%	0	0%	1	1	0	0%	0	0%	1	0	0%	0	0%	1
PMS1	0	0%	8	1.2%	1	0.98	0	0%	4	0.9%	1	1	0	0%	0	0%	1	0	0%	0	0%	1
PMS2	0	0%	5	0.7%	1	1	0	0%	2	0.5%	1	1	0	0%	0	0%	1	0	0%	0	0%	1
PNRC1	0	0%	1	0%	1	1	0	0%	1	0.2%	1	1	0	0%	0	0%	1	0	0%	0	0%	1
POLD1	0	0%	4	0.6%	1	1	0	0%	2	0.5%	1	1	0	0%	0	0%	1	0	0%	0	0%	1
POLE	0	0%	10	1.5%	1	0.99	0	0%	6	1.4%	1	0.97	0	0%	0	0%	1	0	0%	1	6.2%	1
PPM1D	0	0%	4	0.6%	1	1	0	0%	2	0.5%	1	1	0	0%	0	0%	1	0	0%	0	0%	1
PPP2R1A	0	0%	2	0%	1	1	0	0%	1	0.2%	1	1	0	0%	0	0%	1	0	0%	0	0%	1
PPP6C	0	0%	2	0%	1	1	0	0%	1	0.2%	1	1	0	0%	0	0%	1	0	0%	0	0%	1
PRDM1	0	0%	10	1.5%	1	0.95	0	0%	2	0.5%	1	1	0	0%	0	0%	1	0	0%	0	0%	1
PRKAR1A	0	0%	2	0%	1	1	0	0%	1	0.2%	1	1	0	0%	0	0%	1	0	0%	0	0%	1
PTCH1	0	0%	7	1.0%	1	0.98	0	0%	4	0.9%	1	1	0	0%	2	11.8%	0.48	0	0%	0	0%	1
PTEN	0	0%	42	6.1%	0.39	0.49	0	0%	26	5.9%	0.61	0.68	0	0%	3	17.6%	0.23	0	0%	1	6.2%	1
PTPN11	0	0%	2	0%	1	1	0	0%	2	0.5%	1	1	0	0%	0	0%	1	0	0%	0	0%	1
PTPRD	0	0%	19	2.8%	1	0.82	0	0%	12	2.7%	1	0.96	0	0%	1	5.9%	1	0	0%	0	0%	1
PTPRS	0	0%	4	0.6%	1	1	0	0%	2	0.5%	1	1	0	0%	0	0%	1	0	0%	0	0%	1
PTPRT	0	0%	7	1.0%	1	0.99	0	0%	5	1.1%	1	0.99	0	0%	0	0%	1	0	0%	0	0%	1
RAB35	0	0%	2	0%	1	1	0	0%	1	0.2%	1	1	0	0%	0	0%	1	0	0%	0	0%	1
RAC1	0	0%	0	0%	1	1	0	0%	0	0%	1	1	0	0%	0	0%	1	0	0%	0	0%	1
RAD21	0	0%	7	1.0%	1	0.98	0	0%	4	0.9%	1	1	0	0%	0	0%	1	0	0%	0	0%	1
RAD50	0	0%	7	1.0%	1	0.99	0	0%	5	1.1%	1	1	0	0%	0	0%	1	0	0%	0	0%	1
RAD51	0	0%	1	0%	1	1	0	0%	1	0.2%	1	1	0	0%	0	0%	1	0	0%	0	0%	1
RAD51B	0	0%	3	0%	1	1	0	0%	2	0.5%	1	1	0	0%	0	0%	1	0	0%	0	0%	1
RAD51C	0	0%	3	0%	1	1	0	0%	1	0.2%	1	1	0	0%	0	0%	1	0	0%	2	12.5%	0.48
RAD51D	0	0%	1	0%	1	1	0	0%	0	0%	1	1	0	0%	0	0%	1	0	0%	0	0%	1
RAD52	0	0%	0	0%	1	1	0	0%	0	0%	1	1	0	0%	0	0%	1	0	0%	0	0%	1
RAD54L	0	0%	4	0.6%	1	1	0	0%	2	0.5%	1	1	0	0%	0	0%	1	0	0%	0	0%	1
RAF1	0	0%	5	0.7%	1	1	0	0%	3	0.7%	1	1	0	0%	0	0%	1	0	0%	0	0%	1
RARA	0	0%	1	0%	1	1	0	0%	1	0.2%	1	1	0	0%	0	0%	1	0	0%	0	0%	1
RASA1	0	0%	4	0.6%	1	1	0	0%	2	0.5%	1	1	0	0%	0	0%	1	0	0%	0	0%	1
RB1	0	0%	16	2.3%	1	0.90	0	0%	6	1.4%	1	0.99	0	0%	0	0%	1	0	0%	3	18.8%	0.23
RBM10	0	0%	6	0.9%	1	0.99	0	0%	4	0.9%	1	1	0	0%	0	0%	1	0	0%	0	0%	1
RECQL4	1	4.2%	3	0%	0.13	0.32	0	0%	2	0.5%	1	0.30	1	6.2%	1	5.9%	1	1	6.2%	0	0%	1
REL	0	0%	1	0%	1	1	0	0%	1	0.2%	1	1	0	0%	0	0%	1	0	0%	0	0%	1
RET	0	0%	6	0.9%	1	1	0	0%	5	1.1%	1	1	0	0%	1	5.9%	1	0	0%	0	0%	1
RFWD2	0	0%	3	0%	1	1	0	0%	1	0.2%	1	1	0	0%	0	0%	1	0	0%	0	0%	1
RHEB	0	0%	1	0%	1	1	0	0%	0	0%	1	1	0	0%	0	0%	1	0	0%	0	0%	1
RHOA	0	0%	6	0.9%	1	0.99	0	0%	5	1.1%	1	1	0	0%	0	0%	1	0	0%	0	0%	1
RICTOR	0	0%	6	0.9%	1	1	0	0%	2	0.5%	1	1	0	0%	0	0%	1	0	0%	0	0%	1
RIT1	0	0%	0	0%	1	1	0	0%	0	0%	1	1	0	0%	1	5.9%	1	0	0%	0	0%	1
RNF43	0	0%	9	1.3%	1	0.98	0	0%	3	0.7%	1	1	0	0%	0	0%	1	0	0%	1	6.2%	1
ROS1	0	0%	14	2.0%	1	0.92	0	0%	11	2.5%	1	0.94	0	0%	2	11.8%	0.48	0	0%	0	0%	1
RPS6KA4	0	0%	4	0.6%	1	1	0	0%	4	0.9%	1	0.99	0	0%	0	0%	1	0	0%	0	0%	1
RPS6KB2	0	0%	4	0.6%	1	1	0	0%	2	0.5%	1	1	0	0%	0	0%	1	0	0%	0	0%	1
RPTOR	0	0%	13	1.9%	1	0.94	0	0%	5	1.1%	1	1	0	0%	1	5.9%	1	0	0%	0	0%	1
RUNX1	0	0%	33	4.8%	0.62	0.63	0	0%	25	5.7%	0.61	0.69	0	0%	0	0%	1	0	0%	0	0%	1
RYBP	0	0%	3	0%	1	1	0	0%	0	0%	1	1	0	0%	0	0%	1	0	0%	0	0%	1
SDHA	0	0%	3	0%	1	1	0	0%	2	0.5%	1	1	0	0%	0	0%	1	0	0%	0	0%	1
SDHAF2	0	0%	0	0%	1	1	0	0%	0	0%	1	1	0	0%	0	0%	1	0	0%	0	0%	1
SDHB	0	0%	2	0%	1	1	0	0%	2	0.5%	1	1	0	0%	0	0%	1	0	0%	0	0%	1
SDHC	0	0%	0	0%	1	1	0	0%	0	0%	1	1	0	0%	0	0%	1	0	0%	0	0%	1
SDHD	0	0%	0	0%	1	1	0	0%	0	0%	1	1	0	0%	0	0%	1	0	0%	0	0%	1
SETD2	0	0%	17	2.5%	1	0.89	0	0%	11	2.5%	1	0.96	0	0%	2	11.8%	0.48	0	0%	0	0%	1
SF3B1	0	0%	14	2.0%	1	0.94	0	0%	11	2.5%	1	0.94	0	0%	0	0%	1	0	0%	0	0%	1
SH2B3	0	0%	2	0%	1	1	0	0%	0	0%	1	1	0	0%	0	0%	1	0	0%	0	0%	1
SH2D1A	0	0%	3	0%	1	1	0	0%	3	0.7%	1	1	0	0%	0	0%	1	0	0%	0	0%	1
SHQ1	0	0%	2	0%	1	1	0	0%	0	0%	1	1	0	0%	0	0%	1	0	0%	0	0%	1
SMAD2	0	0%	5	0.7%	1	0.99	0	0%	4	0.9%	1	1	0	0%	0	0%	1	0	0%	0	0%	1
SMAD3	0	0%	3	0%	1	1	0	0%	2	0.5%	1	1	0	0%	1	5.9%	1	0	0%	0	0%	1
SMAD4	0	0%	7	1.0%	1	1	0	0%	6	1.4%	1	1	0	0%	0	0%	1	0	0%	0	0%	1
SMARCA4	0	0%	10	1.5%	1	0.96	0	0%	8	1.8%	1	0.98	0	0%	1	5.9%	1	0	0%	1	6.2%	1
SMARCB1	0	0%	1	0%	1	1	0	0%	0	0%	1	1	0	0%	0	0%	1	0	0%	0	0%	1
SMARCD1	0	0%	4	0.6%	1	1	0	0%	3	0.7%	1	1	0	0%	0	0%	1	0	0%	0	0%	1
SMO	0	0%	3	0%	1	1	0	0%	2	0.5%	1	1	0	0%	0	0%	1	0	0%	0	0%	1
SOCS1	0	0%	0	0%	1	1	0	0%	0	0%	1	1	0	0%	0	0%	1	0	0%	0	0%	1
SOX17	0	0%	0	0%	1	1	0	0%	0	0%	1	1	0	0%	0	0%	1	0	0%	0	0%	1
SOX2	0	0%	3	0%	1	1	0	0%	3	0.7%	1	1	0	0%	0	0%	1	0	0%	0	0%	1
SOX9	0	0%	6	0.9%	1	0.99	0	0%	3	0.7%	1	1	0	0%	0	0%	1	0	0%	0	0%	1
SPEN	1	4.2%	25	3.7%	0.60	0.83	0	0%	16	3.6%	1	0.74	1	6.2%	0	0%	0.48	1	6.2%	0	0%	1
SPOP	0	0%	3	0%	1	1	0	0%	3	0.7%	1	1	0	0%	1	5.9%	1	0	0%	0	0%	1
SRC	0	0%	1	0%	1	1	0	0%	1	0.2%	1	1	0	0%	0	0%	1	0	0%	0	0%	1
SRSF2	0	0%	3	0%	1	1	0	0%	2	0.5%	1	1	0	0%	0	0%	1	0	0%	0	0%	1
STAG2	1	4.2%	11	1.6%	0.34	0.54	0	0%	6	1.4%	1	0.46	0	0%	0	0%	1	0	0%	0	0%	1
STAT3	1	4.2%	7	1.0%	0.24	0.46	1	5.6%	4	0.9%	0.18	0.37	0	0%	0	0%	1	0	0%	0	0%	1
STAT5A	0	0%	6	0.9%	1	1	0	0%	3	0.7%	1	1	0	0%	0	0%	1	0	0%	0	0%	1
STAT5B	0	0%	4	0.6%	1	1	0	0%	1	0.2%	1	1	0	0%	0	0%	1	0	0%	0	0%	1
STK11	0	0%	1	0%	1	1	0	0%	1	0.2%	1	1	0	0%	1	5.9%	1	0	0%	0	0%	1
STK40	0	0%	3	0%	1	1	0	0%	3	0.7%	1	1	0	0%	0	0%	1	0	0%	0	0%	1
SUFU	0	0%	1	0%	1	1	0	0%	1	0.2%	1	1	0	0%	0	0%	1	0	0%	0	0%	1
SUZ12	0	0%	7	1.0%	1	1	0	0%	5	1.1%	1	1	0	0%	0	0%	1	0	0%	0	0%	1
SYK	0	0%	2	0%	1	1	0	0%	2	0.5%	1	1	0	0%	0	0%	1	0	0%	1	6.2%	1
TBX3	1	4.2%	24	3.5%	0.58	0.85	0	0%	21	4.8%	1	0.82	0	0%	0	0%	1	0	0%	0	0%	1
TCEB1	0	0%	1	0%	1	1	0	0%	0	0%	1	1	0	0%	0	0%	1	0	0%	0	0%	1
TCF3	0	0%	1	0%	1	1	0	0%	1	0.2%	1	1	0	0%	0	0%	1	0	0%	0	0%	1
TCF7L2	0	0%	4	0.6%	1	1	0	0%	2	0.5%	1	1	0	0%	0	0%	1	0	0%	0	0%	1
TERT	0	0%	6	0.9%	1	0.99	0	0%	4	0.9%	1	1	0	0%	0	0%	1	0	0%	0	0%	1
TET1	0	0%	8	1.2%	1	0.98	0	0%	5	1.1%	1	1	0	0%	1	5.9%	1	0	0%	1	6.2%	1
TET2	0	0%	9	1.3%	1	0.98	0	0%	5	1.1%	1	1	0	0%	1	5.9%	1	0	0%	0	0%	1
TGFBR1	0	0%	3	0%	1	1	0	0%	2	0.5%	1	1	0	0%	0	0%	1	0	0%	0	0%	1
TGFBR2	0	0%	3	0%	1	1	0	0%	3	0.7%	1	1	0	0%	0	0%	1	0	0%	0	0%	1
TMEM127	0	0%	1	0%	1	1	0	0%	1	0.2%	1	1	0	0%	0	0%	1	0	0%	0	0%	1
TMPRSS2	0	0%	2	0%	1	1	0	0%	2	0.5%	1	1	0	0%	0	0%	1	0	0%	0	0%	1
TNFAIP3	0	0%	4	0.6%	1	1	0	0%	2	0.5%	1	1	0	0%	0	0%	1	0	0%	0	0%	1
TNFRSF14	0	0%	2	0%	1	1	0	0%	0	0%	1	1	0	0%	0	0%	1	0	0%	0	0%	1
TOP1	0	0%	4	0.6%	1	1	0	0%	2	0.5%	1	1	0	0%	0	0%	1	0	0%	0	0%	1
TP53	5	20.8%	218	31.9%	0.37	0.50	1	5.6%	80	18.1%	0.22	0.40	3	18.8%	15	88.2%	0.000086	3	18.8%	2	12.5%	1
TP63	0	0%	4	0.6%	1	1	0	0%	4	0.9%	1	1	0	0%	0	0%	1	0	0%	0	0%	1
TRAF2	0	0%	2	0%	1	1	0	0%	0	0%	1	1	0	0%	0	0%	1	0	0%	0	0%	1
TRAF7	0	0%	7	1.0%	1	0.99	0	0%	4	0.9%	1	1	0	0%	0	0%	1	0	0%	0	0%	1
TSC1	0	0%	5	0.7%	1	1	0	0%	2	0.5%	1	1	0	0%	1	5.9%	1	0	0%	0	0%	1
TSC2	1	4.2%	4	0.6%	0.16	0.33	1	5.6%	2	0.5%	0.11	0.29	1	6.2%	1	5.9%	1	1	6.2%	0	0%	1
TSHR	0	0%	6	0.9%	1	0.99	0	0%	4	0.9%	1	1	0	0%	0	0%	1	0	0%	0	0%	1
U2AF1	0	0%	2	0%	1	1	0	0%	2	0.5%	1	1	0	0%	0	0%	1	0	0%	0	0%	1
VEGFA	0	0%	0	0%	1	1	0	0%	0	0%	1	1	0	0%	0	0%	1	0	0%	0	0%	1
VHL	0	0%	0	0%	1	1	0	0%	0	0%	1	1	0	0%	0	0%	1	0	0%	0	0%	1
VTCN1	0	0%	1	0%	1	1	0	0%	1	0.2%	1	1	0	0%	0	0%	1	0	0%	0	0%	1
WT1	0	0%	3	0%	1	1	0	0%	1	0.2%	1	1	0	0%	0	0%	1	0	0%	0	0%	1
XIAP	0	0%	0	0%	1	1	0	0%	0	0%	1	1	0	0%	0	0%	1	0	0%	0	0%	1
XPO1	0	0%	8	1.2%	1	0.98	0	0%	5	1.1%	1	1	0	0%	1	5.9%	1	0	0%	0	0%	1
XRCC2	0	0%	3	0%	1	1	0	0%	1	0.2%	1	1	0	0%	0	0%	1	0	0%	0	0%	1
YAP1	0	0%	1	0%	1	1	0	0%	1	0.2%	1	1	0	0%	0	0%	1	0	0%	0	0%	1
YES1	0	0%	2	0%	1	1	0	0%	2	0.5%	1	1	0	0%	0	0%	1	0	0%	0	0%	1
ZFHX3	1	4.2%	14	2.0%	0.41	0.61	0	0%	8	1.8%	1	0.54	1	6.2%	1	5.9%	1	1	6.2%	0	0%	1
ZRSR2	0	0%	0	0%	1	1	0	0%	0	0%	1	1	0	0%	0	0%	1	0	0%	0	0%	1
